# Intra-population genomic diversity of the bloom-forming cyanobacterium, *Aphanizomenon gracile*, at low spatial scale

**DOI:** 10.1038/s43705-023-00263-3

**Published:** 2023-06-07

**Authors:** Sébastien Halary, Sébastien Duperron, Sandra Kim Tiam, Charlotte Duval, Cécile Bernard, Benjamin Marie

**Affiliations:** 1 Muséum National d’Histoire Naturelle, CNRS, UMR7245 Mécanismes de Communication et Adaptation des Micro-organismes, 12 rue Buffon, 75005 Paris, France; 2 UMR5557 Laboratoire d’Ecologie Microbienne, Université de Lyon, 43 bd du 11 novembre 1918, Villeurbanne, F-69622 Lyon, France

**Keywords:** Biodiversity, Microbial communities

## Abstract

Cyanobacteria are oxygenic photosynthetic bacteria that perform a substantial part of the global primary production. Some species are responsible for catastrophic environmental events, called blooms, which have become increasingly common in lakes and freshwater bodies as a consequence of global changes. Genotypic diversity is considered essential for marine cyanobacterial population, allowing it to cope with spatio-temporal environmental variations and to adapt to specific micro-niches in the ecosystem. This aspect is underestimated in the study of bloom development, however, and given little notice in studies of the ecology of harmful cyanobacteria. Here we compared the genomes of four strains of *Aphanizomenon gracile*, a species of filamentous toxinogenic cyanobacteria (Nostocales) found worldwide in fresh and brackish water. Millimeter-sized fascicles were isolated from a single water sample and have been maintained in culture since 2010. A comparative study revealed extensive heterogeneity in gene contents, despite similar genome size and high similarity indices. These variations were mainly associated with mobile genetic elements and biosynthetic gene clusters. For some of the latter, metabolomic analysis confirmed the production of related secondary metabolites, such as cyanotoxins and carotenoids, which are thought to play a fundamental role in the cyanobacterial fitness. Altogether, these results demonstrated that an *A. gracile* bloom could be a highly diverse population at low spatial scale and raised questions about potential exchanges of essential metabolites between individuals.

## Introduction

The ecological success of cyanobacteria is partly a result of the intra-specific diversity of ecotypes [1–3], groups of individuals sharing close growth optima for environmental factors such as temperature and light intensity, which allow the population to cope with spatiotemporal variations in their habitat. Phylogenetic inferences based on single (*e.g.*, Internal Transcribed Spacer, ITS) or multiple genes allow to group these ecotype members within a clade, suggesting the selection for adaptative capabilities. In terms of gene content, however, the ecotypes can also show significant divergence from each other. For instance the gene sets involved in the phosphorus assimilation, an essential abiotic factor for cyanobacterial growth, diverge between ecotypes from P-rich or P-limited aquatic ecosystems [4–6]. For the well-studied cyanobacterial species *Prochlorochoccus marinus* (Synechococcales) and *Microcystis aeruginosa* (Chroococcales), a single ecotype can also contain many strains with distinct genotypes. This so-called microdiversity relies primarily in these species on a high genomic plasticity [7], driven in part by mobile genetic elements [7–9] and is assumed to be involved in individuals adaptation to specific micro-niches [10].

*Aphanizomenon gracile* is a nitrogen-fixing filamentous cyanobacterium able to form fascicles and is responsible for toxic blooms in freshwater and brackish aquatic ecosystems. It belongs to the order, Nostocales, about which no data are available on genetic intra-population diversity. In this study, we sequenced the genomes of four strains of *A. gracile* isolated from a single sample of water to question the intra-population diversity at low-spatial scale.

## Results and discussion

We sequenced and assembled the genomes of four *A. gracile* strains (PMC627.10, PMC638.10, PMC644.10 and PMC649.10) firstly isolated from a single sample of water and maintained in monoculture (see Supplementary Methods). The resulting genomes displayed almost-perfect completeness (from 99.18% for PMC649.10 to 100% for PMC638.10), but were composed of numerous sequences, between 65 (PMC627.10) and 238 (PMC644.10), because of a large number of repeated sequences. The four genomes were very similar in size (5.40 ± 0.03 Mb), GC content (38.35 ± 0.05%) and number of tRNAs (from 40 to 42) (Table 1). Each strain shared with the others a minimal sequence similarity of 99.86% with the 16S rRNA coding gene, while the ITS sequences were all identical (Fig. S1). At the whole genome level, the proximity among strains was supported by pairwise ANI (Average Nucleotide Identity, Table 1) with values ≥99.12%. Such a level of similarity usually leads to the conclusion that related organisms belong to the same operational taxonomic unit and by extension to the same ecotype as the phylogenetically distant marine unicellular pico-cyanobacterium, *Prochlorococcus marinus*, in which microdiversity has been thoroughly studied [11]. In the absence of physiological data confirming the ecotypes of the *A. gracile* strains, these findings allow us to interpret the internal variability of the *A. gracile* strains in a microdiversity context.

**Table 1:** Genomic features of *A. gracile* strains.

Strain	Size (Mb)	GC (%)	Completeness (%)	Contamination (%)	#Contigs	#tRNA	#CDS (Prodigal)	#COGs (pangenome = 5911)	#Singletons	Min. ANI (%)	Biosamples	Genbank
PMC627	5.40	38.4	99.73	0	65	41	5062	4908	305	99.12 (PMC649)	SAMN17438945	JAQSYX000000000
PMC638	5.37	38.4	100	0.06	162	40	4959	4843	192	99.14 (PMC649)	SAMN17438946	JAQSYY000000000
PMC644	5.44	38.3	99.73	0	238	41	4975	4878	205	99.20 (PMC627)	SAMN17438947	JAQSYZ000000000
PMC649	5.39	38.3	99.18	0	208	42	4953	4859	217	99.12 (PMC627)	SAMN17438948	JAQSZA000000000

**Fig. 1:**
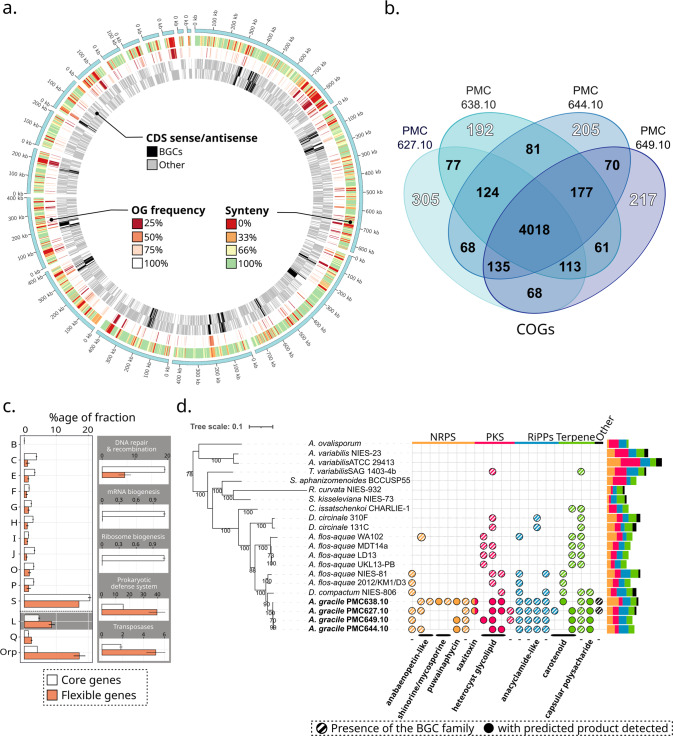
Comparative genomics between the four *A. gracile* strains. **a** Representation of the PMC627.10 *A. gracile* genome displaying from the inner to the outer circle: (i) the coding DNA sequence (CDS) on both strands with biosynthetic gene clusters (BGCs) in black, (ii) frequency (%) of CDS-associated clusters of orthologous genes (COGs) in the four *A. gracile* genomes, (iii) the synteny index, and (iv) the largest (>10kb) assembled scaffolds representing >99% of the genome. **b** Venn diagram of the distribution of COGs and singletons with white labels. **c** Percentage of COG functional categories with significant differences (Student’s *t* test with *p* < 0.01) between core (white) and flexible (red) gene sets, including a focus on the “Replication, Recombination and Repair” COG category (L). COG categories: B-“Chromatin structure/dynamics", C-“Energy production/conversion", E-“Amino acid metabolism/transport", F-“Nucleotide metabolism/transport", G-“Carbohydrate metabolism/transport", H-“Coenzyme metabolism", I-“Lipid metabolism", J-“Translation", O-“Post-translational modification/protein turnover/chaperone functions", P-“Inorganic ion transport/metabolism", Q-“Secondary metabolism", and S-“Function unknown". **d** Presence or absence of BGCs (COG category Q) among *A. gracile* strains and closed Nostocales taxa gathered by BGC types (‘-‘, uncharacterized product). The solid circles indicate BGCs whose products have been formally identified by mass spectrometry. Left: Phylogenomic tree (see Supplementary Methods and Table S1 for the list of genes used). Right: histogram displaying the number of BGCs by strain colored by BGC type. NRPS/PKS non-ribosomal peptide synthetase/polyketide synthetase, RiPPs ribosomally synthesized, post-translationally modified peptides, terpenes and others (uncharacterized). The lists of BGC and analytes detected in each strain can be found in Supplementary Tables S2 and S3, respectively.

Despite the absence of self-evident differences between genetic markers, comparative genomics revealed divergences among the strains. A striking representation of this is given by the low synteny index of 77.01 ± 1.31%, similar to those reported among 12 *Microcystis aeruginosa* strains sampled from distant geographical locations (76 ± 4%) [7]. As exemplified for PMC627.10, the variations of the synteny index along the genome were significantly associated with the COGs frequency distribution (Pearson’s chi-squared test, *p* < 2.2e^−16^) meaning that the low synteny was due to the presence or absence of strain-specific genes rather than intra-genomic rearrangements (Fig. 1a). In total, among the 5911 COGs predicted in all genomes (4972 ± 28 per strain; Table 1), the core genome was represented by only 67.97% of them (4018) (Fig. 1b). The accessory gene fraction consisted of almost half (48.54%) singletons, *i.e.*, strain-specific genes (between 192 and 305 by genome). The latter fraction was significantly enriched in genes encoding prokaryotic defense systems and transposases. These subcategories of “Replication, Recombination and Repair” (COG category L) as well as Orphans (*t* test H_0_: no difference, *p* < 0.01) are usually considered as markers of mobile genetic elements, here potentially involved in genomic plasticity in *Aphanizomenon*. Remarkably, COGs related to the biosynthesis of secondary metabolites (COG category Q) were also enriched in accessory fractions (Fig. 1c).

The study of *A. gracile* secondary metabolite biosynthetic gene clusters (BGC) revealed that their repertoires were unique to each *A. gracile* strain, with only 8 BGCs common to all strains out of a total of 22 distinct BGC characterized in these genomes (Fig. 1d). Although variable, the composition of this BGC set appeared to be clade-associated in the Nostocales tree, as the number of BGCs in common with *A. gracile* genomes decreased with increasing distance from their clade in the phylogenetic tree. Accessory BGCs were notably involved in the production of carotenoids and shinorines, both implicated in several adaptative processes including photoprotection [12, 13], and cyanotoxins (*e.g.* puwainaphycins and saxitoxins), whose production in the associated cultures was confirmed by high-resolution mass spectrometry (Fig. S2, Table S3). Although not-well characterized, cyanotoxins are thought to play key roles in adaptation, given the high cost of BGC maintenance in genomes and resource requirements for their production. For example, saxitoxins, which were only synthesized in our study by PMC 627.10 and PMC 638.10 strains from synthesized from their 26.5kb long BGC, are presumed to regulate Na^+^ homeostasis and have been proved to be actively extracellularly exported in salinity stress conditions [14].

Lastly, the group of accessory genes with resolutive functional annotations at the gene name level, revealed potential discrepancies in important metabolic pathways, such as sulfur metabolism and regulation of photosynthesis, although they did not belong to the COG categories enriched in the flexible fraction (Supplementary Results and Table S4).

After ten years in culture, genetic drift could be responsible to some extent for the observed genomic diversity among strains (e.g., [15]). However, because the number of proteins in the pangenome is much higher than that of individual *A. gracile* strains, which is constrained by their genome size, this diversity probably reflects true genomic diversity in the natural population, as has already been observed at the level of individual marker genes, such as sxtA [16]. Our results lead to the conclusion that the intra-population micro-diversity could be a feature largely shared within the cyanobacterial phylum. On the one hand, the presence of accessory genes related to the regulation of photosynthesis and sulfur metabolism implies that many strains with diverse adaptive capabilities can thrive until bloom development and co-occur at low spatial scale. On the other hand, the inter-strain variability in the production of essential secondary metabolites for which biosynthetic genes are especially enriched in accessory gene fraction, suggests that some of these molecules could be shared to the surrounding cyanobacteria by the producing strains, allowing the population to benefit from their adaptive functions while limiting the resource requirements for individuals. Thus, evaluation of the extent of biodiversity within a blooming cyanobacteria population could be a cornerstone for the establishment of a holistic theoretical framework of the dynamics of toxinogenic cyanobacteria blooms.

## Supplementary information

Supplementary Methods

Supplementary Results

Table S1

Table S2

Table S3

Table S4

## Data Availability

PMC strains are available upon request (https://mcam.mnhn.fr/en/cyanobacteria-and-live-microalgae-1281). Raw reads were deposited into the GENBANK Sequence Read Archive (SRA) database under the BioProject PRJNA693796 as well as *A. gracile* genome assemblies (JAQSYX000000000, JAQSYY000000000, JAQSYZ000000000, JAQSZA000000000).
