# Supplementary material for: Intra-population genomic diversity of the bloom-forming cyanobacterium, Aphanizomenon gracile, at low spatial scale
Source: ISME Commun. 2023 Jun 7;3:57. doi: 10.1038/s43705-023-00263-3 (PMC10244403; doi:10.1038/s43705-023-00263-3)
Supplement: Supplementary file 1 — Supplementary Methods [file 43705_2023_263_MOESM1_ESM.docx]

**Methods**

**Cyanobacteria isolation and culture conditions**

Cyanobacteria were collected during a bloom at the Champs-sur-Marne (France) recreational water body in 2010. Each strain was isolated from a distinct fascicle, all present in the same sample of water, as part of a previous study (1). Strains were maintained in monoclonal and non-axenic cultures at the Paris Museum Collection (PMC) at 25°C, using daylight fluorescent tubes providing an irradiance level of 16 μmol photons.cm^-2^.s^-1^, with a photoperiod of 16h:8h light:dark, in 10 mL of liquid Z8X media (2). Among the strains, 23 have been identified as *Aphanizomenon gracile*, on morphological and phylogenetic criteria (1), and 4 were chosen for the present study only based on their ability to produce saxitoxin (SXT) detected in culture by mass spectrometry (see paragraph below), namely PMC 627.10, PMC 638.10, (both producing SXT), and PMC 644.10 and PMC 649.10 (non-producing SXT).

**Metabolite content characterization by high-resolution mass spectrometry**

Metabolites were extracted from strains cellular biomass using 0.75% methanol (100 μL for 1 mg dry mass). Ultra High Performance Liquid Chromatography (UHPLC) was performed on 2 μL of each of the metabolite extracts using a Polar Advances II 2.5-μm pore C_18_ column (Thermo®) at a 300 μL.min^-1^ flow rate with a linear gradient of acetonitrile in 0.1% formic acid (5 to 90% in 21 min). The metabolite contents were analysed in triplicates using an electrospray ionization hybrid quadrupole time-of-flight (ESI-QqTOF) high resolution mass spectrometer (Maxis II ETD, Bruker) on positive autoMSMS mode with information dependent acquisition (IDA), on the 50-1 500 m/z rang at 2 Hz or between 2-8 Hz speed, for MS and MS/MS respectively, according to relative intensity of parent ions, in consecutive cycle times of 2.5 s, with an active exclusion of previously analysed parents. The data were analysed with MetaboScape 4.0 software for internal recalibration (<0.5 ppm), molecular feature search and MGF export. Peak lists were generated from MS/MS spectra between 1 and 15 min, with a filtering noise threshold at 0.1% maximal intensity and combining various charge states and related isotopic forms. A molecular network then was created using the online workflow at Global Natural Products Social molecular networking (GNPS) (http://gnps.ucsd.edu)(3) as previously described (4). The clustered spectra of the network were annotated by comparing monoisotopic mass to our in-house cyanobacteria metabolite databases according to MS and MS/MS fragmentation pattern matches.

**DNA extraction, sequencing and metagenomes assembly**

DNA was extracted in April 2019 using a ZymoBIOMICS DNA mini kit (Zymo Research, CA) from cyanobacterial strains grown in flasks. Mechanical lysis was carried out using an ultrasonic cell probe Vibra Sonic (Granuloshop) for 30 s at a range of 100 % to 32.5 W. Total DNA was sequenced using both Illumina MiSeq 2x250bp and SMRT cell PacBio RS2 platforms (Genoscreen, France). Scaffolds were assembled from MiSeq and Pacbio reads using SPAdes-based Unicycler hybrid-assembler, with default parameters, for the metagenome of each individual strain culture (5, 6). Nodes from assembly graphs were clustered using MyCC (7) with default parameters, required scaffold coverages being determined by bowtie2 (8) and samtools (9), and taxonomically annotated using CAT (10). 16S rRNA-encoding genes were extracted using Metaxa2 (11) and annotated using ACT (12). Congruent results between these methodologies allowed to extract Metagenome Assembled Genomes (MAGs). Genomes completeness and contamination were assessed using CheckM (13). Furthermore, *A. gracile* MAGs were used as database which all Illumina reads were mapped against using BBmap. For each strain, these reads were used to perform another hybrid assembly as described above. Again, CAT was used to confirm cyanobacterial annotations of each new scaffold.

***A. gracile* comparative genomics**

GC statistics were calculated using custom python scripts. Open Reading Frames (ORF) were predicted using Prodigal (14), and genes encoding rRNA and tRNA were detected using Barrnap (15) and Aragorn (16), respectively. Internal Transcribed Spacer (ITS) sequences were manually extracted using 16S and 23S rRNA-encoding genes coordinates. Clusters of Orthologous genes (COG) were defined using OrthoFinder (E-value≤1E^-05^, identity≥70%) (17). OG frequency was calculated as the proportion of *A. gracile* genome possessing at least one member of a COG.

Given the difficulty for some cyanobacterial genomes to assemble due to numerous repetitive sequences, the presence/absence of genes was double-checked by mapping each set of *A. gracile reads* to the pangenome. For this, cyanobacterial reads from a single tested strain (see section above for details) were mapped to the other three genomes using bowtie2 (default settings, REF). The coverage of each CDS was then calculated using samtools (9) and a custom perl script. All CDS with a minimum coverage of 1x over at least 50% of the CDS length were then considered present in the genome of the tested strain.

Synteny index for each genome position was then calculated based on COG triplets using custom perl scripts. Relationship between OG frequency and synteny index was studied using a Pearson’s Chi-square test (H_0_: independence hypothesis, p-value<0.01). *A. gracile* core genome was defined as constituted by all COGs with an OG frequency of 100%, all other COGs and singletons being considered as belonging to flexible genes set. Functional comparison between these both fractions was performed using Student tests (H_0_: no difference, p-value<0.01). Functional annotations were obtained using eggnog-mapper with default parameters (18). Genome maps were generated using Circos (19).

**Nostocales phylogenomics and BGC distribution**

All available reference genomes of Nostocales (52) were retrieved from NCBI. After ORF prediction by Prodigal, Roary was used to defined the core genome of Nostocales including *A. gracile* genomes, and an outgroup constituted by *Prochlorococcus marinus* str. LG, str. MIT 9123, bv. HNLC2 and *Microcystis aeruginosa* DA14, S633 and PCC 9432. An alignment of 23 common orthologous genes (Table S1) were refined using BGME, and used to perform a phylogenomic inference using RaxML v8.2.12 (GTR model, 100 bootstraps) (20). Biosynthetic gene clusters were retrieved from genomes using AntiSmash v6.1.1 tool (21), except for saxitoxin gene clusters that have been additionally searched and confirmed after specific Blast search with *sxt*A using the MicroScope platform (22). BGC were compared using BigScape v.1.1.5, grouping BGC displaying >=30% homology.

**References**

1. Ledreux A, Thomazeau S, Catherine A, Duval C, Yéprémian C, Marie A, Bernard C. 2010. Evidence for saxitoxins production by the cyanobacterium *Aphanizomenon gracile* in a French recreational water body. Harmful Algae 10:88–97.

2. Rippka R. 1988. Isolation and purification of cyanobacteria. Methods Enzym 167:3–27.

3. Wang M, Carver JJ, Phelan VV, Sanchez LM, Garg N, Peng Y, Nguyen DD, Watrous J, Kapono CA, Luzzatto-Knaan T, Porto C, Bouslimani A, Melnik AV, Meehan MJ, Liu W-T, Crüsemann M, Boudreau PD, Esquenazi E, Sandoval-Calderón M, Kersten RD, Pace LA, Quinn RA, Duncan KR, Hsu C-C, Floros DJ, Gavilan RG, Kleigrewe K, Northen T, Dutton RJ, Parrot D, Carlson EE, Aigle B, Michelsen CF, Jelsbak L, Sohlenkamp C, Pevzner P, Edlund A, McLean J, Piel J, Murphy BT, Gerwick L, Liaw C-C, Yang Y-L, Humpf H-U, Maansson M, Keyzers RA, Sims AC, Johnson AR, Sidebottom AM, Sedio BE, Klitgaard A, Larson CB, P CAB, Torres-Mendoza D, Gonzalez DJ, Silva DB, Marques LM, Demarque DP, Pociute E, O’Neill EC, Briand E, Helfrich EJN, Granatosky EA, Glukhov E, Ryffel F, Houson H, Mohimani H, Kharbush JJ, Zeng Y, Vorholt JA, Kurita KL, Charusanti P, McPhail KL, Nielsen KF, Vuong L, Elfeki M, Traxler MF, Engene N, Koyama N, Vining OB, Baric R, Silva RR, Mascuch SJ, Tomasi S, Jenkins S, Macherla V, Hoffman T, Agarwal V, Williams PG, Dai J, Neupane R, Gurr J, Rodríguez AMC, Lamsa A, Zhang C, Dorrestein K, Duggan BM, Almaliti J, Allard P-M, Phapale P, Nothias L-F, Alexandrov T, Litaudon M, Wolfender J-L, Kyle JE, Metz TO, Peryea T, Nguyen D-T, VanLeer D, Shinn P, Jadhav A, Müller R, Waters KM, Shi W, Liu X, Zhang L, Knight R, Jensen PR, Palsson BO, Pogliano K, Linington RG, Gutiérrez M, Lopes NP, Gerwick WH, Moore BS, Dorrestein PC, Bandeira N. 2016. Sharing and community curation of mass spectrometry data with Global Natural Products Social Molecular Networking. Nat Biotechnol 34:828–837.

4. Kim Tiam S, Gugger M, Demay J, Le Manach S, Duval C, Bernard C, Marie B. 2019. Insights into the Diversity of Secondary Metabolites of Planktothrix Using a Biphasic Approach Combining Global Genomics and Metabolomics. Toxins 11.

5. Bankevich A, Nurk S, Antipov D, Gurevich AA, Dvorkin M, Kulikov AS, Lesin VM, Nikolenko SI, Pham S, Prjibelski AD, Pyshkin AV, Sirotkin AV, Vyahhi N, Tesler G, Alekseyev MA, Pevzner PA. 2012. SPAdes: a new genome assembly algorithm and its applications to single-cell sequencing. J Comput Biol 19:455–77.

6. Wick RR, Judd LM, Gorrie CL, Holt KE. 2017. Unicycler: Resolving bacterial genome assemblies from short and long sequencing reads. PLoS Comput Biol 13:e1005595.

7. Lin HH, Liao YC. 2016. Accurate binning of metagenomic contigs via automated clustering sequences using information of genomic signatures and marker genes. Sci Rep 6:24175.

8. Langmead B, Salzberg SL. 2012. Fast gapped-read alignment with Bowtie 2. Nat Methods 9:357–9.

9. Li H, Handsaker B, Wysoker A, Fennell T, Ruan J, Homer N, Marth G, Abecasis G, Durbin R. 2009. The Sequence Alignment/Map format and SAMtools. Bioinformatics 25:2078–9.

10. von Meijenfeldt FAB, Arkhipova K, Cambuy DD, Coutinho FH, Dutilh BE. 2019. Robust taxonomic classification of uncharted microbial sequences and bins with CAT and BAT. Genome Biol 20:217.

11. Bengtsson-Palme J, Hartmann M, Eriksson KM, Pal C, Thorell K, Larsson DG, Nilsson RH. 2015. METAXA2: improved identification and taxonomic classification of small and large subunit rRNA in metagenomic data. Mol Ecol Resour 15:1403–14.

12. Pruesse E, Peplies J, Glockner FO. 2012. SINA: accurate high-throughput multiple sequence alignment of ribosomal RNA genes. Bioinformatics 28:1823–9.

13. Parks DH, Imelfort M, Skennerton CT, Hugenholtz P, Tyson GW. 2015. CheckM: assessing the quality of microbial genomes recovered from isolates, single cells, and metagenomes. Genome Res 25:1043–55.

14. Hyatt D, Chen GL, Locascio PF, Land ML, Larimer FW, Hauser LJ. 2010. Prodigal: prokaryotic gene recognition and translation initiation site identification. BMC Bioinformatics 11:119.

15. Seemann T. 2020. Bacterial ribosomal RNA predictor. Perl.

16. Laslett D, Canback B. 2004. ARAGORN, a program to detect tRNA genes and tmRNA genes in nucleotide sequences. Nucleic Acids Res 32:11–16.

17. Emms DM, Kelly S. 2019. OrthoFinder: phylogenetic orthology inference for comparative genomics. Genome Biol 20:238.

18. Huerta-Cepas J, Szklarczyk D, Heller D, Hernandez-Plaza A, Forslund SK, Cook H, Mende DR, Letunic I, Rattei T, Jensen LJ, von Mering C, Bork P. 2019. eggNOG 5.0: a hierarchical, functionally and phylogenetically annotated orthology resource based on 5090 organisms and 2502 viruses. Nucleic Acids Res 47:D309–D314.

19. Krzywinski M, Schein J, Birol I, Connors J, Gascoyne R, Horsman D, Jones SJ, Marra MA. 2009. Circos: an information aesthetic for comparative genomics. Genome Res 19:1639–45.

20. RAxML version 8: a tool for phylogenetic analysis and post-analysis of large phylogenies | Bioinformatics | Oxford Academic. https://academic.oup.com/bioinformatics/article/30/9/1312/238053. Retrieved 7 November 2022.

21. Blin K, Shaw S, Steinke K, Villebro R, Ziemert N, Lee SY, Medema MH, Weber T. 2019. antiSMASH 5.0: updates to the secondary metabolite genome mining pipeline. Nucleic Acids Res 47:W81–W87.

22. Médigue C, Calteau A, Cruveiller S, Gachet M, Gautreau G, Josso A, Lajus A, Langlois J, Pereira H, Planel R, Roche D, Rollin J, Rouy Z, Vallenet D. 2017. MicroScope—an integrated resource for community expertise of gene functions and comparative analysis of microbial genomic and metabolic data. Brief Bioinform 20:1071–1084.
