# Supplementary material for: Intra-population genomic diversity of the bloom-forming cyanobacterium, Aphanizomenon gracile, at low spatial scale
Source: ISME Commun. 2023 Jun 7;3:57. doi: 10.1038/s43705-023-00263-3 (PMC10244403; doi:10.1038/s43705-023-00263-3)
Supplement: Supplementary file 2 — Supplementary Results [file 43705_2023_263_MOESM2_ESM.docx]

**Supplementary Results**

**SSU ribosomal RNA encoding gene locus diversity**

**
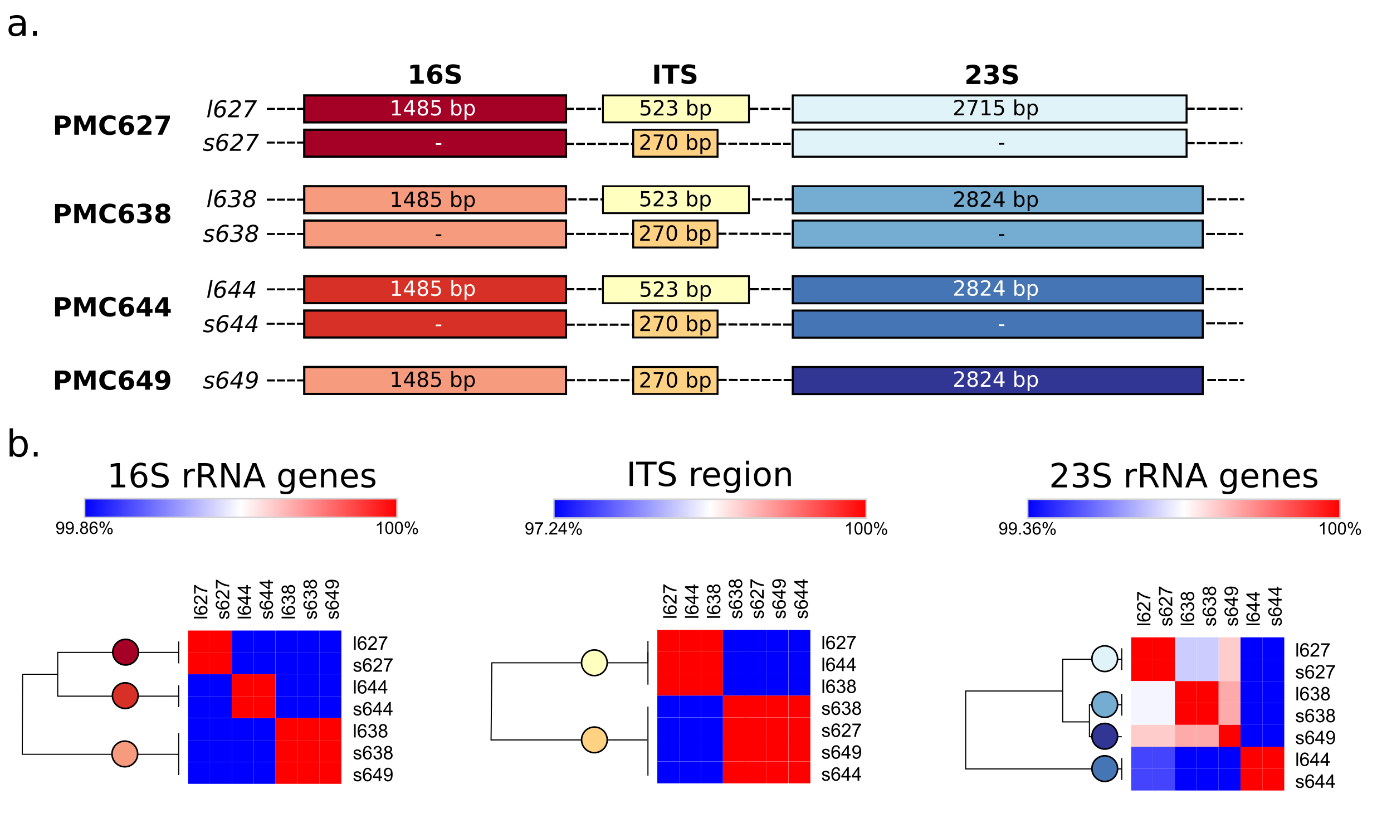
**

**Figure S1.** (**a**) The rrn operon structure in *A. gracile* strains. (**b**) Similarity of 16S rRNA genes among strains, ITS and 23S rRNA genes.

One or two types of *rrn* operons have been found within a genome corresponding to two forms of ITS sequences, a short one (*s*, 270 bp) and a long one (*l*, 523 bp). The long ITS has only been characterized in PMC627.10, PMC638.10 and PMC644.10 genomes (**Fig. S1a**). The 16S rRNA sequences, with a conserved size of 1485 bp, were identical between PMC638.10 and PMC649.10, but displayed a similarity of 99.86% in the other cases, corresponding to substitutions of two nucleotides (**Fig. S1b**). The 23S rRNA sequences were strain specific, with a similarity varying from 99.36 % to 99.78 % between the two strains, and a size of 2715 bp for PMC627.10 and 2824bp for the three other genomes. Both ITS types shared 97.24 % similarity on their aligned regions, and each was 100% identical among strains.

**Functional differences between core and flexible gene fractions**

In total, 18,237 genes (92.85%) were associated with an orthologous group in the EggNog database (4559.25 ± 55.82 by genome), including 53.11% with a functional annotation. The percentage of orphans differed between the core and flexible gene fractions, with 4.66 ± 0.05% and 16.77 ± 1.53 respectively (*p*<0.01, **Fig. 1c**). Significant differences were also found for several functional COG categories between both fractions (*p*<0.01). Genes related to energy production and conversion (C), amino acid transport and metabolism (E), carbohydrate transport and metabolism (G), coenzyme transport and metabolism (H), translation/ribosomal structure and biogenesis (J), post-translational modifications (O) and inorganic ion transport and metabolism (P), were over-represented in the core-genome compared to the flexible fraction. The genes implicated in replication, recombination and repair (L) and secondary metabolite biosynthesis, transport and catabolism (Q) represented a significantly higher proportion of genes in the flexible fraction than in the core-genome. A higher percentage of L-related genes in flexible fractions was associated with a significantly higher percentage of prokaryotic defense system genes and transposases while housekeeping functions related to DNA repair and recombination, messenger RNA biosynthesis and ribosome biogenesis were under-represented compared to the core-genome.

To study the potential metabolic impact of the flexible fraction beyond the enrichment in functional categories, accessory genes that were identified after functional annotation and did not have paralogs in the core genome are listed in table S4. Only 35 genes met these criteria, as the annotation of most accessory COGs (98.15%) did not receive sufficient resolution. In addition to those associated with ‘replication, recombination and repair’ (COG category L), 11 other categories were represented, of which the most frequent was ‘cell wall/membrane/envelope biogenesis” (M). Interestingly, in the flexible fraction, the genes, ssuB, ssuC and ssuD involved in the response to sulfide starvation conditions, were only present in the PMC627.10 genome. Similarly, the genes nblR, rpaB and phoX, which were transcriptional regulators (first two) and an accessory protein of photosystem I, respectively, were all involved in the response to high light intensities and distributed between PMC627.10, PMC638.10 and PMC644.10. These results suggest that adaptation to environmental stress could partly depend on the presence or absence of accessory genes, and that the metabolic responses could, therefore, be diverging within the *A. gracile* population.

**Metabolomic profiles characterization**

The high potential of secondary metabolite production was confirmed by direct metabolomic analysis of biomass extracts by high resolution mass spectrometry (HRMS) and visualization as molecular networks (**Fig. 3a-b**), allowing for instances of the detection of high levels of saxitoxin and neosaxitoxin in both PMC627 and PMC638 strains (**Fig. S1a**). The global molecular network performed by GNPS further highlights the production of a large set of metabolites by the four strains (1400 analytes), including the annotated ones shown in figure S1b. It also reveals the production of various uncharacterized variants of puwainaphycins in the three strains, specifically present as the puwainaphycin gene clusters, namely PMC638.10, PMC644.10 and PMC649.10. Among the 1400 detected analytes, only 64 (4.57%) were synthesized in all cultures (**Fig. S1c**). The great majority of them (75.14%) were specific to a single strain, illustrating the large molecular diversity in the metabolome of the four strains beyond their genomic similarities and differences.

**
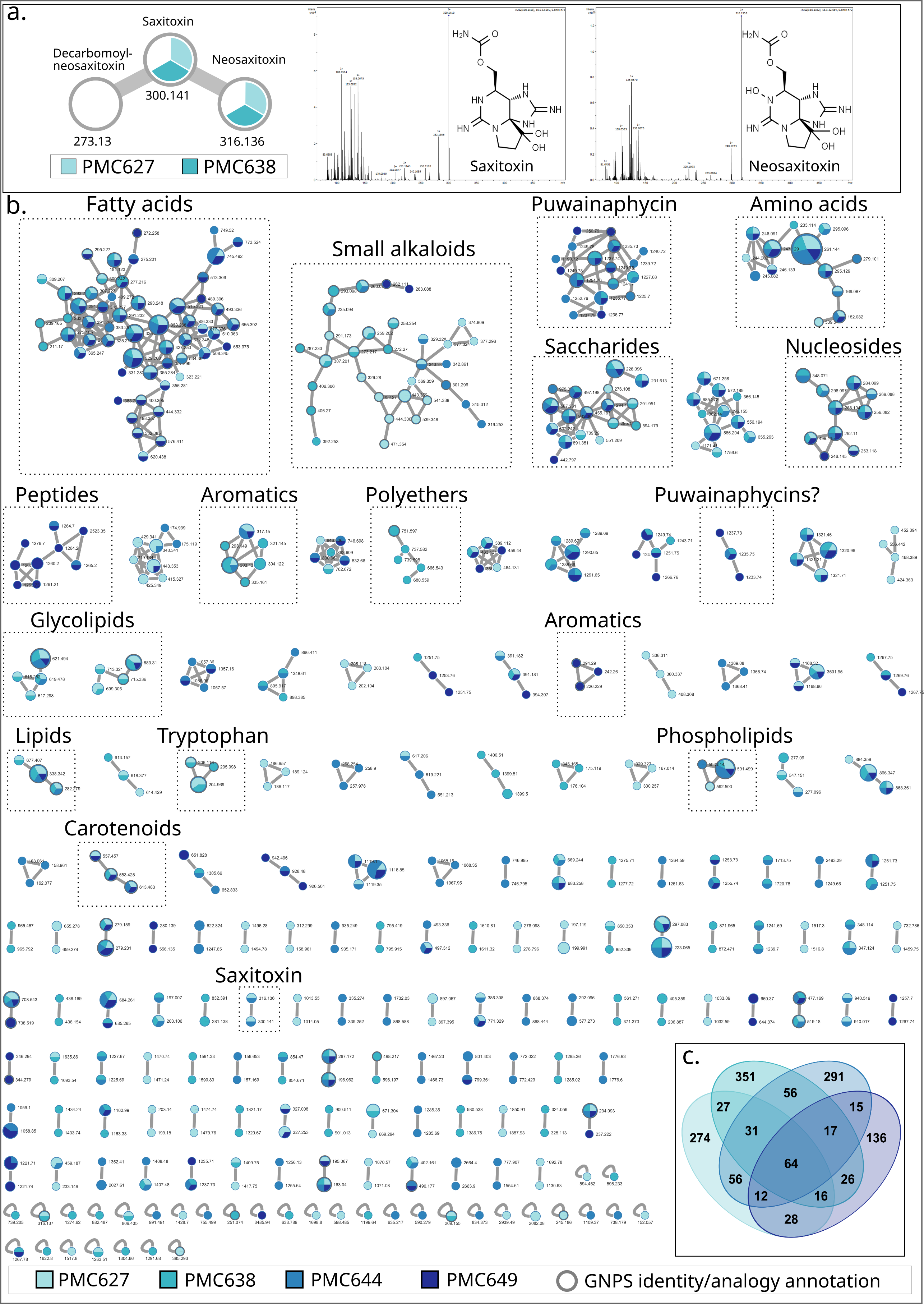
**

**Figure S2. Metabolomic analysis.** (**a**) Saxitoxin and neosaxitoxin production in PMC627.10 and PMC638.10. On the left, an analyte subnetwork performed by GNPS is connected by analogy to the standard analyte decarbomoyl-neosaxitoxin. On the right, the MS/MS spectra of these analytes corresponds to those of saxitoxin in agreement with the *sxt* gene clusters characterized in their respective genomes. (**b**) The global molecular network of the four *Aphanizomenon* strains reveals the presence of various metabolite families and also illustrates the molecular diversity within these strains. (**c**) Venn diagram of analytes detected in all *A. gracile* strains.

**BGC content variability**

Remarkably, the repertoires of biosynthetic gene clusters (BGCs) involved in secondary metabolite production were unique to each *A. gracile* strain (**Fig. 1d**). The *A. gracile* BGCs are notably involved in the production of saxitoxin (PMC627.10 and 638.10), puwainaphycins (PMC638.10, 644.10 and 649.10), anabaenopeptolins (PMC627.10, 638.10 and 649.10), anacyclamides (all strains), among other secondary metabolites. The BGCs can differ slightly in size and gene composition among strains, as for instance in the Sxt cluster responsible for the saxitoxin biosynthesis (**Fig. S3**). The Sxt clusters from PMC 627.10 and PMC 638.10 had 99% nucleotide identity and similar size. The largest difference is the presence in PMC627.10 of the sxtK gene and its absence from the PMC638.10 cluster. The size of these clusters is also identical to that described in other *A. gracile* strains such as NIVA-CYA 676 (1), despite the presence of an additional transposase between sxtPER and sxtC in this strain, and of the sxtJ gene, previously described as a core SXT cluster gene in *A. gracile* (1). These results confirm the versatility of BGC sequences, and suggest that this can occur within a species and within a population.
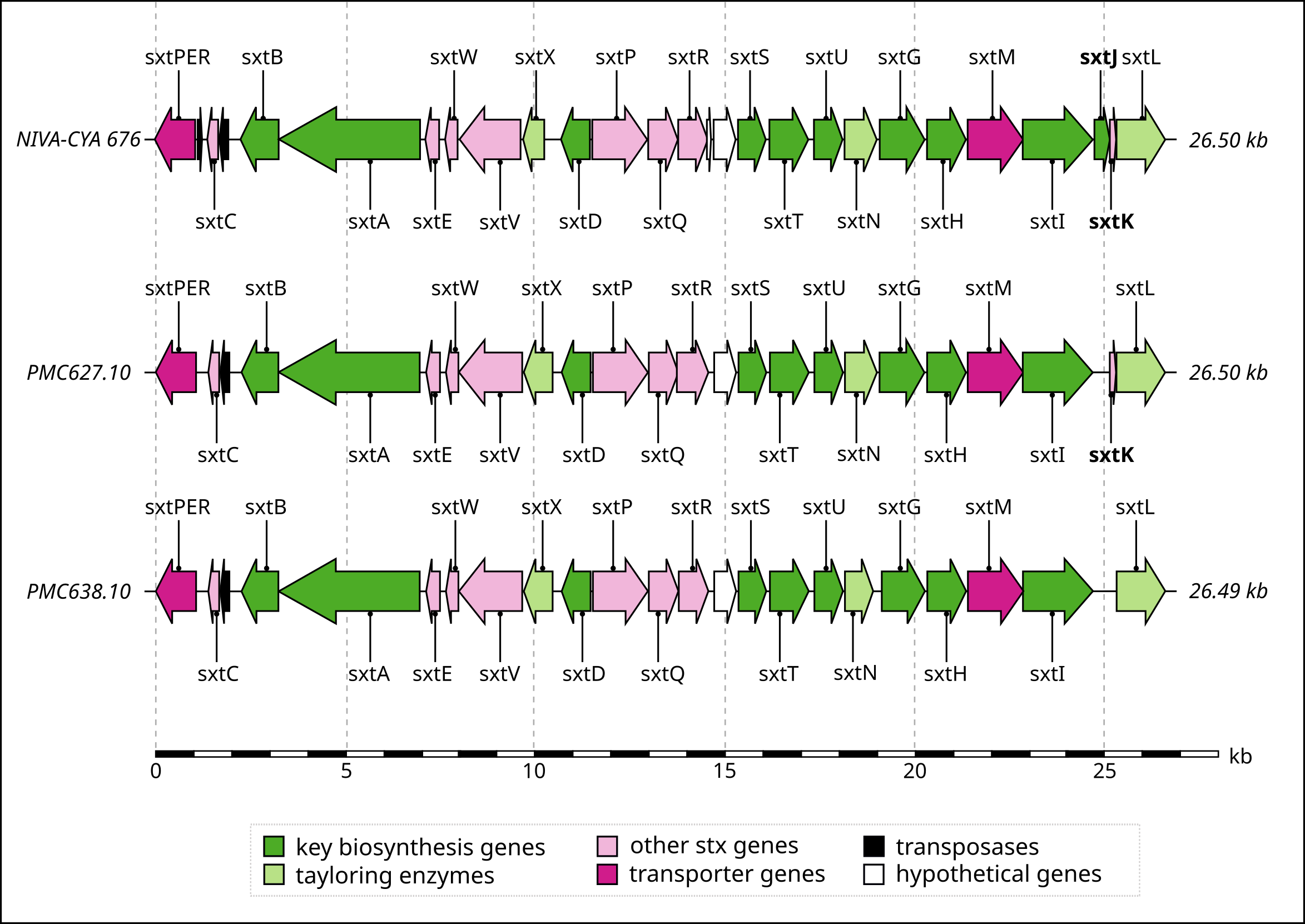


**Figure S3.** Saxitoxin biosynthetic gene cluster in PMC627.10 and PMC638.10 strains compared to the cluster of A. gracile NIVA-CYA 676.
