# Supplementary material for: Intra-population genomic diversity of the bloom-forming cyanobacterium, Aphanizomenon gracile, at low spatial scale
Source: ISME Commun. 2023 Jun 7;3:57. doi: 10.1038/s43705-023-00263-3 (PMC10244403; doi:10.1038/s43705-023-00263-3)
Supplement: Supplementary file 3 — Table S1 [file 43705_2023_263_MOESM3_ESM.pdf]

COG-ID	EggNog Seed Ortholog	E-value	Score	Gene name	COG category
OG001	46234.ANA_C20002	3.93e-176	492.0	dtd3	L
OG002	46234.ANA_C10916	1.15e-134	384.0	plsY	I
OG003	551115.Aazo_2954	1.17e-152	430.0	clpP	O
OG004	46234.ANA_C10404	5.81e-75	226.0	petE	C
OG005	46234.ANA_C12175	5.45e-94	274.0	sll0832	L
OG006	46234.ANA_C13573	1.98e-105	306.0	frr	J
OG007	46234.ANA_C10331	4.85e-139	394.0	plsC	I
OG008	46234.ANA_C20443	2.52e-52	165.0	-	S
OG009	46234.ANA_C10614	1.97e-124	354.0	petC	C
OG010	46234.ANA_C11579	2.69e-117	336.0	pyrR	F
OG011	46234.ANA_C11673	5.46e-62	190.0	minE	D
OG012	46234.ANA_C12467	2.09e-55	173.0	ycf19	S
OG013	46234.ANA_C12453	5.26e-44	143.0	sipA	S
OG014	46234.ANA_C20036	2.37e-175	489.0	rps2	J
OG015	46234.ANA_C20048	1.14e-148	419.0	hisI	E
OG016	46234.ANA_C12592	1.55e-79	238.0	-	S
OG017	46234.ANA_C11179	5.46e-93	273.0	rpl9	J
OG018	46234.ANA_C20574	0.0	914.0	murE	M
OG019	46234.ANA_C13686	1.48e-221	612.0	gshB	H
OG020	46234.ANA_C10078	5.41e-151	427.0	-	C
OG021	56107.Cylst_3698	3.58e-68	208.0	-	S
OG022	46234.ANA_C11594	7.87e-246	677.0	leuB	CE
OG023	46234.ANA_C11357	1.66e-125	358.0	txlA	CO
