# Supplementary material for: Intra-population genomic diversity of the bloom-forming cyanobacterium, Aphanizomenon gracile, at low spatial scale
Source: ISME Commun. 2023 Jun 7;3:57. doi: 10.1038/s43705-023-00263-3 (PMC10244403; doi:10.1038/s43705-023-00263-3)
Supplement: Supplementary file 4 — Table S2 [file 43705_2023_263_MOESM4_ESM.pdf]

BGC	Best hit from Antismash between 'Known Cluster Blast' and 'MiBiG comparison' - Score (%)				Putative product	Corresponding Annotated Analyte (Metaboscape/GNPS)	Analyte detection				Label in Figure 1.d
	PMC627.10	PMC638.10	PMC644.10	PMC649.10			PMC627.10	PMC638.10	PMC644.10	PMC649.10	
NRPS_1	xenotetrapeptide - 65%		shinorine - 69%	curacomycin - 64%	?	-					**NRPS -**
NRPS_2	anabaenopeptin - 100%	anabaenopeptin - 100%	anabaenopeptin - 100%		Anabaenopeptin	-					**Anabaenopeptin-like**
NRPS_3		anabaenopeptin - 100%			Anabaenopeptin	-					**Anabaenopeptin-like**
NRPS_4		shinorine/mycosporine glycine - 70%			shinorine/mycoscporine	Mycoscporine-lysine		X			**Shinorine/mycosporine**
NRPS_5		hexose-shinorine - 64%			Hexose-shinorine	-					**Shinorine/mycosporine**
NRPS_6		minutissamide/puwainaphycin – 100%	minutissamide/puwainaphycin – 100%	minutissamide/puwainaphycin – 100%	puwainaphycin	Minutissamide/Puwainaphycin		X	X	X	**Puwainaphycin**
NRPS_7	lincomycin - 58%	lincomycin - 57%	lincomycin - 58%	lincomycin - 58%	lincomycin	-					**NRPS -**
NRPS/PKS	Saxitoxin – 58%	Saxitoxin – 58%			saxitoxin	Saxitoxin	X	X			**Saxitoxin**
PKS_1				heterocyst glycolipids – 57%							**Heterocyst glycolipids**
PKS_2	heterocyst glycolipids – 85%	heterocyst glycolipids – 85%	heterocyst glycolipids – 85%	heterocyst glycolipids – 85%	heterocyst glycolipids	Heterocyst glycolipids	X	X	X	X	**Heterocyst glycolipids**
PKS_3	heterocyst glycolipids – 57%	heterocyst glycolipids – 57%	heterocyst glycolipids – 57%								**Heterocyst glycolipids**
PKS_4	1-heptadecene - 52%			1-heptadecene/1-nonadecene - 52%	heptadecene	-					**PKS -**
RiPPs_1	heme D1 - 55%	heme 1 - 55%	heme D1 - 55%	heme D1 - 55%	heme D1	-					**RiPP -**
RiPPs_2	Prenylgaramide B/C - 49%	prenylgaramide B/C - 49%	prenylgaramide B/C - 49%	prenylgaramide B/C - 49%	prenylgaramide	-					**RiPP -**
RiPPs_4	anacyclamide A10 – 85%	anacyclamide A10 – 81%	anacyclamide A10 – 92%	anacyclamide A10 – 92%	anacyclamide	-					**Anacyclamide-like**
RiPPs_5	curacozole - 48%	curacozole - 48%	curacozole - 58%	curacozole - 58%	curacozole	-					**RiPP -**
RiPPs_3	carotenoid - 49%										**Carotenoid**
Terpene_1		carotenoid - 65%			carotenoid	Carotenoid	X	X	X	X	**Carotenoid**
Terpene_2	carotenoid - 65%		carotenoid - 65%	carotenoid - 65%							**Carotenoid**
Terpene_3	hopene - 41%	hopene - 41%	hopene - 41%	hopene - 41%	hopene	-					**Terpene -**
Terpene_4	bryostatin - 37%	fosfomycin - 36%	capsular polysaccharide - 43%	capsular poysaccharide - 43%	Capsular polysaccharide	Capsular polysaccharide	X	X	X	X	**Capsular polysaccharide**
Others_5	?	?			?	-					**Other -**

X:see Metaboscape_Analysis Page
X:see Figure S2-GNPS network
