# Supplementary material for: Intra-population genomic diversity of the bloom-forming cyanobacterium, Aphanizomenon gracile, at low spatial scale
Source: ISME Commun. 2023 Jun 7;3:57. doi: 10.1038/s43705-023-00263-3 (PMC10244403; doi:10.1038/s43705-023-00263-3)
Supplement: Supplementary file 5 — Table S3 [file 43705_2023_263_MOESM5_ESM.pdf]

FEATURE_ID	Retention Time	PEPMASS	SIGMA SCORE	NAME_METABOSCAPE	MOLECULAR_FORMULA	ADDUCT	CAS	Mean Count			
								PMC627.10	PMC638.10	PMC644.10	PMC649.10
752	188.59	316.16325	15.249	Mycosporine-lysine	C14H24N2O6	ION=[M+H]+		0.00	2520.67	0.00	0.00
1674	633.77	1236.739	18.402	Minutissamide K	C58H100N12O17	ION=[M+H]+		0.00	6696.17	26697.67	74906.33
1682	636.18	1198.63129	8.713	Minutissamide J	C54H91ClN12O16	ION=[M+H+H]2+		0.00	10948.33	7320.50	0.00
1697	639.13	1234.72424	26.304	Minutissamide I	C58H98N12O17	ION=[M+H]+		0.00	4670.67	9118.23	139117.33
3064	586.1	1250.74376	12.549	Minutissamide G	C59H102N12O17	ION=[M+H+H]2+		0.00	3674.33	2878.67	1463.67
3114	635.73	1236.72942	5.72	Minutissamide K	C58H100N12O17	ION=[M+H]+		0.00	2267.33	1750.00	43714.00
3124	643.9	1212.64743	25.538	Minutissamide F	C55H93ClN12O16	ION=[M+H+H]2+		0.00	6342.67	4235.83	0.00
3144	661.76	1212.64898	13.738	Minutissamide F	C55H93ClN12O16	ION=[M+H+H]2+		0.00	7076.67	11883.33	0.00
3167	683.44	1212.64982	30.931	Minutissamide F	C55H93ClN12O16	ION=[M+H+H]2+		0.00	23615.33	12552.17	0.00
3153	669.01	1226.66066	16.475	Puwainaphycin C	C56H95ClN12O16	ION=[M+H+H]2+		0.00	3728.50	5897.17	0.00
3180	693.04	1226.66199	10.569	Puwainaphycin C	C56H95ClN12O16	ION=[M+H+H]2+		0.00	64847.50	280568.50	0.00
3181	692.87	1226.67435	6.993	Puwainaphycin C	C56H95ClN12O16	ION=[M+H]+		0.00	5079.17	40494.33	35.33
1904	715.99	1224.68254	14.068	Puwainaphycin D	C57H97ClN12O15	ION=[M+H+H]2+		0.00	37995.33	149509.00	0.00
1911	721.28	1192.70283	10.615	Puwainaphycin E	C56H96N12O16	ION=[M+H+H]2+		0.00	3794.17	45124.17	340.33
26	42.94	299.1338	5.856	Saxitoxin	C10H17N7O4	ION=[M+H]+		5120.31	15190.00	0.00	0.00
2228	40	315.12673	7.651	11?-hydroxysaxitoxin	C10H17N7O5	ION=[M+H+H]2+		9512.46	30772.83	0.00	0.00
2231	40.95	315.12937	2.638	11?-hydroxysaxitoxin	C10H17N7O5	ION=[M+H]+		12956.15	44931.67	0.00	0.00
1	30.83	371.90073				ION=[M+H+H]2+		5797.23	5354.33	6766.83	2026.00
2	32.96	198.98241				ION=[M+H]+		7166.15	6505.17	6254.00	5625.00
3	34.02	226.97712				ION=[M+H]+		4950.77	4670.00	4395.67	3808.33
4	34.19	315.90779				ION=[M+H+H]2+		0.00	5501.17	5360.00	0.00
5	35.69	223.12228	8.552	Tetrahydroindol 3	C12H17NO3	ION=[M+H]+		4207.08	4312.17	4249.83	1624.00
6	36.88	164.57135				ION=[M+H]+		169.69	624.33	0.00	0.00
7	37.2	501.3573				ION=[M+H+H2]3+		11.54	18.17	425.67	0.00
8	38.27	159.17416	4.535	Dimethyldipropylenetriamine	C8H21N3	ION=[M+H]+		3182.62	8292.33	4787.33	6500.33
9	39.62	430.47514				ION=[M+H+H]2+		0.00	2361.33	0.00	266.00
10	40.39	215.23747				ION=[M+H]+		0.00	1405.67	0.00	0.00
11	40.8	157.56341				ION=[M+H]+		1032.62	968.83	0.00	0.00
12	41.28	215.20122				ION=[M+H]+		440.31	169.83	30581.00	370.00
13	41.49	270.24191				ION=[M+H+H]2+		117.23	0.00	7003.50	13.00
14	41.49	151.5982				ION=[M+H]+		131.69	0.00	2713.17	63.00
15	41.52	134.62192				ION=[M+H]+		0.00	0.00	3206.67	0.00
16	41.52	317.21206				ION=[M+H+H]2+		5066.77	0.00	449.33	22.67
17	41.76	249.15158	11.994	Benzoctamine	C18H19N	ION=[M+H]+		50.92	0.00	3972.83	0.00
18	41.88	241.25266				ION=[M+H]+		0.00	0.00	0.00	81234.00
19	41.89	156.16188				ION=[M+H]+		0.00	26.83	3358.67	269.33
20	41.91	275.62637				ION=[M+H]+		50.31	1267.17	341.17	0.00
21	42.06	201.22168	7.9	Ethyl(3-{[3-(propylamino)propyl]amino}propyl)amine	C11H27N3	ION=[M+H]+		6789.85	5624.33	6151.50	11053.00
22	42.15	444.30436				ION=[M+H+H]2+		1492.92	738.50	998.83	509.00
23	42.22	187.20603				ION=[M+H]+		16147.85	1309.17	90940.67	36969.67
24	42.42	208.19537				ION=[M+H]+		0.00	0.00	0.00	4523.33
25	42.86	356.1558				ION=[M+H+H]2+		0.00	5167.33	0.00	0.00
27	42.99	445.96545				ION=[M+H+H]2+		1323.23	0.00	0.00	0.00
28	43	295.29797				ION=[M+H]+		0.00	0.00	0.00	132189.33
29	43.06	149.56571				ION=[M+H]+		2053.85	5566.33	0.00	0.00
30	43.1	338.14007				ION=[M+H+H]2+		908.15	2516.17	0.00	0.00
31	43.33	178.07828				ION=[M+H]+		2639.08	3590.00	10.33	0.00
32	43.33	415.69423				ION=[M+H+H]2+		0.00	1048.50	0.00	0.00
33	43.51	312.32422				ION=[M+H+H]2+		0.00	93.00	3031.00	1603.33
34	43.7	551.25244				ION=[M+H+H]2+		4.77	1062.50	0.00	0.00
35	43.73	243.12965				ION=[M+H+H]2+		110.15	5941.00	651.00	35.67
36	43.75	831.51902				ION=[M+H+H2]3+		0.00	1833.33	0.00	0.00
37	43.84	329.24909				ION=[M+H+H]2+		419.38	0.00	0.00	0.00
38	43.97	207.84722				ION=[M+H]+		16.77	2620.17	288.83	0.00
39	44	280.13095				ION=[M+H]+		375.69	12287.00	1964.83	22.00
40	44.07	208.09812	NA	Neocuproine	C14H12N2	ION=[M+H]+		40.92	1794.33	154.50	0.00
41	44.07	171.41895				ION=[M+H]+		25.54	1176.50	281.50	0.00
42	44.07	283.13748				ION=[M+H]+		1563.38	3608.50	12.33	55.00
43	44.07	853.35382				ION=[M+H+H2]3+		32.62	3114.67	248.33	0.00
44	44.11	467.911				ION=[M+H+H]2+		1304.31	1315.33	1361.17	0.00
45	44.25	164.62384				ION=[M+H]+		2581.69	0.00	0.00	11.67
46	44.25	241.99402				ION=[M+H]+		2502.77	4038.83	5447.17	0.00
47	44.33	435.95461				ION=[M+H+H]2+		0.00	0.00	419.00	0.00
48	44.35	318.24556				ION=[M+H]+		108.46	0.00	11399.50	0.00
49	44.4	582.23566				ION=[M+H+H]2+		0.00	2397.50	391.50	0.00
50	44.72	155.6458				ION=[M+H]+		0.00	0.00	3833.83	0.00
51	44.74	186.17414				ION=[M+H]+		4398.15	18.50	181.33	0.00
52	44.82	227.23694				ION=[M+H]+		0.00	0.00	6.67	6037.00
53	45.58	186.75424				ION=[M+H]+		0.00	632.00	107.50	0.00
54	46.05	486.26247				ION=[M+H+H]2+		50.46	1198.67	344.67	0.00
55	46.16	314.30486				ION=[M+H]+		4604.31	628.50	22557.67	29.67

56	46.17	1031.52319				ION=[M+H]+		186.31	1607.33	0.00	0.00
57	47.44	286.27356				ION=[M+H]+		20.77	9.00	5030.67	0.00
58	47.44	174.11111	5.273	Arginine	C6H14N4O2	ION=[M+H]+		4936.77	4893.33	5235.33	1102.33
59	48.25	847.77458				ION=[M+H+H]2+		0.00	0.00	464.83	0.00
60	48.53	219.92811				ION=[M+H]+		2218.77	3075.33	3112.33	316.33
61	48.93	1621.27569				ION=[M+H+H]2+		0.00	510.83	0.00	0.00
62	49.05	770.64688				ION=[M+H]+		1868.92	2301.00	708.83	1401.33
63	49.09	198.18614	13.173	Guanethidine	C10H22N4	ION=[M+H]+		39.08	16.17	0.00	14392.33
64	49.19	1639.23725				ION=[M+H+H]2+		797.08	2042.33	0.00	325.33
65	49.27	915.61605				ION=[M+H]+		2298.46	1738.33	2188.50	5315.67
66	49.45	121.91658				ION=[M+H]+		54222.92	62759.83	51835.17	91930.33
67	49.49	812.62886				ION=[M+H]+		1950.15	2762.83	427.17	1841.33
68	49.57	831.6532				ION=[M+H]+		2857.54	2281.67	2842.50	6597.33
69	49.63	695.68568				ION=[M+H]+		417.69	66.33	730.50	4889.33
70	49.66	733.80601				ION=[M+H]+		560.15	1480.83	1173.17	0.00
71	50.08	1031.53436				ION=[M+H]+		1276.46	2647.17	0.00	0.00
72	50.08	896.59291				ION=[M+H]+		1440.00	2273.00	351.33	699.00
73	50.14	854.60934				ION=[M+H]+		1282.92	2502.00	479.33	1075.67
74	50.36	997.56664				ION=[M+H]+		232.15	2199.67	85.17	332.33
75	50.4	531.41419				ION=[M+H+H]2+		7143.54	0.00	535.83	0.00
76	50.7	334.26663				ION=[M+H]+		45.38	8.67	11990.17	0.00
77	50.71	820.61591				ION=[M+H]+		1096.92	2691.17	179.00	212.67
78	50.75	863.61044				ION=[M+H]+		2745.69	44.83	6.00	0.00
79	50.82	375.80079				ION=[M+H]+		2012.15	3272.17	941.00	2288.67
80	50.94	1013.55181				ION=[M+H]+		1865.54	3164.17	594.33	1871.00
81	50.99	345.27637				ION=[M+H+H]2+		4298.15	15.17	0.00	18.00
82	51.08	1539.29975				ION=[M+H+H]2+		1571.69	2183.00	368.50	1250.00
83	51.08	947.56643				ION=[M+H]+		864.77	3963.17	0.00	0.00
84	51.4	1541.29237				ION=[M+H+H]2+		0.00	448.83	0.00	278.67
85	51.61	711.65348				ION=[M+H]+		104.92	4192.00	0.00	0.00
86	51.95	268.1535				ION=[M+H]+		1576.46	430.83	3341.83	652.33
87	52.4	321.27791				ION=[M+H]+		0.00	0.00	0.00	5507.67
88	52.64	912.31943				ION=[M+H+H]2+		839.23	363.67	2863.83	23.67
89	52.96	386.36286				ION=[M+H]+		0.00	2795.00	7.67	0.00
90	52.96	373.30881				ION=[M+H+H]2+		24.00	5088.00	23.67	0.00
91	52.96	99.10422	7.133	Cyclohexylamine	C6H13N	ION=[M+H]+		40.15	4522.00	60.50	60.33
92	53.22	202.08346				ION=[M+H]+		0.00	934.83	0.00	0.00
93	53.33	412.30216				ION=[M+H+H]2+		2755.69	0.00	70.83	0.00
94	53.35	731.17609				ION=[M+H+H]2+		2368.62	815.50	815.50	0.00
95	53.46	417.23152				ION=[M+H+H]2+		2558.00	79.83	499.67	164.67
96	53.96	693.23552				ION=[M+H]+		12223.69	3747.50	6330.67	944.33
97	54.03	464.14456				ION=[M+H]+		0.00	445.33	48.33	0.00
98	54.1	554.26959				ION=[M+H]+		3841.69	526.83	640.50	220.00
99	54.17	404.16697				ION=[M+H+H]2+		54.15	6826.50	1590.17	266.00
100	54.19	446.13071				ION=[M+H+H]2+		4081.69	3468.50	3907.50	414.00
101	54.2	431.20273	7.352	1-(2-{[3-(benzylcarbamoyl)oxiran-2-yl]formamido}-3-methylpentanoyl)pyrrolidine-2-carboxylic acid	C22H29N3O6	ION=[M+H+H]2+		0.00	2593.67	930.00	0.00
102	54.57	380.19401				ION=[M+H+H]2+		42.31	0.00	4443.67	788.33
103	54.6	233.10396	NA	5,8-Dimethoxy-1,4-Dimethylquinolin-2(1h)-One	C13H15NO3	ION=[M+H]+	131451-78-0	2987.69	988.17	1064.17	1868.33
104	54.61	890.34297				ION=[M+H+H]2+		30537.23	32081.50	59981.50	18020.00
105	54.64	334.25796				ION=[M+H]+		72.77	0.00	2336.17	55.00
106	54.64	436.66757				ION=[M+H]+		950.62	858.17	1427.00	804.00
107	54.72	127.06273	NA	N-Ethylsuccinimide	C6H9NO2	ION=[M+H]+	2314-78-5	0.00	0.00	462.50	0.00
108	54.83	321.2416				ION=[M+H]+		9.85	2680.67	112.50	0.00
109	54.85	344.16451				ION=[M+H+H]2+		5156.62	3996.00	7156.67	3221.67
110	54.86	319.13838	11.439	TAN-868 A	C13H17N7O3	ION=[M+H]+		9115.23	8021.33	9557.50	4158.67
111	54.86	215.60256				ION=[M+H]+		4102.77	1632.17	5106.17	1664.00
112	54.99	254.12402				ION=[M+H+H]2+		2658.15	2247.33	2686.17	2068.67
113	55.11	445.9334				ION=[M+H+H]2+		495.38	0.00	0.00	0.00
114	55.29	289.13648				ION=[M+H]+		3153.69	9031.83	3046.00	1661.67
115	55.47	229.17212	NA	Icaridin	C12H23NO3	ION=[M+H]+	119515-38-7	2188.77	0.00	0.00	0.00
116	55.58	472.32813				ION=[M+H+H]2+		2117.85	3.67	0.00	0.00
117	55.85	483.10675				ION=[M+H+H]2+		1302.15	893.83	2470.33	773.67
118	55.88	261.09406	NA	N-(5-isopropyl-thiazol-2-yl)-2-pyridin-3-yl-acetamide	C13H15N3OS	ION=[M+H]+		2610.46	567.83	237.17	1586.67
119	55.9	259.18929	20.333	Phormidinine A	C17H25NO	ION=[M+H]+		4926.77	1268.17	1342.17	3214.33
120	56.13	230.60542				ION=[M+H]+		8349.38	0.00	0.00	3094.00
121	56.24	199.09397				ION=[M+H]+		516.62	587.00	757.83	23.33
122	56.28	242.16385	NA	Anaephene B	C17H22O	ION=[M+H]+		2894.92	0.00	22.17	0.00
123	56.48	631.33294				ION=[M+H+H]2+		102.92	22.67	23.00	6221.67
124	56.6	146.06901	NA	D-(-)-Glutamine	C5H10N2O3	ION=[M+H]+		2752.46	1321.83	2811.33	1761.33
125	56.63	277.7886				ION=[M+H]+		1117.69	208.00	0.00	326.33
126	56.73	638.27783				ION=[M+H+H]2+		0.00	0.00	2134.83	1329.67

127	57	190.11816				ION=[M+H]+		322.00	1227.67	3273.33	6447.67
128	57.08	287.19499				ION=[M+H]+		7246.31	1430.33	2346.50	5463.67
129	57.56	872.33618				ION=[M+H+H]2+		395.54	1211.50	2505.00	0.00
130	57.63	532.24983				ION=[M+H+H]2+		2164.31	353.83	3414.50	3190.00
131	57.79	436.17603				ION=[M+H+H]2+		8413.69	17225.83	19367.00	42243.67
132	57.83	243.19477				ION=[M+H]+		9602.92	30.17	0.00	5484.00
133	57.84	957.41714				ION=[M+H+H2]3+		0.00	43.83	0.00	1383.33
134	57.85	306.16162				ION=[M+H]+		64602.62	2187.33	0.00	140.67
135	57.9	195.08734	NA	N-Acetyldopamine	C10H13NO3	ION=[M+H]+		623.23	131.83	494.50	0.00
136	57.99	267.09512	9.327	Vidarabine | Adenosin	C10H13N5O4	ION=[M+H]+		2477.23	3017.17	3825.50	2370.33
137	58.02	376.23469				ION=[M+H+H]2+		294.77	526.50	0.00	0.00
138	58.03	241.55389				ION=[M+H]+		56.46	76.67	589.33	108.33
139	58.05	281.10904				ION=[M+H]+		69.85	1959.33	370.17	77.33
140	58.13	492.12554				ION=[M+H+H]2+		3276.00	4680.17	7930.00	2353.00
141	58.19	601.23413				ION=[M+H]+		6002.77	3970.67	17650.50	15671.00
142	58.28	432.22351				ION=[M+H+H]2+		387.23	0.00	0.00	0.00
143	58.3	171.64307				ION=[M+H]+		1705.54	0.00	0.00	0.00
144	58.36	426.22801				ION=[M+H+H]2+		813.08	0.00	0.00	0.00
145	58.43	700.3931				ION=[M+H+H2]3+		11.38	0.00	26.67	5019.67
146	58.54	946.30048				ION=[M+H+H]2+		1009.69	1060.33	2979.00	771.00
147	58.65	293.04248				ION=[M+H]+		103.69	42.50	108.67	2691.33
148	58.74	266.12989	NA	(E,E)-Dienestrol	C18H18O2	ION=[M+H]+		110.77	81.17	1011.67	319.33
149	58.79	227.049				ION=[M+H]+		1184.15	1227.83	0.00	0.00
150	58.86	175.09665				ION=[M+H]+		2196.46	3712.83	2354.17	373.00
151	58.89	247.56384				ION=[M+H]+		0.00	927.50	0.00	0.00
152	59.04	514.92527				ION=[M+H+H2]3+		1136.46	0.00	0.00	0.00
153	59.08	202.09635				ION=[M+H]+		4563.23	487.17	2049.83	2085.67
154	59.15	267.05511	NA	Oxycarboxin	C12H13NO4S	ION=[M+H]+		384.92	0.00	2219.83	0.00
155	59.22	563.20763				ION=[M+H]+		5456.15	6410.33	3233.67	5377.67
156	59.23	495.12611				ION=[M+H+H]2+		1304.92	1456.00	330.00	72.33
157	59.38	456.16243				ION=[M+H]+		12099.54	875.00	290.17	402.00
158	59.53	454.09729				ION=[M+H+H]2+		2660.31	4245.33	1060.00	874.67
159	59.59	215.05854	NA	1-Nitro-2-phenoxybenzene	C12H9NO3	ION=[M+H]+		3514.62	956.00	1033.67	507.00
160	59.6	549.18886				ION=[M+H]+		942.92	1822.17	453.50	571.67
161	59.7	489.16938				ION=[M+H]+		4743.23	2606.50	6691.00	11404.00
162	59.71	232.1077	NA	N~2~-(3-Carboxy-1-hydroxypropylidene)ornithine	C9H16N2O5	ION=[M+H]+	80102-04-1	5744.31	926.33	1837.33	2546.33
163	59.75	672.18589				ION=[M+H+H]2+		1338.31	1387.67	2002.00	900.00
164	59.81	634.2451				ION=[M+H]+		1221.23	1075.50	273.83	479.33
165	59.92	796.30132				ION=[M+H]+		4100.00	1302.33	3364.67	10264.33
166	60.01	244.57579				ION=[M+H]+		21.54	23.50	2884.67	0.00
167	60.04	293.05325				ION=[M+H]+		2889.08	1214.17	193.33	5697.33
168	60.14	346.27263	16.906	N-[2,6-Bis(diethylamino)pyridin-3-yl]cyclohexanecarboxamide	C20H34N4O	ION=[M+H+H]2+		487.69	2696.67	0.00	982.00
169	60.18	416.08369				ION=[M+H+H]2+		998.31	0.00	0.00	0.00
170	60.2	370.33525				ION=[M+H+H]2+		1683.85	0.00	97.17	0.00
171	60.21	165.0465	NA	Dl-methionine sulfoxide	C5H11NO3S	ION=[M+H]+		1027.08	0.00	0.00	0.00
172	60.21	161.06951	NA	Fenamole	C7H7N5	ION=[M+H]+	5467-78-7	2436.92	1945.00	3933.83	2344.00
173	60.23	133.03764				ION=[M+H]+		1664.46	1973.50	3602.50	1681.33
174	60.24	472.10807				ION=[M+H+H]2+		5089.08	6930.67	974.50	1714.67
175	60.28	870.33764				ION=[M+H]+		2031.23	2944.00	969.00	1808.00
176	60.3	219.54949				ION=[M+H]+		1845.85	2990.33	0.00	0.00
177	60.4	304.13771				ION=[M+H]+		662.00	230.17	2919.67	557.00
178	60.4	216.11024				ION=[M+H]+		8035.54	1253.50	2293.33	4003.00
179	60.44	439.09855				ION=[M+H+H]2+		5207.85	542.67	2371.67	2559.33
180	60.45	401.15363				ION=[M+H]+		10978.15	19092.17	8499.67	13676.67
181	60.58	135.05409				ION=[M+H]+		108.15	1797.67	237.83	53.67
182	60.6	245.1377	NA	Tasimelteon	C15H19NO2	ION=[M+H]+	609799-22-6	656.31	143.33	0.00	408.33
183	60.6	185.10923				ION=[M+H]+		1380.62	1545.17	0.00	687.67
184	60.63	708.28323				ION=[M+H]+		7285.69	11096.83	3583.50	6226.00
185	60.73	488.15036				ION=[M+H+H]2+		0.00	0.00	7530.17	110.67
186	60.73	708.19515				ION=[M+H+H]2+		2509.85	5629.67	103.00	542.00
187	60.77	544.24951				ION=[M+H]+		19.54	0.00	2012.00	0.00
188	60.89	462.11592				ION=[M+H+H]2+		2227.23	1447.00	195.17	975.33
189	60.89	218.58589				ION=[M+H]+		3909.69	3162.17	6470.50	3461.33
190	60.94	343.14825	18.81	Amphistin	C13H21N5O6	ION=[M+H]+		1806.31	2391.17	530.17	1245.33
191	60.94	746.22125				ION=[M+H+H]2+		2208.46	3434.83	1506.33	797.33
192	60.97	174.102				ION=[M+H]+		1464.46	436.67	416.83	0.00
193	60.98	554.24469				ION=[M+H]+		1805.38	26.83	415.83	332.67
194	61.14	560.12446				ION=[M+H+H]2+		2378.46	1343.17	706.50	1355.33
195	61.3	722.1733				ION=[M+H+H]2+		2123.69	694.50	628.50	1175.33
196	61.44	834.2474				ION=[M+H+H]2+		1619.54	259.33	1049.83	806.67
197	61.49	495.20667				ION=[M+H+H]2+		2082.15	1402.83	2684.50	2255.67
198	61.5	458.18164				ION=[M+H+H]2+		644.15	0.00	0.00	0.00
199	61.5	796.21247				ION=[M+H+H]2+		1150.62	1921.33	239.00	495.33

200	61.52	245.13222				ION=[M+H]+		8275.85	2109.17	814.67	4984.33
201	61.58	634.16174				ION=[M+H+H]2+		2466.00	4254.50	806.17	3112.00
202	61.66	387.13786				ION=[M+H]+		2818.77	6660.67	3380.17	2489.67
203	61.66	962.29124				ION=[M+H+H]2+		564.92	2083.83	50.00	98.67
204	61.66	800.24191				ION=[M+H+H]2+		521.69	3358.50	113.50	40.33
205	61.7	188.11705	7.821	Cyclohexyl phenyl ketone	C13H16O	ION=[M+H]+		7812.15	1583.67	3298.33	4166.00
206	61.72	733.2799				ION=[M+H]+		868.31	434.67	4009.00	4824.33
207	61.73	338.05319	18.721	Tmp	C10H15N2O9P	ION=[M+H]+	3590-47-4	2194.62	4110.17	984.17	860.00
208	61.79	426.08611				ION=[M+H+H]2+		1965.08	933.17	3223.83	1271.67
209	61.82	166.53645				ION=[M+H]+		1436.92	3021.17	775.33	573.00
210	61.82	398.10638				ION=[M+H]+		508.15	0.00	0.00	0.00
211	61.9	561.11396				ION=[M+H]+		380.92	1529.50	0.00	264.00
212	61.95	878.26371				ION=[M+H+H]2+		1562.00	2484.83	1308.33	1399.00
213	62	546.14326				ION=[M+H+H]2+		3623.85	11607.33	627.83	1009.67
214	62.11	310.05535	5.733	{[3-hydroxy-5-(4-hydroxy-2-oxo-1,2,3,4-tetrahydropyrimidin-1-yl)oxolan-2-yl]methoxy}phosphonic acid	C9H15N2O8P	ION=[M+H+H]2+		4925.69	9842.67	2084.33	1791.00
215	62.2	231.1478				ION=[M+H]+		0.00	4403.17	0.00	0.00
216	62.24	291.11892	NA	Danegaptide	C14H17N3O4	ION=[M+H]+	943134-39-2	1833.69	6544.50	3159.00	1120.00
217	62.31	381.16354	18.241	Zeatin-7-N-glucoside	C16H23N5O6	ION=[M+H]+		75.08	14154.67	352.67	1706.33
218	62.46	550.21976				ION=[M+H+H]2+		737.08	322.50	0.00	0.00
219	62.48	675.27317				ION=[M+H]+		2073.38	794.33	3627.50	2345.67
220	62.58	699.26593				ION=[M+H]+		1392.31	547.83	5135.50	1498.33
221	62.67	648.21034				ION=[M+H+H]2+		261.23	442.50	426.50	0.00
222	62.71	247.60299				ION=[M+H]+		2179.23	1300.17	2311.67	0.00
223	63.02	231.11846				ION=[M+H]+		4556.62	0.00	221.00	0.00
224	63.03	202.1329	NA	Glutamine T-Butyl Ester	C9H18N2O3	ION=[M+H]+	39741-62-3	3252.15	360.00	2025.67	6754.33
225	63.14	273.11446	NA	[(2-Ethoxy-1-Naphthoyl)amino]methylboronic Acid	C14H16BNO4	ION=[M+H]+		0.00	1606.83	0.00	0.00
226	63.14	256.56701				ION=[M+H]+		1686.15	1646.83	710.33	759.33
227	63.3	291.10971	NA	Obscurolide A1	C15H17NO5	ION=[M+H]+		1511.23	0.00	2146.17	1081.33
228	63.54	192.04724	NA	4-Methyl-3-(2-pyridyl)-4,5-dihydro-1H-1,2,4-triazole-5-thione	C8H8N4S	ION=[M+H]+		1279.69	1050.33	605.00	202.00
229	63.62	384.09404				ION=[M+H+H]2+		484.31	1128.83	0.00	0.00
230	63.63	353.01787				ION=[M+H]+		1325.23	2451.00	622.00	772.00
231	63.8	486.15385				ION=[M+H+H]2+		641.38	303.67	180.00	0.00
232	63.93	624.16542				ION=[M+H+H]2+		952.15	1762.17	5357.17	1517.67
233	64.42	530.18291				ION=[M+H]+		2603.54	9200.50	249.83	1646.33
234	64.86	346.14551				ION=[M+H+H]2+		1741.38	791.33	202.83	2205.33
235	64.99	173.07816	19.545	4.8-dimethyl-6-hydroxyquinoline	C11H11NO	ION=[M+H]+		1806.46	12931.67	7367.83	0.00
236	65.2	642.17026				ION=[M+H+H]2+		1237.54	923.67	3026.67	873.00
237	65.21	424.16975				ION=[M+H]+		2219.08	1554.83	5488.67	2384.67
238	65.79	408.22126				ION=[M+H+H]2+		1329.38	251.00	503.83	550.33
239	71.03	379.96391				ION=[M+H+H]2+		2105.85	1024.17	1673.50	2753.67
240	72.39	343.94017				ION=[M+H+H]2+		0.00	2325.33	0.00	0.00
241	72.45	595.1601				ION=[M+H+H]2+		66.62	21.50	2540.17	34.67
242	72.94	648.23081				ION=[M+H+H2]3+		826.00	632.67	449.50	1206.67
243	73.64	212.99778				ION=[M+H]+		4376.77	4089.17	3721.83	3547.00
244	77	582.18527				ION=[M+H+H]2+		4992.31	147.33	4759.67	3159.67
245	77.08	527.83343				ION=[M+H]+		3353.54	3629.33	2479.83	5757.33
246	83.22	390.33523				ION=[M+H+H]2+		0.00	4084.00	8.83	0.00
247	83.35	212.04458				ION=[M+H]+		381.08	0.00	4717.83	1036.67
248	83.42	458.13988				ION=[M+H]+		9.85	4.33	2480.50	179.67
249	85.29	115.98662				ION=[M+H]+		2689.08	2471.33	1843.67	3690.33
250	85.35	194.96134				ION=[M+H]+		2114.15	1974.50	1597.83	560.00
251	86.18	140.95079				ION=[M+H]+		1632.46	2325.50	1933.17	47.67
252	86.37	640.77552				ION=[M+H]+		8146.46	6977.50	6535.33	12583.67
253	86.58	489.10021				ION=[M+H]+		286.15	0.00	5965.00	1032.33
254	87.22	475.84215				ION=[M+H+H]2+		412.62	253.83	1053.17	0.00
255	88.4	358.00731				ION=[M+H]+		1565.54	820.00	3074.17	5664.33
256	89.47	542.79644				ION=[M+H]+		3994.62	3417.50	3322.17	6120.33
257	90.87	576.26467				ION=[M+H+H]2+		0.00	145.67	6647.33	0.00
258	91.21	389.08931				ION=[M+H]+		1541.85	390.67	6815.83	2566.67
259	91.71	243.19499				ION=[M+H]+		3117.85	283.17	0.00	381.00
260	91.81	301.46743				ION=[M+H]+		1126.62	566.33	1399.83	29.00
261	92.45	289.12868	14.816	Tabtoxin	C11H19N3O6	ION=[M+H]+		1952.00	4082.00	3550.67	454.33
262	92.92	741.94716				ION=[M+H+H]2+		494.92	0.00	0.00	0.00
263	93.12	416.03896				ION=[M+H]+		7871.08	1160.50	2715.17	4805.67
264	93.66	291.10813	NA	Obscurolide A1	C15H17NO5	ION=[M+H]+		50.15	1851.83	0.00	0.00
265	93.81	636.1271				ION=[M+H]+		1185.23	13.83	68.50	0.00

266	93.82	449.15647	13.379	1-({3-methyl-4-[4-(1,1,1-trifluoro-2-hydroxypropan-2-yl)benzenesulfonyl]piperazin-1-yl}methyl)cyclopropane-1-carboxamide	C19H26F3N3O4S	ION=[M+H]+		1345.69	190.50	260.50	17.33
267	93.95	370.97362				ION=[M+H]+		1401.85	591.00	907.00	23.00
268	93.96	374.01943				ION=[M+H]+		2190.46	704.83	986.83	0.00
269	94.26	172.01435				ION=[M+H]+		2547.23	991.00	3204.00	3590.00
270	94.47	271.25859				ION=[M+H+H]2+		0.00	2309.83	9.33	0.00
271	94.63	420.34541				ION=[M+H+H]2+		352.15	2503.83	0.00	0.00
272	94.66	904.40412				ION=[M+H+H2]3+		1120.62	1096.17	5043.17	2158.67
273	94.96	344.96772				ION=[M+H]+		1190.46	472.00	1937.33	0.00
274	95.1	315.66646				ION=[M+H]+		54.00	0.00	0.00	1211.67
275	95.11	658.79813				ION=[M+H+H]2+		433.08	840.83	3051.17	625.00
276	95.16	329.64922				ION=[M+H]+		317.23	526.00	2027.33	0.00
277	95.22	757.34087				ION=[M+H+H]2+		42.31	8.83	71.00	5641.67
278	95.23	393.29849				ION=[M+H+H]2+		0.00	0.00	0.00	4751.00
279	95.27	196.64972				ION=[M+H]+		0.00	19.00	0.00	4341.00
280	95.28	631.33228				ION=[M+H+H]2+		1296.92	385.17	761.50	7735.33
281	95.33	343.28267				ION=[M+H+H]2+		6689.08	433.00	0.00	0.00
282	95.34	1081.4825				ION=[M+H+H]2+		2518.77	1753.50	7611.67	5128.00
283	95.41	403.22385				ION=[M+H]+		35.23	2150.33	0.00	0.00
284	95.46	860.38404				ION=[M+H+H]2+		3414.46	2590.50	13658.00	5900.67
285	95.59	642.35594				ION=[M+H+H]2+		585.23	0.00	0.00	0.00
286	95.59	346.27199	16.696	N-[2,6-Bis(diethylamino)pyridin-3-yl]cyclohexanecarboxamide	C20H34N4O	ION=[M+H+H]2+		0.00	7732.33	8.83	0.00
287	95.59	329.39759				ION=[M+H]+		467.23	0.00	0.00	0.00
288	95.73	431.32637				ION=[M+H+H]2+		0.00	0.00	539.83	0.00
289	95.74	433.35212				ION=[M+H+H]2+		0.00	0.00	2305.67	0.00
290	95.91	638.28147				ION=[M+H+H]2+		3518.31	2823.50	13396.67	2916.33
291	95.94	608.23044				ION=[M+H]+		14.77	0.00	1685.83	0.00
292	95.95	981.94398				ION=[M+H+H]2+		368.77	330.83	1335.33	187.33
293	95.95	981.43945				ION=[M+H+H]2+		731.08	503.50	1689.33	231.00
294	95.95	429.69516				ION=[M+H]+		0.00	0.00	424.50	28.00
295	96.01	638.78197				ION=[M+H+H]2+		2424.46	1298.50	8491.17	1661.00
296	96.03	512.35081				ION=[M+H+H]2+		4106.46	0.00	0.00	26.67
297	96.12	387.30933				ION=[M+H+H]2+		0.00	2827.00	12.00	30.00
298	96.12	893.38697				ION=[M+H]+		0.00	1666.17	80.33	0.00
299	96.13	613.39606				ION=[M+H+H]2+		4866.15	1129.00	7.83	0.00
300	96.14	300.28598				ION=[M+H+H]2+		6.62	6.00	3111.50	6753.33
301	96.2	1456.57834				ION=[M+H+H]2+		3709.54	1385.50	5410.00	1552.67
302	96.23	306.69773				ION=[M+H]+		0.00	1068.50	0.00	0.00
303	96.25	684.42302				ION=[M+H+H]2+		2332.15	116.67	394.50	28.67
304	96.28	492.23619				ION=[M+H+H]2+		4440.62	792.33	1212.33	0.00
305	96.31	859.88309				ION=[M+H+H]2+		4405.54	5070.00	22459.83	7350.67
306	96.34	1347.59631				ION=[M+H+H2]3+		3931.69	4426.17	14301.83	5129.00
307	96.35	319.38998				ION=[M+H]+		0.00	443.00	127.17	0.00
308	96.35	1080.49161				ION=[M+H+H]2+		169.08	31.67	1079.33	482.33
309	96.36	1668.7301				ION=[M+H+H2]3+		279.85	784.00	2054.67	624.67
310	96.37	401.32527				ION=[M+H+H]2+		0.00	2606.50	0.00	10.33
311	96.44	744.40438				ION=[M+H+H]2+		853.23	1659.67	228.00	910.33
312	96.44	872.33468				ION=[M+H+H]2+		1842.00	2532.67	1974.17	1442.67
313	96.46	893.38018				ION=[M+H]+		0.00	1925.83	180.33	0.00
314	96.47	200.66152				ION=[M+H]+		0.00	535.33	0.00	0.00
315	96.52	598.96529				ION=[M+H]+		12.00	3180.00	0.00	0.00
316	96.6	859.39022				ION=[M+H+H]2+		2027.08	4047.17	8124.17	3114.67
317	96.63	1117.49851				ION=[M+H+H]2+		120.62	312.33	2117.00	893.00
318	96.64	1028.47585				ION=[M+H+H]2+		425.23	0.00	0.00	0.00
319	96.64	354.33463				ION=[M+H+H]2+		2480.00	0.00	0.00	14.00
320	96.66	602.22084				ION=[M+H]+		0.00	5433.50	0.00	0.00
321	96.66	140.04711	10.144	2-Methoxyresorcinol	C7H8O3	ION=[M+H]+		27.54	4176.50	0.00	0.00
322	96.66	138.06785	4.854	2-Phenoxyethanol	C8H10O2	ION=[M+H]+	122-99-6	61.38	10059.00	24.83	105.67
323	96.66	152.04762				ION=[M+H]+		21.08	2592.83	0.00	66.00
324	96.69	343.95262				ION=[M+H]+		413.69	629.17	2928.67	583.33
325	96.69	688.29209				ION=[M+H]+		145.69	9.00	4719.83	11.67
326	96.75	528.28641				ION=[M+H+H]2+		1152.46	421.67	2819.17	1082.00
327	96.79	700.39037				ION=[M+H+H2]3+		0.00	21.17	0.00	18622.67
328	96.86	413.37265				ION=[M+H+H]2+		6.62	0.00	29250.17	0.00
329	96.87	1080.9952				ION=[M+H+H]2+		1341.85	2633.50	5571.17	3207.00
330	96.9	826.33876				ION=[M+H+H]2+		26802.77	25564.00	25325.83	21167.33
331	96.9	721.32385				ION=[M+H]+		13.08	122.00	2341.83	0.00
332	96.9	457.17759	NA	Dankastatin B	C23H33Cl2NO4	ION=[M+H]+		14.92	14.67	509.17	0.00
333	96.9	720.99009				ION=[M+H]+		0.00	0.00	479.00	0.00
334	96.9	865.18134				ION=[M+H+H]2+		841.85	908.33	3849.33	1692.00
335	96.9	521.23126				ION=[M+H]+		748.92	396.17	2167.00	1968.67
336	96.9	432.99035				ION=[M+H]+		216.62	56.00	1348.00	270.33

337	96.9	1225.5418				ION=[M+H+H2]3+		143.38	58.67	2177.67	0.00
338	96.92	229.63599				ION=[M+H]+		1606.31	252.17	575.33	615.67
339	96.92	171.80704				ION=[M+H]+		616.46	0.00	0.00	0.00
340	96.92	275.14745				ION=[M+H]+		491.38	60.33	0.00	0.00
341	96.92	426.675				ION=[M+H]+		393.85	0.00	0.00	0.00
342	96.92	402.22306				ION=[M+H+H]2+		1819.08	682.50	526.00	1965.33
343	96.92	703.36904				ION=[M+H+H]2+		2336.00	335.33	500.50	958.00
344	96.92	745.39313				ION=[M+H+H]2+		2281.08	840.00	144.00	662.00
345	96.92	498.28648				ION=[M+H+H]2+		898.62	42.67	315.83	0.00
346	96.93	894.91034				ION=[M+H+H]2+		399.23	2391.17	8066.33	506.33
347	96.96	673.79961				ION=[M+H+H]2+		1719.23	1952.67	8569.33	1919.33
348	96.96	1347.61366				ION=[M+H+H]2+		339.54	501.83	3110.83	179.00
349	96.97	1719.7697				ION=[M+H+H2]3+		1746.62	1517.33	11701.17	3304.67
350	96.97	370.32976				ION=[M+H+H]2+		4982.92	0.00	182.17	0.00
351	97.02	446.80006				ION=[M+H]+		51.38	115.00	1870.33	141.33
352	97.06	1790.80773				ION=[M+H+H2]3+		371.38	987.67	5231.67	778.00
353	97.07	687.35103				ION=[M+H+H2]3+		0.00	0.00	0.00	989.33
354	97.07	193.1262				ION=[M+H]+		23.54	0.00	0.00	7557.33
355	97.07	219.12073	NA	Prumycin	C8H17N3O4	ION=[M+H]+		297.08	84.83	293.83	3693.00
356	97.08	1117.00524				ION=[M+H+H]2+		198.46	450.67	2782.83	1113.67
357	97.1	331.55257				ION=[M+H]+		1555.85	1242.67	2433.50	888.67
358	97.11	472.32291	14.551	Comnostin D	C29H44O5	ION=[M+H+H]2+		6641.38	0.00	484.67	25.67
359	97.13	2160.99128				ION=[M+H+H2]3+		0.00	356.83	891.50	0.00
360	97.13	628.34732				ION=[M+H+H]2+		498.15	981.67	2023.17	748.33
361	97.13	1392.4351				ION=[M+H+H]2+		0.00	0.00	601.83	0.00
362	97.13	520.83875				ION=[M+H]+		194.92	1417.00	2446.33	925.00
363	97.13	343.75308				ION=[M+H]+		90.77	492.67	1109.50	0.00
364	97.13	446.60433				ION=[M+H]+		9.69	363.67	1793.83	170.00
365	97.13	192.05915	NA	4-(carboxyvin-2-Yl)phenylboronic Acid	C9H9BO4	ION=[M+H]+		1177.23	1026.50	2613.50	0.00
366	97.13	1030.3376				ION=[M+H+H]2+		914.15	364.00	232.17	3583.33
367	97.13	657.3561				ION=[M+H+H2]3+		0.00	0.00	109.67	851.67
368	97.13	596.60972				ION=[M+H]+		0.00	0.00	1123.83	0.00
369	97.13	596.94233				ION=[M+H]+		22.92	203.00	1894.33	114.33
370	97.13	1073.95677				ION=[M+H+H]2+		1002.62	2342.67	3240.67	1780.00
371	97.13	720.33306				ION=[M+H]+		0.00	0.00	433.17	0.00
372	97.13	651.30072				ION=[M+H]+		562.15	612.33	1004.83	458.00
373	97.13	432.79611				ION=[M+H]+		445.69	397.17	1951.00	910.67
374	97.13	954.33944				ION=[M+H+H]2+		716.46	1775.67	2239.50	0.00
375	97.13	540.24844				ION=[M+H]+		367.38	2153.33	1934.83	812.00
376	97.13	720.6684				ION=[M+H]+		23.08	324.67	1682.33	0.00
377	97.14	2162.97959				ION=[M+H+H2]3+		136.92	316.67	1046.17	98.67
378	97.16	458.34395				ION=[M+H+H]2+		3823.38	0.00	0.00	0.00
379	97.18	674.30572				ION=[M+H+H]2+		877.54	1390.50	5249.17	279.67
380	97.18	1706.69536				ION=[M+H+H2]3+		2738.00	2017.50	6294.83	4336.67
381	97.19	852.84696				ION=[M+H+H]2+		2099.23	1443.17	4436.00	2669.33
382	97.23	520.27659				ION=[M+H+H]2+		0.00	19.83	0.00	15107.00
383	97.24	942.54231				ION=[M+H+H2]3+		1648.92	693.17	0.00	510.67
384	97.25	574.30234				ION=[M+H+H]2+		840.62	0.00	0.00	0.00
385	97.27	405.13894				ION=[M+H]+		514.46	839.50	6313.00	3098.67
386	97.28	277.56336				ION=[M+H]+		0.00	1387.00	0.00	0.00
387	97.3	428.18377				ION=[M+H]+		98.46	0.00	71.00	1406.33
388	97.32	932.35422				ION=[M+H+H]2+		22884.46	24320.50	39578.17	48007.00
389	97.34	362.1535				ION=[M+H+H]2+		9.23	437.67	0.00	0.00
390	97.36	896.51981				ION=[M+H+H2]3+		2326.15	762.50	292.67	285.33
391	97.36	1705.7021				ION=[M+H+H2]3+		3706.31	2592.17	6623.50	6057.67
392	97.37	895.90052				ION=[M+H+H]2+		925.23	1421.83	11559.50	1708.00
393	97.38	430.19247				ION=[M+H]+		448.15	843.33	3680.67	0.00
394	97.39	460.30663				ION=[M+H+H]2+		29582.62	21.83	0.00	120.00
395	97.4	1199.54874				ION=[M+H+H]2+		60.00	115.67	2696.00	85.00
396	97.41	599.77078				ION=[M+H]+		92.77	85.17	809.00	40.33
397	97.46	217.1122	NA	Glutethimide	C13H15NO2	ION=[M+H]+	77-21-4	288.31	0.00	37.33	8223.67
398	97.47	895.40339				ION=[M+H+H]2+		1001.23	2751.17	15225.17	2152.67
399	97.53	519.63042				ION=[M+H]+		1131.54	1750.50	3578.00	2888.33
400	97.53	1116.51503				ION=[M+H+H]2+		188.15	533.83	2804.33	162.00
401	97.53	853.35002				ION=[M+H+H]2+		1281.54	1023.00	3630.33	2365.33
402	97.53	608.07355				ION=[M+H]+		729.23	1148.50	2135.67	1374.33
403	97.53	1718.78674				ION=[M+H+H2]3+		699.23	1457.00	5118.00	1061.00
404	97.53	914.34738				ION=[M+H+H]2+		1700.92	1994.83	3515.83	2954.33
405	97.53	2147.92463				ION=[M+H+H2]3+		676.77	941.50	2757.50	636.67
406	97.55	808.3335				ION=[M+H+H]2+		3555.38	2830.67	5325.17	1162.33
407	97.57	294.6639				ION=[M+H]+		1306.77	216.83	286.33	1253.67
408	97.57	301.1643	NA	4-amino-6-(diethylcarbamoyl)-5-acetamidooxane-2-carboxylic acid	C13H23N3O5	ION=[M+H]+		1807.08	593.33	1063.33	474.67
409	97.57	487.29563				ION=[M+H+H]2+		1998.46	721.83	830.17	2537.67

410	97.57	550.29063				ION=[M+H+H]2+		1864.62	975.00	86.67	0.00
411	97.57	307.67478				ION=[M+H]+		1743.69	647.17	1063.33	298.00
412	97.57	570.33478				ION=[M+H+H]2+		634.15	0.00	0.00	0.00
413	97.57	428.43215				ION=[M+H]+		1007.23	0.00	0.00	0.00
414	97.57	257.71026				ION=[M+H]+		497.38	0.00	0.00	0.00
415	97.57	746.39086				ION=[M+H+H]2+		2364.77	1452.00	550.67	1511.33
416	97.57	372.6941				ION=[M+H]+		1144.00	0.00	114.67	0.00
417	97.58	632.34669				ION=[M+H+H]2+		3111.23	941.33	1395.33	3914.33
418	97.58	294.15896				ION=[M+H]+		1988.77	1165.83	603.67	507.33
419	97.59	128.09464				ION=[M+H]+		1786.46	501.00	1217.17	1629.33
420	97.59	748.36485				ION=[M+H+H]2+		666.00	61.83	0.00	438.67
421	97.59	332.16964				ION=[M+H]+		2125.38	404.17	837.00	1660.67
422	97.59	813.47176				ION=[M+H+H2]3+		562.46	0.00	296.00	1985.67
423	97.59	714.64292				ION=[M+H]+		446.77	0.00	0.00	134.67
424	97.59	342.19219	NA	Compound iv	C20H26N2O3	ION=[M+H]+	87495-31-6	2241.54	680.67	688.67	1195.33
425	97.59	303.14836	NA	Fenoterol	C17H21NO4	ION=[M+H]+		450.15	0.00	0.00	0.00
426	97.59	507.25725				ION=[M+H+H]2+		760.15	0.00	29.67	0.00
427	97.59	984.52587				ION=[M+H+H2]3+		2162.46	524.00	572.17	1563.33
428	97.65	1074.95107				ION=[M+H+H]2+		2615.23	2018.00	5410.67	4547.67
429	97.67	161.06951	NA	Fenamole	C7H7N5	ION=[M+H]+	5467-78-7	1814.92	1258.83	2670.33	2000.33
430	97.68	538.9787				ION=[M+H]+		39.38	0.00	433.00	137.67
431	97.72	1789.82377				ION=[M+H+H2]3+		62.77	813.00	2811.33	0.00
432	97.72	639.25933				ION=[M+H]+		823.08	659.67	1442.83	86.33
433	97.72	1116.0185				ION=[M+H+H]2+		116.00	616.83	1907.83	147.00
434	97.72	1302.59897				ION=[M+H+H]2+		76.92	150.17	1336.17	148.00
435	97.72	1068.47082				ION=[M+H+H]2+		1000.46	973.83	1992.83	1323.33
436	97.72	1302.0989				ION=[M+H+H]2+		120.77	747.50	2023.50	345.33
437	97.72	518.2214				ION=[M+H]+		887.38	1034.83	2204.17	992.33
438	97.72	518.42238				ION=[M+H]+		827.85	1140.67	2454.00	1094.00
439	97.78	185.16499				ION=[M+H]+		4012.92	0.00	0.00	0.00
440	97.78	185.66421				ION=[M+H]+		5186.00	0.00	15.67	0.00
441	97.8	643.23097	11.522	Sabarubicin	C32H37NO13	ION=[M+H+H]2+	211100-13-9	160.92	8745.33	9.83	0.00
442	97.8	1299.07668				ION=[M+H+H]2+		1639.85	1547.17	3185.00	2765.00
443	97.84	313.08505				ION=[M+H]+		4808.00	7806.17	16077.83	8513.33
444	97.85	400.29784				ION=[M+H+H]2+		744.31	80.83	462.67	14858.00
445	97.92	568.90207				ION=[M+H]+		168.77	361.67	583.83	830.67
446	97.92	446.99867				ION=[M+H]+		48.92	81.83	1740.67	73.67
447	97.93	649.53635				ION=[M+H]+		188.15	663.00	1519.00	650.33
448	97.95	826.35104				ION=[M+H]+		980.77	1311.67	868.00	229.00
449	97.95	612.14879	8.491	Oxiglutatione	C20H32N6O12S2	ION=[M+H+H]2+	27025-41-8	30970.62	19369.50	178439.00	61146.67
450	98.01	333.15337	NA	4.8-dimethyl-6-O-(2í.4í-di-O-methyl-?-D-xylopyranosyl)hydroxyquinoline	C18H23NO5	ION=[M+H]+		2848.77	540.00	645.83	3254.00
451	98.05	932.36605				ION=[M+H]+		3802.00	5445.33	10783.83	7652.67
452	98.09	1077.47256				ION=[M+H+H]2+		1344.77	3565.33	3147.33	3357.00
453	98.1	546.33452				ION=[M+H+H]2+		2822.31	8934.67	2182.50	3778.67
454	98.12	1687.69228				ION=[M+H+H2]3+		861.08	793.17	2334.50	1555.67
455	98.12	597.16868				ION=[M+H]+		3.38	6067.17	4.50	0.00
456	98.13	1065.94519				ION=[M+H+H]2+		838.00	715.33	2499.00	1323.00
457	98.14	1149.63357				ION=[M+H+H2]3+		2105.69	3445.17	1917.17	0.00
458	98.16	538.49317	15.172	4,5-ditridecyl-octanedioic acid	C34H66O4	ION=[M+H+H2]3+		14157.69	0.00	2587.17	0.00
459	98.19	1287.56315				ION=[M+H+H]2+		332.92	486.17	2677.17	8700.33
460	98.2	627.23579				ION=[M+H+H]2+		326.92	2790.67	0.00	0.00
461	98.22	374.18358				ION=[M+H]+		2796.15	684.50	1018.50	2405.00
462	98.22	542.31764				ION=[M+H+H]2+		2762.62	991.50	1319.83	680.33
463	98.23	855.50257				ION=[M+H+H2]3+		3428.31	696.67	1568.33	2722.67
464	98.25	608.26654				ION=[M+H]+		1464.31	1325.83	3482.67	2640.00
465	98.29	1711.75315				ION=[M+H+H2]3+		1692.46	4153.50	5267.00	4941.33
466	98.31	874.4443				ION=[M+H+H]2+		342.00	1047.33	2356.33	0.00
467	98.31	344.20589	NA	Oxyphencyclimine	C20H28N2O3	ION=[M+H]+	125-53-1	3492.77	1320.67	703.50	2006.67
468	98.32	538.73785				ION=[M+H]+		2398.46	2140.00	6746.83	3762.00
469	98.32	1520.68671				ION=[M+H+H]2+		338.00	798.83	1220.00	303.67
470	98.32	413.17014				ION=[M+H]+		0.00	0.00	2814.83	0.00
471	98.32	432.59484				ION=[M+H]+		146.31	140.67	495.83	0.00
472	98.32	568.2483				ION=[M+H]+		1150.62	747.17	3116.67	750.67
473	98.32	1372.59786				ION=[M+H+H]2+		33.54	73.17	627.50	50.33
474	98.32	431.18961				ION=[M+H]+		1418.46	1371.33	3317.50	1996.33
475	98.32	568.56822				ION=[M+H]+		567.38	0.00	1897.17	0.00
476	98.32	758.33491				ION=[M+H]+		315.54	758.83	1049.17	101.00
477	98.32	970.29587				ION=[M+H+H2]3+		1192.15	1835.17	1809.00	16.33
478	98.32	1520.18486				ION=[M+H+H]2+		177.23	504.83	1001.67	229.00
479	98.32	386.2517	3.904	Circinamide	C18H34N4O5	ION=[M+H+H]2+		482.46	290.00	205.17	34624.00
480	98.34	608.46477				ION=[M+H]+		1281.23	297.83	2042.17	649.67
481	98.34	519.824				ION=[M+H]+		1650.62	669.17	3378.17	2291.67
482	98.39	1299.57016				ION=[M+H+H]2+		2355.69	1686.00	5402.83	4012.67

483	98.4	587.32571				ION=[M+H+H]2+		1895.23	2137.17	1971.67	1676.00
484	98.4	1298.56986				ION=[M+H+H]2+		670.77	1580.67	2284.17	1441.00
485	98.4	437.21965				ION=[M+H]+		0.00	610.50	0.00	112.33
486	98.41	847.85703				ION=[M+H+H]2+		920.00	443.17	2349.83	592.33
487	98.42	1074.4517				ION=[M+H+H]2+		2932.15	2910.17	7172.50	4738.67
488	98.44	313.61734				ION=[M+H]+		0.00	424.17	0.00	0.00
489	98.44	1251.53561				ION=[M+H+H2]3+		1997.23	874.83	3654.33	29.67
490	98.45	499.19076				ION=[M+H]+		16.31	18352.67	0.00	0.00
491	98.45	385.3304				ION=[M+H+H]2+		117.08	16409.50	73.17	0.00
492	98.47	555.12642				ION=[M+H+H]2+		22049.85	17822.00	52044.17	41869.33
493	98.49	985.26415				ION=[M+H+H]2+		1345.69	1922.33	1849.50	541.67
494	98.52	2154.95522				ION=[M+H+H2]3+		1394.15	1659.00	3051.17	1530.67
495	98.57	1077.96833				ION=[M+H+H]2+		6156.62	5417.17	14900.17	9068.67
496	98.58	578.32297				ION=[M+H+H]2+		0.00	452.67	0.00	1006.67
497	98.59	1295.55776				ION=[M+H+H]2+		1418.31	2407.00	3689.00	1868.00
498	98.61	352.32005				ION=[M+H+H]2+		521.85	0.00	0.00	0.00
499	98.62	986.27895				ION=[M+H+H]2+		1544.92	2270.67	3479.17	152.33
500	98.63	642.3969				ION=[M+H+H2]3+		920.31	0.00	0.00	0.00
501	98.63	207.15872				ION=[M+H]+		390.00	0.00	0.00	0.00
502	98.7	448.68822				ION=[M+H]+		0.00	0.00	595.50	1390.67
503	98.7	855.87167				ION=[M+H+H]2+		1101.85	3151.00	3280.00	2783.67
504	98.7	1694.72744				ION=[M+H+H2]3+		1616.46	692.50	3455.67	1619.67
505	98.71	716.37638				ION=[M+H+H]2+		3117.85	2713.67	4581.83	7303.33
506	98.72	192.17315				ION=[M+H]+		2015.08	0.00	0.00	0.00
507	98.75	1712.73609				ION=[M+H+H2]3+		9775.08	5891.83	22134.83	14196.67
508	98.79	563.41123				ION=[M+H+H]2+		7186.77	0.00	0.00	0.00
509	98.79	1065.44501				ION=[M+H+H]2+		1358.00	992.33	3024.00	1867.33
510	98.82	289.16142	NA	2-[(3-Phenyl-1H-pyrazol-4-yl)methyl]-1,2,3,4-tetrahydroisoquinoline	C19H19N3	ION=[M+H]+		5087.08	1117.50	1419.00	4324.33
511	98.86	1112.9965				ION=[M+H+H]2+		288.92	1388.00	2420.17	632.00
512	98.87	606.29395				ION=[M+H+H]2+		1035.38	1026.83	791.33	676.33
513	98.94	384.34486				ION=[M+H+H]2+		2442.46	1019.67	41.83	0.00
514	98.97	414.32011				ION=[M+H+H]2+		9593.38	0.00	7.33	0.00
515	98.98	431.58307				ION=[M+H]+		345.38	338.50	1148.17	636.33
516	98.99	856.36573				ION=[M+H+H]2+		6358.00	4857.00	17339.33	6728.67
517	99.06	612.28857				ION=[M+H+H]2+		0.00	492.83	0.00	0.00
518	99.07	1068.95645				ION=[M+H+H]2+		921.85	402.50	1762.17	1109.67
519	99.07	2155.93968				ION=[M+H+H2]3+		1583.54	1220.00	3688.83	1927.33
520	99.07	1300.06677				ION=[M+H+H]2+		1332.92	1073.67	4019.00	2755.67
521	99.08	502.32853				ION=[M+H+H]2+		2516.77	24.17	0.00	0.00
522	99.1	430.98647	NA	Benzthiazide	C15H14ClN3O4S3	ION=[M+H]+	91-33-8	519.38	1095.50	1641.50	1116.67
523	99.12	643.78131				ION=[M+H]+		0.00	16.50	0.00	1391.00
524	99.17	856.86513				ION=[M+H+H]2+		3815.85	3582.83	13869.67	6627.33
525	99.2	1078.46641				ION=[M+H+H]2+		4885.08	3975.83	13733.67	7419.00
526	99.28	356.20599				ION=[M+H]+		2644.46	1271.67	2478.33	1582.33
527	99.29	328.28458				ION=[M+H]+		0.00	22.00	6287.00	0.00
528	99.38	1296.05656				ION=[M+H+H]2+		1788.15	2184.17	4321.17	2239.33
529	99.5	616.33972	31.453	Aeruginosin A	C34H44N6O5	ION=[M+H+H]2+		771.54	2975.83	3347.33	11923.00
530	99.52	1292.5459				ION=[M+H+H]2+		1419.85	1432.67	2555.83	1660.67
531	99.53	319.1676				ION=[M+H]+		2204.00	0.00	313.00	3223.00
532	99.55	897.37676				ION=[M+H+H]2+		4911.54	8380.17	3307.17	4705.33
533	99.56	607.3035				ION=[M+H+H]2+		1169.54	272.50	593.17	578.00
534	99.59	401.2269				ION=[M+H]+		4619.54	69.17	299.67	511.33
535	99.59	243.64778				ION=[M+H]+		431.69	0.00	0.00	0.00
536	99.64	287.18303				ION=[M+H]+		4402.62	815.17	1616.33	4067.33
537	99.65	1113.9843				ION=[M+H+H]2+		452.92	977.17	3007.17	790.67
538	99.73	844.42459				ION=[M+H+H]2+		3801.69	1812.00	1120.00	2251.67
539	99.81	266.07697	NA	6-Methyl-3-[4-(trifluoromethyl)phenyl]-1H-pyrazolo[5,1-c][1,2,4]triazole	C12H9F3N4	ION=[M+H]+		1121.23	3274.00	23.83	0.00
540	99.86	428.27465				ION=[M+H+H]2+		448.77	24.17	613.83	5018.67
541	99.88	248.73278				ION=[M+H]+		0.00	0.00	456.67	0.00
542	99.88	431.38464				ION=[M+H]+		848.92	719.00	2896.00	1570.67
543	99.88	1104.47995				ION=[M+H+H]2+		0.00	87.67	2072.83	0.00
544	100.01	388.19637				ION=[M+H]+		4814.92	1575.33	1709.33	3423.67
545	100.01	482.3301				ION=[M+H+H]2+		2791.08	18.17	0.00	0.00
546	100.01	358.21648	NA	Cannabidiolic acid	C22H30O4	ION=[M+H]+		2581.23	513.00	263.50	2534.33
547	100.08	357.29807				ION=[M+H+H]2+		12.77	6158.83	46.33	125.67
548	100.09	235.08929	2.025	S-(d-carboxybutyl)-l-homocysteine	C9H17NO4S	ION=[M+H]+		87.08	0.00	63.50	56751.00
549	100.1	1783.77491				ION=[M+H+H2]3+		1016.00	984.83	2651.83	1639.67
550	100.12	379.11188				ION=[M+H]+		3576.15	5355.67	7641.33	4416.67
551	100.15	277.60884				ION=[M+H]+		677.38	1784.00	2897.17	107.67
552	100.19	303.17816	13.29	[1-(5-Fluoropentyl)-1H-indazol-3-yl](1-pyrrolidinyl)methanone	C17H22FN3O	ION=[M+H]+		4921.85	912.83	1756.67	4926.00
553	100.24	135.05574				ION=[M+H]+		4742.46	9544.50	10922.33	12221.67

554	100.27	1286.55648				ION=[M+H+H]2+		305.54	862.50	1565.17	1206.00
555	100.29	613.42088				ION=[M+H+H]2+		8162.77	52.83	0.00	0.00
556	100.31	993.52994				ION=[M+H+H2]3+		1881.54	2748.50	3232.17	4765.67
557	100.32	891.88158				ION=[M+H+H]2+		905.23	869.50	2359.17	1490.00
558	100.32	892.38667				ION=[M+H+H]2+		922.77	1040.83	2173.17	1178.00
559	100.35	924.50675				ION=[M+H+H2]3+		319.54	1198.33	0.00	0.00
560	100.41	258.17394	NA	Ergotryptamine	C16H22N2O	ION=[M+H]+		2540.00	0.00	0.00	0.00
561	100.45	516.34865				ION=[M+H+H]2+		10332.46	0.00	43.50	0.00
562	100.63	233.12699				ION=[M+H]+		370.00	1399.50	0.00	8599.33
563	100.68	524.22303				ION=[M+H]+		940.31	1067.17	1941.00	1468.67
564	100.74	1296.55217				ION=[M+H+H]2+		1746.77	1715.50	3512.50	2095.33
565	100.75	502.205				ION=[M+H+H]2+		528.31	0.00	0.00	0.00
566	100.83	1753.7663				ION=[M+H+H2]3+		540.92	1321.50	2426.33	2288.67
567	100.93	317.10811	NA	Oxyphenisatin	C20H15NO3	ION=[M+H]+	125-13-3	1411.54	0.00	985.33	0.00
568	100.93	179.52995				ION=[M+H]+		1257.23	874.33	168.83	0.00
569	100.93	972.49001				ION=[M+H+H]2+		1048.92	320.17	104.17	759.00
570	100.93	303.65453				ION=[M+H]+		438.92	28.50	5.33	44.67
571	100.94	436.25411				ION=[M+H+H]2+		3578.62	0.00	843.33	0.00
572	101.01	201.10151	NA	(3'-hydroxybutan-20-yl)5-oxopyrrolidine-2-carboxylate	C9H15NO4	ION=[M+H]+		55.85	4246.83	782.17	516.67
573	101.04	1113.48316				ION=[M+H+H]2+		674.77	1123.00	3130.00	915.67
574	101.13	112.02673				ION=[M+H]+		8888.92	4251.50	6510.17	10556.00
575	101.15	293.13239	NA	Dnc007141	C18H16FN3	ION=[M+H]+		1740.15	670.67	1538.83	3153.00
576	101.24	342.29998				ION=[M+H]+		0.00	0.00	11689.50	0.00
577	101.25	300.14446				ION=[M+H]+		174.46	3988.17	948.33	15468.00
578	101.26	155.09535	NA	Arecoline	C8H13NO2	ION=[M+H]+		36.00	0.00	5693.17	25.67
579	101.29	317.16017				ION=[M+H]+		3639.38	784.17	1851.17	3316.33
580	101.4	455.30919				ION=[M+H+H]2+		164.62	106.00	87.33	12247.33
581	101.45	415.20817				ION=[M+H]+		305.54	4016.83	1337.67	18625.00
582	101.49	442.32786				ION=[M+H+H]2+		48888.00	0.00	0.00	0.00
583	101.5	218.12719	9.513	Meprobamate	C9H18N2O4	ION=[M+H]+	57-53-4	24252.31	7853.33	7645.00	15393.67
584	101.54	705.36455				ION=[M+H+H]2+		1452.77	97.00	66.67	164.33
585	101.59	571.21672				ION=[M+H]+		0.00	5654.00	118.83	197.33
586	101.67	617.36916				ION=[M+H+H]2+		6775.08	20218.50	1033.50	912.33
587	101.95	2148.91393				ION=[M+H+H2]3+		1320.62	1794.50	3276.67	1012.00
588	102.01	333.18676				ION=[M+H]+		2101.38	0.00	907.33	2745.00
589	102.16	558.27726				ION=[M+H+H]2+		357.23	169.00	58.17	10383.33
590	102.21	488.14587				ION=[M+H+H]2+		0.00	0.00	1001.50	0.00
591	102.5	311.29155				ION=[M+H+H]2+		8232.77	0.00	117.00	25.67
592	102.58	665.27972				ION=[M+H]+		1895.54	2874.00	4493.83	3409.00
593	102.64	443.88164				ION=[M+H+H]2+		8949.38	27.00	0.00	0.00
594	102.96	428.28824				ION=[M+H+H]2+		150.00	0.00	0.00	5539.67
595	103.01	735.41018				ION=[M+H+H2]3+		85.23	119.00	441.33	0.00
596	103.05	595.36513				ION=[M+H+H]2+		20.77	0.00	2499.67	0.00
597	103.24	402.21143				ION=[M+H]+		1352.62	266.83	378.17	10619.33
598	103.44	720.36046				ION=[M+H+H]2+		1852.92	70.83	202.17	0.00
599	103.53	390.17994	NA	3-{3-[(2E)-3,7-Dimethyl-2,6-octadien-1-yl]-4-hydroxyphenyl}-7-hydroxy-4H-chromen-4-one	C25H26O4	ION=[M+H]+		1346.46	394.17	430.17	1584.00
600	103.67	345.00289				ION=[M+H]+		1865.85	2259.17	2825.67	944.33
601	103.77	146.04367				ION=[M+H]+		0.00	0.00	27280.00	0.00
602	103.94	1108.47275				ION=[M+H+H]2+		847.54	782.33	3310.00	2113.33
603	103.98	777.45955				ION=[M+H+H2]3+		841.54	55.00	0.00	0.00
604	104.28	276.13143				ION=[M+H]+		2452.15	487.17	2788.67	2762.67
605	104.29	595.18935				ION=[M+H]+		0.00	5029.00	0.00	0.00
606	104.32	694.1635				ION=[M+H+H]2+		586.46	810.67	2526.83	71.33
607	104.6	615.27444				ION=[M+H]+		174.46	33267.83	214.17	392.00
608	104.75	650.20554				ION=[M+H]+		581.38	34.00	4923.50	695.00
609	104.78	361.18307				ION=[M+H]+		2986.15	809.33	1046.83	2248.67
610	104.95	718.35589				ION=[M+H+H]2+		930.00	214.17	282.83	378.33
611	105.23	221.1025	8.525	Metaxalone	C12H15NO3	ION=[M+H]+	1665-48-1	3361.08	786.83	1150.83	1387.67
612	105.56	186.1745				ION=[M+H]+		3839.54	0.00	11.17	0.00
613	105.63	540.33093				ION=[M+H]+		9126.46	0.00	0.00	0.00
614	105.88	507.27701				ION=[M+H+H]2+		1830.15	121.50	720.83	312.00
615	106.26	389.18129				ION=[M+H]+		930.92	1665.50	940.50	1286.33
616	106.4	436.96593				ION=[M+H]+		1599.85	2368.83	1015.17	52.33
617	106.46	568.3517				ION=[M+H]+		4540.62	10.17	0.00	0.00
618	106.47	394.11216				ION=[M+H+H]2+		546.31	1586.00	2945.33	1006.67
619	106.62	686.37046				ION=[M+H+H]2+		1818.15	2125.83	371.33	287.00
620	106.66	910.32642				ION=[M+H+H]2+		1018.00	172.33	0.00	0.00
621	106.7	421.99933				ION=[M+H+H]2+		782.15	1218.67	430.33	0.00
622	106.8	543.26518				ION=[M+H]+		0.00	698.33	0.00	3397.33
623	106.8	252.18413				ION=[M+H]+		0.00	0.00	25.50	3941.00
624	106.8	455.16406				ION=[M+H]+		4952.00	1558.17	6597.17	6797.33
625	106.97	189.06521	9.351	N-Acetyl-DL-glutamic acid	C7H11NO5	ION=[M+H]+		1564.00	1074.50	3857.83	1904.00

626	107.2	450.25838				ION=[M+H+H]2+		143.69	128.83	83.00	3883.33
627	107.55	261.5407				ION=[M+H]+		646.00	995.00	1490.17	0.00
628	107.68	494.2505				ION=[M+H+H]2+		102.15	52.33	0.00	5615.33
629	107.72	523.08191				ION=[M+H+H]2+		1076.77	0.00	159.67	0.00
630	107.82	507.12222				ION=[M+H]+		2106.46	2006.67	1391.17	0.00
631	107.92	322.08374				ION=[M+H]+		0.00	2411.00	0.00	85.67
632	108.03	174.53341				ION=[M+H]+		2002.15	814.33	1484.67	84.67
633	108.36	408.10501				ION=[M+H+H]2+		2343.54	965.83	2124.83	0.00
634	108.37	624.16443				ION=[M+H+H]2+		2005.08	281.33	1734.50	0.00
635	108.45	390.09062				ION=[M+H+H]2+		1899.69	1181.17	2597.50	609.33
636	108.56	349.06583				ION=[M+H+H]2+		0.00	0.00	426.67	22.33
637	108.95	586.21684				ION=[M+H+H]2+		0.00	1165.83	0.00	0.00
638	109.4	561.19222				ION=[M+H]+		4238.46	106.33	2594.50	1707.00
639	109.51	276.07605	NA	Clenbuterol	C12H18Cl2N2O	ION=[M+H]+		708.31	242.67	0.00	0.00
640	109.51	741.13077				ION=[M+H]+		1080.62	1234.50	1029.33	0.00
641	109.71	811.2894				ION=[M+H]+		1529.08	981.83	2408.67	0.00
642	109.91	827.24263				ION=[M+H+H]2+		3448.00	1558.17	4294.00	126.00
643	110.19	1014.36987				ION=[M+H]+		1062.31	1291.17	2361.33	0.00
644	110.3	726.15991				ION=[M+H+H]2+		2465.54	2737.67	2828.17	60.67
645	110.49	549.10413				ION=[M+H]+		1737.08	1919.50	2907.67	986.00
646	110.56	550.10639				ION=[M+H+H]2+		0.00	1028.83	964.17	0.00
647	110.59	802.21151				ION=[M+H+H]2+		1513.54	78.00	1584.67	20.00
648	110.59	536.16369				ION=[M+H]+		2627.38	58.83	1392.83	347.33
649	110.66	552.1438				ION=[M+H+H]2+		2556.15	4096.67	5465.33	0.00
650	110.73	534.13353				ION=[M+H+H]2+		2497.54	3288.17	4519.50	0.00
651	110.78	570.15537				ION=[M+H+H]2+		3458.77	5077.33	6087.33	246.33
652	110.81	1030.32158				ION=[M+H+H]2+		3743.69	5537.33	6431.67	132.00
653	110.97	764.27486	13.782	Boholmycin	C27H48N4O21	ION=[M+H]+		3961.69	110.00	2602.17	1608.00
654	110.99	478.32923				ION=[M+H]+		1463.85	0.00	0.00	0.00
655	111.15	593.17175				ION=[M+H+H]2+		2272.92	3081.67	3712.50	229.33
656	111.2	231.11111				ION=[M+H]+		0.00	5976.33	1373.00	2092.33
657	111.59	694.12799				ION=[M+H+H]2+		0.00	0.00	0.00	926.00
658	111.99	1190.36002				ION=[M+H+H]2+		0.00	254.50	1842.17	0.00
659	112.06	494.17485	36.487			ION=[M+H]+		1067.38	1582.17	5427.00	3087.00
660	112.06	712.17664				ION=[M+H+H]2+		730.31	1141.17	2848.17	0.00
661	112.1	1056.50209				ION=[M+H+H]2+		2242.31	38.50	15.50	70.00
662	112.29	779.28801				ION=[M+H]+		1277.23	1471.17	670.67	1045.00
663	112.43	275.05423	NA	Nifuroxazide	C12H9N3O5	ION=[M+H]+		1619.85	1105.50	1718.50	0.00
664	112.76	462.22288				ION=[M+H+H]2+		0.00	0.00	1433.50	0.00
665	114.16	430.26139				ION=[M+H+H]2+		21.85	12.83	0.00	11304.67
666	114.51	340.19975	15.65	2-[(2-aminocyclohexyl)amino]-4-[(3-methylphenyl)amino]pyrimidine-5-carboxamide	C18H24N6O	ION=[M+H]+	1194961-19-7	0.00	0.00	0.00	8781.00
667	114.81	261.10353				ION=[M+H]+		0.00	0.00	0.00	44972.33
668	115.15	126.0424	3.841	Imidazoleacetic acid	C5H6N2O2	ION=[M+H]+		129.23	2510.00	572.33	42.00
669	115.89	359.20569				ION=[M+H]+		2961.23	1018.00	1117.50	843.33
670	118.38	299.18416				ION=[M+H]+		2281.69	57.33	929.33	680.33
671	118.62	265.11599	15.067	3-fluoro-4-hydroxybenzaldehyde o-(cyclohexylcarbonyl)oxime	C14H16FNO3	ION=[M+H]+		0.00	4347.50	1329.33	0.00
672	119.11	229.09588	13.887	2-(pyrido[1,2-E]purin-4-Yl)amino-Ethanol	C11H11N5O	ION=[M+H]+		1369.38	2007.33	3364.17	743.33
673	119.17	117.07845	4.754	L(+)-norvaline | L-norvaline	C5H11NO2	ION=[M+H]+		2522.00	3504.17	5461.00	1350.00
674	119.33	398.2887	35.992	Hermitamide B	C25H38N2O2	ION=[M+H+H]2+		0.00	0.00	7.83	6939.67
675	119.75	391.28418				ION=[M+H+H]2+		0.00	1546.00	0.00	0.00
676	120.59	363.05755				ION=[M+H]+		751.38	6788.00	2669.50	609.33
677	120.6	348.03342				ION=[M+H+H]2+		95.08	401.67	427.00	0.00
678	121.31	165.07946	7.075	D-Phenylalanine	C9H11NO2	ION=[M+H]+		22562.62	7807.00	15780.83	8926.33
679	122.15	199.14443				ION=[M+H]+		0.00	0.00	0.00	1725.33
680	123.97	534.27231				ION=[M+H]+		2168.77	0.00	5.50	0.00
681	124.13	485.17514				ION=[M+H]+		2162.00	82.00	4514.50	2776.33
682	126.05	513.20609	17.226	Validamycin B	C20H35NO14	ION=[M+H]+		3.54	3902.67	11.17	36.33
683	126.13	258.1937	14.549	Cyclopenta[g]-2-benzopyran, 1,3,4,6,7,8-hexahydro-4,6,6,7,8,8-hexamethyl-	C18H26O	ION=[M+H]+		3816.15	17.17	10.50	50.67
684	126.26	456.36217				ION=[M+H+H]2+		8791.85	246.50	0.00	0.00
685	126.7	280.14166	12.016	Bohemamine E	C14H20N2O4	ION=[M+H]+		3881.85	91.00	673.67	2498.67
686	129.21	1012.31975				ION=[M+H+H]2+		2669.69	1791.00	6010.17	3334.00
687	130.89	290.1661				ION=[M+H]+		41868.46	21.17	16.17	168.00
688	131.88	278.09385	24.091	Pukeleimide A	C13H14N2O5	ION=[M+H]+		6391.69	9178.50	15745.50	9722.33
689	132.5	950.36649				ION=[M+H]+		333.54	0.00	12.00	12811.33
690	134.09	265.09526				ION=[M+H]+		2203.38	896.50	1169.00	560.33
691	134.44	279.12262	NA	Nicotinaldehyde N-(6,7-dihydro-5H-cyclopenta[d][1,2,4]triazolo[1,5-a]pyrimidin-8-yl)hydrazone	C14H13N7	ION=[M+H]+		3886.00	328.33	632.50	2533.00
692	141.48	160.0376	8.878	3,4-dihydroxy-3,4-dihydro-2h-pyran-6-carboxylic acid	C6H8O5	ION=[M+H]+		1245.69	625.50	2362.50	1871.00

693	141.89	618.2139				ION=[M+H]+		1530.92	8.50	33045.50	2317.33
694	143.26	239.10244	8.24	7,8-Dihydrobiopterin	C9H13N5O3	ION=[M+H]+		98.46	0.00	2733.67	106.67
695	144.83	336.13224				ION=[M+H]+		836.00	2853.17	630.67	887.67
696	145.11	236.11599	19.246	Carbetamide	C12H16N2O3	ION=[M+H]+	16118-49-3	8967.54	1064.83	2453.17	6572.00
697	145.38	237.08785	9.88	Biopterin	C9H11N5O3	ION=[M+H]+		2749.85	27.00	7176.00	2382.00
698	145.8	654.1402				ION=[M+H+H]2+		1632.77	10.83	2527.00	185.00
699	146.47	428.27333				ION=[M+H+H]2+		0.00	25.17	0.00	17313.67
700	146.53	185.07015	12.978	Alaremycin	C8H11NO4	ION=[M+H]+		3166.46	751.17	2359.33	2966.67
701	146.71	550.20131				ION=[M+H]+		4264.46	98.17	410.33	2307.67
702	148.16	379.11153				ION=[M+H]+		2135.69	31.00	5385.00	2029.33
703	148.66	266.12667	15.61	2-{2-Oxo-2-[4-(1H-pyrrol-1-yl)piperidino]ethoxy}acetic acid	C13H18N2O4	ION=[M+H]+		2815.69	977.67	1259.33	2312.67
704	149.33	235.09202	NA	S-(d-carboxybutyl)-l-homocysteine	C9H17NO4S	ION=[M+H]+		1544.00	1710.00	1295.33	3957.33
705	151.53	385.33005				ION=[M+H+H]2+		0.00	2972.00	0.00	0.00
706	154.21	274.10305				ION=[M+H]+		30.31	1990.17	0.00	92.67
707	154.28	463.24146				ION=[M+H]+		0.00	5595.00	0.00	1473.00
708	154.44	306.12519				ION=[M+H]+		650.00	431.67	3973.17	167.00
709	156.39	126.04233	12.172	Imidazoleacetic acid	C5H6N2O2	ION=[M+H]+		3155.69	1109.33	1951.83	4599.67
710	156.39	355.14821				ION=[M+H]+		2048.31	1941.67	84.67	1401.33
711	157.64	264.07215	17.226	Streptomyceamide B	C12H12N2O5	ION=[M+H]+		1073.85	1295.33	2496.50	416.67
712	158.18	597.22634				ION=[M+H+H]2+		34.62	2356.50	0.00	0.00
713	160.58	210.99981				ION=[M+H]+		0.00	692.67	847.67	0.00
714	160.71	370.01436				ION=[M+H]+		132.31	3291.17	6829.33	203.00
715	160.72	237.02557				ION=[M+H]+		3.69	1243.67	2593.17	0.00
716	160.8	201.99462				ION=[M+H]+		0.00	0.00	763.00	0.00
717	161.15	559.99165				ION=[M+H+H]2+		11.69	7082.83	17471.83	0.00
718	161.25	574.96261				ION=[M+H]+		0.00	3378.83	7325.00	9.67
719	161.69	418.955				ION=[M+H]+		18.46	1800.00	2712.00	23.00
720	162.02	476.33313	4.536	Cybastacine A	C26H44N4O4	ION=[M+H+H]2+		22986.31	0.00	0.00	0.00
721	162.13	312.01803				ION=[M+H+H]2+		0.00	441.17	2050.50	0.00
722	162.49	313.96091				ION=[M+H]+		52.62	1663.67	1249.67	85.33
723	162.53	298.61331				ION=[M+H]+		146.92	1094.50	13.50	0.00
724	164	627.92172				ION=[M+H+H]2+		8.62	1479.67	2318.00	32.00
725	165.19	363.05791	9.815	5'-Guanylic acid	C10H14N5O8P	ION=[M+H]+		157.69	6767.00	936.33	818.00
726	165.55	599.24052	9.588	Methyl 4-{[4-(dimethylamino)-5-hydroxy-6-methyloxan-2-yl]oxy}-2-ethyl-2,5,12-trihydroxy-7-methoxy-6,11-dioxo-1,2,3,4,6,11-hexahydrotetracene-1-carboxylate	C31H37NO11	ION=[M+H+H]2+		427.54	77009.83	0.00	0.00
727	166.02	581.23086				ION=[M+H+H]2+		112.92	10894.50	5.83	0.00
728	166.35	556.23539	8.969	N-[1,3-bis(morpholin-4-yl)propan-2-yl]-3-nitro-5-{[3,4,5-trihydroxy-6-(hydroxymethyl)oxan-2-yl]oxy}benzamide	C24H36N4O11	ION=[M+H+H]2+		142.92	5842.17	18.50	303.00
729	166.69	151.04952	3.54	Guanine	C5H5N5O	ION=[M+H]+		234.77	3219.67	548.83	415.67
730	168.58	443.1288				ION=[M+H]+		372.46	892.17	1698.83	6766.33
731	168.78	294.12167	15.253	Aspartame	C14H18N2O5	ION=[M+H]+		2871.54	733.50	1035.33	1337.67
732	169.4	208.06132				ION=[M+H]+		3274.15	2091.83	2579.50	3005.33
733	169.88	506.15647				ION=[M+H]+		271.54	589.50	959.67	59.67
734	170.65	456.36232				ION=[M+H+H]2+		1181.08	0.00	0.00	0.00
735	172.13	290.1664	NA	2-(1-{2-Oxo-2-[(2-pyridinylmethyl)amino]ethyl}cyclohexyl)acetic acid	C16H22N2O3	ION=[M+H]+		138.77	2822.33	0.00	0.00
736	172.48	228.18113				ION=[M+H]+		1576.31	9.33	0.00	0.00
737	173.07	315.21451				ION=[M+H]+		2175.69	383.00	553.33	1600.00
738	174	996.34719				ION=[M+H+H]2+		75.85	403.50	1289.00	73.00
739	174	1012.31355				ION=[M+H+H]2+		118.31	90.33	1776.17	0.00
740	174.04	462.32076				ION=[M+H+H]2+		2153.23	0.00	49.50	0.00
741	174.6	540.23954				ION=[M+H+H]2+		43.69	2459.17	0.00	0.00
742	176.08	974.36386				ION=[M+H+H]2+		674.77	2114.00	6214.67	3077.00
743	177.37	803.43232				ION=[M+H+H]2+		75.85	1782.33	282.50	137.33
744	177.43	1010.42248				ION=[M+H+H]2+		1584.15	94.17	3095.50	540.00
745	178.45	418.2419	19.028	Bisucaberin B	C18H34N4O7	ION=[M+H]+		2386.31	1165.50	929.17	140.33
746	180.35	565.23538	12.528	Pyripyropene S	C31H35NO9	ION=[M+H+H]2+		54.92	5523.00	0.00	0.00
747	180.49	583.24551				ION=[M+H+H]2+		307.69	33759.17	0.00	0.00
748	182.57	421.29345				ION=[M+H+H]2+		0.00	4888.50	0.00	0.00
749	185.65	596.14471				ION=[M+H+H]2+		103.69	1885.33	1717.50	0.00
750	185.66	632.32877				ION=[M+H+H]2+		0.00	507.83	0.00	0.00
751	188.52	411.09583				ION=[M+H+H]2+		1523.23	1516.00	2033.00	572.67
753	190.63	359.2058				ION=[M+H]+		1722.46	266.50	375.17	1006.00
754	190.78	313.08451				ION=[M+H]+		6.31	2326.83	6674.00	0.00
755	192.55	235.10615	14.298	1-[(2-amino-9h-purin-6-yl)oxy]-3-methylbutan-2-one	C10H13N5O2	ION=[M+H]+		47.85	75.00	15.33	9326.33
756	192.73	264.14751	17.337	Vorinostat	C14H20N2O3	ION=[M+H]+	149647-78-9	9259.85	1431.17	2427.83	3666.33

757	192.79	310.11684	13.212	6-[2-(4-hydroxy-3-nitrophenyl)acetamido]hexanoic acid	C14H18N2O6	ION=[M+H]+	10463-23-7	1705.08	202.83	3358.83	540.33
758	193.15	504.19733				ION=[M+H]+		0.00	0.00	0.00	903.00
759	193.3	476.33242	1.942	Cybastacine A	C26H44N4O4	ION=[M+H+H]2+		14973.23	127.00	0.00	0.00
760	193.56	468.1167				ION=[M+H+H]2+		85.38	133.33	1742.17	0.00
761	194.28	1008.39412				ION=[M+H+H]2+		0.00	0.00	22.17	3682.33
762	195.38	272.2093				ION=[M+H]+		477904.31	12.00	210.50	2412.67
763	198.66	460.03843				ION=[M+H+H]2+		0.00	1416.33	0.00	0.00
764	199.41	230.01961	13.79	L-xylulose 5-phosphate	C5H11O8P	ION=[M+H]+	108321-99-9	125.85	1426.67	796.50	0.00
765	199.47	151.04949	9.513	Guanine	C5H5N5O	ION=[M+H]+		109.54	3259.67	898.33	93.33
766	201.66	244.17891				ION=[M+H]+		12168.00	2842.17	2123.67	4939.33
767	203.38	206.17963				ION=[M+H]+		0.00	9.33	5.00	4935.67
768	204.78	488.35675				ION=[M+H+H]2+		1528.92	304.00	616.50	0.00
769	209.32	238.14342	10.8	Pirimicarb	C11H18N4O2	ION=[M+H]+	23103-98-2	5882.92	4161.17	3230.33	14137.00
770	213.99	255.1685				ION=[M+H]+		1102.15	710.67	366.67	1121.00
771	215.21	205.10936	13.063	3-[(Dimethylamino)methyl]-2,3-dihydro-4H-chromen-4-one	C12H15NO2	ION=[M+H]+		2505.85	2139.83	921.00	1736.00
772	222.29	470.38058				ION=[M+H+H]2+		0.00	4204.50	0.00	0.00
773	225.11	292.1786	2.395	Carteolol	C16H24N2O3	ION=[M+H]+	51781-06-7	100.46	8647.33	40.67	56.33
774	225.46	468.11605				ION=[M+H+H]2+		1457.38	800.33	4998.17	1506.00
775	230.26	234.05975				ION=[M+H]+		139.54	258.67	1810.83	297.33
776	231.33	612.14235				ION=[M+H+H]2+		205.23	1851.17	1592.17	217.67
777	234.52	343.24737				ION=[M+H]+		2723.08	446.67	0.00	0.00
778	234.52	252.08752	15.667	Cndac	C10H12N4O4	ION=[M+H]+		1025.69	2153.83	39.17	74.67
779	235.47	267.11103	18.407	6-amino-3-methyl-1-phenylpyrazolo[3,4-b]pyridine-5-carboxamide	C14H13N5O	ION=[M+H]+		20.31	1830.17	0.00	0.00
780	235.83	816.42716				ION=[M+H+H]2+		224.77	1113.50	138.50	157.00
781	240.3	330.17914	17.563	L-n(omega)-nitroarginine-(4r)-amino-l-proline amide	C11H22N8O4	ION=[M+H]+		49.54	10732.33	10.33	30.33
782	241.73	252.12126				ION=[M+H]+		2009.54	797.17	350.83	2900.67
783	241.89	363.14297	12.173	Kahakamide B	C17H21N3O6	ION=[M+H]+		34.62	3651.67	20.00	0.00
784	242.23	446.3596				ION=[M+H+H]2+		0.00	1833.33	0.00	0.00
785	244.52	488.35848				ION=[M+H+H]2+		3437.38	1670.67	1409.83	6005.00
786	244.75	583.2459	15.074	3',4'-syn-prepyridomacrolidin B	C31H37NO10	ION=[M+H+H]2+		250.00	27965.17	0.00	0.00
787	245.17	556.24416				ION=[M+H+H]2+		26.00	1066.50	300.17	0.00
788	245.55	1009.56903				ION=[M+H+H2]3+		397.69	1207.50	190.33	61.67
789	246.97	278.12264	14.288	Oxadixyl	C14H18N2O4	ION=[M+H]+	77732-09-3	0.00	4329.00	88.50	0.00
790	248.13	553.23458				ION=[M+H+H]2+		499.69	2053.50	0.00	0.00
791	248.33	631.42987				ION=[M+H+H]2+		5635.38	0.00	0.00	0.00
792	248.48	689.37265				ION=[M+H+H]2+		13234.00	229.33	160.00	144.67
793	249.2	276.61754				ION=[M+H]+		28.92	490.17	0.00	0.00
794	250.86	713.40208	34.883	Microginin 713	C37H55N5O9	ION=[M+H+H]2+		2532.00	1468.33	5953.33	2294.67
795	251.17	603.26432				ION=[M+H+H]2+		0.00	16.83	23.67	10763.33
796	251.24	1266.7011				ION=[M+H+H2]3+		2001.08	109.33	278.67	0.00
797	252.48	1593.72881				ION=[M+H+H2]3+		1821.69	0.00	55.50	119.33
798	252.98	586.24046	47.075	Tolyporphin F	C32H34N4O7	ION=[M+H+H]2+		0.00	99.33	0.00	4001.33
799	253.22	1906.81529				ION=[M+H+H2]3+		442.62	25.00	3962.33	165.33
800	253.77	1447.78849				ION=[M+H+H2]3+		319.23	134.00	2965.83	2115.00
801	254.28	357.26248				ION=[M+H]+		29287.38	0.00	0.00	45.00
802	254.57	514.2591				ION=[M+H]+		25.38	574.83	0.00	0.00
803	254.74	600.35709				ION=[M+H+H]2+		1810.62	180.83	718.17	1006.67
804	254.91	675.35678				ION=[M+H+H]2+		4180.15	1046.33	3426.67	3412.00
805	255.22	251.10243	9.542	2-(2-Hydroxy-Phenyl)-1h-Indole-5-Carboxamidine	C15H13N3O	ION=[M+H]+	179748-10-8	9.69	0.00	0.00	2901.33
806	256.03	272.20957				ION=[M+H]+		1481.54	274937.67	15.00	0.00
807	256.13	1499.79024				ION=[M+H+H2]3+		4351.85	326.50	86.00	38.67
808	256.21	404.35075				ION=[M+H+H]2+		0.00	2285.17	0.00	0.00
809	256.93	1028.51877				ION=[M+H+H]2+		277.38	1162.33	158.50	23.00
810	257.19	1048.57971				ION=[M+H+H2]3+		1179.69	2234.50	3384.50	232.33
811	257.45	1272.63914				ION=[M+H+H2]3+		459.08	397.00	2614.83	606.00
812	257.74	490.34952	7.505	PEG Diethylhexanoate n5	C26H50O8	ION=[M+H+H]2+		123.38	10767.17	80.67	42.67
813	258.01	641.40599				ION=[M+H+H]2+		1736.15	1604.17	4249.33	2365.00
814	258.75	1244.65101				ION=[M+H+H2]3+		1619.69	1503.33	779.00	71.67
815	259.97	157.95403				ION=[M+H]+		6195.38	10319.00	7134.50	5660.00
816	260.51	294.15765	11.932	Cyclocarbamide B	C15H22N2O4	ION=[M+H]+		11509.23	831.00	2782.50	4203.33
817	260.9	470.29322				ION=[M+H+H]2+		0.00	0.00	0.00	103958.00
818	261.21	1038.49306				ION=[M+H+H]2+		110.92	173.83	2994.17	552.00
819	261.83	1229.68769				ION=[M+H+H2]3+		1927.38	69.33	64.33	0.00
820	261.83	1188.65628				ION=[M+H+H2]3+		9139.69	131.00	247.33	202.33
821	262.18	278.16318	14.542	Santacruzamate A	C15H22N2O3	ION=[M+H]+		5712.92	168.00	1470.83	0.00
822	262.2	647.42546				ION=[M+H+H]2+		2370.15	0.00	0.00	0.00
823	263.07	712.87769				ION=[M+H+H]2+		1489.23	1368.67	628.33	0.00
824	263.21	588.30873				ION=[M+H+H]2+		0.00	248.33	60.17	3504.00
825	264.03	1552.73286				ION=[M+H+H]2+		2698.92	9.50	0.00	170.00
826	264.05	292.13589				ION=[M+H]+		0.00	2348.17	64.17	0.00

827	264.29	239.18922				ION=[M+H]+		3939.38	607.33	1757.33	2493.33
828	264.95	158.08476	5.863	Benzylimidazole	C10H10N2	ION=[M+H]+	4238-71-5	2476.31	1149.83	517.33	993.67
829	264.99	335.18544	19.983	JWH-250	C22H25NO2	ION=[M+H]+		4007.08	318.67	1363.17	1506.33
830	266.3	198.98389				ION=[M+H]+		4018.92	4207.17	3553.33	2668.67
831	266.72	890.45414				ION=[M+H+H]2+		14851.85	1776.83	801.17	371.00
832	266.83	669.32399				ION=[M+H]+		50.15	4055.83	61.67	2409.00
833	267.46	346.13706	38.509	Porphyra-334	C14H22N2O8	ION=[M+H]+		70.77	2438.50	114.50	368.00
834	268.51	413.19794				ION=[M+H+H]2+		2931.69	322.50	3163.50	1902.00
835	268.56	454.30043				ION=[M+H+H]2+		0.00	16.67	13.67	4284.33
836	268.88	1214.62966	15.469	Nostoweipeptin W1	C61H86N10O16	ION=[M+H+H2]3+		240.46	3293.00	431.67	259.00
837	269.02	759.42007				ION=[M+H+H]2+		234.46	1509.50	363.17	345.33
838	269.75	818.41563				ION=[M+H+H]2+		4135.23	5099.83	1170.33	784.33
839	269.89	392.21622				ION=[M+H]+		504.31	205.67	59.50	0.00
840	270.11	1718.94294				ION=[M+H+H2]3+		106.62	378.50	3348.33	1632.67
841	270.3	1327.70214				ION=[M+H+H2]3+		440.62	1529.00	276.00	71.00
842	270.38	379.71074				ION=[M+H]+		0.00	481.50	32.83	108.00
843	270.62	1199.58452				ION=[M+H+H2]3+		441.08	788.17	3089.33	115.67
844	271.03	286.22613				ION=[M+H]+		0.00	3789.17	0.00	0.00
845	271.27	784.43147				ION=[M+H+H]2+		1522.46	1783.67	1544.33	2757.00
846	271.39	1965.74744				ION=[M+H+H]2+		0.00	0.00	2948.67	0.00
847	271.43	427.2242				ION=[M+H]+		255.69	2072.17	369.67	301.33
848	271.47	486.26999				ION=[M+H+H]2+		0.00	12.50	0.00	9427.33
849	271.73	1116.56911				ION=[M+H+H]2+		1621.85	401.17	421.33	667.67
850	271.84	853.95021				ION=[M+H+H]2+		16.15	2127.83	153.50	0.00
851	271.91	1275.67588				ION=[M+H+H]2+		1170.62	764.17	0.00	0.00
852	271.99	982.8742				ION=[M+H]+		0.00	0.00	456.83	0.00
853	272.06	351.18096				ION=[M+H]+		2265.54	201.83	723.50	1148.00
854	272.32	282.16796	5.404	PEG n6	C12H26O7	ION=[M+H]+		5910.46	2765.33	2030.67	11064.00
855	272.38	299.19445				ION=[M+H]+		6151.38	1800.17	1258.17	8292.00
856	272.88	677.30873				ION=[M+H]+		1837.08	118.00	23.67	140.00
857	273.05	1029.53869				ION=[M+H+H]2+		1445.08	185.17	152.33	18.00
858	273.16	459.18721				ION=[M+H]+		3757.85	0.00	142.17	1902.00
859	273.7	1289.60121				ION=[M+H+H2]3+		1562.15	0.00	822.00	769.67
860	274.68	680.22564				ION=[M+H+H]2+		144.77	3020.33	39.50	58.00
861	275.38	326.13473	17.798	4-{6-[(1-hydroxybutan-2-yl)amino]imidazo[1,2-b]pyridazin-3-yl}benzoic acid	C17H18N4O3	ION=[M+H]+		1453.23	1315.33	2999.33	696.33
862	276.27	1122.6481				ION=[M+H+H2]3+		2306.62	816.83	274.83	630.67
863	276.55	1002.49315				ION=[M+H+H]2+		12966.31	2172.50	1797.00	1264.00
864	276.8	1256.68996				ION=[M+H+H2]3+		65.23	8046.83	190.50	370.67
865	277.16	520.35872				ION=[M+H+H]2+		0.00	5127.83	0.00	0.00
866	277.24	889.45137				ION=[M+H+H]2+		5649.69	9336.33	880.33	441.00
867	278.06	3811.43346				ION=[M+H+H2]3+		9.85	1584.50	0.00	0.00
868	278.95	201.04766	8.201	Diphenylphosphine oxide	C12H10OP	ION=[M+H]+		2229.69	2440.67	1072.50	6223.00
869	279.1	980.373				ION=[M+H+H]2+		0.00	0.00	1008.17	8253.00
870	279.56	927.46054				ION=[M+H+H]2+		14.92	1371.50	14348.67	108553.33
871	279.59	927.47234				ION=[M+H]+		10.92	332.00	5165.17	39268.33
872	279.63	812.39829				ION=[M+H+H]2+		129.08	514.83	4479.67	36608.67
873	279.72	767.48648				ION=[M+H+H]2+		0.00	1452.50	235.00	175.00
874	280.07	1294.64459				ION=[M+H+H2]3+		1694.62	217.17	2007.33	908.33
875	280.47	965.40327				ION=[M+H+H2]3+		0.00	260.67	2625.67	1605.67
876	280.7	676.38568				ION=[M+H+H]2+		1489.23	385.50	132.67	48.67
877	280.8	714.40237				ION=[M+H+H]2+		158.00	0.00	339.00	866.00
878	281.91	1114.55122				ION=[M+H+H]2+		1213.69	0.00	0.00	0.00
879	281.94	557.27386				ION=[M+H]+		2169.08	768.00	772.17	2144.67
880	283.02	383.18008				ION=[M+H]+		4126.31	2088.17	2274.00	4718.67
881	283.14	1550.71878				ION=[M+H+H2]3+		213.85	458.50	2330.83	21.00
882	283.24	190.08539	8.433	Diethyleneglycol diacetate	C8H14O5	ION=[M+H]+		3012.15	1873.67	1680.50	3754.33
883	284.16	398.13226				ION=[M+H]+		2341.69	27.17	0.00	0.00
884	284.64	1187.60902				ION=[M+H+H]2+		3328.15	337.33	207.00	25.33
885	285.18	975.47807				ION=[M+H+H]2+		2040.31	710.67	77.50	40.00
886	285.18	701.39077				ION=[M+H+H]2+		3206.92	1058.17	290.00	3421.00
887	285.94	322.18816	12.991	N-ethyl-3-fluoro-3-[3-fluoro-4-(pyrrolidin-1-ylmethyl)phenyl]cyclobutane-1-carboxamide	C18H24F2N2O	ION=[M+H]+		253.85	2419.00	39.17	0.00
888	286.11	345.19069				ION=[M+H]+		2110.15	1301.17	1013.33	2323.33
889	286.99	165.07959	6.912	D-Phenylalanine	C9H11NO2	ION=[M+H]+		2081.38	2691.83	4214.00	6235.67
890	287.07	252.65019				ION=[M+H]+		431.38	113.17	0.00	0.00
891	287.6	746.39239				ION=[M+H+H]2+		2234.00	694.17	6411.17	2713.00
892	287.68	313.1744				ION=[M+H]+		2467.54	586.67	327.67	3006.67
893	287.79	828.37201				ION=[M+H+H]2+		887.08	77.33	0.00	0.00
894	287.94	505.30196	17.92	Borrelidin C	C28H43NO7	ION=[M+H+H]2+		1291.08	1637.17	1111.17	682.67
895	288.16	296.14603	14.772	Caryophyllose	C12H24O8	ION=[M+H]+		1425.69	411.00	220.00	2666.33
896	288.5	881.47987				ION=[M+H+H]2+		546.62	3171.50	710.67	651.33
897	288.57	294.12162	3.758	Aspartame	C14H18N2O5	ION=[M+H]+		59969.85	69789.33	133169.00	160222.33
898	288.82	414.18695	NA	Ptesculentoside	C20H30O9	ION=[M+H]+		623.38	407.00	0.00	0.00

899	288.93	1386.69864				ION=[M+H+H2]3+		641.54	4796.17	398.83	570.67
900	289	579.52049				ION=[M+H]+		0.00	26.83	1641.83	46.00
901	289.26	896.52843				ION=[M+H+H]2+		0.00	1369.17	0.00	0.00
902	289.34	579.71916				ION=[M+H]+		8.77	0.00	1460.00	0.00
903	289.44	303.1593				ION=[M+H]+		2549.85	446.00	333.83	1757.33
904	290.05	900.51698				ION=[M+H+H]2+		1301.38	214.33	261.17	171.33
905	290.09	1216.64329				ION=[M+H+H2]3+		264.77	14.83	3082.00	131.00
906	290.81	1500.74706				ION=[M+H+H2]3+		198.46	2353.33	262.50	201.67
907	290.89	1147.56102				ION=[M+H+H]2+		2380.62	368.00	118.33	0.00
908	290.99	518.27837				ION=[M+H]+		2151.08	135.67	58.83	162.67
909	291.69	393.19401	NA	2-{[8-(3-aminopiperidin-1-yl)-1,3-dimethyl-2,6-dioxo-2,3,6,7-tetrahydro-1h-purin-7-yl]methyl}benzonitrile	C20H23N7O2	ION=[M+H]+		2020.00	659.00	655.00	2057.00
910	291.78	943.56359				ION=[M+H+H]2+		11096.92	577.67	172.67	0.00
911	292.79	1211.65828				ION=[M+H+H2]3+		37.69	544.33	2091.83	0.00
912	292.8	240.13669	21.121	12?-Deoxy-decarbamoylsaxitoxin	C9H16N6O2	ION=[M+H]+		2919.38	3674.67	512.83	1066.33
913	292.83	1167.6812				ION=[M+H+H2]3+		1522.00	1541.33	3093.50	214.33
914	292.85	1272.70069				ION=[M+H+H2]3+		3710.00	55.33	200.17	317.33
915	292.96	899.41227				ION=[M+H+H]2+		99.23	1357.67	19.00	0.00
916	293.33	826.42471				ION=[M+H]+		0.00	31.67	615.83	5991.67
917	293.34	941.47543				ION=[M+H+H]2+		0.00	947.83	7454.17	46827.33
918	293.39	941.48829				ION=[M+H]+		0.00	22.00	2301.33	13525.33
919	293.39	826.41182				ION=[M+H+H]2+		74.92	291.67	2346.00	15678.00
920	294.51	1308.74777				ION=[M+H+H2]3+		215.23	1751.00	2807.00	1067.67
921	294.81	279.00683				ION=[M+H]+		0.00	10426.83	0.00	0.00
922	294.84	794.90836				ION=[M+H+H]2+		534.62	1565.00	413.33	16.33
923	294.97	701.36153				ION=[M+H]+		2330.15	104.83	546.33	5903.33
924	294.99	1251.69191				ION=[M+H+H2]3+		2924.00	1809.17	582.83	784.33
925	295.22	361.05971				ION=[M+H]+		635.08	2448.00	553.17	239.33
926	295.25	920.48959				ION=[M+H+H]2+		1863.38	1503.00	1215.17	760.67
927	295.31	794.41143				ION=[M+H+H]2+		957.38	2080.83	742.50	488.00
928	295.38	817.42608				ION=[M+H+H]2+		1611.54	137.83	1779.00	762.00
929	295.54	264.15638				ION=[M+H+H]2+		410.92	0.00	0.00	0.00
930	295.7	1762.87274				ION=[M+H+H2]3+		2174.00	0.00	14.50	11.33
931	296.23	632.36022	7.556	Pseudoxylallemycin D	C36H48N4O6	ION=[M+H+H]2+		844.15	2216.83	399.67	2597.00
932	296.62	1244.65356				ION=[M+H+H2]3+		647.38	297.00	1868.17	497.67
933	296.99	1272.65563				ION=[M+H+H2]3+		599.08	3402.83	619.50	528.33
934	297.12	1586.57294				ION=[M+H+H]2+		24.15	2244.17	11.33	0.00
935	297.35	870.95732				ION=[M+H+H]2+		77.08	1839.33	123.00	45.00
936	297.58	955.56176				ION=[M+H+H]2+		296.92	2701.00	492.17	121.67
937	297.59	1849.90558				ION=[M+H+H2]3+		1426.92	0.00	0.00	29.33
938	297.71	871.46284				ION=[M+H+H]2+		460.00	1329.17	315.67	337.00
939	297.84	1002.40108				ION=[M+H]+		5.08	11.67	0.00	2821.67
940	297.97	325.27284				ION=[M+H]+		16627.08	19.33	6.17	67.67
941	298.06	435.7295				ION=[M+H]+		36.46	420.67	87.83	0.00
942	298.59	2004.80138				ION=[M+H+H]2+		0.00	9.33	0.00	815.00
943	298.62	901.42352				ION=[M+H]+		0.00	2238.17	23.17	0.00
944	299.09	1912.97222				ION=[M+H+H2]3+		121.85	372.50	1739.00	33.33
945	299.56	1856.92486				ION=[M+H+H2]3+		1872.15	99.50	15.67	0.00
946	299.86	1489.71653				ION=[M+H+H2]3+		8260.92	396.33	618.00	968.67
947	299.88	329.23153				ION=[M+H]+		4829.08	1552.83	1702.17	6202.33
948	300.2	525.45659				ION=[M+H+H]2+		2453.85	0.00	0.00	0.00
949	300.67	1784.98109				ION=[M+H+H2]3+		811.38	1843.50	404.50	0.00
950	301.07	378.72534				ION=[M+H]+		416.00	1486.17	388.67	233.00
951	301.16	1264.60152				ION=[M+H+H]2+		3425.38	42.17	253.00	534.00
952	301.18	2052.00162				ION=[M+H+H2]3+		1590.00	44.00	71.33	0.00
953	301.6	343.2205				ION=[M+H]+		5633.23	2109.00	1020.00	6765.00
954	301.71	756.91525				ION=[M+H+H]2+		51.85	2080.83	196.00	10.33
955	302.03	326.19498				ION=[M+H]+		2057.38	1095.00	638.00	3455.33
956	302.13	1042.51902				ION=[M+H+H]2+		1221.69	362.33	268.17	557.67
957	302.27	378.71036				ION=[M+H]+		118.46	1379.83	306.83	96.00
958	302.79	1056.60902	15.965	Thalassospiramide J	C53H84N8O14	ION=[M+H+H]2+		171.38	4467.67	47.67	192.00
959	302.9	1089.56677				ION=[M+H+H]2+		2299.23	1372.83	704.50	455.67
960	303.69	515.29253				ION=[M+H]+		2934.92	244.67	141.00	748.67
961	303.94	422.2161				ION=[M+H]+		1882.15	2580.17	2627.50	971.67
962	304.15	812.45706				ION=[M+H+H]2+		2852.31	397.17	437.83	4298.00
963	304.85	1359.69592				ION=[M+H+H]2+		2111.69	108.67	240.00	34.33
964	304.85	1878.03167				ION=[M+H+H2]3+		3336.92	0.00	536.67	108.00
965	304.88	601.19204				ION=[M+H]+		2213.23	138.17	417.67	234.67
966	304.96	286.22564				ION=[M+H]+		138.00	16627.17	0.00	0.00
967	305.11	1444.75255				ION=[M+H+H2]3+		1444.31	90.83	58.00	18.33
968	305.28	960.02518				ION=[M+H+H]2+		2135.08	18.00	399.33	44.00
969	305.39	959.52298				ION=[M+H+H]2+		2356.00	254.33	361.67	304.33
970	305.4	939.01045				ION=[M+H+H]2+		18873.85	309.50	2534.67	861.00

971	305.58	391.24605				ION=[M+H]+		666.15	5581.33	0.00	0.00
972	305.78	373.21907				ION=[M+H]+		2973.08	1050.67	1377.33	2511.33
973	306.04	947.50033				ION=[M+H+H]2+		1445.08	1330.50	434.83	919.00
974	306.86	1245.63014				ION=[M+H+H]2+		204.15	2528.50	364.50	792.33
975	307.08	583.4626				ION=[M+H+H]2+		2841.69	0.00	0.00	0.00
976	307.28	928.54956				ION=[M+H+H]2+		280.77	2503.50	877.00	105.33
977	307.73	785.9398				ION=[M+H+H]2+		371.08	4263.83	237.17	172.00
978	307.89	412.13469				ION=[M+H]+		2610.62	764.00	1211.00	575.00
979	308.13	320.21029	9.786	Iprovalicarb	C18H28N2O3	ION=[M+H]+	140923-17-7	0.00	5705.83	11.83	0.00
980	308.35	1556.83769				ION=[M+H+H2]3+		1802.31	3600.00	594.50	0.00
981	308.64	1473.73808				ION=[M+H+H2]3+		889.23	2058.50	343.83	474.67
982	308.66	313.27308				ION=[M+H]+		0.00	2131.00	9.67	12.67
983	309.1	1233.6578				ION=[M+H+H2]3+		599.23	180.17	2630.50	344.33
984	309.5	1330.71205				ION=[M+H+H2]3+		361.38	3638.33	339.83	527.33
985	310.15	998.56917				ION=[M+H+H]2+		4366.00	856.33	551.83	0.00
986	310.15	1487.73668				ION=[M+H+H2]3+		6851.38	68.67	853.50	617.00
987	310.33	1129.62753				ION=[M+H+H]2+		6456.46	903.83	328.50	75.00
988	310.47	1062.53227				ION=[M+H+H]2+		3358.92	1902.33	4055.67	2659.00
989	310.69	1691.77555				ION=[M+H+H2]3+		6946.46	224.17	1350.00	416.33
990	310.93	221.09561				ION=[M+H]+		581.54	395.67	2653.83	434.00
991	311.2	597.4772				ION=[M+H+H]2+		129638.00	0.00	0.00	466.33
992	311.44	872.50438	19.096	Marformycin C	C43H68N8O11	ION=[M+H+H]2+		363.54	6814.67	130.67	652.00
993	311.51	810.42817	6.01	[DMAdda3]Nodularin-R	C40H58N8O10	ION=[M+H]+		4.62	54.33	1352.17	11911.00
994	311.51	925.49361				ION=[M+H]+		0.00	128.00	1844.83	18950.00
995	311.64	1792.82477				ION=[M+H+H2]3+		3156.92	0.00	378.00	0.00
996	311.66	810.41702				ION=[M+H+H]2+		188.00	325.50	3154.17	28799.00
997	311.97	925.48098				ION=[M+H+H]2+		26.77	944.33	5342.50	46177.00
998	311.99	1190.58766	14.482	Janthinocin B	C57H82N12O16	ION=[M+H+H]2+		1821.54	229.67	917.83	1395.67
999	312.09	180.08293				ION=[M+H]+		1066.00	1975.50	198.50	39.33
1000	312.48	385.10644				ION=[M+H]+		0.00	2797.33	10.33	22.67
1001	313.13	790.24311				ION=[M+H+H]2+		0.00	2095.50	0.00	0.00
1002	313.24	816.42013				ION=[M+H+H]2+		1479.38	1470.50	270.50	1256.67
1003	314.12	1505.80699				ION=[M+H+H2]3+		159.69	1779.67	15.50	75.67
1004	314.44	968.40283				ION=[M+H]+		0.00	0.00	0.00	5148.67
1005	314.89	357.20047	18.591	AB-CHMINACA metabolite M2	C20H27N3O3	ION=[M+H]+		2611.23	638.83	489.50	3607.33
1006	314.93	879.46332				ION=[M+H+H]2+		1522.00	7318.00	1094.00	4592.67
1007	315.64	315.21589				ION=[M+H]+		4090.62	1102.50	1746.83	4591.67
1008	315.67	1668.91484				ION=[M+H+H2]3+		1951.08	218.83	67.50	361.33
1009	316.3	808.42651				ION=[M+H+H]2+		9618.46	16533.50	3090.17	1002.67
1010	316.43	1613.82694				ION=[M+H+H2]3+		580.62	1617.33	171.83	83.67
1011	317.14	1433.72121				ION=[M+H+H2]3+		14.62	1710.17	21.17	27.33
1012	317.65	371.27844				ION=[M+H]+		0.00	8352.00	0.00	0.00
1013	317.84	1184.64223				ION=[M+H+H]2+		1461.69	87.17	0.00	78.00
1014	318.9	1447.67676				ION=[M+H+H]2+		13.23	599.83	32.00	37.67
1015	318.92	770.30899				ION=[M+H+H]2+		417.38	97.83	1795.00	0.00
1016	319.22	1329.70812				ION=[M+H+H]2+		2320.31	2137.33	466.33	25.00
1017	319.26	572.28209	NA	Anabaenolysin var 9	C29H40N4O8	ION=[M+H]+		107.54	544.50	36.33	87.00
1018	319.36	1202.65218				ION=[M+H+H]2+		1778.62	165.33	132.67	492.67
1019	319.8	1403.74301				ION=[M+H+H2]3+		542.31	2455.83	85.50	23.00
1020	319.82	1604.73987				ION=[M+H+H2]3+		1479.54	20.33	302.67	300.67
1021	319.86	294.10862	16.155	7-Methoxy-6-(1,2,3-trihydroxy-3-methylbutyl)-2H-chromen-2-one	C15H18O6	ION=[M+H]+		42.77	126.00	2399.00	108.00
1022	319.87	1692.94856				ION=[M+H+H2]3+		548.15	138.17	2024.67	785.00
1023	320.14	900.005				ION=[M+H+H]2+		0.00	2057.50	61.67	0.00
1024	320.23	236.0927	17.875	Carbazochrome	C10H12N4O3	ION=[M+H]+	69-81-8	805.38	2992.83	613.50	89.67
1025	320.26	574.32215				ION=[M+H]+		189.69	52.83	3564.50	101.33
1026	320.33	899.50324				ION=[M+H+H]2+		121.23	1982.00	141.50	76.00
1027	320.56	1685.88791				ION=[M+H+H2]3+		2056.15	153.33	53.83	0.00
1028	320.72	500.32066				ION=[M+H]+		10921.38	0.00	392.50	0.00
1029	320.77	1466.72755				ION=[M+H+H]2+		254.92	85.17	2810.67	243.67
1030	320.85	1466.22537				ION=[M+H+H]2+		0.00	0.00	2502.67	62.00
1031	321.01	1465.72727				ION=[M+H+H]2+		55.23	9.33	1691.17	31.67
1032	321.04	579.98282				ION=[M+H]+		5120.62	231.00	26.00	41.33
1033	321.05	680.22516				ION=[M+H+H]2+		80.31	3755.67	0.00	38.67
1034	321.36	1741.94534				ION=[M+H+H2]3+		2761.23	90.50	0.00	13.33
1035	321.66	212.10657	9.336	Rabenzazole	C12H12N4	ION=[M+H]+		5528.00	6287.00	3704.67	2167.33
1036	321.74	1040.57494				ION=[M+H+H]2+		2549.85	364.67	1996.33	883.67
1037	322.03	381.13291	16.154	Metatacarboline A	C20H19N3O5	ION=[M+H]+		460.00	18566.33	1114.33	552.67
1038	322.14	953.47452	26.042	[Asp3]MC-LE	C47H67N7O14	ION=[M+H+H]2+		93.08	64.00	622.50	7797.33
1039	322.22	288.11126	14.61	Ethyl 2-cyano-2-[4-(hydroxymethyl)-5-phenyl-1,3-oxazolan-2-yliden]acetate	C15H16N2O4	ION=[M+H]+		2770.62	2654.17	2017.00	2831.67
1040	322.78	824.4453				ION=[M+H]+		10.62	0.00	436.67	4709.00
1041	322.78	838.41232				ION=[M+H+H]2+		6.46	105.33	294.83	3779.67
1042	322.82	824.43298				ION=[M+H+H]2+		278.46	102.33	1891.00	14494.67

1043	322.82	939.49611				ION=[M+H+H]2+		0.00	135.67	2306.67	17753.67
1044	322.93	465.22145				ION=[M+H]+		340.46	28.00	2052.67	94.00
1045	322.97	1160.60882				ION=[M+H+H]2+		19686.31	3914.17	2692.83	866.00
1046	323.16	1362.66363				ION=[M+H+H]2+		1398.15	304.50	236.33	196.00
1047	323.57	470.34649				ION=[M+H]+		10312.92	0.00	0.00	0.00
1048	324.04	515.94599				ION=[M+H]+		407.23	0.00	0.00	0.00
1049	324.43	1689.79639				ION=[M+H+H2]3+		2776.46	95.83	446.33	511.33
1050	324.62	1547.8408				ION=[M+H+H2]3+		1058.46	353.83	162.00	31.33
1051	324.86	1073.57249				ION=[M+H+H]2+		21032.92	5121.83	1774.17	1037.33
1052	325.09	1810.9886				ION=[M+H+H2]3+		1270.31	265.50	15.00	0.00
1053	325.62	1536.82342				ION=[M+H+H2]3+		14730.62	3579.67	894.50	1727.33
1054	325.62	102.06749	3.54	Isovaleric acid	C5H10O2	ION=[M+H]+		1100.77	1799.67	3401.00	1808.00
1055	325.94	146.09435	5.206	Propanoic acid, 3-ethoxy-, ethyl ester	C7H14O3	ION=[M+H]+		1749.69	2526.50	5346.50	2636.67
1056	326.09	1858.96809				ION=[M+H+H2]3+		80.31	68.50	1799.33	216.33
1057	326.1	239.17386				ION=[M+H]+		22427.85	30179.67	42258.67	20562.33
1058	326.26	1788.88842				ION=[M+H+H2]3+		563.69	7249.33	820.33	256.33
1059	326.47	1537.82732				ION=[M+H+H2]3+		11822.92	2919.17	693.50	1427.67
1060	326.53	240.05872				ION=[M+H]+		40.92	2325.00	51.17	0.00
1061	326.82	670.40366				ION=[M+H]+		1223.85	0.00	0.00	0.00
1062	326.93	661.51485				ION=[M+H]+		693.08	0.00	0.00	0.00
1063	326.93	1790.85757				ION=[M+H+H2]3+		1052.46	133.00	272.00	86.67
1064	327.05	1323.53385				ION=[M+H+H]2+		2902.00	999.33	556.33	619.67
1065	327.05	1607.86147				ION=[M+H+H2]3+		6291.69	0.00	742.17	230.00
1066	327.1	216.09292				ION=[M+H]+		3700.31	2388.17	421.83	1464.67
1067	327.11	1323.03331				ION=[M+H+H]2+		1421.23	666.17	453.17	423.67
1068	327.32	1443.79112				ION=[M+H+H2]3+		756.92	4923.33	360.00	306.33
1069	327.6	631.46228				ION=[M+H+H]2+		10375.23	0.00	0.00	21.67
1070	327.71	1400.74519				ION=[M+H+H]2+		1037.54	1903.50	2481.00	90.33
1071	327.72	1138.65348				ION=[M+H+H]2+		0.00	7488.00	3199.17	605.00
1072	328.37	219.0772				ION=[M+H]+		3628.00	1208.83	5573.67	2600.00
1073	328.66	571.1812				ION=[M+H]+		9492.00	32206.83	11981.67	16182.00
1074	329.24	1447.7377				ION=[M+H+H]2+		3033.38	2450.50	333.17	128.33
1075	329.56	347.10304	13.396	Pseurotin H	C17H17NO7	ION=[M+H]+		1277.54	5691.17	1104.83	0.00
1076	329.7	700.26277				ION=[M+H]+		11062.77	1411.50	5444.67	3497.00
1077	330.64	1171.63361				ION=[M+H+H2]3+		13.69	5251.67	35.67	97.67
1078	331.4	1111.48949				ION=[M+H+H]2+		1314.77	0.00	297.17	3566.67
1079	331.65	782.45969				ION=[M+H+H]2+		277.23	2682.00	250.50	63.00
1080	331.83	1485.5227				ION=[M+H+H]2+		327.54	2585.67	0.00	0.00
1081	331.86	1691.89361				ION=[M+H+H2]3+		1368.92	488.33	2393.50	728.33
1082	331.99	1508.86919				ION=[M+H+H2]3+		811.23	1910.17	5151.17	692.00
1083	332.07	1123.60181				ION=[M+H+H]2+		1944.62	1926.33	898.33	1018.00
1084	332.26	564.29691				ION=[M+H]+		1426.46	660.50	2357.50	1048.00
1085	332.71	359.20546				ION=[M+H]+		2830.92	729.50	1666.67	2224.00
1086	332.99	2052.93966				ION=[M+H+H2]3+		1295.38	15.33	216.17	0.00
1087	334.11	401.2259				ION=[M+H]+		1537.69	645.50	402.17	2488.67
1088	334.65	668.23654				ION=[M+H]+		4657.69	22043.17	6261.00	3427.33
1089	335.22	343.24698				ION=[M+H]+		5244.15	988.50	1872.67	5467.00
1090	335.31	929.52611				ION=[M+H+H]2+		54.77	3368.00	563.00	42.00
1091	335.57	1140.55394				ION=[M+H+H]2+		1118.62	1058.33	715.83	260.67
1092	335.59	524.35308				ION=[M+H+H]2+		8021.54	0.00	0.00	0.00
1093	335.79	2123.97926				ION=[M+H+H2]3+		1727.85	0.00	953.00	30.00
1094	335.85	570.27584				ION=[M+H]+		388.31	92.50	0.00	0.00
1095	335.97	1590.32295				ION=[M+H+H]2+		0.00	2809.83	7.00	12.67
1096	336.02	1589.8208				ION=[M+H+H]2+		33.08	3015.00	27.50	0.00
1097	336.12	1589.31998				ION=[M+H+H]2+		0.00	2085.67	9.67	0.00
1098	336.71	661.26				ION=[M+H+H]2+		0.00	25.33	9.67	4226.00
1099	337.07	365.19558				ION=[M+H]+		3692.92	1958.00	1933.83	5027.33
1100	338.02	1615.86903				ION=[M+H+H2]3+		222.46	1373.33	70.50	147.33
1101	338.27	1211.58925				ION=[M+H+H]2+		4874.62	795.67	389.00	221.00
1102	338.68	431.27312				ION=[M+H]+		2470.31	1064.67	354.33	3375.67
1103	339.57	364.1993				ION=[M+H]+		693.85	2197.83	964.83	1510.33
1104	339.71	754.49049				ION=[M+H+H]2+		1179.38	1002.00	4574.00	1924.00
1105	339.79	155.09517	5.032	Arecoline	C8H13NO2	ION=[M+H]+		2462.00	5287.17	1204.83	7120.33
1106	340.03	846.40331				ION=[M+H+H]2+		0.00	0.00	445.50	346.67
1107	340.13	423.20057				ION=[M+H]+		3446.62	295.33	3573.67	1152.00
1108	340.54	950.50944				ION=[M+H+H]2+		392.46	1529.67	401.00	384.00
1109	340.54	333.133	18.371	Golotimod	C16H19N3O5	ION=[M+H]+	229305-39-9	4707.54	7088.00	13362.33	9351.33
1110	340.57	1854.99453				ION=[M+H+H2]3+		293.23	2432.83	2141.00	894.00
1111	340.6	312.14772	13.123	OSU-HDAC42	C18H20N2O3	ION=[M+H]+	935881-37-1	10301.08	1837.83	2778.67	8895.33
1112	341.24	1258.15031				ION=[M+H+H]2+		1264.77	0.00	292.50	0.00
1113	341.42	1433.23425				ION=[M+H+H]2+		218.92	1379.33	31.83	0.00
1114	341.72	377.23134				ION=[M+H]+		204.92	2280.83	65.50	557.67
1115	341.75	1215.66329				ION=[M+H+H]2+		520.15	2311.17	37.17	127.33
1116	341.77	601.19246				ION=[M+H]+		8277.69	623.83	412.50	1586.67

1117	341.85	651.0837				ION=[M+H]+		1141.08	21.00	105.67	0.00
1118	341.85	1301.66672				ION=[M+H+H]2+		1490.62	55.00	345.50	334.00
1119	342.13	805.83485				ION=[M+H]+		23.08	0.00	3834.17	130.33
1120	342.29	1346.67191				ION=[M+H+H]2+		14346.15	1149.50	650.83	707.67
1121	342.93	1096.07795				ION=[M+H+H]2+		1552.46	3501.17	2081.00	588.33
1122	343.05	386.21588				ION=[M+H]+		2985.38	533.17	1834.00	1901.33
1123	343.07	1096.5767				ION=[M+H+H]2+		1960.92	4170.83	2585.33	1037.67
1124	343.45	1442.81606				ION=[M+H+H2]3+		2423.38	290.33	402.33	254.33
1125	343.99	771.01455				ION=[M+H]+		0.00	0.00	3535.00	94.33
1126	344.32	2223.04627				ION=[M+H+H2]3+		14095.54	77.33	455.50	0.00
1127	344.4	771.41536				ION=[M+H]+		0.00	80.67	7372.00	334.00
1128	344.59	242.12675	18.417	2,4-Diamino-6-Phenyl-5,6,7,8,-Tetrahydropteridine	C12H14N6	ION=[M+H]+		3009.38	417.67	515.00	1866.00
1129	344.83	1231.64185				ION=[M+H+H]2+		10020.77	3479.83	571.83	170.00
1130	344.98	771.21503				ION=[M+H]+		17.38	81.33	6746.83	364.67
1131	345.33	1815.96947				ION=[M+H+H2]3+		1261.69	1381.50	150.83	272.67
1132	345.49	365.13783	14.666	3-{3-[(3,4-dihydroxybutoxy)amino]-1h-indol-2-yl}-2h-indol-2-one	C20H19N3O4	ION=[M+H]+		3581.69	10493.33	4092.83	1692.00
1133	345.86	1951.95445				ION=[M+H+H2]3+		999.08	1566.00	0.00	99.00
1134	346	1576.78665				ION=[M+H+H]2+		2403.69	2703.17	222.50	128.67
1135	346.29	1575.77749				ION=[M+H+H]2+		2426.62	3381.00	220.17	171.00
1136	346.3	407.20652				ION=[M+H]+		2272.46	397.33	1687.67	1522.67
1137	346.54	1632.80089				ION=[M+H+H]2+		3178.00	2188.83	1155.50	415.33
1138	346.96	226.97776				ION=[M+H]+		4010.15	3359.67	2769.33	2424.67
1139	347.18	1222.13349				ION=[M+H+H]2+		1497.08	29.67	0.00	0.00
1140	347.32	1222.62378				ION=[M+H+H]2+		2283.85	297.50	209.83	299.00
1141	349.34	1234.69609				ION=[M+H+H2]3+		1135.23	17.33	23.00	0.00
1142	350.85	1670.92472				ION=[M+H+H2]3+		0.00	1292.17	51.67	25.67
1143	351.09	555.1865				ION=[M+H]+		8842.46	16502.33	21624.83	11009.67
1144	351.22	1012.54387				ION=[M+H+H]2+		4860.77	280.00	863.83	689.00
1145	351.22	1013.04704				ION=[M+H+H]2+		5050.00	137.33	805.67	178.67
1146	351.22	1442.79231				ION=[M+H+H]2+		5140.46	15.83	94.33	80.67
1147	351.32	239.05028				ION=[M+H]+		145.69	1151.17	273.00	4054.33
1148	351.36	2083.10381				ION=[M+H+H2]3+		226.62	1344.50	922.50	0.00
1149	351.81	1270.74534				ION=[M+H+H]2+		4041.69	0.00	90.83	175.00
1150	351.97	290.12966	7.242	N,N'-Bis(tert-butoxycarbonyl)-S-methylisothiourea	C12H22N2O4S	ION=[M+H]+	107819-90-9	815.54	616.17	10798.33	475.33
1151	352.01	475.29973				ION=[M+H]+		1292.31	573.50	234.67	1915.00
1152	352.04	2154.14729				ION=[M+H+H2]3+		209.85	519.67	2850.33	638.00
1153	352.27	117.07834	4.707	L(+)-norvaline | L-norvaline	C5H11NO2	ION=[M+H]+		333.38	117.17	4904.00	47.00
1154	352.47	1387.70919				ION=[M+H+H]2+		1468.00	31.50	97.00	74.00
1155	352.68	212.10673	12.883	(E)-2-(1,5-dihydroxypent-2-en-2-yl)-3-(hydroxymethyl)cyclopent-2-enone	C11H16O4	ION=[M+H]+		10121.85	11653.00	4569.67	3979.33
1156	352.74	723.15579				ION=[M+H+H]2+		1705.85	6201.00	6311.33	2512.67
1157	352.76	708.19112				ION=[M+H+H]2+		1764.62	7357.17	7126.83	199.33
1158	353.26	283.19955				ION=[M+H]+		6210.00	6509.50	10163.83	5421.33
1159	353.27	566.39937				ION=[M+H+H]2+		612.62	902.67	1573.50	0.00
1160	353.43	1251.73805				ION=[M+H+H]2+		0.00	8963.83	213.33	279.33
1161	353.65	361.57699				ION=[M+H]+		402.15	0.00	0.00	0.00
1162	353.78	1177.60923				ION=[M+H+H]2+		1479.54	192.83	112.67	229.33
1163	354.01	576.34365				ION=[M+H+H]2+		4541.54	0.00	88.67	113.33
1164	354.06	376.1384	24.407	N-methylwelwitindolinone D isonitrile	C22H20N2O4	ION=[M+H]+		2982.15	1408.33	2106.00	1509.33
1165	354.11	907.49501				ION=[M+H+H]2+		9430.62	3668.67	141.83	113.00
1166	355.31	1092.5374				ION=[M+H+H]2+		383.85	2426.33	252.00	122.00
1167	355.64	347.11225				ION=[M+H]+		2622.31	10332.50	2645.17	0.00
1168	355.84	1634.84802				ION=[M+H+H2]3+		17062.92	1479.17	473.50	112.67
1169	355.86	1493.26753				ION=[M+H+H]2+		996.15	32.17	0.00	0.00
1170	355.92	1493.76848				ION=[M+H+H]2+		2087.23	79.33	38.50	38.33
1171	355.92	1070.06849				ION=[M+H+H]2+		1874.00	52.33	34.00	270.67
1172	356.06	1494.27343				ION=[M+H+H]2+		1769.69	0.00	0.00	0.00
1173	356.06	746.63404				ION=[M+H]+		447.08	16.17	0.00	0.00
1174	356.23	692.23401				ION=[M+H+H]2+		0.00	0.00	964.67	0.00
1175	356.24	1069.56827				ION=[M+H+H]2+		1709.08	559.33	316.00	583.67
1176	356.95	2363.17998				ION=[M+H+H2]3+		456.46	4274.00	0.00	18.33
1177	357.84	211.12215				ION=[M+H]+		438.31	5193.33	31.67	114.00
1178	358.24	2310.12341				ION=[M+H+H2]3+		0.00	1342.67	78.83	0.00
1179	358.43	183.09087				ION=[M+H]+		2777.54	6216.33	127.17	2452.67
1180	358.53	434.25246	12.364	ribociclib succinate	C23H30N8O	ION=[M+H]+	1211441-98-3	133.54	40.17	160.67	9747.00
1181	358.56	972.50561				ION=[M+H+H]2+		1558.62	307.83	388.50	90.00
1182	359.08	1935.95888				ION=[M+H+H2]3+		12717.08	9693.33	635.67	742.67
1183	359.22	1820.91184				ION=[M+H+H2]3+		16646.92	75.33	236.67	674.33
1184	360.25	1305.72703				ION=[M+H+H2]3+		377.08	1556.83	43.50	781.00
1185	360.36	1408.73984				ION=[M+H+H]2+		4612.31	3623.83	783.33	1564.67
1186	360.52	1245.65836				ION=[M+H+H]2+		1592.00	270.33	205.67	228.67

1187	360.53	1392.7617				ION=[M+H+H2]3+		516.92	3952.00	311.33	376.67
1188	360.57	1416.74081				ION=[M+H+H]2+		3136.46	136.00	204.17	452.33
1189	360.94	198.98431				ION=[M+H]+		4934.31	4843.50	4588.50	3988.33
1190	361.51	645.81649				ION=[M+H]+		267.23	1400.50	137.67	0.00
1191	362.02	243.05859				ION=[M+H]+		0.00	2471.33	0.00	0.00
1192	362.02	1485.52454				ION=[M+H+H]2+		172.31	1892.33	0.00	0.00
1193	362.34	2434.22575				ION=[M+H+H2]3+		156.31	1700.17	159.33	210.33
1194	362.64	1089.09153				ION=[M+H+H]2+		3336.62	662.83	24.83	0.00
1195	362.84	1032.07847				ION=[M+H+H]2+		2717.38	129.83	47.00	0.00
1196	362.84	2221.07136				ION=[M+H+H2]3+		3224.00	55.00	382.17	66.00
1197	362.84	357.26341				ION=[M+H]+		3082.15	712.00	1043.33	3869.33
1198	363.1	1089.59121				ION=[M+H+H]2+		3810.62	787.17	118.17	0.00
1199	363.17	1093.59527				ION=[M+H+H]2+		16740.62	402.67	687.83	125.33
1200	363.44	1221.65494				ION=[M+H+H]2+		9126.77	213.33	2695.67	732.00
1201	363.5	1031.57902				ION=[M+H+H]2+		2282.15	162.50	42.17	0.00
1202	363.97	2276.1529				ION=[M+H+H2]3+		361.08	4351.17	0.00	107.67
1203	364.33	1621.96332	13.816	Ampullosporin	C77H127N19O19	ION=[M+H+H2]3+		26.77	3000.67	17.00	111.00
1204	364.46	1633.91514				ION=[M+H+H]2+		1922.46	0.00	0.00	0.00
1205	364.49	395.14822				ION=[M+H]+		37484.77	81424.83	10487.17	9248.00
1206	365.97	393.19014				ION=[M+H]+		3763.08	473.33	2310.83	2001.67
1207	366.44	1090.53061				ION=[M+H+H]2+		3011.08	605.83	346.33	208.67
1208	366.44	1128.4691				ION=[M+H+H2]3+		1331.85	410.50	22.50	110.33
1209	366.45	1170.39799				ION=[M+H]+		3976.92	732.17	446.67	953.67
1210	366.52	1756.60053				ION=[M+H+H]2+		3968.00	2074.00	754.33	1995.00
1211	366.56	638.10344				ION=[M+H+H]2+		3274.92	2554.83	1845.67	2974.33
1212	366.61	1755.58907				ION=[M+H+H]2+		6306.31	2322.33	808.83	2888.00
1213	366.66	585.69505				ION=[M+H]+		12655.85	6575.83	2833.83	6832.33
1214	366.77	1168.37428				ION=[M+H+H]2+		2730.00	2110.33	1490.00	3067.67
1215	366.84	1514.75024				ION=[M+H+H]2+		1136.31	103.50	373.00	180.33
1216	366.97	647.33267				ION=[M+H]+		452.00	1485.17	407.50	159.00
1217	367	371.16974				ION=[M+H]+		34.62	2091.67	42.83	184.67
1218	367.29	1294.16939				ION=[M+H+H]2+		0.00	1689.83	296.33	0.00
1219	367.42	371.89977				ION=[M+H+H]2+		4479.38	6554.00	8334.83	4220.67
1220	367.65	2270.12498				ION=[M+H+H2]3+		1266.31	0.00	10.83	0.00
1221	367.65	623.13974				ION=[M+H+H]2+		2040.77	1352.67	1620.33	768.00
1222	367.66	2250.12719				ION=[M+H+H2]3+		1062.92	210.50	374.17	0.00
1223	367.87	831.20134				ION=[M+H]+		0.00	130.67	2185.50	17.67
1224	368.48	1723.00799				ION=[M+H+H2]3+		0.00	2784.67	40.50	0.00
1225	368.54	831.40341				ION=[M+H]+		152.15	354.33	2595.50	37.00
1226	368.57	1550.81791				ION=[M+H+H]2+		7715.23	113.33	390.83	39.67
1227	369.09	274.13131	12.873	Mablin	C10H18N4O5	ION=[M+H]+		1579.38	1938.17	1192.67	1975.33
1228	369.64	1257.65117				ION=[M+H+H]2+		353.38	1331.83	371.33	279.00
1229	369.83	612.23947	38.665	Glycosylated Palythine-threonine	C24H40N2O16	ION=[M+H+H]2+		813.85	421.83	4730.17	168.00
1230	369.89	442.31426				ION=[M+H]+		1509.23	714.33	411.83	4456.67
1231	369.98	574.29708				ION=[M+H]+		1107.54	207.33	5469.67	1433.33
1232	370.16	279.18368	15.766	Tramadol N-Oxide	C16H25NO3	ION=[M+H]+		0.00	3445.67	0.00	0.00
1233	370.71	1315.70239				ION=[M+H+H]2+		386.92	1806.50	168.83	168.33
1234	370.77	1852.05906				ION=[M+H+H2]3+		1560.15	296.17	162.83	0.00
1235	371.78	1007.54337	7.57	Mulundocandin	C48H77N7O16	ION=[M+H+H]2+		2364.46	148.00	49.00	293.00
1236	371.79	2064.07431				ION=[M+H+H2]3+		1017.69	2336.83	1893.83	32.33
1237	371.99	1990.94022				ION=[M+H+H2]3+		0.00	148.00	1891.17	0.00
1238	372.37	718.27984				ION=[M+H+H]2+		294.46	2081.00	364.83	0.00
1239	372.49	680.33337				ION=[M+H+H]2+		454.46	2453.50	469.50	0.00
1240	373.15	689.21669				ION=[M+H+H]2+		244.92	1724.83	316.17	35.33
1241	373.28	720.17757				ION=[M+H]+		56.31	24.17	3109.00	0.00
1242	373.37	1795.95008				ION=[M+H+H]2+		74.62	0.00	425.17	0.00
1243	373.4	1683.89052				ION=[M+H+H2]3+		3668.15	1447.00	292.17	570.67
1244	373.4	1407.72143				ION=[M+H+H]2+		8033.23	368.50	607.83	596.00
1245	373.4	1754.93084				ION=[M+H+H2]3+		1600.00	117.67	109.17	92.67
1246	373.4	1216.66049				ION=[M+H+H]2+		1879.08	94.33	0.00	369.67
1247	373.59	719.57792				ION=[M+H]+		14.15	11.17	2203.17	0.00
1248	373.65	1234.67586				ION=[M+H+H]2+		1388.92	3030.00	49.83	28.67
1249	373.7	892.51119	9.549			ION=[M+H+H]2+		2463.69	24.83	0.00	479.33
1250	373.91	1356.65098				ION=[M+H+H]2+		75.85	3494.67	0.00	0.00
1251	374.07	1799.95375				ION=[M+H+H]2+		0.00	0.00	2310.67	0.00
1252	374.82	1292.68989				ION=[M+H+H]2+		4861.08	244.67	1118.17	399.67
1253	375.32	373.22147				ION=[M+H]+		3413.08	480.33	3508.00	2562.33
1254	375.51	646.34892				ION=[M+H]+		1851.08	54.67	83.33	403.33
1255	375.65	819.87544				ION=[M+H]+		689.54	0.00	21.00	0.00
1256	375.68	1639.76227				ION=[M+H+H]2+		1250.15	0.00	0.00	0.00
1257	376.17	1609.30174				ION=[M+H+H]2+		20.15	1312.50	118.17	0.00
1258	376.21	351.16017	13.991	Adipiplon	C18H18FN7	ION=[M+H]+	840486-93-3	3215.54	750.33	973.00	2926.33
1259	376.33	1610.30971				ION=[M+H+H]2+		52.46	2385.00	190.33	0.00
1260	376.58	1609.80692				ION=[M+H+H]2+		60.92	2410.00	393.83	40.33

1261	376.86	1417.55866				ION=[M+H+H2]3+		1712.31	116.33	9.67	0.00
1262	376.86	1379.62207				ION=[M+H+H]2+		6027.08	634.83	0.00	0.00
1263	377.26	1473.7339				ION=[M+H+H]2+		1449.38	1426.83	1389.00	50.00
1264	377.41	1676.82416				ION=[M+H+H2]3+		199.85	1312.67	33.00	0.00
1265	377.47	1401.60803				ION=[M+H+H]2+		1101.69	0.00	0.00	0.00
1266	377.54	1797.937				ION=[M+H+H2]3+		1339.69	793.50	301.50	320.00
1267	377.83	739.37664	0.854	4'-O-Œ≤-D-Glucose glucopiericidin A	C37H57NO14	ION=[M+H]+		1072.77	124.83	776.83	433.00
1268	377.83	1472.62808				ION=[M+H+H2]3+		429.85	26.67	15.17	0.00
1269	378.49	2931.48986				ION=[M+H+H2]3+		18.46	603.67	1355.83	0.00
1270	378.5	738.19902				ION=[M+H+H]2+		2557.23	2590.00	844.50	42.67
1271	379.26	1478.75304				ION=[M+H+H]2+		448.92	62.17	35.17	0.00
1272	379.93	654.25604				ION=[M+H]+		22794.77	20586.00	15431.17	5066.67
1273	380.03	539.22375				ION=[M+H+H]2+		1653.69	330.67	6281.00	249.00
1274	381.24	683.01383				ION=[M+H]+		746.62	430.33	0.00	40.00
1275	381.32	683.34402				ION=[M+H]+		1562.62	411.50	191.67	0.00
1276	381.82	1337.68641				ION=[M+H+H]2+		254.31	1420.17	249.33	32.67
1277	381.95	1780.83822				ION=[M+H+H2]3+		1193.69	33.83	0.00	1695.00
1278	381.95	1780.85801				ION=[M+H+H]2+		906.15	41.00	159.50	1553.33
1279	382.47	238.12158	12.218	7-(3-butanonyl)-6,6-dimethyl-2-cyclohex-4-none-2-carboxylic acid	C13H18O4	ION=[M+H]+		2170.15	203.50	200.50	363.33
1280	382.57	736.86726				ION=[M+H]+		17.23	439.00	354.50	0.00
1281	382.66	923.59689				ION=[M+H+H]2+		17.85	1657.83	6.00	0.00
1282	382.84	729.624				ION=[M+H]+		1129.08	585.00	0.00	40.00
1283	383.1	746.38556				ION=[M+H]+		1138.15	156.83	5805.17	53.00
1284	383.16	746.18599				ION=[M+H]+		1214.31	189.50	6907.00	142.67
1285	383.19	499.33942				ION=[M+H]+		2123.69	451.33	497.83	1525.67
1286	383.31	745.98748				ION=[M+H]+		1024.77	161.83	6371.33	0.00
1287	383.31	745.78781				ION=[M+H]+		595.69	75.83	3517.00	0.00
1288	383.82	1515.79073				ION=[M+H+H]2+		3133.85	815.83	24.17	27.67
1289	383.82	1458.739				ION=[M+H+H]2+		1383.85	812.83	211.17	111.67
1290	384.43	978.49885				ION=[M+H+H]2+		1463.85	391.17	112.17	924.33
1291	384.68	1516.29591				ION=[M+H+H]2+		4306.31	1290.83	61.17	0.00
1292	384.8	1516.7954				ION=[M+H+H]2+		3684.31	1384.00	312.67	236.67
1293	385.21	746.58738				ION=[M+H]+		640.15	25.50	3282.33	20.67
1294	385.56	1685.75294				ION=[M+H+H]2+		0.00	2017.33	41.17	0.00
1295	385.56	1716.67239				ION=[M+H+H2]3+		0.00	1952.67	0.00	0.00
1296	385.83	1663.7716				ION=[M+H+H]2+		0.00	9236.83	325.83	0.00
1297	386.21	1701.70517				ION=[M+H+H2]3+		0.00	3897.50	64.67	0.00
1298	386.37	593.308				ION=[M+H]+		2859.54	2696.17	385.17	1320.33
1299	386.39	731.77875				ION=[M+H]+		2442.00	202.83	703.00	0.00
1300	386.39	1255.62966				ION=[M+H+H]2+		1313.38	13.67	147.83	33.33
1301	386.5	494.2172				ION=[M+H]+		3799.23	926.33	1193.67	1072.00
1302	386.77	425.22554				ION=[M+H]+		908.00	2184.67	2599.67	2534.00
1303	387.13	1306.70769				ION=[M+H+H]2+		3118.92	27.33	370.00	53.33
1304	387.17	1967.98207				ION=[M+H+H2]3+		63.69	10052.17	215.83	81.67
1305	388.16	377.25685				ION=[M+H]+		0.00	2078.83	45.83	0.00
1306	388.2	732.37857				ION=[M+H]+		1649.08	142.17	565.83	158.33
1307	389.12	218.0713				ION=[M+H]+		2383.54	2710.33	860.33	965.33
1308	389.29	1227.57394				ION=[M+H+H]2+		1017.23	0.00	16.50	0.00
1309	389.5	1332.67764				ION=[M+H+H]2+		231.69	347.83	4398.83	234.00
1310	389.8	1066.40446				ION=[M+H+H2]3+		1090.15	72.17	9.17	27.33
1311	389.83	1666.35062				ION=[M+H+H]2+		440.31	2301.17	93.67	0.00
1312	390.04	1028.46063				ION=[M+H+H]2+		3900.31	242.50	0.00	0.00
1313	390.35	1666.85033				ION=[M+H+H]2+		406.46	2100.00	389.83	94.67
1314	390.43	1028.47751				ION=[M+H]+		1193.85	0.00	17.83	16.33
1315	390.62	166.07695	6.544	Diisopropylphosphono group	C6H15O3P	ION=[M+H]+	1809-20-7	3324.77	1449.00	1603.33	4560.00
1316	390.73	3062.53164				ION=[M+H+H2]3+		3427.85	4339.50	2293.00	38.67
1317	391.02	1663.86972				ION=[M+H+H]2+		866.92	15.83	0.00	0.00
1318	391.05	358.16297	17.512	Aurantiomide B	C18H22N4O4	ION=[M+H]+		917.08	1558.83	2445.00	4592.67
1319	391.17	389.09132				ION=[M+H]+		558.00	1149.33	1466.50	4236.33
1320	391.29	2417.19456				ION=[M+H+H2]3+		1635.85	32.50	0.00	0.00
1321	391.42	358.18436				ION=[M+H]+		0.00	119.00	0.00	8241.67
1322	391.44	1664.36814				ION=[M+H+H]2+		1121.23	0.00	30.17	0.00
1323	391.45	353.20521				ION=[M+H]+		1079.38	2049.17	2704.67	5668.00
1324	391.59	2593.29761				ION=[M+H+H2]3+		100.00	4671.67	200.33	153.00
1325	392.24	1154.66265				ION=[M+H+H]2+		1003.54	109.00	211.50	5089.67
1326	392.27	414.11843				ION=[M+H]+		1623.08	0.00	1939.00	0.00
1327	392.32	1664.86698				ION=[M+H+H]2+		1269.85	182.83	242.67	64.33
1328	392.83	2826.34418				ION=[M+H+H2]3+		0.00	1792.83	192.33	0.00
1329	394.74	737.17102				ION=[M+H+H]2+		26974.77	18623.00	24479.50	19186.00
1330	394.74	341.62653				ION=[M+H]+		0.00	0.00	430.17	4168.33
1331	394.75	1368.53433				ION=[M+H]+		8124.46	872.00	4181.33	4055.67
1332	394.8	684.25771				ION=[M+H+H]2+		8844.15	5239.83	8431.50	7485.67
1333	394.93	1421.43773				ION=[M+H+H]2+		13857.54	7300.33	13763.83	9634.33

1334	395.11	722.20691				ION=[M+H+H]2+		20596.00	19672.83	27094.83	1862.67
1335	395.13	1406.47293				ION=[M+H+H]2+		4324.15	3842.50	6839.50	206.33
1336	395.14	1369.03423				ION=[M+H]+		3407.54	580.33	1059.67	1269.33
1337	395.14	728.22575				ION=[M+H+H]2+		1827.38	1735.33	2146.67	326.00
1338	395.14	1053.35887				ION=[M+H]+		1270.31	250.00	1792.50	1463.67
1339	395.15	683.25238				ION=[M+H+H]2+		12041.38	6651.00	10620.00	5069.00
1340	395.44	1407.4778				ION=[M+H+H]2+		3336.92	2781.67	5039.83	200.00
1341	396.45	1498.78871				ION=[M+H+H]2+		2414.62	102.17	0.00	173.00
1342	397.1	335.15865	14.194	Riboprine	C15H21N5O4	ION=[M+H]+	7724-76-7	2557.08	496.17	336.67	1203.67
1343	398.76	1715.88873				ION=[M+H+H]2+		1488.15	100.33	599.67	0.00
1344	399.34	1500.78066				ION=[M+H+H2]3+		202.00	60.17	4311.83	57.33
1345	401.02	1595.84247				ION=[M+H+H]2+		7194.62	197.17	602.67	0.00
1346	402.5	622.26873				ION=[M+H]+		26.15	3743.17	0.00	0.00
1347	402.56	303.11152	5.384	8-[(2,5-dimethoxyphenyl)methyl]-2-fluoro-9h-purin-6-amine	C14H14FN5O2	ION=[M+H]+		0.00	44503.67	65.67	0.00
1348	402.77	320.13736	8.045	Z-ala-pro-oh	C16H20N2O5	ION=[M+H]+	21027-01-0	143.23	22689.00	49.00	76.33
1349	404.12	939.79552				ION=[M+H]+		80.77	1234.83	167.67	47.67
1350	404.59	235.10721	8.807	1-[(2-amino-9h-purin-6-yl)oxy]-3-methylbutan-2-one	C10H13N5O2	ION=[M+H]+		1848.77	528.83	456.50	3749.67
1351	404.71	1339.61652				ION=[M+H+H]2+		7625.54	0.00	11.00	0.00
1352	405.21	158.05861	13.682	Gabosine H	C7H10O4	ION=[M+H]+		1662.00	682.67	565.83	3261.00
1353	405.71	117.07812	2.101	L(+)-norvaline | L-norvaline	C5H11NO2	ION=[M+H]+		720.92	255.50	22323.83	309.67
1354	405.76	566.32908				ION=[M+H]+		0.00	0.00	1925.33	0.00
1355	406.16	330.17881	17.217	L-n(omega)-nitroarginine-(4r)-amino-l-proline amide	C11H22N8O4	ION=[M+H]+		770.15	41.50	11487.17	225.67
1356	406.19	1595.33224				ION=[M+H+H]2+		1166.00	13.83	0.00	56.67
1357	406.39	393.25151				ION=[M+H]+		176.77	3238.17	7.33	46.33
1358	406.59	1663.81445				ION=[M+H+H]2+		1208.92	194.17	0.00	49.67
1359	406.86	1298.62845				ION=[M+H+H]2+		163.69	3271.50	120.50	50.33
1360	407.03	1141.5599				ION=[M+H+H]2+		2438.92	479.50	410.83	0.00
1361	407.43	2081.06951				ION=[M+H+H2]3+		1017.38	96.17	62.00	33.00
1362	407.81	1806.26765				ION=[M+H+H]2+		0.00	24.17	1076.50	55.67
1363	408.32	693.68912				ION=[M+H]+		429.85	40.00	0.00	21.33
1364	408.93	1516.77047				ION=[M+H+H]2+		333.23	316.83	451.50	4000.00
1365	409.31	2803.39167				ION=[M+H+H2]3+		221.85	1382.50	84.00	33.00
1366	411.01	180.09116				ION=[M+H]+		747.69	2312.17	544.67	750.33
1367	413.31	306.12334				ION=[M+H]+		145.85	1957.33	194.33	102.67
1368	413.5	248.11662				ION=[M+H]+		2827.85	1594.50	1435.33	1664.33
1369	413.65	499.196				ION=[M+H]+		193.23	2962.17	136.33	38.67
1370	414.33	1135.57055				ION=[M+H+H]2+		1610.92	52.00	0.00	59.00
1371	414.36	1320.16179				ION=[M+H+H]2+		66.77	2193.67	82.67	24.33
1372	414.37	2639.33413				ION=[M+H+H2]3+		148.62	8062.83	331.83	30.00
1373	414.48	1319.65985				ION=[M+H+H]2+		25.38	1609.50	0.00	29.67
1374	414.56	507.1654				ION=[M+H]+		2320.46	1300.00	643.33	2927.33
1375	414.69	301.01388				ION=[M+H]+		2200.31	2168.50	1536.00	3226.33
1376	415.63	2617.36138				ION=[M+H+H2]3+		1245.54	257.50	58.83	0.00
1377	417.7	168.11578	6.089	2-(2-hydroxy-cyclopentyl)-pent-4-el	C10H16O2	ION=[M+H]+		2164.77	840.00	1666.83	12429.00
1378	418.18	1214.68045				ION=[M+H+H]2+		1897.38	72.17	81.17	4035.00
1379	418.58	726.35427				ION=[M+H]+		1417.69	230.50	254.33	382.00
1380	419.83	1398.51263				ION=[M+H]+		22.31	1149.33	0.00	0.00
1381	420.03	1553.59874				ION=[M+H+H]2+		12.31	25.00	2700.50	81.00
1382	420.03	776.89959				ION=[M+H]+		59.23	168.00	2859.00	46.00
1383	420.18	874.46714				ION=[M+H+H]2+		0.00	22.33	14109.33	6748.67
1384	420.52	1554.00222				ION=[M+H+H]2+		102.15	0.00	2561.00	0.00
1385	421.21	254.99565				ION=[M+H]+		1315.69	611.00	625.00	2259.00
1386	421.28	1399.51113				ION=[M+H]+		11.69	1485.33	0.00	0.00
1387	421.53	1399.50013				ION=[M+H]+		0.00	1239.00	0.00	0.00
1388	421.82	863.44375				ION=[M+H]+		279.38	35.17	2024.67	89.67
1389	421.82	863.33437				ION=[M+H]+		0.00	0.00	2821.00	41.00
1390	422.47	174.09024	6.168	Suberic acid	C8H14O4	ION=[M+H]+		92.62	5277.33	0.00	0.00
1391	422.68	274.09665	13.984	4-{[(5-oxo-2-phenyl-4,5-dihydro-1,3-oxazol-4-ylidene)methyl]amino}butanoic acid	C14H14N2O4	ION=[M+H]+		3254.92	1941.83	2021.83	3032.00
1392	423.17	1321.64113				ION=[M+H+H]2+		1679.54	199.50	76.50	24.67
1393	423.47	1398.48921				ION=[M+H+H]2+		98.92	5453.50	0.00	40.33
1394	423.58	1451.4018				ION=[M+H+H]2+		46.15	2745.17	0.00	0.00
1395	424.14	1224.41291				ION=[M+H]+		43.54	2574.33	0.00	0.00
1396	424.15	265.10039				ION=[M+H]+		546.46	4792.17	76.33	879.00
1397	424.3	1394.67795				ION=[M+H+H]2+		1296.46	197.50	95.17	67.67
1398	424.34	947.37079				ION=[M+H+H]2+		4482.15	3183.67	0.00	33.67
1399	424.62	544.20307				ION=[M+H]+		0.00	1266.83	0.00	0.00
1400	424.62	142.06347	5.102	Glycidyl methacrylate	C7H10O3	ION=[M+H]+	106-91-2	77.23	1246.50	104.17	97.00
1401	424.89	854.2987				ION=[M+H]+		0.00	1518.33	179.67	27.67
1402	426.77	1630.87636				ION=[M+H+H]2+		30.62	1463.33	79.17	336.33
1403	427.45	281.09522				ION=[M+H]+		3694.31	2988.50	1205.33	9652.33

1404	428.23	963.36596				ION=[M+H+H]2+		25382.00	17522.17	25.17	21.33
1405	428.27	468.27036				ION=[M+H]+		3507.08	1777.00	1318.33	468.33
1406	428.28	1532.74416				ION=[M+H+H]2+		391.08	1802.67	434.00	39.67
1407	428.82	537.28358				ION=[M+H+H]2+		7140.77	1158.33	4687.17	7108.00
1408	428.87	3096.56099				ION=[M+H+H2]3+		1675.08	693.33	65.83	0.00
1409	428.94	1532.23838				ION=[M+H+H]2+		345.23	1863.17	390.00	0.00
1410	428.99	443.25331				ION=[M+H]+		0.00	2400.83	0.00	0.00
1411	429.38	196.11193				ION=[M+H]+		6503.08	5507.00	1972.83	2067.67
1412	429.41	1398.5036				ION=[M+H]+		14.46	1539.00	0.00	0.00
1413	429.43	1548.77064				ION=[M+H+H]2+		1144.46	780.00	354.33	52.67
1414	429.85	519.27377				ION=[M+H+H]2+		2311.69	480.50	1872.67	2618.67
1415	429.96	274.19371	14.678	Empenthrin	C18H26O2	ION=[M+H]+	54406-48-3	2899.69	460.67	2128.33	3236.00
1416	430.13	555.29857				ION=[M+H]+		2794.77	517.17	1700.83	3011.00
1417	430.75	1469.23568				ION=[M+H+H]2+		2347.69	262.17	30.17	25.67
1418	431.09	306.1587	19.009	Ruxolitinib	C17H18N6	ION=[M+H]+		48.77	5.67	2088.17	45.00
1419	431.1	250.09668				ION=[M+H]+		1788.62	209.33	750.67	2354.67
1420	431.51	700.26265				ION=[M+H]+		8116.46	2656.00	3710.50	5696.00
1421	431.93	1469.73707				ION=[M+H+H]2+		3528.31	475.50	401.17	64.00
1422	432.36	2938.48852				ION=[M+H+H2]3+		8085.38	567.67	474.67	0.00
1423	432.4	701.26401				ION=[M+H]+		3458.92	941.50	1641.00	2304.33
1424	432.67	1470.23589				ION=[M+H+H]2+		2985.23	303.83	227.83	0.00
1425	433	181.07603				ION=[M+H]+		1681.08	1809.50	408.00	638.67
1426	433.31	1242.6102				ION=[M+H+H]2+		4964.92	899.33	318.33	0.00
1427	433.5	182.07363	8.364	3-Hydroxy-5-(hydroxymethyl)-2-methylisonicotinaldehyde oxime	C8H10N2O3	ION=[M+H]+		3915.85	906.33	1817.33	5097.33
1428	433.58	1484.78619				ION=[M+H+H2]3+		0.00	0.00	5041.83	0.00
1429	434.04	804.41511				ION=[M+H]+		1708.15	301.00	607.67	0.00
1430	434.2	224.14103	10.917	12?-Deoxy-deoxysaxitoxin	C9H16N6O	ION=[M+H]+		4364.00	3768.50	8234.67	5399.00
1431	435.02	604.25491				ION=[M+H+H]2+		1775.38	42971.83	2144.00	1010.00
1432	435.3	419.05625				ION=[M+H]+		98.62	2813.83	157.00	0.00
1433	436.46	303.2048				ION=[M+H]+		0.00	0.00	58.33	8169.67
1434	436.77	233.14287	9.187	Methylphenidate	C14H19NO2	ION=[M+H]+		100.62	1876.67	90.17	266.00
1435	437.18	882.44404				ION=[M+H+H]2+		0.00	27.33	5378.00	0.00
1436	438.94	795.41283				ION=[M+H+H]2+		10.92	0.00	4071.00	0.00
1437	439.31	268.64208				ION=[M+H]+		5151.38	883.83	3236.83	4279.00
1438	440.32	375.12607				ION=[M+H]+		36.15	3118.83	15.00	0.00
1439	440.94	1769.31205				ION=[M+H+H]2+		1550.00	115.50	720.67	0.00
1440	442.33	2952.49675				ION=[M+H+H2]3+		945.08	0.00	113.50	0.00
1441	443.37	1356.65285				ION=[M+H+H]2+		1690.77	218.00	134.17	75.00
1442	443.49	2460.19363				ION=[M+H+H2]3+		1758.00	85.50	31.67	0.00
1443	443.72	392.18134				ION=[M+H]+		696.46	834.67	2037.83	239.33
1444	445.48	1406.61601				ION=[M+H+H]2+		1751.38	345.50	391.50	40.00
1445	446.08	1427.68956				ION=[M+H+H]2+		3794.46	263.50	29.83	0.00
1446	446.52	539.29846				ION=[M+H+H]2+		2645.38	400.33	2327.67	2685.67
1447	446.67	2689.43039				ION=[M+H+H2]3+		1452.00	801.50	0.00	57.00
1448	449.53	194.09331	10.164	Nakienone A	C11H14O3	ION=[M+H]+		2113.38	1401.00	1955.33	830.67
1449	450.09	2852.50713				ION=[M+H+H2]3+		808.31	105.17	106.33	0.00
1450	450.41	553.27895				ION=[M+H+H]2+		3056.31	416.83	2273.50	3576.00
1451	450.54	693.18825				ION=[M+H]+		0.00	3671.50	0.00	0.00
1452	450.75	615.17228				ION=[M+H]+		12122.62	2226.83	1183.33	5185.00
1453	451.33	182.13157	6.304	(2Z,4E)-3-methyl-2,4-decadienoic acid	C11H18O2	ION=[M+H]+		329.54	5102.83	0.00	0.00
1454	451.73	2892.34247				ION=[M+H+H2]3+		1522.92	1876.00	903.33	0.00
1455	451.79	964.44941				ION=[M+H]+		1692.92	2147.83	1249.67	0.00
1456	451.79	964.78464				ION=[M+H]+		1430.62	1903.00	956.83	20.00
1457	452.39	606.23446				ION=[M+H]+		9674.15	3286.83	4609.00	8475.00
1458	453.14	1214.58728				ION=[M+H]+		566.92	0.00	0.00	20.67
1459	454.06	359.00899				ION=[M+H]+		1581.85	2465.33	10.67	47.00
1460	455.01	242.08113	0.977	S-8510	C12H10N4O2	ION=[M+H]+		47328.77	73176.00	1479.50	3156.33
1461	455.88	182.09567	8.623	Mephenesin	C10H14O3	ION=[M+H]+	59-47-2	504.77	328.00	2668.33	6032.33
1462	456.02	279.07954				ION=[M+H]+		7417.23	12208.00	998.83	7769.67
1463	456.12	1556.73431				ION=[M+H+H]2+		2997.85	481.83	0.00	0.00
1464	457.26	1698.29397				ION=[M+H+H]2+		1137.23	54.33	82.83	0.00
1465	458.69	263.22533				ION=[M+H]+		5287.85	4736.67	4745.50	9171.33
1466	461.48	1940.91742				ION=[M+H+H]2+		2236.31	19.00	47.67	0.00
1467	462.14	1812.85632				ION=[M+H+H]2+		2270.31	1266.00	528.33	62.33
1468	463.76	805.3338				ION=[M+H]+		6754.31	5156.83	536.33	7292.33
1469	464.21	231.12704	7.265	Fenfluramine	C12H16F3N	ION=[M+H]+	458-24-2	421.69	5774.00	496.83	221.00
1470	466.77	265.1003	15.933	Dipraglurant	C16H12FN3	ION=[M+H]+	872363-17-2	9444.77	5323.33	17.00	2943.00
1471	467.43	209.10699	11.072	11-Carboxyanatoxin-a	C11H15NO3	ION=[M+H]+		1121.54	3773.17	253.50	333.00
1472	468.08	276.14654				ION=[M+H]+		224.46	3761.00	362.50	242.67
1473	468.5	238.15741	17.728	Saccharomonopyrone B	C14H22O3	ION=[M+H]+		469.85	2021.83	321.33	107.33
1474	470.39	721.2731				ION=[M+H+H]2+		8.62	2318.50	15.83	0.00
1475	471.7	196.11206				ION=[M+H]+		2697.85	6186.50	987.17	2280.00
1476	472.45	2125.25445				ION=[M+H+H2]3+		514.15	985.83	1875.17	339.67

1477	478.9	443.25316				ION=[M+H]+		16.15	1759.83	0.00	49.00
1478	478.9	548.26299				ION=[M+H+H]2+		0.00	0.00	417.83	0.00
1479	479.05	1621.29759				ION=[M+H+H]2+		63.08	2106.67	31.17	0.00
1480	479.13	1621.79732				ION=[M+H+H]2+		117.69	3134.50	176.50	206.67
1481	482.1	377.25652	13.496	3-((3E,6E,8E,10R,11R,12E)-11-hydroxy-4,8,10,12-tetramethyltetradeca-3,6,8,12-tetraenamido)butanoic acid	C22H35NO4	ION=[M+H]+		6563.23	2605.50	1615.83	3975.00
1482	482.25	896.38786				ION=[M+H]+		2044.92	162.17	0.00	0.00
1483	482.25	896.04997				ION=[M+H]+		869.08	0.00	0.00	0.00
1484	482.25	2704.12074				ION=[M+H+H2]3+		1548.92	82.83	0.00	0.00
1485	482.68	1325.61684				ION=[M+H]+		2169.85	213.50	0.00	0.00
1486	482.84	2688.15087				ION=[M+H+H2]3+		1680.92	63.83	0.00	0.00
1487	482.99	1326.61092				ION=[M+H]+		1544.92	77.33	0.00	0.00
1488	482.99	1326.11507				ION=[M+H]+		2779.23	149.83	0.00	51.33
1489	483.67	1398.49148				ION=[M+H+H]2+		192.46	4386.67	20.83	23.00
1490	484.46	1615.21753				ION=[M+H+H]2+		68.15	83.00	452.67	0.00
1491	484.61	1224.41418				ION=[M+H]+		0.00	1688.83	0.00	0.00
1492	484.7	174.09048	7.238	Suberic acid	C8H14O4	ION=[M+H]+		149.23	4144.50	18.00	0.00
1493	485.09	934.01119				ION=[M+H]+		373.08	773.67	4258.50	438.00
1494	485.54	867.43697				ION=[M+H]+		290.15	911.33	2925.17	133.33
1495	486.05	1451.40386				ION=[M+H+H]2+		97.85	2369.67	46.50	0.00
1496	486.62	933.85723				ION=[M+H]+		126.15	531.17	2886.17	82.00
1497	487.64	867.5809				ION=[M+H]+		249.69	748.33	2677.00	101.67
1498	487.71	1731.0209				ION=[M+H+H]2+		0.00	24.00	1014.67	34.00
1499	488.8	809.54428				ION=[M+H]+		140.77	639.33	2462.67	0.00
1500	491.2	483.20021				ION=[M+H]+		28.62	4281.17	12.17	19.33
1501	492.45	181.07545	9.936	Tyrosin	C9H11NO3	ION=[M+H]+		1062.31	3089.00	120.33	1797.33
1502	496.4	867.36668				ION=[M+H]+		362.46	885.17	3812.83	558.00
1503	496.66	858.47207	19.035	Avermectin B1b	C47H70O14	ION=[M+H+H]2+	65195-56-4	37.08	0.00	7690.67	1836.00
1504	497	858.48317				ION=[M+H]+		11.38	0.00	3520.33	597.67
1505	497.43	934.16373				ION=[M+H]+		490.00	1133.17	4431.17	620.00
1506	498.5	1202.62679				ION=[M+H+H]2+		17.85	2046.83	0.00	0.00
1507	498.6	1730.85221				ION=[M+H+H]2+		0.00	142.50	634.33	0.00
1508	501.13	317.13914				ION=[M+H]+		57.69	2473.17	76.33	30.00
1509	501.19	1240.721				ION=[M+H+H]2+		0.00	10687.50	207.67	1791.67
1510	502.42	1238.70566				ION=[M+H+H]2+		24.00	8637.17	142.83	2311.67
1511	504	1216.64214				ION=[M+H+H]2+		0.00	4340.17	67.67	47.33
1512	504.44	1187.592				ION=[M+H+H]2+		0.00	9353.83	0.00	0.00
1513	505.09	1306.6333				ION=[M+H+H2]3+		6.62	373.67	0.00	967.67
1514	505.63	1252.72146				ION=[M+H+H]2+		10.77	19027.67	1649.50	45849.33
1515	505.64	435.54324				ION=[M+H]+		6.62	1747.33	215.17	3327.33
1516	505.77	512.24346				ION=[M+H+H]2+		0.00	3133.67	1057.50	0.00
1517	506.3	310.1421	19.554	1,3,4-trihydroxy-5-(3-phenoxypropyl)cyclohexane-1-carboxylic acid	C16H22O6	ION=[M+H]+		412.31	516.17	2114.17	1371.00
1518	506.58	1254.73741				ION=[M+H+H]2+		16.15	24870.83	3670.00	42160.00
1519	506.65	1250.60342				ION=[M+H+H]2+		0.00	2667.50	82.83	62.33
1520	507.77	261.06884				ION=[M+H]+		2592.15	5679.33	629.83	6953.00
1521	508.44	1307.63507				ION=[M+H+H2]3+		12.77	2470.00	302.17	2935.00
1522	508.44	1292.66856				ION=[M+H+H2]3+		0.00	3748.83	676.33	291.33
1523	508.44	1276.71746				ION=[M+H+H]2+		24.46	2340.67	406.50	784.00
1524	508.44	1308.64491				ION=[M+H+H2]3+		0.00	2598.67	472.83	3929.00
1525	508.44	1290.66422				ION=[M+H+H2]3+		290.46	3265.00	474.50	409.33
1526	509.1	164.12065				ION=[M+H]+		2957.38	2707.50	261.67	268.67
1527	510.41	1274.70038				ION=[M+H+H]2+		0.00	2178.17	226.50	1163.33
1528	511.72	1268.75133				ION=[M+H+H]2+		32.15	9257.00	1510.67	12765.00
1529	511.91	1266.73866				ION=[M+H+H]2+		14.92	7876.33	639.00	16459.67
1530	512.13	351.93043				ION=[M+H+H]2+		7601.23	5667.33	5070.33	5930.00
1531	514.04	632.28511				ION=[M+H+H]2+		0.00	0.00	0.00	918.67
1532	516.62	400.20804				ION=[M+H]+		725.38	1750.50	1070.83	1557.33
1533	517.42	1238.74068				ION=[M+H+H]2+		18.77	5123.17	156.83	447.00
1534	518.06	1236.72602				ION=[M+H+H]2+		15.38	3104.67	45.50	834.67
1535	518.9	410.11174				ION=[M+H]+		1655.85	447.50	364.83	829.67
1536	519.51	2260.24834				ION=[M+H+H2]3+		126.15	1533.83	70.00	0.00
1537	520.03	395.25177				ION=[M+H]+		742.46	2269.33	999.83	1641.67
1538	522.08	1252.75829				ION=[M+H+H]2+		28.62	9944.83	1610.83	14928.00
1539	522.44	1135.68042				ION=[M+H+H]2+		0.00	980.00	24.67	3571.33
1540	522.49	712.26448				ION=[M+H]+		2460.77	467.50	1194.17	1143.00
1541	522.89	1250.74389				ION=[M+H+H]2+		23.85	8001.67	698.17	18019.67
1542	524.9	324.06512				ION=[M+H+H]2+		1182.31	290.67	667.33	0.00
1543	525.92	431.13764	18.484	6-(2-amino-6-ethoxy-9h-purin-9-yl)-7-fluoro-7-methyl-2-(propan-2-yloxy)-hexahydro-2??-furo[3,2-d][1,3,2]dioxaphosphinin-2-one	C16H23FN5O6P	ION=[M+H]+		1276.31	2975.67	1875.50	3374.00
1544	526.63	315.27763				ION=[M+H]+		0.00	15.17	0.00	3686.67

1545	529.9	252.1373	13.797	(8aR,12S,12aR)-12-Hydroxy-4-methyl-4,5,6,7,8,8a,12,12a-octahydro-2H-3-benzoxecine-2,9(1H)-dione	C14H20O4	ION=[M+H]+		2243.08	1217.00	491.83	1532.00
1546	530.54	1266.77269				ION=[M+H+H]2+		10.92	4112.67	516.17	4580.33
1547	530.58	483.20152				ION=[M+H]+		2488.31	2433.00	2112.83	390.00
1548	530.6	338.98136				ION=[M+H]+		3063.85	2274.00	1674.67	6816.33
1549	530.74	162.03248	3.352	4-hydroxycoumarins	C9H6O3	ION=[M+H]+	1076-38-6	31544.15	18197.50	14326.00	61250.33
1550	530.77	459.03583				ION=[M+H+H]2+		843.38	207.33	130.50	2262.00
1551	530.92	1264.75735				ION=[M+H+H]2+		0.00	2822.50	268.17	7400.67
1552	530.92	310.98623				ION=[M+H]+		4272.92	2870.50	2398.50	9089.33
1553	531.51	702.27095				ION=[M+H+H]2+		859.23	1934.17	1371.83	66.00
1554	532.88	388.11936				ION=[M+H+H]2+		0.00	1370.33	8.83	0.00
1555	535.05	728.35212				ION=[M+H+H]2+		1752.15	134.50	63.33	84.67
1556	538.69	1230.65849				ION=[M+H+H]2+		0.00	6025.50	250.00	0.00
1557	542.44	406.26572				ION=[M+H+H]2+		0.00	568.83	1014.17	0.00
1558	543.05	1244.67408				ION=[M+H+H]2+		25.08	16066.00	4914.00	0.00
1559	544.19	203.13254	40.007	Aphanorphine	C13H17NO	ION=[M+H]+		8715.08	1957.83	2498.67	5616.33
1560	545.53	452.23848				ION=[M+H+H]2+		1592.77	36.67	119.17	0.00
1561	545.97	1775.5951				ION=[M+H+H]2+		0.00	0.00	1833.50	85.33
1562	546.49	317.27226	4.746	Amorolfine	C21H35NO	ION=[M+H]+		2347.85	2466.00	2685.50	5258.00
1563	546.5	551.28755	5.899	Sinefungin VA	C23H37N9O7	ION=[M+H]+		0.00	0.00	5620.50	67.33
1564	547.51	1775.92514				ION=[M+H+H]2+		13.54	26.00	4099.00	258.67
1565	547.91	1258.68915				ION=[M+H+H]2+		53.23	6509.67	2110.33	39.33
1566	547.94	690.25762				ION=[M+H]+		4919.85	534.33	124.83	539.00
1567	549.71	221.16372				ION=[M+H]+		2556.15	2084.33	1279.00	4918.00
1568	550.6	1776.25839				ION=[M+H+H]2+		12.92	0.00	5723.33	365.00
1569	550.84	321.06508				ION=[M+H]+		1534.92	1544.83	1140.67	4056.33
1570	551.42	739.28426				ION=[M+H+H]2+		0.00	2678.83	0.00	0.00
1571	551.44	2663.39248				ION=[M+H+H2]3+		0.00	0.00	1677.50	170.33
1572	552.94	2662.89065				ION=[M+H+H2]3+		35.38	106.33	4857.00	205.67
1573	554.81	512.24301				ION=[M+H+H]2+		1296.31	45.00	0.00	616.33
1574	555	199.06876				ION=[M+H]+		1941.85	720.33	699.17	2863.67
1575	556	256.12202	15.087	N-[4-(benzyloxy)phenyl]glycinamide	C15H16N2O2	ION=[M+H]+		5957.08	2302.50	1500.50	3268.67
1576	556.86	887.79605				ION=[M+H]+		15.23	47.17	3696.67	174.33
1577	558.84	1242.69428				ION=[M+H+H]2+		16.00	6170.17	1526.67	30.33
1578	559.54	2026.6034				ION=[M+H+H2]3+		67.54	0.00	1332.67	0.00
1579	559.65	208.14759	7.902	Nopyl acetate	C13H20O2	ION=[M+H]+		870.31	7586.17	496.67	1458.33
1580	560.25	675.86811				ION=[M+H]+		80.46	19.50	3020.67	38.67
1581	560.31	246.16267	7.259	1,3,5-Trimethyl-4-[2-(1,3,5-trimethyl-1H-pyrazol-4-yl)diaz-1-enyl]-1H-pyrazole	C12H18N6	ION=[M+H]+		2975.54	1525.00	848.17	2241.67
1582	560.75	1351.40432				ION=[M+H+H]2+		117.23	48.83	3368.00	0.00
1583	561.9	403.25937				ION=[M+H]+		0.00	1327.50	0.00	0.00
1584	562.25	1351.07046				ION=[M+H+H]2+		68.92	45.67	2707.83	0.00
1585	563.68	1252.72429				ION=[M+H+H]2+		11.23	6648.00	10780.33	4433.00
1586	564.06	238.15573	10.129	Saccharomonopyrone B	C14H22O3	ION=[M+H]+		736.00	3673.33	138.33	408.00
1587	565.06	645.34109				ION=[M+H]+		10.46	76.33	528.50	61.67
1588	565.64	1290.67279				ION=[M+H+H]2+		0.00	2805.67	2634.00	0.00
1589	566.29	381.1449				ION=[M+H]+		4419.85	155.00	133.00	1959.33
1590	566.52	236.14193	10.02	3-(heptyloxy)benzoic Acid	C14H20O3	ION=[M+H]+		1981.85	6009.17	177.83	490.67
1591	568.46	322.21451	18.34	Decylubiquinone	C19H30O4	ION=[M+H]+	55486-00-5	2396.46	2662.17	2671.17	2222.00
1592	569.45	350.20762				ION=[M+H]+		3171.85	2341.50	2037.50	1198.67
1593	570.65	1266.73862				ION=[M+H+H]2+		29.69	3012.33	5066.67	1105.33
1594	571.05	348.19189				ION=[M+H]+		1954.15	1360.17	891.17	732.33
1595	571.41	218.06026	13.188	4-Methylumbelliferyl acetate	C12H10O4	ION=[M+H]+		44.00	3234.33	318.83	25.33
1596	573.27	1236.70668				ION=[M+H+H]2+		0.00	2475.17	537.50	295.33
1597	576.42	869.33489				ION=[M+H+H]2+		2362.46	288.17	1923.00	4477.67
1598	577.18	853.66373				ION=[M+H]+		966.62	3738.67	3945.17	0.00
1599	577.37	1208.69571				ION=[M+H+H]2+		0.00	4232.17	12776.17	7423.67
1600	577.58	345.28722				ION=[M+H]+		0.00	647.67	1679.50	135161.67
1601	577.74	853.46312				ION=[M+H]+		1204.62	3176.00	3169.50	45.33
1602	578.77	404.19724				ION=[M+H+H]2+		1929.69	699.00	0.00	0.00
1603	578.8	853.86444				ION=[M+H]+		1634.31	3182.33	3525.33	37.00
1604	579.09	336.1341				ION=[M+H+H]2+		1697.08	824.33	1303.00	0.00
1605	579.39	1246.64547				ION=[M+H+H]2+		0.00	1605.33	3617.33	294.00
1606	579.62	243.1253	11.534	2-(4-Methoxybenzylidene)quinuclidin-3-one	C15H17NO2	ION=[M+H]+		4232.62	2682.50	2747.67	1170.33
1607	581.79	1250.72242				ION=[M+H+H]2+		0.00	7306.50	7881.83	7434.67
1608	581.88	1289.66899				ION=[M+H+H]2+		13.69	2022.83	1345.83	83.00
1609	582.24	197.14431				ION=[M+H]+		2682.77	1681.50	575.33	1337.00
1610	582.41	297.04302				ION=[M+H]+		2144.77	1508.83	849.67	1355.00
1611	582.54	1288.65624				ION=[M+H+H]2+		18.62	2399.17	1481.33	206.33
1612	583.59	325.03835				ION=[M+H]+		3478.00	2562.17	1253.17	2251.33
1613	583.71	343.27192				ION=[M+H]+		0.00	522.50	975.00	218124.33
1614	585.25	249.10516				ION=[M+H]+		17064.92	3013.83	0.00	4223.67

1615	586	1288.68691				ION=[M+H+H]2+		0.00	548.33	0.00	0.00
1616	586.12	644.34511	9.559	Virescenoside R	C32H52O13	ION=[M+H]+		0.00	1737.67	1947.00	0.00
1617	588.47	674.35225				ION=[M+H]+		2710.77	2029.83	2922.17	3906.00
1618	588.9	1264.72617				ION=[M+H+H]2+		19.85	3709.83	4070.50	1634.33
1619	589.12	459.33063				ION=[M+H]+		3698.31	280.83	298.67	5353.67
1620	589.84	248.14254	NA	Parthenium	C15H20O3	ION=[M+H]+		552.77	3303.00	183.67	395.33
1621	590.28	352.22301				ION=[M+H]+		2558.77	1597.33	1803.00	811.00
1622	590.62	1264.75144				ION=[M+H+H]2+		0.00	2170.00	2968.67	1094.33
1623	590.75	294.22007	9.278	9-Oxo-10(E),12(E)-octadecadienoic acid	C18H30O3	ION=[M+H]+		3745.38	1718.50	2098.00	3002.33
1624	591.6	730.29854				ION=[M+H+H]2+		1667.54	2364.83	1905.17	424.67
1625	591.88	316.14297				ION=[M+H]+		13079.54	8377.33	19318.83	6528.67
1626	592.65	851.36133				ION=[M+H+H]2+		1507.54	2603.67	1391.33	2354.33
1627	593.04	632.28658				ION=[M+H+H]2+		0.00	490.17	0.00	901.67
1628	593.46	250.067	17.865	4-(Morpholin-4-yl)-7-nitro-2,1,3-benzoxadiazole	C10H10N4O4	ION=[M+H]+	18378-24-0	578.15	22086.33	297.50	531.33
1629	596.31	208.10898	9.391	2,2-Dimethyl-3-hydroxy-3-(p-tolyl)propionic acid	C12H16O3	ION=[M+H]+		1909.38	1506.33	1294.33	745.00
1630	597.19	853.34138				ION=[M+H+H]2+		4904.00	1356.17	6158.00	12020.00
1631	597.75	337.07599	16.958	2,6-Difluorophenyl N-(2,6-dimethoxybenzoyl)carbamate	C16H13F2NO5	ION=[M+H]+		33.54	3498.00	0.00	0.00
1632	597.8	1328.70889	6.79	Bisoxazolomycin	C70H100N6O19	ION=[M+H+H]2+		26.31	12417.67	0.00	0.00
1633	601.58	324.22923	17.046	TOFA	C19H32O4	ION=[M+H]+		4578.62	3116.33	3772.83	5104.00
1634	602.89	248.19847				ION=[M+H]+		2329.23	3392.33	1652.00	3153.33
1635	603.64	637.81236				ION=[M+H]+		0.00	1190.00	1421.67	5101.67
1636	608.45	1273.61058				ION=[M+H+H]2+		0.00	471.33	487.17	5250.67
1637	609.89	441.67488				ION=[M+H]+		0.00	571.17	0.00	0.00
1638	611.1	899.34638				ION=[M+H+H]2+		2342.31	1572.67	454.67	961.33
1639	611.45	473.34654				ION=[M+H]+		38889.85	1367.67	732.67	38740.00
1640	617.14	457.35137				ION=[M+H]+		6693.23	811.33	1391.67	2980.67
1641	618.8	222.05757				ION=[M+H]+		38345.38	42573.33	26746.33	51622.33
1642	619.06	278.08653	39.988	Pukeleimide A	C13H14N2O5	ION=[M+H]+		3539.23	3606.00	3448.83	6508.00
1643	620.05	791.42778				ION=[M+H]+		591.23	1602.50	7298.67	5803.67
1644	620.11	791.22818				ION=[M+H]+		720.00	560.83	5470.50	5072.33
1645	620.52	809.3127				ION=[M+H+H]2+		265.38	20.33	239.83	4486.67
1646	621.66	735.49507				ION=[M+H]+		28.77	20.83	0.00	3891.67
1647	622.48	140.9506				ION=[M+H]+		776.31	567.50	597.67	233.00
1648	622.49	466.19788				ION=[M+H]+		1019.69	3041.83	1364.17	684.00
1649	625.28	227.18966				ION=[M+H]+		4792.62	5398.67	4617.17	5562.67
1650	626.3	222.08547	NA	Diethyl phthalate	C12H14O4	ION=[M+H]+		133.54	180.83	269.67	8271.67
1651	626.4	148.01611	6.523	1,3-Isobenzofurandione	C8H4O3	ION=[M+H]+		466.15	518.33	412.50	7787.33
1652	627.17	686.27771				ION=[M+H+H]2+		358.31	2230.67	2107.83	0.00
1653	627.25	630.33106				ION=[M+H]+		36.31	0.00	530.50	526.00
1654	627.25	219.03699	13.915	3-demethylchuangxinmycin	C11H9NO2S	ION=[M+H]+		304.46	750.00	766.83	4903.33
1655	627.28	1260.6583				ION=[M+H+H]2+		458.15	9613.33	2486.17	438.67
1656	627.56	1276.63027				ION=[M+H+H]2+		12.15	4777.83	2069.50	6961.33
1657	627.56	1244.67876				ION=[M+H+H]2+		0.00	3137.33	1087.50	771.67
1658	627.56	1184.61583				ION=[M+H+H]2+		0.00	4225.67	419.50	0.00
1659	628.11	1273.62119				ION=[M+H+H]2+		27.54	4643.50	1364.17	7444.33
1660	628.51	1274.63784				ION=[M+H+H]2+		0.00	2780.33	1138.67	6343.67
1661	628.83	1266.73809				ION=[M+H+H]2+		0.00	186.83	2692.00	6707.00
1662	630.25	851.32615				ION=[M+H+H]2+		2036.77	172.67	1588.50	113.00
1663	631	2526.38676				ION=[M+H+H]2+		0.00	0.00	1638.17	3285.67
1664	631	829.33893				ION=[M+H+H]2+		2545.08	316.50	20.17	61.33
1665	631.24	350.24501				ION=[M+H]+		5525.23	3353.00	6567.50	7280.67
1666	631.82	1263.19344				ION=[M+H]+		0.00	0.00	0.00	2496.33
1667	632.53	1264.19183				ION=[M+H]+		0.00	75.00	1105.33	6517.33
1668	632.61	2527.40738				ION=[M+H+H]2+		0.00	25.83	441.50	975.33
1669	632.94	1289.64115				ION=[M+H+H]2+		0.00	0.00	0.00	8691.00
1670	632.94	1263.69856				ION=[M+H]+		0.00	50.00	524.83	3397.67
1671	633.05	644.8193				ION=[M+H]+		17.23	12537.17	27337.33	43263.67
1672	633.26	1264.19884				ION=[M+H]+		0.00	100.67	562.50	1281.67
1673	633.44	1263.68939				ION=[M+H]+		12.00	59.83	2583.00	9624.00
1675	634.34	1290.64239				ION=[M+H+H]2+		0.00	10649.83	26020.67	34548.00
1676	634.51	1258.70258				ION=[M+H+H]2+		16.77	6184.00	12484.83	7700.33
1677	634.85	1264.72366				ION=[M+H+H]2+		10.46	110.83	613.00	8541.00
1678	635.2	645.319				ION=[M+H]+		0.00	62.00	38.33	6479.33
1679	635.43	486.2825				ION=[M+H]+		2641.08	1388.67	2465.50	4159.33
1680	635.58	1274.67487				ION=[M+H+H]2+		14.77	16523.17	35868.33	2485.33
1681	635.89	648.33546				ION=[M+H]+		2363.38	1277.17	2077.83	2922.00
1683	636.42	665.36522				ION=[M+H]+		1828.15	174.83	1229.33	1090.67
1684	636.54	648.32696				ION=[M+H]+		217.23	1315.67	3579.00	4678.00
1685	637.48	2522.32912				ION=[M+H+H2]3+		26.15	31.83	0.00	3547.67
1686	637.61	2522.34512				ION=[M+H+H]2+		0.00	57.83	40.50	7798.33
1687	637.7	1237.57689				ION=[M+H+H]2+		23.23	1173.83	0.00	0.00
1688	637.83	1236.58694				ION=[M+H+H]2+		22.15	1998.50	0.00	0.00
1689	637.85	928.5103				ION=[M+H]+		754.00	1905.33	1284.00	1988.67

1690	637.9	1235.22377				ION=[M+H]+		22.31	0.00	28.50	9071.67
1691	638.11	1262.18204				ION=[M+H]+		0.00	79.17	257.33	6658.33
1692	638.27	618.29357				ION=[M+H]+		0.00	867.50	0.00	0.00
1693	638.27	1232.59304				ION=[M+H+H]2+		0.00	6055.50	4970.00	0.00
1694	638.37	1272.67142				ION=[M+H+H]2+		24.77	11029.00	18059.83	2285.00
1695	638.9	867.35362				ION=[M+H+H]2+		18586.31	29161.00	16451.33	30752.67
1696	638.98	1270.54023				ION=[M+H+H]2+		0.00	1871.67	1358.17	0.00
1698	639.15	1367.73603				ION=[M+H]+		557.85	785.00	4318.17	888.67
1699	639.4	1256.69346				ION=[M+H+H]2+		0.00	5336.83	6393.50	8814.67
1700	639.67	1368.07032				ION=[M+H]+		677.08	861.33	4162.50	1127.33
1701	640.08	1167.31289				ION=[M+H]+		9789.08	5567.00	21065.17	13267.67
1702	640.37	363.12682				ION=[M+H]+		3669.69	1384.67	883.33	966.67
1703	640.4	1167.64743				ION=[M+H]+		10210.92	6101.83	24398.67	13495.00
1704	640.74	1287.62687				ION=[M+H+H]2+		22.15	10631.83	13200.83	50762.67
1705	640.85	1251.75432				ION=[M+H]+		0.00	1076.00	7511.00	21339.00
1706	641.03	1267.76583				ION=[M+H+H]2+		0.00	364.00	2261.00	3940.00
1707	641.08	1303.65331				ION=[M+H+H]2+		0.00	1389.00	0.00	0.00
1708	641.21	362.20726	16.536	5-Fluoro ADB-PINACA	C19H27FN4O2	ION=[M+H]+		1774.62	1255.83	1061.50	363.00
1709	641.4	271.25088				ION=[M+H]+		1058.31	1104.33	1029.33	5587.00
1710	641.45	651.82613				ION=[M+H]+		11.08	5056.33	16845.67	18955.67
1711	641.45	1751.96438				ION=[M+H+H]2+		1135.08	1258.67	3517.33	1453.33
1712	641.72	1751.46593				ION=[M+H+H]2+		1964.15	1816.67	4554.83	2041.33
1713	641.74	1250.74101				ION=[M+H+H]2+		14.62	28914.33	102914.50	143228.00
1714	641.77	1250.76566				ION=[M+H]+		0.00	1367.17	1091.67	229.33
1715	641.85	1750.95841				ION=[M+H+H]2+		1475.38	1590.50	5203.33	1775.33
1716	642.38	1304.65712				ION=[M+H+H]2+		26.77	5941.50	15320.00	20021.00
1717	642.51	1204.73389				ION=[M+H+H]2+		14.77	1968.67	927.00	4545.67
1718	643.45	1288.68453				ION=[M+H+H]2+		0.00	8902.00	18937.17	0.00
1719	643.74	1367.40106				ION=[M+H]+		379.08	739.50	2620.17	63.00
1720	643.79	1272.70733				ION=[M+H+H]2+		19.54	2434.00	6663.67	3528.67
1721	644.24	1276.19165				ION=[M+H]+		0.00	0.00	114.17	3907.00
1722	644.26	4101.1968				ION=[M+H+H2]3+		194.77	176.83	3328.67	0.00
1723	644.47	1248.72595				ION=[M+H+H]2+		48.46	28095.83	50288.33	362887.33
1724	644.49	1275.6924				ION=[M+H]+		0.00	0.00	72.00	5963.00
1725	644.73	1277.19194				ION=[M+H]+		0.00	0.00	17.67	2098.33
1726	645.12	1270.70666				ION=[M+H+H]2+		22.00	2670.50	3652.83	6873.00
1727	645.21	1248.73947				ION=[M+H]+		0.00	2077.00	4201.00	58360.67
1728	645.44	650.82152				ION=[M+H]+		0.00	5562.17	10271.33	36826.00
1729	646.08	1302.64464				ION=[M+H+H]2+		14.46	4861.83	9020.33	35130.67
1730	646.29	1265.75615				ION=[M+H+H]2+		12.62	424.83	1435.33	9932.33
1731	646.91	1301.64002				ION=[M+H+H]2+		0.00	787.67	0.00	6886.33
1732	647.1	324.19513				ION=[M+H]+		2906.46	1259.50	1042.33	3347.00
1733	647.47	1220.72999				ION=[M+H+H]2+		19.69	12118.17	4002.83	13102.00
1734	647.98	1242.70108				ION=[M+H+H]2+		0.00	1963.67	353.67	446.33
1735	648.35	1258.67931				ION=[M+H+H]2+		0.00	4401.00	1046.00	877.67
1736	648.54	1274.64991				ION=[M+H+H]2+		100.92	2912.50	949.67	3280.00
1737	648.54	1286.67046				ION=[M+H+H]2+		14.15	7056.67	9913.33	1940.33
1738	649.33	1770.44171				ION=[M+H+H]2+		1096.31	985.83	1670.33	0.00
1739	649.83	403.19967				ION=[M+H]+		2270.46	2027.17	1908.00	3526.33
1740	649.88	1273.64718				ION=[M+H+H]2+		0.00	3074.83	959.00	3825.00
1741	650.26	386.17348	7.427	(2Z,4S,5S,6S,7R,8Z)-2,9-diphenyldeca-2,8-diene-3,4,5,6,7,8-hexol	C22H26O6	ION=[M+H]+	69158-41-4	4800.31	4336.17	4433.00	7341.00
1742	651	224.1898				ION=[M+H]+		9790.15	12548.67	6854.17	14379.00
1743	652.02	1320.69646				ION=[M+H]+		1074.46	1555.17	343.50	0.00
1744	653.27	1235.76289				ION=[M+H]+		0.00	2065.33	7843.50	31446.67
1745	653.78	1261.71032				ION=[M+H]+		0.00	0.00	413.33	4343.00
1746	654.03	1256.66642				ION=[M+H+H]2+		12.00	2832.83	355.33	478.67
1747	654.55	1236.77341				ION=[M+H]+		0.00	470.67	2176.00	1751.00
1748	655.35	1319.9518				ION=[M+H]+		1255.85	657.67	378.00	2739.67
1749	655.62	643.82925				ION=[M+H]+		0.00	6790.67	16830.50	42692.67
1750	655.74	1262.74507				ION=[M+H+H]2+		9.38	138.17	177.00	5197.00
1751	655.77	1287.6585				ION=[M+H+H]2+		0.00	536.67	0.00	0.00
1752	655.9	1067.34096				ION=[M+H]+		841.23	542.83	2490.83	958.67
1753	655.96	1320.70226				ION=[M+H]+		5818.15	4188.33	24707.33	16342.33
1754	656.62	1234.74603				ION=[M+H+H]2+		0.00	35544.50	92839.83	278635.67
1755	656.66	1066.94108				ION=[M+H]+		759.85	782.00	3079.50	891.00
1756	656.81	1288.66352				ION=[M+H+H]2+		11.23	7030.83	15841.50	34697.00
1757	656.85	1234.7613				ION=[M+H]+		0.00	3749.00	11645.00	43507.00
1758	656.9	352.26029				ION=[M+H]+		2533.54	1485.00	2175.67	3644.67
1759	656.95	1066.73902				ION=[M+H]+		718.31	589.50	3055.83	1275.00
1760	657.27	1056.35723				ION=[M+H]+		2146.62	1922.67	11735.67	7171.67
1761	657.31	1056.15665				ION=[M+H]+		2134.15	1992.17	11400.67	6875.67
1762	657.33	1067.13827				ION=[M+H]+		821.23	651.83	3432.17	1378.00
1763	657.54	1055.9557				ION=[M+H]+		1109.23	1302.67	6985.17	3464.00

1764	657.67	1056.75387				ION=[M+H]+		835.38	941.67	6960.83	3497.67
1765	657.85	1256.72535				ION=[M+H+H]2+		42.31	3877.17	6223.83	5530.67
1766	658.22	2639.90289				ION=[M+H+H]2+		3530.92	4319.00	28064.33	11982.95
1767	658.43	1272.69477				ION=[M+H+H]2+		10.46	10113.33	18703.17	1093.00
1768	658.51	688.29399				ION=[M+H+H]2+		210.00	1524.33	1378.50	0.00
1769	658.97	1056.55858				ION=[M+H]+		1679.23	1993.33	10181.67	5883.00
1770	659.04	1196.65206				ION=[M+H+H]2+		0.00	7161.83	3440.67	0.00
1771	659.13	1259.19391				ION=[M+H]+		0.00	0.00	0.00	1186.00
1772	659.42	1259.20505				ION=[M+H]+		0.00	0.00	0.00	1574.33
1773	659.57	1232.72987				ION=[M+H+H]2+		13.54	27361.00	37997.83	493810.33
1774	660.17	1286.65005				ION=[M+H+H]2+		38.31	5785.17	5598.00	39416.67
1775	660.36	1257.72033				ION=[M+H+H]2+		9.54	2537.33	3735.67	3272.00
1776	660.52	2518.3883				ION=[M+H+H]2+		0.00	10.83	0.00	5151.00
1777	660.54	1259.69513				ION=[M+H]+		6.31	26.67	0.00	7321.67
1778	660.94	1260.20322				ION=[M+H]+		0.00	0.00	0.00	6509.00
1779	660.97	851.32688				ION=[M+H+H]2+		6575.08	2155.33	10899.17	17434.00
1780	661.1	1254.70944				ION=[M+H+H]2+		0.00	2538.50	2657.00	7420.33
1781	661.21	643.32393				ION=[M+H]+		24.15	0.00	421.00	0.00
1782	661.73	1259.70759				ION=[M+H]+		0.00	21.83	0.00	1289.33
1783	661.75	2273.18939				ION=[M+H+H]2+		70.15	0.00	559.67	0.00
1784	661.95	2272.67985				ION=[M+H+H]2+		290.00	378.83	1493.67	0.00
1785	662.02	1230.61233				ION=[M+H+H]2+		0.00	3697.83	2062.67	98.33
1786	662.92	1136.84331				ION=[M+H]+		1044.00	1126.17	3719.83	146.33
1787	662.95	1136.59335				ION=[M+H]+		788.62	621.33	2341.17	65.67
1788	663.09	849.34536				ION=[M+H+H]2+		2304.00	3862.00	2825.33	3918.33
1789	663.17	1137.09062				ION=[M+H]+		436.15	857.67	2695.33	234.33
1790	663.23	1264.56347				ION=[M+H+H]2+		0.00	447.17	0.00	0.00
1791	663.34	1268.56936				ION=[M+H+H]2+		11.08	2249.17	2637.33	0.00
1792	663.44	335.22311				ION=[M+H]+		30.00	355.33	3323.33	0.00
1793	663.57	1248.77563				ION=[M+H]+		0.00	1648.33	5255.00	13703.33
1794	663.65	642.82471				ION=[M+H]+		0.00	6221.33	6761.50	51988.33
1795	663.76	1249.77885				ION=[M+H]+		0.00	783.83	3919.17	9087.33
1796	664.8	1248.76118				ION=[M+H+H]2+		10.92	17131.83	47763.50	88371.33
1797	664.87	1302.67918				ION=[M+H+H]2+		111.08	3493.67	8414.83	12370.33
1798	665.07	650.83783				ION=[M+H]+		0.00	4483.83	10276.17	14224.67
1799	666.27	1286.70685				ION=[M+H+H]2+		30.62	4580.50	8231.50	178.67
1800	666.33	3484.94161				ION=[M+H+H2]3+		864.62	2230.00	9718.67	3112.33
1801	666.49	326.00096				ION=[M+H]+		4544.00	3607.83	2956.17	4621.00
1802	666.93	1161.98235				ION=[M+H]+		1688.92	3341.00	10444.33	3698.00
1803	667.08	1270.72161				ION=[M+H+H]2+		34.62	1912.83	1988.67	2421.33
1804	667.34	1270.7396				ION=[M+H+H]2+		10.00	398.00	1628.17	1037.33
1805	667.59	488.29875				ION=[M+H]+		2385.69	951.83	1866.17	4815.33
1806	667.6	643.35106				ION=[M+H]+		0.00	530.33	0.00	0.00
1807	667.7	1246.74712				ION=[M+H+H]2+		42.92	13923.17	20843.33	202299.00
1808	667.7	1246.76				ION=[M+H]+		0.00	1223.00	1061.17	27693.00
1809	668.23	1268.72585				ION=[M+H+H]2+		0.00	1408.67	1642.83	4389.67
1810	668.28	650.35324				ION=[M+H]+		2259.23	741.33	188.83	0.00
1811	668.39	1118.5988				ION=[M+H]+		1943.54	1034.00	6036.67	5643.33
1812	668.51	667.38152				ION=[M+H]+		1929.23	176.50	1122.67	1615.33
1813	668.63	4085.18853				ION=[M+H+H2]3+		78.46	224.17	1527.50	529.00
1814	668.65	326.24515				ION=[M+H]+		3680.31	1550.33	3421.17	7900.67
1815	668.8	1162.31885				ION=[M+H]+		1969.69	3505.67	6369.83	2518.33
1816	668.85	1362.40642				ION=[M+H]+		154.92	882.33	3581.17	1363.67
1817	669.03	1300.66759				ION=[M+H+H]2+		55.38	2595.83	2198.17	27731.00
1818	669.03	4470.41005				ION=[M+H+H2]3+		182.00	170.33	1219.50	340.00
1819	669.39	652.49164				ION=[M+H+H]2+		587.85	0.00	0.00	0.00
1820	669.76	1263.76728				ION=[M+H+H]2+		0.00	138.50	320.83	3456.33
1821	669.97	881.33534				ION=[M+H+H]2+		6836.15	3590.67	1037.50	2059.33
1822	670.68	171.16326	4.65	Decanamide	C10H21NO	ION=[M+H]+		5789.23	5402.83	4059.00	9999.67
1823	670.99	582.24908				ION=[M+H]+		117.38	1516.00	17.33	0.00
1824	671.48	721.516				ION=[M+H]+		221.08	117.00	93.00	5729.33
1825	671.52	642.85028				ION=[M+H]+		0.00	2680.83	2810.50	239.67
1826	671.56	378.24058				ION=[M+H]+		0.00	47.83	3527.50	0.00
1827	671.95	1162.65248				ION=[M+H]+		1008.00	2482.00	4028.17	1318.33
1828	672.29	649.83139				ION=[M+H]+		19.85	3420.33	4422.50	32731.67
1829	672.74	807.29895				ION=[M+H+H]2+		202.92	54.33	145.33	4178.33
1830	673.14	1284.68926				ION=[M+H+H]2+		0.00	3741.83	3712.33	1124.33
1831	673.72	253.94274				ION=[M+H]+		4257.54	4466.00	3888.50	1736.67
1832	677.34	1084.55766				ION=[M+H+H]2+		9.85	0.00	3074.67	44.00
1833	678.19	362.24483	14.235	Diheptyl phthalate	C22H34O4	ION=[M+H]+		0.00	0.00	4312.00	0.00
1834	678.46	524.22547				ION=[M+H+H]2+		311.85	723.50	3075.17	0.00
1835	678.89	897.33247	14.137	Mureidomycin C	C40H51N9O13S	ION=[M+H+H]2+		4480.77	3341.50	769.83	1373.67
1836	680.33	185.95002				ION=[M+H]+		7644.62	7838.83	8754.83	8643.33
1837	682.21	632.7819				ION=[M+H]+		0.00	5863.83	2633.83	0.00
1838	682.29	1263.5819				ION=[M+H+H]2+		0.00	1510.17	1836.33	0.00

1839	682.94	228.03656	9.975	2-(2-Thienyl)-1,4-dihydroquinazolin-4-one	C12H8N2OS	ION=[M+H]+		4033.38	93.50	125.17	1749.33
1840	683.15	1265.56872				ION=[M+H+H]2+		0.00	353.33	504.83	0.00
1841	683.26	1266.5706				ION=[M+H+H]2+		16.62	5043.67	2722.83	30.33
1842	683.35	1108.36143				ION=[M+H]+		800.62	534.33	3599.00	312.67
1843	683.84	625.30051				ION=[M+H]+		0.00	76.00	511.67	0.00
1844	684.81	1234.62281	7.862	Pseudomycin-C'	C53H91ClN12O19	ION=[M+H+H]2+		0.00	2520.83	1053.83	0.00
1845	687.49	542.19605				ION=[M+H+H]2+		1824.00	818.00	2253.00	3741.00
1846	687.93	503.30888				ION=[M+H]+		3721.23	840.33	4101.17	6776.00
1847	688.62	1256.67254				ION=[M+H+H]2+		22.00	346.67	4238.17	0.00
1848	690.71	897.36144				ION=[M+H+H]2+		739.08	1530.17	385.33	842.67
1849	690.86	1255.12716				ION=[M+H]+		0.00	269.00	2577.17	23.33
1850	691.56	325.37033				ION=[M+H]+		2778.00	1123.00	1332.33	2407.67
1851	691.91	729.28254				ION=[M+H]+		2803.85	109.33	943.83	5482.33
1852	692.01	1254.12781				ION=[M+H]+		13.38	277.83	2511.83	0.00
1853	692.15	1226.68587				ION=[M+H]+		16.00	3068.17	2213.83	0.00
1854	692.25	1279.57812				ION=[M+H+H]2+		0.00	12961.00	28658.67	38.67
1855	692.25	1253.62776				ION=[M+H]+		7.54	121.17	2418.50	26.67
1856	692.27	1254.62775				ION=[M+H]+		25.85	301.50	4163.50	0.00
1857	692.32	1258.62864				ION=[M+H+H]2+		0.00	97.00	2071.50	40.67
1858	692.35	732.27918				ION=[M+H+H]2+		2605.38	363.83	1256.17	5336.33
1859	692.42	1254.63624				ION=[M+H]+		0.00	327.00	1550.33	0.00
1860	692.52	437.25414				ION=[M+H]+		3597.85	1300.00	4821.00	1793.67
1861	692.89	1280.58024				ION=[M+H+H]2+		0.00	11603.17	27023.83	0.00
1862	692.94	514.31171	12.131	Microsporin B	C28H42N4O5	ION=[M+H]+		4365.23	452.50	3896.50	11505.00
1863	693.18	352.26121	5.163	1-[4-(2,4,4-trimethylpentan-2-yl)phenyl]-1,4,7,10-tetraoxaundecane	C21H36O4	ION=[M+H]+		10789.23	798.00	10073.83	28027.67
1864	693.33	2492.28441				ION=[M+H+H]2+		0.00	0.00	1297.17	0.00
1865	693.74	693.39539				ION=[M+H]+		3377.85	78.17	1951.33	5423.67
1866	693.74	849.34555				ION=[M+H+H]2+		7269.54	2183.00	86.83	865.33
1867	694.02	1248.64141				ION=[M+H+H]2+		19.85	5695.50	17000.83	0.00
1868	694.25	1264.61066				ION=[M+H+H]2+		0.00	13628.83	40643.67	0.00
1869	694.27	198.12722	7.529	Alkyldihydropyrone A	C11H18O3	ION=[M+H]+		1852.46	3312.17	950.67	4214.33
1870	695.08	324.21532				ION=[M+H]+		1769.54	1155.33	3847.17	5265.33
1871	696.16	1058.59106				ION=[M+H]+		1600.62	1488.67	5349.17	6039.67
1872	696.67	708.35748	8.407	VM48642	C40H52O11	ION=[M+H+H]2+		3574.00	1219.17	0.00	107.33
1873	697.4	1057.83854				ION=[M+H]+		384.00	759.50	1478.83	3345.67
1874	697.43	2115.68206				ION=[M+H+H]2+		1703.23	1518.33	5851.67	5902.33
1875	697.61	1058.09179				ION=[M+H]+		2014.46	2413.17	9668.67	9584.33
1876	697.92	1274.63945				ION=[M+H+H]2+		0.00	1046.50	3809.33	128.33
1877	698.58	1410.45574				ION=[M+H]+		623.69	622.67	2083.33	1433.33
1878	699.08	1267.13813				ION=[M+H]+		0.00	0.00	979.50	0.00
1879	699.12	248.14267				ION=[M+H]+		3156.00	2937.83	397.17	3288.33
1880	699.38	578.28527				ION=[M+H+H]2+		3724.46	607.33	0.00	0.00
1881	699.81	1296.59714				ION=[M+H+H]2+		0.00	7158.83	18577.00	0.00
1882	699.83	1257.70169				ION=[M+H+H]2+		0.00	825.00	3224.50	8.67
1883	700	1240.6912				ION=[M+H]+		0.00	2768.33	20875.67	0.00
1884	700.33	316.20413	18.855	Cafestol	C20H28O3	ION=[M+H]+		1946.62	1468.83	3760.00	3784.33
1885	700.45	1240.67825				ION=[M+H+H]2+		47.85	35334.00	153897.33	0.00
1886	700.46	1294.59463				ION=[M+H+H]2+		28.31	7044.67	18926.33	0.00
1887	701.4	1262.65753				ION=[M+H+H]2+		45.85	2537.17	9392.50	0.00
1888	701.7	1278.62652				ION=[M+H+H]2+		27.23	7064.83	26102.50	0.00
1889	703.42	333.09943	14.811	Flurtamone	C18H14F3NO2	ION=[M+H]+	96525-23-4	1676.77	1204.83	646.33	2449.67
1890	703.77	865.33945				ION=[M+H+H]2+		21616.31	38527.00	34113.50	62648.00
1891	703.83	479.20579	13.976	Nicardipine	C26H29N3O6	ION=[M+H]+		6663.85	10999.00	11318.00	6081.00
1892	704.34	821.34785				ION=[M+H+H]2+		1431.08	127.67	100.67	230.33
1893	705.38	526.25818				ION=[M+H+H]2+		0.00	38.83	452.33	4422.33
1894	706.18	708.28389				ION=[M+H+H]2+		2079.54	823.50	1516.33	4473.33
1895	706.44	705.28499				ION=[M+H]+		2090.15	524.17	1407.67	3823.67
1896	706.94	388.19099				ION=[M+H+H]2+		0.00	0.00	983.33	0.00
1897	706.95	490.31413	11.305	Sapacitabine	C26H42N4O5	ION=[M+H]+	151823-14-2	3367.38	921.00	2257.33	7955.00
1898	707.11	194.09556	10.222	Nakienone A	C11H14O3	ION=[M+H]+		3346.92	3459.67	2311.33	0.00
1899	707.62	328.26092	9.135	Valsafungin B	C19H36O4	ION=[M+H]+		9896.15	2021.50	5881.67	22249.67
1900	707.98	669.39594				ION=[M+H]+		3122.31	288.00	1529.33	5101.33
1901	710.16	212.14494				ION=[M+H]+		2334.62	1865.67	1194.00	1732.33
1902	711.58	864.62044				ION=[M+H+H2]3+		413.69	0.00	0.00	0.00
1903	715.9	1278.60074				ION=[M+H+H]2+		44.00	7320.83	18308.00	75.00
1905	716.24	1224.69631				ION=[M+H]+		0.00	2579.00	13986.33	0.00
1906	716.55	526.24089				ION=[M+H+H]2+		221.54	657.50	2473.00	78.00
1907	717.28	1262.6307				ION=[M+H+H]2+		0.00	7319.33	22381.00	0.00
1908	717.9	451.26968	8.695	Microtermolide A	C23H37N3O6	ION=[M+H]+		6763.23	10890.00	5198.17	10554.33
1909	719.2	360.19191				ION=[M+H]+		2319.23	960.17	1794.50	1367.00
1910	720.58	1246.62054				ION=[M+H+H]2+		0.00	678.83	4788.50	0.00
1912	723.04	1230.6492				ION=[M+H+H]2+		0.00	615.67	2692.83	0.00
1913	723.18	1294.61472				ION=[M+H+H]2+		16.00	4164.17	11150.50	0.00

1914	723.31	493.31708				ION=[M+H]+		2106.62	1739.33	3011.17	3310.67
1915	723.34	1239.71367				ION=[M+H]+		0.00	808.50	3504.17	15.67
1916	723.54	1238.71381				ION=[M+H]+		0.00	1031.17	6102.17	12.67
1917	724.03	1292.61621				ION=[M+H+H]2+		0.00	3779.83	11498.00	0.00
1918	724.44	1238.69836				ION=[M+H+H]2+		17.08	19163.17	75944.00	0.00
1919	724.66	544.21384				ION=[M+H+H]2+		2381.69	1335.00	2307.50	4973.67
1920	725.18	541.2113	18.626	SMTP-42	C29H35NO7S	ION=[M+H]+		2232.15	1011.00	1907.83	4919.00
1921	725.19	1260.67476				ION=[M+H+H]2+		65.23	1501.00	3517.50	0.00
1922	725.64	1276.64631				ION=[M+H+H]2+		0.00	3535.33	10710.50	0.00
1923	725.93	678.38436	18.724	18-O-b-d-Glucopyranosyl-18R-hydroxydihydroalloprotolichesterinate21-O-a-l-rhamnopyranoside	C33H58O14	ION=[M+H]+		1380.92	183.33	1612.50	3032.00
1924	726.51	439.26988				ION=[M+H]+		3800.62	9334.00	3232.83	3517.00
1925	726.65	516.32771	18.77	IC202C	C23H44N6O7	ION=[M+H]+		1393.23	56.50	1663.83	3736.00
1926	726.9	354.2768	11.714	2-Linoleoyl glycerol	C21H38O4	ION=[M+H]+		2680.46	401.50	3197.00	7182.67
1927	727.5	1206.72982				ION=[M+H]+		21.23	100.00	2141.33	0.00
1928	729.59	1206.71622	7.2	Oasomycin C	C61H106O23	ION=[M+H+H]2+		0.00	2592.50	30849.83	287.33
1929	733.88	526.33142				ION=[M+H+H]2+		16.77	0.00	507.50	4296.67
1930	734.24	881.33194				ION=[M+H+H]2+		2244.00	756.17	315.50	839.33
1931	735.19	281.19955	15.004	Penibruguieramine A	C16H27NO3	ION=[M+H]+		2080.00	3315.17	60.83	29.67
1932	735.28	3591.85549				ION=[M+H+H2]3+		230.31	326.00	939.00	836.67
1933	736.99	233.15538				ION=[M+H]+		1061.85	2099.83	938.17	2784.67
1934	738.25	865.33946				ION=[M+H+H]2+		26836.92	9306.17	1099.50	3934.33
1935	738.37	453.2851	8.472	Glycerophospho-N-palmitoyl ethanolamine	C21H44NO7P	ION=[M+H]+		6836.92	241.33	331.67	1046.00
1936	740.44	501.28513				ION=[M+H]+		0.00	0.00	7374.67	0.00
1937	740.68	245.0518				ION=[M+H]+		0.00	24159.00	124.67	0.00
1938	740.73	222.05774				ION=[M+H]+		16534.92	18894.17	6979.50	18422.33
1939	740.94	298.06351				ION=[M+H]+		3212.15	3689.50	1485.50	3906.00
1940	740.95	296.07549				ION=[M+H]+		9412.31	10626.33	3407.83	9957.00
1941	742.7	592.15164				ION=[M+H+H]2+		50.00	0.00	675.00	0.00
1942	743.56	1244.64173				ION=[M+H+H]2+		0.00	367.17	2569.00	0.00
1943	743.72	274.15488				ION=[M+H]+		1502.31	711.17	878.33	343.67
1944	743.97	621.81702				ION=[M+H]+		0.00	0.00	944.67	0.00
1945	744.04	1246.643				ION=[M+H+H]2+		16.00	427.33	2719.17	0.00
1946	744.04	1243.6341				ION=[M+H+H]2+		0.00	462.33	2211.50	0.00
1947	745	355.14911				ION=[M+H]+		1322.46	1482.33	1791.83	2254.00
1948	745.22	543.33283				ION=[M+H]+		12.92	0.00	2146.50	0.00
1949	745.4	1190.72332	17.991	Desertomycin B	C61H106O22	ION=[M+H+H]2+		56.46	2182.50	21864.83	0.00
1950	745.58	692.32436				ION=[M+H+H]2+		84.31	1309.83	2988.33	0.00
1951	747.95	749.34611				ION=[M+H]+		14.15	12873.50	0.00	26.33
1952	751.02	1084.5412				ION=[M+H+H]2+		1531.38	200.83	2310.83	3472.00
1953	751.77	570.22918				ION=[M+H+H]2+		3894.77	1036.33	5732.50	6619.67
1954	751.89	260.21415				ION=[M+H]+		3897.54	829.00	5727.17	9020.00
1955	751.99	531.3409	6.63	AAL Toxin Te1	C27H49NO9	ION=[M+H]+		9688.00	818.67	11165.33	15163.33
1956	752.3	567.22703	14.38	Saframycin C	C29H33N3O9	ION=[M+H]+		2420.77	448.83	3254.17	4196.33
1957	753.04	391.18494	19.904	Orysastrobin	C18H25N5O5	ION=[M+H+H]2+	248593-16-0	779.85	2047.00	712.83	102.67
1958	753.18	1204.73601				ION=[M+H+H]2+		0.00	1769.00	12024.33	0.00
1959	754.19	707.29671				ION=[M+H]+		2279.38	479.33	1966.83	4730.67
1960	754.21	552.25682				ION=[M+H+H]2+		440.00	201.17	2939.33	0.00
1961	755.57	420.20853				ION=[M+H+H]2+		0.00	0.00	156.50	1715.67
1962	756.19	671.41105				ION=[M+H]+		4791.08	351.67	2508.50	7206.33
1963	756.25	210.10387	7.615	Dibutyl phosphate	C8H19O4P	ION=[M+H]+		8594.15	10012.83	7518.00	9331.33
1964	756.55	154.03973	5.744	Diethyl phosphate	C4H11O4P	ION=[M+H]+		3022.62	3528.00	2672.17	4030.00
1965	757.18	913.31078				ION=[M+H+H]2+		2550.46	1594.00	303.83	1387.67
1966	758.01	465.28678				ION=[M+H]+		1593.85	425.50	1053.33	3260.00
1967	763.94	528.25664				ION=[M+H+H]2+		178.31	479.33	3277.83	0.00
1968	763.99	1284.01606				ION=[M+H]+		3691.38	2839.00	3871.83	233.00
1969	764.47	1284.67749				ION=[M+H]+		309.54	1458.50	888.17	233.67
1970	764.78	3851.0464				ION=[M+H+H2]3+		3666.46	2193.00	4224.83	60.67
1971	764.81	1284.34921				ION=[M+H]+		3578.62	3420.67	4876.33	277.67
1972	767.38	546.22825				ION=[M+H+H]2+		2147.85	985.00	2355.67	3596.67
1973	768.44	564.24011				ION=[M+H+H]2+		1914.15	1226.67	3281.00	3832.67
1974	768.56	192.15267	11.169	4-Heptylphenol	C13H20O	ION=[M+H]+		8260.31	2390.00	7039.17	12175.67
1975	768.92	576.45751				ION=[M+H+H2]3+		0.00	399.50	675.67	0.00
1976	769.18	849.34274				ION=[M+H+H]2+		1657.54	518.83	395.17	817.00
1977	770.08	1026.66307				ION=[M+H+H2]3+		443.85	0.00	0.00	0.00
1978	771.55	1036.54073				ION=[M+H+H]2+		1861.85	667.67	1811.67	6106.67
1979	771.79	507.33746				ION=[M+H]+		8699.85	2511.67	6516.83	19159.00
1980	772.29	572.26334				ION=[M+H]+		3928.92	853.83	14.67	0.00
1981	772.37	543.22596				ION=[M+H]+		2249.23	923.33	2183.83	5258.33
1982	772.99	199.19476	6.923	Decanamide, N,N-dimethyl-	C12H25NO	ION=[M+H]+		8103.38	9573.00	6951.00	12701.67
1983	773.21	233.08534				ION=[M+H]+		49942.77	10278.17	13578.33	53870.67
1984	773.7	786.49907				ION=[M+H+H]2+		1552.92	1205.83	3633.50	0.00
1985	775.13	663.45761				ION=[M+H]+		1812.62	612.00	337.00	2448.00

1986	776.72	170.09563	7.439	Tetrahydrofurfuryl methacrylate	C9H14O3	ION=[M+H]+		4416.92	4903.83	4474.50	6984.00
1987	777.34	716.30794				ION=[M+H]+		2845.08	756.83	0.00	440.00
1988	777.45	619.43082	22.358	Dragonamide C	C33H57N5O6	ION=[M+H]+		3018.62	1401.67	861.17	4429.00
1989	779.46	575.40383				ION=[M+H]+		3932.62	1793.67	1063.50	6651.00
1990	779.84	634.50558				ION=[M+H]+		9.85	213.33	2183.50	75.67
1991	780.16	195.58597				ION=[M+H]+		3620.77	1785.67	2002.00	3150.33
1992	781.45	682.5134				ION=[M+H+H]2+		1837.85	707.83	1333.33	2232.33
1993	781.97	364.23871	44.452	2-acyloxyethylphosphonate	C18H37O5P	ION=[M+H]+		1094.31	1048.50	3498.00	3056.67
1994	782.63	531.37752				ION=[M+H]+		4836.77	2061.50	1246.83	9145.33
1995	782.97	350.13997				ION=[M+H]+		5987.54	4869.83	4975.33	3709.67
1996	783.38	347.1323	9.123	N-Desmethyl ofloxacin	C17H18FN3O4	ION=[M+H]+	82419-52-1	5092.31	3265.00	2919.17	4048.33
1997	783.79	1274.64983				ION=[M+H+H]2+		8.15	68.50	789.50	4959.33
1998	783.84	391.17699				ION=[M+H+H]2+		3182.00	2650.33	2794.50	0.00
1999	784.36	626.38706				ION=[M+H]+		1166.62	1139.50	1177.67	0.00
2000	784.86	1220.73116				ION=[M+H+H]2+		9.08	423.67	8443.67	39624.33
2001	785.02	644.36023				ION=[M+H]+		7715.38	4022.00	2521.50	4524.33
2002	785.13	409.17511				ION=[M+H+H]2+		3229.69	2476.00	2065.83	1958.00
2003	785.13	414.18425				ION=[M+H+H]2+		3739.23	2890.50	2506.00	2306.00
2004	785.31	487.34966				ION=[M+H]+		6359.38	2195.83	1012.50	12437.67
2005	785.35	605.33081				ION=[M+H]+		3797.69	1411.00	1089.67	3133.67
2006	785.36	552.41819	5.757	Merocyclophane A	C36H56O4	ION=[M+H]+		14478.15	4870.50	2758.67	15528.00
2007	785.51	154.0995	6.373	2-Norbornaneacetic acid	C9H14O2	ION=[M+H]+		498.00	3871.00	297.33	489.67
2008	785.81	558.38832	19.964	2-(Tetradecanoylamino)-3-methylbutyric acid 2-deoxy-2-(butyrylamino)-alpha-D-glucopyranosyl ester	C29H54N2O8	ION=[M+H]+		281.69	1626.50	68.67	127.00
2009	785.84	610.41839				ION=[M+H]+		2076.00	1156.83	195.33	0.00
2010	786.13	593.44409				ION=[M+H]+		3033.69	1010.00	386.83	3179.67
2011	787.7	443.32524				ION=[M+H]+		8136.00	3142.17	1516.17	17071.33
2012	787.71	448.28007	16.016	(E)-lowdenic acid	C26H40O6	ION=[M+H]+		3442.46	2644.50	2449.17	4756.00
2013	789.24	664.39119				ION=[M+H+H]2+		418.92	0.00	0.00	0.00
2014	789.63	690.25838				ION=[M+H]+		1259.69	190.33	0.00	22.67
2015	790.03	300.16963	18.221	3-amino-4-(5,8,11-trioxa-1-azatetradecan-1-yl)cyclobut-3-ene-1,2-dione	C14H24N2O5	ION=[M+H]+		1674.15	776.17	1025.17	287.33
2016	790.65	399.29778				ION=[M+H]+		6903.08	2801.83	1518.67	16832.67
2017	792.03	672.53186				ION=[M+H+H]2+		0.00	0.00	1031.67	0.00
2018	792.17	262.22924	13.277	Phenol, 4-dodecyl-	C18H30O	ION=[M+H]+		4103.85	878.50	6384.50	9793.67
2019	792.56	628.37291				ION=[M+H+H]2+		141.38	496.67	837.00	0.00
2020	792.72	533.35632				ION=[M+H]+		4167.54	472.83	5032.67	6589.67
2021	792.8	1234.7457				ION=[M+H+H]2+		0.00	189.83	4129.67	16444.67
2022	793.17	572.24584				ION=[M+H+H]2+		1663.38	614.50	4008.50	2896.00
2023	793.8	226.12115	18.429	1,4-Butanediol dimethacrylate	C12H18O4	ION=[M+H]+	2082-81-7	1415.23	1007.33	789.33	2941.00
2024	793.86	708.55403				ION=[M+H+H]2+		2939.23	0.00	0.00	0.00
2025	794.22	227.22581				ION=[M+H]+		2147.23	2110.17	2182.83	0.00
2026	794.23	355.27304	19.337	(Z)-N-16-hydroxyhexadec-9-enoyl-L-alanine methyl ester	C20H37NO4	ION=[M+H]+		4770.31	1532.83	2345.67	11691.33
2027	794.41	617.37118				ION=[M+H]+		0.00	270.33	506.67	0.00
2028	794.78	686.2954				ION=[M+H]+		1913.85	545.83	0.00	21.67
2029	795.25	218.16828	8.6	Anaephene A	C15H22O	ION=[M+H]+		5041.08	2623.00	2473.17	7894.00
2030	799.8	234.16126				ION=[M+H]+		2486.62	2293.33	2174.17	3670.33
2031	801.12	501.32966				ION=[M+H]+		921.85	346.33	140.33	1956.67
2032	801.55	521.34847				ION=[M+H]+		5235.08	948.33	4185.00	4405.67
2033	802.97	162.0686	8.309	4-hydroxy-7-methyl-1-indanone	C10H10O2	ION=[M+H]+		11671.38	12398.17	12936.83	24899.67
2034	803.83	706.27851				ION=[M+H]+		3474.00	4322.33	4077.50	8031.00
2035	804.05	359.17318	18.487	2-({2-[(3r)-3-aminopiperidin-1-yl]-4-oxoquizolin-3(4h)-yl}methyl)benzonitrile	C21H21N5O	ION=[M+H]+	940907-93-7	2364.46	2239.17	2770.33	4850.33
2036	805.71	680.53563				ION=[M+H]+		2281.69	0.00	31.00	806.33
2037	809.8	296.03105				ION=[M+H+H]2+		19.69	1070.17	2153.50	0.00
2038	809.81	1218.75232				ION=[M+H+H]2+		0.00	266.33	4262.50	21125.33
2039	811.11	534.26433	17.571	SB236057A	C33H34N4O3	ION=[M+H]+		1122.62	523.33	151.67	152.33
2040	811.66	390.25491				ION=[M+H]+		2139.38	1196.83	3742.33	4379.67
2041	811.67	408.16054				ION=[M+H+H]2+		9809.85	2453.83	4830.67	0.00
2042	812.83	148.01608	3.525	1,3-Isobenzofurandione	C8H4O3	ION=[M+H]+		8023.69	6261.33	6592.17	20035.33
2043	813.03	670.27042				ION=[M+H]+		1476.31	222.00	49.67	185.00
2044	813.91	634.50426				ION=[M+H]+		8.92	540.33	1725.33	0.00
2045	814.83	204.08026	4.1	7-formyl-3-methoxy-5-methylindanone	C12H12O3	ION=[M+H]+		13118.00	14962.00	15858.33	34556.33
2046	815.29	672.28153				ION=[M+H]+		13738.15	1377.00	48.50	684.67
2047	815.84	264.20959	13.768	1H-3a,7-Methanoazulen-6-ol, octahydro-3,6,8,8-tetramethyl-, 6-acetate, (3R,3aS,6R,7R,8aS)-	C17H28O2	ION=[M+H]+		3186.62	1647.17	2175.33	1761.33
2048	818.04	1232.77015				ION=[M+H+H]2+		0.00	110.33	1898.83	6425.33
2049	819.22	294.63691				ION=[M+H]+		0.00	0.00	419.00	0.00
2050	819.72	352.1545	19.629	1,3-Dimethyl-N-[2-(1-pyrrolidinyl)-5-(trifluoromethyl)phenyl]-1H-pyrazole-5-carboxamide	C17H19F3N4O	ION=[M+H]+		2950.15	2228.17	1878.00	1692.67

2051	820.44	265.13494	NA	N-(2,6-Dimethylphenyl)-N-(methoxyacetyl) alanine	C14H19NO4	ION=[M+H]+		111.23	359.33	1915.83	0.00
2052	820.71	648.39268				ION=[M+H]+		3376.31	927.67	579.33	1061.67
2053	821.85	556.44953				ION=[M+H]+		4334.31	698.50	568.50	4588.33
2054	822.13	219.10579	5.607	1-Naphthalenamine, N-phenyl-	C16H13N	ION=[M+H]+		187801.38	75593.50	145066.17	248251.33
2055	823.57	834.66933				ION=[M+H+H2]3+		0.00	515.33	595.33	0.00
2056	824.38	498.23007				ION=[M+H+H]2+		0.00	0.00	0.00	1924.33
2057	825.56	249.11603				ION=[M+H]+		2867.69	1978.83	4542.33	11415.67
2058	826.94	1532.90662				ION=[M+H+H]2+		705.38	178.17	1698.00	3332.67
2059	827.43	222.05798				ION=[M+H]+		3911.23	4730.50	1340.17	4770.67
2060	827.44	296.07533				ION=[M+H]+		6137.08	7796.00	2159.83	6859.33
2061	827.93	166.04889				ION=[M+H]+		3144.15	3787.00	1051.67	4098.00
2062	828.52	509.35605	13.994	Chrysosporide	C26H47N5O5	ION=[M+H]+		9423.38	1199.00	9352.83	17866.00
2063	831.85	343.27312				ION=[M+H]+		351.08	1387.83	0.00	1030.67
2064	834.86	642.26871	5.885	8-hydroxythiomarinol C	C30H46N2O9S2	ION=[M+H]+		2384.62	1038.50	69.50	488.67
2065	835.01	742.46876				ION=[M+H]+		2283.69	415.83	6280.00	3747.67
2066	837.12	731.54278				ION=[M+H]+		2771.54	473.83	248.00	348.67
2067	839.43	692.46785	37.776	Veraguamide I	C37H64N4O8	ION=[M+H]+		1941.38	634.17	691.33	0.00
2068	840.04	522.406				ION=[M+H]+		0.00	0.00	0.00	4452.00
2069	840.23	687.51525				ION=[M+H]+		4614.62	323.17	260.50	526.33
2070	840.27	592.45809				ION=[M+H]+		1000.46	1065.83	1892.17	3292.33
2071	841.32	363.27733	18.997	11(12)-EET ethanolamide	C22H37NO3	ION=[M+H]+		24.31	19.17	1060.00	4074.33
2072	841.93	1296.88916				ION=[M+H+H]2+		768.92	214.67	272.33	0.00
2073	842.49	648.44161				ION=[M+H]+		2213.54	831.00	653.33	259.00
2074	843.02	643.48858				ION=[M+H]+		6350.62	330.00	315.50	341.33
2075	843.12	552.27557				ION=[M+H]+		982.77	223.67	0.00	25.33
2076	843.26	356.29227	5.906	Octadec-9-en-1-yl 2,3-dihydroxypropanoate	C21H40O4	ION=[M+H]+		4641.23	1463.33	7692.33	16064.67
2077	844.88	314.14949	18.276	2-(2-Hydroxy-2-propanyl)-9-(3-methyl-2-buten-1-yl)-2,3-dihydro-7H-furo[3,2-g]chromen-7-one	C19H22O4	ION=[M+H]+		884.31	1763.83	2462.83	0.00
2078	845.77	604.41652				ION=[M+H]+		3276.46	857.67	991.83	333.33
2079	846.03	218.16872	9.358	Anaephene A	C15H22O	ION=[M+H]+		2971.08	1620.83	3250.67	4812.67
2080	846.07	599.46144				ION=[M+H]+		8620.31	667.83	420.67	993.33
2081	846.5	338.24645				ION=[M+H]+		2213.85	2907.33	1389.67	3822.67
2082	849.26	555.43509				ION=[M+H]+		10747.54	647.00	596.50	1379.00
2083	849.61	796.51439				ION=[M+H]+		1206.77	554.50	2780.67	316.00
2084	850.5	267.07544	14.678	Methyl(2-acetoxy-2-(2-carboxy-4-amino-phenyl))acetate	C12H13NO6	ION=[M+H]+		9055.85	9721.67	8991.33	14793.33
2085	850.97	340.20389				ION=[M+H]+		2401.38	869.50	2365.17	5782.00
2086	852.25	640.28037				ION=[M+H]+		1505.85	244.17	221.17	492.00
2087	852.67	511.4086				ION=[M+H]+		13175.69	711.17	613.50	1366.00
2088	854.23	656.28376				ION=[M+H]+		5928.46	683.00	0.00	331.00
2089	854.37	328.15222	19.528	3-hydroxy-3-[(1,7,7-trimethyl-2-bicyclo[2.2.1]heptanyl)oxycarbonyl]pentanedioic acid	C16H24O7	ION=[M+H]+		1184.77	1092.17	1156.17	2352.00
2090	854.49	183.97814				ION=[M+H]+		3903.85	3960.17	3477.33	1464.00
2091	855.58	535.36295				ION=[M+H]+		1646.15	770.67	1638.50	1403.00
2092	856.58	668.28582				ION=[M+H]+		1852.31	276.50	60.67	103.67
2093	856.58	670.26928				ION=[M+H]+		2326.31	880.17	162.00	453.00
2094	856.91	467.38183				ION=[M+H]+		12808.46	631.50	615.67	1608.67
2095	860.73	423.35563				ION=[M+H]+		13319.54	700.33	723.00	1643.00
2096	861.48	598.41178				ION=[M+H]+		664.00	312.67	1995.33	1657.33
2097	865.51	379.32961				ION=[M+H]+		10824.46	662.83	585.00	1264.67
2098	866.72	582.42811	8.942	Hericene D	C37H58O5	ION=[M+H]+		1317.38	425.50	4499.50	3785.67
2099	867.89	336.2667				ION=[M+H]+		992.31	532.33	1995.50	647.33
2100	867.91	640.28697				ION=[M+H]+		2807.08	1256.33	1428.83	1570.67
2101	868.17	306.1132				ION=[M+H]+		2858.92	3969.50	1814.00	1884.00
2102	868.2	744.48499				ION=[M+H]+		3194.46	901.83	12006.67	9338.67
2103	868.33	312.26623				ION=[M+H]+		2072.77	1377.83	2787.67	593.00
2104	871.11	335.30382				ION=[M+H]+		6732.62	489.00	561.83	1188.67
2105	872.51	962.45616				ION=[M+H+H]2+		1611.54	342.33	3897.50	1362.67
2106	874.84	534.263	6.35	SB236057A	C33H34N4O3	ION=[M+H]+		79696.46	3396.17	14806.33	12275.33
2107	874.85	382.25055	13.156	Anhydroophiobolin A	C25H34O3	ION=[M+H]+		2414.46	1971.50	8278.67	8332.00
2108	876.01	752.60303				ION=[M+H]+		0.00	2076.33	0.00	0.00
2109	876.25	642.5205				ION=[M+H]+		0.00	1865.67	528.33	0.00
2110	877.52	770.50119				ION=[M+H]+		2625.54	725.50	6563.67	7930.67
2111	881.11	284.15229	18.192	Tropicamide	C17H20N2O2	ION=[M+H]+		3654.31	1246.67	251.33	1056.33
2112	881.49	292.24025	4.081	9(Z),11(E),13(E)-Octadecatrienoic Acid methyl ester	C19H32O2	ION=[M+H]+		16792.15	12477.00	12197.50	17753.00
2113	881.73	702.29243				ION=[M+H]+		1800.77	316.50	60.67	236.00
2114	883.14	756.34274				ION=[M+H+H]2+		890.77	65.67	2091.67	6458.67
2115	883.9	650.50063				ION=[M+H]+		0.00	4532.00	4581.83	139.33
2116	884.71	677.5015				ION=[M+H]+		129.85	1231.67	250.17	83.67
2117	891.21	830.44011				ION=[M+H+H]2+		2594.46	1333.00	4100.83	2733.67
2118	893.03	730.32239				ION=[M+H]+		1908.46	1054.33	32.00	371.67
2119	893.66	535.36374				ION=[M+H]+		970.46	20.83	2642.67	42.67

2120	894.25	665.53531				ION=[M+H]+		501.54	6842.00	1046.33	251.33
2121	894.74	658.26632				ION=[M+H]+		3342.62	767.33	88.33	259.67
2122	896.39	791.55891				ION=[M+H]+		1562.77	443.00	2042.83	1187.33
2123	900.52	166.04894				ION=[M+H]+		1580.46	2120.33	518.83	2272.33
2124	900.9	370.09462	2.611	Decamethylcyclopentasiloxane	C10H30O5Si5	ION=[M+H]+	541-02-6	2106.31	2998.83	796.17	3109.33
2125	900.9	462.12354				ION=[M+H]+		5815.85	7916.33	2024.17	7545.00
2126	901.08	463.12335				ION=[M+H]+		2706.62	3420.50	935.83	3638.67
2127	901.36	222.05791				ION=[M+H]+		1752.92	2301.33	658.33	2506.00
2128	901.37	296.07541				ION=[M+H]+		2470.15	3124.00	958.33	3832.33
2129	901.48	549.4103				ION=[M+H]+		2224.92	1465.00	1831.83	0.00
2130	901.73	592.15329				ION=[M+H+H]2+		23.85	433.00	15.33	270.33
2131	902.34	670.29329				ION=[M+H]+		1579.38	111.00	11.67	203.33
2132	902.37	458.43279				ION=[M+H]+		527.85	695.83	451.67	2590.33
2133	903.18	714.29492				ION=[M+H]+		4184.31	1732.50	79.33	1121.67
2134	903.6	746.50023				ION=[M+H]+		1796.31	784.83	5020.33	5374.33
2135	903.79	584.4435				ION=[M+H]+		1178.46	435.67	3316.17	3051.67
2136	904.82	279.25609	14.875	(2E)-2-Decylidene-5-(dimethylaminomethyl)cyclopentan-1-one	C18H33NO	ION=[M+H]+		4290.46	4314.33	4594.33	5578.00
2137	906.54	851.50311				ION=[M+H+H]2+		1341.38	731.67	1347.83	250.67
2138	907.08	675.48347				ION=[M+H]+		291.08	609.33	2549.67	284.00
2139	908.65	408.26513	19.378	Carbobenzyloxy-(l)-leucinyl-(l)leucinyl methoxymethylketone	C22H36N2O5	ION=[M+H]+		3824.15	1944.83	8035.83	6799.67
2140	909.48	258.19829	5.548	Cyclopenta[g]-2-benzopyran, 1,3,4,6,7,8-hexahydro-4,6,6,7,8,8-hexamethyl-	C18H26O	ION=[M+H]+		13687.23	3502.00	14765.67	28510.33
2141	909.53	656.53559				ION=[M+H]+		0.00	5870.17	1004.33	98.33
2142	909.86	964.46509				ION=[M+H+H]2+		1427.69	578.33	2824.00	1565.00
2143	910.24	609.17882				ION=[M+H]+		2360.92	997.33	643.00	1205.67
2144	915.57	816.52608				ION=[M+H+H]2+		624.46	43.00	809.83	0.00
2145	916.55	308.12542	9.979	1-[6-(2-chloro-4-methyxyphenoxy)-hexyl]-imidazole	C16H21ClN2O2	ION=[M+H]+		2651.23	2385.17	1317.50	1463.00
2146	916.57	419.32379				ION=[M+H]+		1522.00	3504.67	594.33	4565.33
2147	916.83	424.2793				ION=[M+H]+		3420.46	4214.83	1301.00	5842.33
2148	918.45	679.55146				ION=[M+H]+		610.62	5707.00	1860.17	439.00
2149	919.24	610.46004				ION=[M+H]+		1153.85	342.00	3586.67	4127.33
2150	919.26	586.27994	17.751	Phycoerythrobilin	C33H38N4O6	ION=[M+H]+		3018.00	1221.00	323.17	649.67
2151	919.32	671.52075				ION=[M+H]+		2620.77	332.33	359.00	341.33
2152	919.93	772.51658				ION=[M+H]+		1388.77	396.17	5651.67	6634.67
2153	920.41	335.31922				ION=[M+H]+		2981.54	2554.67	2200.33	3178.00
2154	920.59	310.25071				ION=[M+H]+		4822.15	5750.00	1404.50	2644.00
2155	920.74	269.2718	13.1	Bacillamidin G	C17H35NO	ION=[M+H]+		4180.62	4264.50	4015.83	667.67
2156	922.51	627.49231				ION=[M+H]+		3836.31	431.00	456.00	763.67
2157	922.86	770.32177				ION=[M+H+H]2+		12931.23	473.17	27656.33	53325.67
2158	922.95	738.29571				ION=[M+H+H]2+		2935.69	77.50	5789.83	11485.33
2159	923.41	351.31338				ION=[M+H]+		3635.08	4169.67	2799.17	3914.00
2160	923.74	405.2363				ION=[M+H]+		0.00	1613.67	197.17	52.33
2161	925.69	364.23995	40.029	2-acyloxyethylphosphonate	C18H37O5P	ION=[M+H]+		13809.23	3740.33	15918.00	37733.67
2162	925.93	591.49539				ION=[M+H]+		4077.08	3653.00	8288.00	0.00
2163	926.28	990.48408				ION=[M+H+H]2+		1654.62	494.83	4179.00	2066.67
2164	926.52	583.46598				ION=[M+H]+		4951.54	638.67	246.33	422.33
2165	927.1	1008.49136				ION=[M+H+H]2+		1868.92	520.17	4038.00	2197.00
2166	927.85	424.29712				ION=[M+H]+		0.00	3434.50	0.00	81.67
2167	927.93	2314.56297				ION=[M+H+H]2+		680.31	274.17	448.00	0.00
2168	928	336.26414				ION=[M+H]+		733.23	949.33	1820.00	1809.67
2169	930.31	544.39594				ION=[M+H]+		2820.00	981.17	1059.33	536.33
2170	930.45	539.4395				ION=[M+H]+		5702.46	615.83	552.17	888.00
2171	932.51	1294.87226				ION=[M+H+H]2+		730.77	366.00	617.83	464.67
2172	932.94	549.40987				ION=[M+H]+		1970.92	1470.83	1845.00	0.00
2173	933.78	425.24404				ION=[M+H]+		1655.54	1241.00	1430.33	0.00
2174	934.13	667.55062				ION=[M+H]+		905.23	5289.67	559.83	0.00
2175	934.64	714.32824				ION=[M+H]+		9511.69	6026.00	324.83	1596.67
2176	934.69	500.36929				ION=[M+H]+		3724.31	1198.17	1193.00	780.00
2177	934.78	495.41229				ION=[M+H]+		5490.77	643.50	508.83	1043.00
2178	935.31	614.27456				ION=[M+H]+		7266.92	4556.50	121.83	488.67
2179	935.56	425.7501				ION=[M+H]+		571.69	910.00	1520.17	691.67
2180	937.05	754.32787				ION=[M+H+H]2+		616.62	0.00	1766.67	5082.33
2181	937.96	970.50906				ION=[M+H+H]2+		1952.46	870.00	3179.00	165.00
2182	938.21	779.52081				ION=[M+H]+		946.15	89.00	2248.50	1651.67
2183	939.32	456.34221				ION=[M+H]+		4927.38	1356.67	1221.67	859.33
2184	939.34	451.38658				ION=[M+H]+		5483.54	492.17	502.50	937.33
2185	939.76	1126.51758				ION=[M+H+H]2+		1747.23	979.00	2813.33	946.67
2186	940.69	532.24649				ION=[M+H]+		1064.62	61.17	453.83	107.67
2187	940.99	434.06993				ION=[M+H]+		1552.15	1989.33	809.17	682.67
2188	941.06	334.14463	18.296	Combretastatin	C18H22O6	ION=[M+H]+	82855-09-2	8677.54	10371.67	5968.83	5488.67
2189	942.1	800.39512				ION=[M+H+H]2+		2977.69	636.50	5687.50	2608.33

2190	942.15	278.22445	18.499	Gamolenic acid	C18H30O2	ION=[M+H]+	506-26-3	6895.08	4646.00	5320.17	15266.67
2191	942.34	798.35262				ION=[M+H+H]2+		1336.31	96.00	5119.67	12726.00
2192	943.07	748.51307				ION=[M+H]+		995.23	342.83	2315.00	2386.67
2193	943.26	798.36576				ION=[M+H]+		150.46	0.00	1200.33	3242.33
2194	943.57	766.32678				ION=[M+H+H]2+		570.46	14.50	1764.33	3943.33
2195	944.48	407.36027				ION=[M+H]+		5106.15	399.83	431.00	840.00
2196	946.01	650.49549				ION=[M+H]+		0.00	905.17	453.17	0.00
2197	947.07	343.27241				ION=[M+H]+		1424.92	139.00	577.67	1400.00
2198	947.34	624.29507	13.919	Methyl n-[1-({6-hydroxy-5-[n-(2-methylpropyl)4-aminobenzenesulfonamido]hexyl}carbamoyl)-2,2-diphenylethyl]carbamate	C33H44N4O6S	ION=[M+H]+	612547-11-2	15189.54	8817.67	681.50	2752.00
2199	947.67	589.4389				ION=[M+H]+		1702.92	274.17	252.67	277.33
2200	948.08	283.28734	12.034	octadecanamide	C18H37NO	ION=[M+H]+	124-26-5	5162.92	5418.83	5505.67	9212.00
2201	948.17	582.42851				ION=[M+H]+		2944.92	509.33	8846.67	4641.33
2202	948.66	826.34684				ION=[M+H+H]2+		4810.77	285.83	10927.00	19843.00
2203	950.07	780.42672				ION=[M+H+H]2+		1205.85	191.33	713.33	0.00
2204	950.08	390.255				ION=[M+H]+		15821.38	4058.50	16409.83	39850.00
2205	950.11	826.35744				ION=[M+H]+		1017.08	41.67	1764.83	3408.67
2206	950.98	1064.67312				ION=[M+H+H]2+		1756.15	1692.00	1148.00	884.00
2207	951.24	363.33456				ION=[M+H]+		3478.46	441.33	299.50	780.67
2208	952.6	608.26501				ION=[M+H]+		4733.08	1013.50	2446.67	3700.00
2209	952.87	647.41874				ION=[M+H]+		400.77	4457.00	65.67	292.67
2210	953.53	640.2574				ION=[M+H]+		2269.38	369.00	1283.00	554.67
2211	953.82	656.24458				ION=[M+H]+		1736.31	974.00	0.00	53.33
2212	954.04	654.27416				ION=[M+H]+		6137.69	1302.50	731.17	5669.33
2213	954.8	773.25041				ION=[M+H]+		422.46	13.17	0.00	0.00
2214	956.49	255.2566	8.251	Hexadecanamide	C16H33NO	ION=[M+H]+		12764.92	12211.83	13422.33	11984.33
2215	956.6	549.62173				ION=[M+H]+		332.46	1742.67	2109.67	0.00
2216	964.23	281.27153	12.538	Elaidoylamide	C18H35NO	ION=[M+H]+	4303-70-2	48155.85	49317.83	47679.33	59464.00
2217	965.05	1134.58009				ION=[M+H+H]2+		1433.85	839.17	2228.67	0.00
2218	967.98	970.50879				ION=[M+H+H]2+		1999.38	3298.67	6662.50	1176.00
2219	32.94	175.96519				ION=[M-H2O+H]+		15259.85	14782.00	14200.83	11987.33
2220	33.24	203.96087				ION=[M-H2O+H]+		15312.77	14876.00	13579.50	10650.67
2221	35.38	253.94252				ION=[M+H]+		5274.62	4570.67	4641.83	1626.67
2222	36.42	181.97864				ION=[M+H]+		4404.46	4037.00	4933.83	1947.67
2223	34.05	158.95924				ION=[M+H]+		3166.77	2781.83	2972.83	1398.00
2224	36.14	140.95084				ION=[M+H]+		3054.92	3474.83	3533.33	1690.00
2225	39.38	357.3098				ION=[M+H+H]2+		0.00	39.50	44.33	4603.67
2226	38.81	293.2802				ION=[M+H+H]2+		0.00	0.00	5.33	136846.00
2227	40.68	297.11556				ION=[M+H+H]2+		3958.77	11348.17	0.00	0.00
2229	41.98	329.14244				ION=[M+H+H]2+		2777.08	6700.33	0.00	0.00
2230	40.12	343.29476				ION=[M+H]+		0.00	4046.17	0.00	0.00
2232	40.51	300.28661				ION=[M+H+H]2+		40.00	0.00	30830.67	27.00
2233	40.39	269.24347				ION=[M+H+H]2+		92.15	0.00	13004.50	0.00
2234	40.45	353.08582				ION=[M+H]+		1150.46	2885.67	0.00	19.67
2235	43.19	240.99318				ION=[M+H]+		7949.23	12373.83	17211.00	75.00
2236	43.25	128.04826				ION=[M+Na]+		4838.92	7121.67	8593.33	426.00
2237	44.21	215.94469				ION=[M+H]+		2192.15	1644.67	3039.83	0.00
2238	44.15	256.97061				ION=[M+H]+		3343.69	2398.00	5149.00	0.00
2239	42.29	168.03984				ION=[M+H]+		1955.85	2846.17	2955.67	98.33
2240	44.38	238.95935				ION=[M+H]+		1724.92	1470.67	2958.00	0.00
2241	44.11	272.94556				ION=[M+H]+		1622.15	1801.50	2074.50	12.67
2242	43.18	199.96667				ION=[M+H]+		696.46	1350.17	2001.67	0.00
2243	43.22	217.97732				ION=[M+H]+		1296.77	2092.50	2213.00	0.00
2244	41.65	334.2399				ION=[M+H+H]2+		234.62	0.00	7749.50	0.00
2245	43.85	269.2468				ION=[M+H]+		7.38	14.67	4399.00	430.67
2246	43	264.00996				ION=[M+H]+		2765.69	4914.17	6724.50	0.00
2247	44.2	831.38382				ION=[M+H+H2]3+		96.77	15130.50	1124.17	66.00
2248	44.07	342.32967				ION=[M+H+H2]3+		2528.31	0.00	20.67	89.33
2249	43.87	269.11319				ION=[M+H]+		1297.08	3369.83	16.17	46.00
2250	43.1	297.11673				ION=[M+H]+		1604.00	4543.67	0.00	0.00
2251	43.75	329.1446				ION=[M+H]+		2039.69	5646.83	16.67	7.67
2252	41.52	376.28733				ION=[M+H+H]2+		5747.85	0.00	47.33	0.00
2253	41.63	360.29147				ION=[M+H+H]2+		5940.92	0.00	138.83	0.00
2254	41.88	357.31049				ION=[M+H]+		0.00	1195.33	350.67	4698.33
2255	42.21	376.28894				ION=[M+H]+		17525.08	0.00	0.00	0.00
2256	42.63	334.24175				ION=[M+H]+		597.08	0.00	12774.67	304.67
2257	43.99	311.2913				ION=[M+H+H]2+		115775.38	29.83	2152.83	1124.67
2258	43.5	328.31827				ION=[M+H+H]2+		3370.92	0.00	5677.17	0.00
2259	44.31	342.33447				ION=[M+H+H]2+		183721.69	400.17	10534.17	501.67
2260	43.57	293.2831				ION=[M+H]+		0.00	0.00	0.00	666952.67
2261	42.77	291.26808				ION=[M+H]+		0.00	0.00	5.83	4863.33
2262	43.39	360.29266				ION=[M+H]+		9738.15	0.00	194.50	22.00

2263	45.2	225.22182	8.067	N1-(2-amino-4-methylpentyl)octahydro-pyrrolo[1	C13H27N3	ION=[M+H]+		0.00	17.83	0.00	53524.67
2264	45.32	338.24492				ION=[M+H]+		52.77	0.00	17025.33	0.00
2265	44.49	560.26314				ION=[M+H+H2]3+		158.00	7700.83	935.67	0.00
2266	44.4	831.38857				ION=[M+H+H]2+		48.92	3482.00	337.00	18.33
2267	44.9	560.2615				ION=[M+H+H]2+		0.00	7986.67	471.17	0.00
2268	43.6	514.25821				ION=[M+H+H2]3+		0.00	1159.17	0.00	0.00
2269	44.11	231.92133				ION=[M+H]+		1306.46	662.33	1009.33	0.00
2270	43.82	124.97813				ION=[M+H]+		2982.46	2158.83	3241.00	29.67
2271	43.99	315.26478				ION=[M+H]+		0.00	0.00	0.00	6407.33
2272	44.4	239.23729				ION=[M+H]+		0.00	11.67	0.00	5466.00
2273	46.31	342.33567				ION=[M+H]+		443444.00	760.50	23098.33	1833.33
2274	45.92	328.3204				ION=[M+H]+		15571.85	109.33	29905.67	19.67
2275	45.95	309.27876				ION=[M+H]+		41.08	5.83	3.17	7545.00
2276	44.11	233.95491				ION=[M+H]+		482.15	0.00	900.83	0.00
2277	44.4	280.17726	NA	Mefexamide	C15H24N2O3	ION=[M+H]+	1227-61-8	0.00	1821.67	132.83	20.33
2278	44.7	322.27105				ION=[M+H]+		0.00	0.00	20501.50	0.00
2279	45.09	319.86132				ION=[M+H]+		4447.08	5316.83	6278.50	2393.67
2280	47.35	300.2884				ION=[M+H]+		1744.31	59.33	472928.67	118.00
2281	47.57	407.91182				ION=[M+H]+		740.77	1786.83	1176.83	0.00
2282	46.53	257.24694				ION=[M+H]+		83630.31	25031.50	4658.33	1501.33
2283	47.17	271.93695				ION=[M+H]+		2199.54	4070.17	3356.50	157.00
2284	49	203.96334				ION=[M+K]+		10596.15	18474.50	22087.67	9177.67
2285	48.38	265.93178				ION=[M+Na]+		5057.38	7016.83	7600.33	447.67
2286	48.62	135.97449				ION=[M+Na]+		2310.92	3645.67	3693.00	174.00
2287	49	355.91154				ION=[M+Na]+		1714.15	3421.83	3362.17	639.67
2288	48.99	423.89913				ION=[M+Na]+		1587.08	3694.83	2964.83	83.67
2289	48.46	401.90592				ION=[M+Na]+		1906.92	3441.17	2938.67	20.00
2290	49.18	491.88643				ION=[M+Na]+		997.38	2243.17	1553.33	0.00
2291	48.09	469.8942				ION=[M+Na]+		940.92	2067.67	1681.17	0.00
2292	48.44	537.88163				ION=[M+K]+		973.08	1765.50	1969.50	0.00
2293	48.98	559.87508				ION=[M+Na]+		847.85	2028.17	1233.00	0.00
2294	48.84	605.86853				ION=[M+K]+		993.38	1594.67	1795.33	0.00
2295	49.57	627.86186				ION=[M+K]+		652.77	1503.50	1382.00	0.00
2296	48.28	455.83646				ION=[M+H]+		1299.85	1604.00	2919.00	585.67
2297	48.61	361.91857				ION=[M+H]+		902.00	2242.00	1217.33	0.00
2298	48.42	429.90623				ION=[M+H]+		1063.23	2369.33	1184.67	0.00
2299	50.48	309.90462				ION=[M+H]+		777.23	1531.00	1665.33	607.33
2300	48.83	355.90057				ION=[M+H]+		1380.15	2552.17	2521.00	62.33
2301	48.44	371.87576				ION=[M+H]+		1308.62	1912.17	2633.17	445.33
2302	47.63	560.26688				ION=[M+H]+		0.00	1962.67	336.00	0.00
2303	48.03	945.5674				ION=[M+H]+		1259.08	3948.00	315.50	978.67
2304	48.6	797.69888				ION=[M+H]+		531.23	0.00	3543.00	6091.33
2305	48.7	713.73901				ION=[M+H]+		774.77	0.00	3255.50	5696.33
2306	48.81	645.75393				ION=[M+H]+		2438.46	1242.50	5295.33	9675.33
2307	48.92	729.71456				ION=[M+H]+		2729.54	756.67	5485.33	9847.00
2308	49.14	897.63993				ION=[M+H]+		2452.00	235.17	3495.83	7079.00
2309	49.38	577.76572				ION=[M+H]+		4836.46	3318.17	7496.33	13982.33
2310	49.77	257.89253				ION=[M+H]+		16780.77	17356.50	34675.00	40136.67
2311	49.17	409.84143				ION=[M+H]+		3558.00	2843.00	8491.67	12072.33
2312	49.66	493.80318				ION=[M+H]+		5638.62	3575.67	11025.33	19040.00
2313	49.22	881.66211				ION=[M+H]+		650.00	0.00	3064.50	5714.33
2314	49.33	813.67569				ION=[M+H]+		2162.00	37.67	4580.50	8578.67
2315	49.34	913.6142				ION=[M+H]+		3432.92	1598.50	3112.83	6291.00
2316	49.36	477.82917				ION=[M+H]+		2028.62	1778.83	6568.67	7424.33
2317	49.6	745.68931				ION=[M+H]+		5109.23	4114.50	6283.50	12670.67
2318	49.38	981.60142				ION=[M+H]+		583.08	277.67	2235.33	5027.33
2319	49.38	561.79156				ION=[M+H]+		1767.23	1019.00	5277.67	8047.33
2320	49.88	503.76952				ION=[M+Na]+		18462.46	24581.17	11329.67	24427.33
2321	49.75	419.80732				ION=[M+Na]+		15876.00	19225.33	11329.33	24976.00
2322	49.88	335.84622				ION=[M+Na]+		12864.31	14875.67	11278.83	22511.67
2323	49.65	655.72114				ION=[M+Na]+		8140.31	8796.67	6635.67	13854.67
2324	50.09	587.73241				ION=[M+Na]+		8342.31	12378.33	4477.83	10084.67
2325	51.95	370.36723				ION=[M+H]+		0.00	14371.17	0.00	0.00
2326	51.5	376.31668				ION=[M+H]+		22821.23	1854.00	0.00	0.00
2327	49.85	929.59032				ION=[M+H]+		3362.46	4126.83	1581.67	4073.00
2328	49.88	289.84007				ION=[M+H]+		76427.85	103747.83	50565.00	108318.00
2329	50.06	845.62789				ION=[M+H]+		4856.31	5889.67	3107.17	6690.67
2330	49.47	793.61511				ION=[M+H]+		881.08	3817.33	184.33	717.67
2331	49.95	167.92409				ION=[M+K]+		49384.77	66322.17	32905.17	67821.67
2332	49.83	739.68374				ION=[M+Na]+		6699.54	7445.67	4606.33	9762.67
2333	49.79	661.72791				ION=[M+H]+		5515.38	4059.50	7624.33	14451.33
2334	50.31	709.65207				ION=[M+H]+		2761.38	5662.33	690.50	2011.00
2335	50	629.77882				ION=[M+H]+		778.31	64.33	2970.83	4472.67

2336	50.08	861.60217				ION=[M+H]+		3295.08	5724.67	544.17	1666.33
2337	49.67	341.85421				ION=[M+H]+		5124.92	3950.17	7699.67	12868.00
2338	49.8	965.62283				ION=[M+H]+		366.31	0.00	2587.00	4822.33
2339	50.02	425.81541				ION=[M+H]+		10089.38	8284.83	12541.50	25134.33
2340	49.78	273.86616				ION=[M+H]+		50890.92	50801.67	58947.83	104341.67
2341	49.62	829.65188				ION=[M+H]+		4298.46	3727.17	4758.83	9456.00
2342	49.95	509.77731				ION=[M+H]+		13123.54	12002.00	14335.33	29027.67
2343	49.85	593.73992				ION=[M+H]+		8372.62	8143.17	7142.67	15386.00
2344	51.41	539.18252				ION=[M+H]+		803.69	3919.83	3344.50	712.33
2345	51.61	376.31676				ION=[M+H+H]2+		9070.62	1502.17	317.83	648.00
2346	52.98	404.35099				ION=[M+H+H]2+		0.00	64675.50	52.50	39.67
2347	52.8	390.33534				ION=[M+H]+		14.62	28690.67	38.00	24.00
2348	52.71	476.30193				ION=[M+H]+		18036.31	0.00	0.00	0.00
2349	52.43	356.35134				ION=[M+H]+		1611.08	4863.83	0.00	0.00
2350	54.77	244.94779				ION=[M+H]+		1760.00	1029.83	3502.67	4638.67
2351	52.86	305.24673				ION=[M+H]+		0.00	13175.50	18.50	237.33
2352	52.96	205.13303	5.879	Trapidil	C10H15N5	ION=[M+H]+	15421-84-8	0.00	15570.33	0.00	305.33
2353	52.88	243.23145				ION=[M+H]+		437.54	3271.33	838.50	882.33
2354	53.35	390.17344				ION=[M+Na]+		3147.23	3075.67	4572.67	2125.00
2355	51.89	254.13843	8.775	Pimonidazole	C11H18N4O3	ION=[M+H]+	70132-50-2	999.85	0.00	4188.50	1218.67
2356	52.05	370.36639				ION=[M+H+H]2+		0.00	12524.50	0.00	0.00
2357	53.61	928.28501				ION=[M+H+H2]3+		2098.77	1794.83	3915.33	216.67
2358	53.79	928.29112				ION=[M+H+H]2+		2893.54	2231.33	6400.33	984.33
2359	54.39	675.28802				ION=[M+H+H]2+		106.15	2835.50	802.00	0.00
2360	54.03	675.29021				ION=[M+H+H2]3+		0.00	1475.33	418.67	0.00
2361	54.44	172.08141	NA	Glycylproline	C7H12N2O3	ION=[M+H]+		391.23	1148.00	0.00	25.00
2362	54.45	578.27559				ION=[M+H+H]2+		592.15	580.00	921.67	0.00
2363	54.97	659.37042				ION=[M+H]+		0.00	0.00	0.00	11738.67
2364	55.28	185.01085				ION=[M+K]+		3113.54	2338.17	5670.17	7676.33
2365	55.05	420.34624				ION=[M+H]+		0.00	5382.83	0.00	0.00
2366	55.39	393.29991				ION=[M+H]+		0.00	21.33	4.17	4784.00
2367	55.15	659.36328				ION=[M+H+H]2+		5.38	10.67	0.00	84568.67
2368	55.21	517.20117				ION=[M+H]+		4979.08	18916.83	16882.00	6036.67
2369	55.25	217.11197	NA	Glutethimide	C13H15NO2	ION=[M+H]+	77-21-4	3121.38	1073.67	1803.50	868.33
2370	56.76	461.21168				ION=[M+H]+		2416.92	2844.17	2882.83	8064.67
2371	55.89	514.12043				ION=[M+H]+		1349.85	210.00	1525.67	3931.00
2372	55.3	187.12121				ION=[M+H]+		68.77	44.50	20.50	15361.00
2373	55.3	659.3634				ION=[M+H+H2]3+		0.00	0.00	0.00	44835.67
2374	55.58	256.04758	NA	3-Hydroxy-1-[3-(trifluoromethyl)phenyl]pyrazin-2(1H)-one	C11H7F3N2O2	ION=[M+H]+		2290.46	157.83	1899.83	3395.33
2375	56.14	204.07806	NA	7-formyl-3-methoxy-5-methylindanone	C12H12O3	ION=[M+H]+		2244.15	1447.17	1240.00	219.33
2376	55.83	111.04273	11.121	Imexon	C4H5N3O	ION=[M+H]+	59643-91-3	235.23	891.83	88.67	70.00
2377	56.33	390.17422	5.078	AS-I toxin	C15H26N4O8	ION=[M+H+H]2+		30411.85	30178.33	41851.67	16302.33
2378	55.35	833.37155				ION=[M+H+H]2+		1572.77	941.17	4624.17	2027.00
2379	55.35	833.36722				ION=[M+H+H2]3+		1012.77	1692.67	6124.50	2067.33
2380	55.62	390.1751	6.709	AS-I toxin	C15H26N4O8	ION=[M+H]+		16266.31	12725.83	21207.50	17211.33
2381	56.3	190.09755	3.436	Dicyclanil	C8H10N6	ION=[M+H]+	112636-83-6	16409.23	6915.00	11160.67	4926.33
2382	56.02	386.15578				ION=[M+H+H]2+		21.54	1528.00	504.00	0.00
2383	56.11	643.36846				ION=[M+H+H]2+		16.31	0.00	16.83	10620.33
2384	56.34	643.36914				ION=[M+H+H2]3+		0.00	31.83	0.00	6095.00
2385	56.11	393.29926				ION=[M+H+H]2+		7.54	0.00	0.00	12525.00
2386	56.16	257.24783				ION=[M+H]+		0.00	4377.00	0.00	0.00
2387	56.73	185.01085				ION=[M+H]+		1075.85	572.00	2210.67	3124.33
2388	56.51	454.17991				ION=[M+K]+		13829.08	2030.33	11890.00	38182.67
2389	56.69	271.26236				ION=[M+H]+		23.08	74077.17	14.33	17.00
2390	58.83	292.0567				ION=[M+H]+		23781.85	23675.67	5411.50	47657.67
2391	58.5	229.10599	NA	2-Pyridinecarbaldehyde N-(4-amino-6-methyl-1,3,5-triazin-2-yl)hydrazone	C10H11N7	ION=[M+H]+		2974.92	902.33	1254.50	1924.33
2392	58.41	343.28457				ION=[M+H]+		6209.08	88.50	0.00	0.00
2393	58.81	436.16856	23.868	Hexose-palythine-serine	C17H28N2O11	ION=[M+H]+		7781.85	6181.83	11735.00	14256.33
2394	57.93	171.12604				ION=[M+H]+		0.00	0.00	0.00	11969.67
2395	56.58	890.35538				ION=[M+H]+		1879.23	1872.00	5466.33	2534.00
2396	56.69	200.08215	NA	Bis(4-hydroxyphenyl)methane	C13H12O2	ION=[M+H]+		3204.15	1666.33	3188.50	1660.00
2397	57.23	218.08867	12.942	4-[2-(3,5-diamino-1h-pyrazol-4-yl)diazen-1-yl]phenol	C9H10N6O	ION=[M+H]+		60222.31	20492.50	50255.83	0.00
2398	56.71	461.21051				ION=[M+H+H]2+		2826.46	20037.33	13847.50	21200.00
2399	57.01	231.0538				ION=[M+H]+		34.46	3635.00	451.00	0.00
2400	57.52	454.11034				ION=[M+H]+		6341.38	3308.33	1277.67	15225.00
2401	57.04	271.2587				ION=[M+H+H]2+		0.00	3216.50	32.50	0.00
2402	57.54	261.13228	NA	Methapyrilene	C14H19N3S	ION=[M+H]+		3119.69	2014.00	2537.50	2145.33
2403	60.35	145.08457	NA	4-Guanidinobutyric acid	C5H11N3O2	ION=[M+H]+		775.85	2791.67	314.67	40.00
2404	57.83	438.13618				ION=[M+H]+		5015.38	7207.50	1572.50	6701.33
2405	59	213.11291				ION=[M+H]+		3936.92	1649.33	9861.50	3031.00
2406	59	262.16398				ION=[M+H]+		0.00	3407.67	0.00	0.00

2407	58.67	342.11684	7.978	Sucrose	C12H22O11	ION=[M+K]+		7146.92	4247.67	7136.33	31449.67
2408	58.51	251.10058	NA	Muramic acid	C9H17NO7	ION=[M+H]+		3831.54	3385.33	3943.83	555.67
2409	57.88	193.09674	11.027	(2R)-1-(6-Amino-9H-purin-9-yl)propan-2-ol	C8H11N5O	ION=[M+H]+		61.23	14323.00	199.83	0.00
2410	58.75	293.03224				ION=[M+H]+		0.00	0.00	2675.50	0.00
2411	58.72	169.11126				ION=[M+H]+		0.00	0.00	0.00	24485.33
2412	58.92	291.23126				ION=[M+H]+		2011.23	10642.33	0.00	0.00
2413	58.12	243.12416	NA	2-(4-Methoxybenzylidene)quinuclidin-3-one	C15H17NO2	ION=[M+H]+		2352.77	1026.50	2052.33	579.33
2414	58.17	234.08652	NA	(2R)-5-Methoxy-2-methyl-2,3,8,9-tetrahydro-4H-furo[2,3-h]chromen-4-one	C13H14O4	ION=[M+H]+		3023.08	1726.17	2704.00	1578.33
2415	58.31	343.28493				ION=[M+H+H]2+		4424.62	37.50	0.00	0.00
2416	59.78	283.03974				ION=[M+H]+		1283.38	965.50	4050.33	1956.67
2417	58.36	205.09655	12.456	6-Morpholinopurine	C9H11N5O	ION=[M+H]+	2846-96-0	167.23	11182.00	115.00	0.00
2418	58.36	203.09217	NA	Norfenfluramine	C10H12F3N	ION=[M+H]+		1126.62	0.00	343.17	106.33
2419	59.26	454.17868	17.813	Curvularin-7-O-Œ≤-D-glucopyranoside	C22H30O10	ION=[M+H]+		127332.77	85082.50	185302.83	176881.67
2420	60.15	472.19227	12.379	Methyl 2-hydroxy-4-{3-[(4-oxo-3-phenyl-4H-chromen-7-yl)oxy]propoxy}-3-propylbenzoate	C29H28O6	ION=[M+H]+		2468.62	2666.33	430.83	1390.33
2421	61.32	573.2281				ION=[M+H]+		3996.31	332.67	8120.50	0.00
2422	60.07	186.10147				ION=[M+H]+		5002.77	1076.50	1776.83	3333.33
2423	61.18	416.15357				ION=[M+H]+		2898.15	3995.33	1061.00	2144.00
2424	60.6	246.06445				ION=[M+H]+		909.08	0.00	0.00	0.00
2425	59.21	147.05312	2.035	N-Acetylserine	C5H9NO4	ION=[M+H]+		17897.85	20667.17	37014.50	34148.33
2426	59.36	227.0892				ION=[M+H]+		2720.77	3460.00	7518.83	5464.33
2427	60.33	330.09399				ION=[M+H+H]2+		1855.38	711.17	1686.50	1473.00
2428	60.95	183.08357				ION=[M+H]+		1564.77	837.50	4738.67	1428.00
2429	59.39	257.10154				ION=[M+H]+		4757.38	1770.67	7227.17	898.67
2430	58.63	201.10562				ION=[M+H]+		1009.54	62.50	229.50	0.00
2431	58.65	216.14259				ION=[M+H]+		0.00	0.00	0.00	3662.67
2432	60.84	305.24543				ION=[M+H]+		0.00	30994.00	7.83	145.67
2433	58.88	294.1066				ION=[M+H+H]2+		3504.62	496.00	0.00	0.00
2434	58.9	908.36503				ION=[M+H]+		617.23	458.00	2128.00	2565.00
2435	60.63	624.17952				ION=[M+H]+		1813.08	418.00	1705.83	5610.00
2436	60.69	259.04677	19.847	Glucosamine 6-phosphate	C6H14NO8P	ION=[M+H]+		4890.31	642.83	2964.67	8918.00
2437	59.8	659.3639				ION=[M+H+H]2+		0.00	0.00	0.00	13687.67
2438	59.08	229.07428	NA	Tebamin	C13H11NO3	ION=[M+H]+	133-11-9	1351.38	0.00	0.00	0.00
2439	60.07	208.04152				ION=[M+H]+		2363.85	1417.83	1302.67	1492.67
2440	60.35	244.07512				ION=[M+H]+		1070.77	692.00	5037.33	632.67
2441	60.47	276.08553	9.327	N-(1,3-Benzodioxol-5-ylmethyl)-1-methyl-4-nitro-1H-imidazol-5-amine	C12H12N4O4	ION=[M+H]+		14626.62	21191.00	2401.17	15710.00
2442	62.28	162.05322	10.988	3-Hydroxy-3-methylglutaric acid	C6H10O5	ION=[M+H]+		7944.15	10646.00	3390.83	7757.67
2443	61.95	254.10041	NA	Diprophylline	C10H14N4O4	ION=[M+H]+	479-18-5	10613.54	18097.17	3570.17	8497.00
2444	61.45	123.03146	3.933	Nicotinic acid	C6H5NO2	ION=[M+H]+		14251.08	10064.83	28351.67	19790.67
2445	61.26	255.07461	4.269	N2-[3-(Trifluoromethyl)phenyl]-1,3,5-triazine-2,4-diamine	C10H8F3N5	ION=[M+H]+		60465.23	48548.33	124070.67	76864.33
2446	60.6	426.08097				ION=[M+H]+		1312.92	853.33	1549.67	1690.67
2447	60.89	428.33486				ION=[M+H+H]2+		8950.77	0.00	0.00	312.00
2448	62.64	229.08982				ION=[M+H]+		6952.77	3984.50	22531.50	6606.33
2449	61.37	252.09837	47.054	Guanitoxin	C7H17N4O4P	ION=[M+H]+		2153.69	3237.67	370.00	0.00
2450	62.45	259.15307				ION=[M+H]+		4728.31	1318.67	2519.50	7624.67
2451	62.37	238.16799	15.428	1,5-Ditetrahydro-1H-pyrrol-1-ylpentane-1,5-dione	C13H22N2O2	ION=[M+H]+		0.00	0.00	0.00	4845.00
2452	63.01	232.14194	12.408	(+)-Costunolide	C15H20O2	ION=[M+H]+	553-21-9	6938.15	1508.50	3482.50	9510.00
2453	60.75	219.11137	5.597	trans-Zeatin	C10H13N5O	ION=[M+H]+		0.00	2604.50	0.00	218.67
2454	62.54	201.09574				ION=[M+H]+		3606.77	1914.50	11477.67	3315.67
2455	62.19	245.08413	9.909	5-Fluoro-2'-deoxycytidine	C9H12FN3O4	ION=[M+H]+		33962.77	15172.33	109445.17	25360.00
2456	62.6	221.09206	17.86	N-Acetyl-D-galactosamine	C8H15NO6	ION=[M+H]+		6827.08	3326.17	9127.17	8102.33
2457	62.64	275.10732	NA	N-Benzyl-N-isopropyl-4-methyl-1,2,3-thiadiazole-5-carboxamide	C14H17N3OS	ION=[M+H]+		6650.77	5742.67	6170.00	5211.67
2458	63.18	317.08455	18.915	Polyoxin C	C11H15N3O8	ION=[M+H]+		914.77	290.33	38.50	2913.67
2459	62.48	368.14348				ION=[M+H]+		1298.15	865.00	2934.17	2470.33
2460	61.56	324.1058	19.235	Furaltadone	C13H16N4O6	ION=[M+H]+	139-91-3	9252.77	5329.17	3316.67	9969.33
2461	61.57	111.04265				ION=[M+H]+		842.62	2099.17	664.50	1152.00
2462	62.3	230.09245	NA	Naproxen	C14H14O3	ION=[M+H]+	22204-53-1	3011.69	1158.00	3642.50	2177.67
2463	61.82	277.09054	NA	12-(aminomethyl)-4-methoxy-8-thia-11,14-diazatricyclo[7.5.0.0≤,?]tetradeca-1(9),2(7),3,5-tetraen-10-one	C13H15N3O2S	ION=[M+H]+		1716.15	1496.33	894.67	1247.67
2464	62.32	203.08208				ION=[M+H]+		8603.38	4305.00	10092.50	8501.33
2465	62.34	204.11079				ION=[M+H]+		4512.31	2236.50	2129.17	3302.00
2466	62.85	217.0964				ION=[M+H]+		220.00	4106.67	406.00	889.00
2467	62.94	274.11625	NA	Palythine-serine	C11H18N2O6	ION=[M+H]+		126.15	151.33	3794.17	147.00
2468	63.09	243.07582	NA	2-[2-(2h-1,2,3,4-tetrazol-5-yl)ethyl]-2,3-dihydro-1h-isoindole-1,3-dione	C11H9N5O2	ION=[M+H]+	412314-56-8	3390.31	2278.33	3928.33	4521.67

2469	63.12	246.12115	NA	1-methyl-2-[(2,4,6-trimethylphenyl)methylsulfanyl]imidazole	C14H18N2S	ION=[M+H]+		6689.23	1589.33	143.83	4891.00
2470	63.18	243.05223				ION=[M+H]+		0.00	7867.33	0.00	0.00
2471	64.22	306.14274	14.957	9a-Hydroxy-3,8a-dimethyl-5-methylene-2-oxo-2,4,4a,5,6,7,8,8a,9,9a-decahydronaphtho[2,3-b]furan-8-yl acetate	C17H22O5	ION=[M+K]+		63.08	214.50	9818.50	175.33
2472	64.12	294.08827				ION=[M+H]+		18457.08	13293.83	21786.00	14384.33
2473	63.26	586.2236				ION=[M+H]+		6119.23	4771.83	13654.50	12901.00
2474	64.05	185.94971				ION=[M+H]+		6788.00	6395.17	5826.33	2050.00
2475	65.15	218.12167	8.04	Meprobamate	C9H18N2O4	ION=[M+H]+	57-53-4	11970.15	0.00	0.00	0.00
2476	65.91	172.08407				ION=[M+H]+		470.92	643.00	1397.67	1800.67
2477	67.37	248.10189	6.108	Tetrahydrouridine	C9H16N2O6	ION=[M+H]+		3157.85	4113.83	4949.83	2822.00
2478	65.24	305.04101				ION=[M+H]+		445.38	6518.33	1235.50	2271.67
2479	66.4	312.0823				ION=[M+H]+		1397.85	650.83	65.67	204.33
2480	69.34	276.08771				ION=[M+H]+		1841.69	4643.33	3574.17	1484.00
2481	73.76	171.97023				ION=[M+H]+		13245.38	12124.33	12705.17	10976.67
2482	74.13	217.09597	NA	Pymetrozine	C10H11N5O	ION=[M+H]+	123312-89-0	343.23	6012.17	295.33	1602.67
2483	77.15	123.0313	10.575	Nicotinic acid	C6H5NO2	ION=[M+H]+		3718.31	1977.67	4985.17	9375.33
2484	74.19	216.07688	NA	2,6-diamino-3h,7h,8h-imidazo[4,5-g]quinazolin-8-one	C9H8N6O	ION=[M+H]+		3306.92	3175.00	3220.00	6579.00
2485	76.08	255.07546	12.878	N2-[3-(Trifluoromethyl)phenyl]-1,3,5-triazine-2,4-diamine	C10H8F3N5	ION=[M+H]+		11800.77	3586.67	14489.00	37632.67
2486	74.21	189.98158				ION=[M+H]+		6691.23	6099.33	5149.17	4569.67
2487	74.55	252.09847	NA	Guanitoxin	C7H17N4O4P	ION=[M+H]+		1078.31	2475.83	32.83	0.00
2488	77.05	291.09351	NA	Neu5ac2en	C11H17NO8	ION=[M+H]+		0.00	425.17	0.00	3238.00
2489	75.31	187.08623				ION=[M+H]+		641.08	2846.17	253.67	926.33
2490	76.57	444.81948				ION=[M+H]+		4680.46	4443.83	2473.83	7235.00
2491	76.57	234.08694	19.047	(2R)-5-Methoxy-2-methyl-2,3,8,9-tetrahydro-4H-furo[2,3-h]chromen-4-one	C13H14O4	ION=[M+H]+		5531.38	4125.33	3385.83	4604.67
2492	76.93	218.1185				ION=[M+H]+		2176.00	1811.83	645.17	1019.00
2493	78.1	246.06519	NA	Doxifluridine	C9H11FN2O5	ION=[M+H]+		1490.46	1244.83	1799.33	0.00
2494	78.98	293.06913				ION=[M+H]+		4582.31	1481.00	7183.50	3762.00
2495	80.74	157.95411				ION=[M+H]+		4948.62	2210.83	6036.00	1996.00
2496	79.59	253.94244				ION=[M+H]+		3678.15	2778.17	2153.50	162.67
2497	79.63	276.09456				ION=[M+H]+		2756.15	2291.83	4766.33	2097.33
2498	79.69	328.12502				ION=[M+H]+		15.85	0.00	3857.50	0.00
2499	81.07	552.15771				ION=[M+H]+		3622.46	1448.83	3287.00	8280.00
2500	82.03	305.24661				ION=[M+H]+		6.46	3314.00	7.50	11.00
2501	82.8	404.35005				ION=[M+H+H]2+		0.00	24001.17	0.00	0.00
2502	81.11	229.07105	4.818	N1-(2-Hydrazino-2-oxoethyl)-2,6-difluorobenzamide	C9H9F2N3O2	ION=[M+H]+		22607.08	0.00	260346.50	63824.33
2503	82.91	404.34526				ION=[M+H+H2]3+		0.00	2397.33	0.00	0.00
2504	82.64	235.93218				ION=[M+H]+		2815.23	2829.00	1787.50	56.33
2505	84.37	218.11996				ION=[M+H]+		360.00	434.00	380.00	572.67
2506	82.94	181.97836				ION=[M+H]+		3148.77	3089.50	3279.33	351.33
2507	85.98	255.9322				ION=[M-H2O+H]+		4223.54	3601.83	4180.00	2790.00
2508	85.93	323.05201				ION=[M+H]+		73.23	3263.67	678.17	177.00
2509	83.36	129.04212	1.751	Pyroglutamate	C5H7NO3	ION=[M+H]+		740.00	111.50	10254.17	2108.67
2510	83.81	183.06587	15.472	DL-4-Fluorophenylalanine	C9H10FNO2	ION=[M+H]+		326.62	6.67	4440.00	962.00
2511	83.42	185.07716				ION=[M+H]+		373.08	272.67	3356.83	1141.67
2512	85.04	232.1422	1.703	Mebutamate	C10H20N2O4	ION=[M+H]+		7021.54	2008.50	2160.00	514.00
2513	84.82	259.15341	14.66	(tert-butyloxycarbonyl)-alanyl-amino ethyl-formamide	C11H21N3O4	ION=[M+H]+		4642.31	1710.67	1579.33	870.33
2514	85.4	195.95477				ION=[M+H]+		123013.69	132710.17	88633.00	185475.33
2515	88.61	242.02047				ION=[M+H]+		4224.00	2561.50	8694.17	9440.33
2516	88.5	282.01183				ION=[M+H]+		2711.85	1958.83	4549.00	517.67
2517	88.14	246.12165				ION=[M+H]+		6042.77	702.17	1505.67	1586.00
2518	87.85	260.03013	3.443	D-Fructose 1-phosphate	C6H13O9P	ION=[M+H]+		6923.54	3810.83	14472.33	21256.33
2519	90.46	203.96071				ION=[M-H2O+H]+		6991.85	7342.67	5873.00	649.33
2520	91.77	291.08982				ION=[M+H]+		10047.69	4464.33	14452.33	8515.67
2521	91.92	157.954				ION=[M+H]+		5372.77	6250.83	3275.00	0.00
2522	91.75	274.11643	NA	Palythine-serine	C11H18N2O6	ION=[M+H]+		112.77	79.17	9861.67	857.33
2523	92.98	323.08555				ION=[M+H]+		370.92	5463.83	0.00	0.00
2524	93.08	444.81917				ION=[M+H]+		4802.31	3936.50	4259.00	7435.67
2525	93.66	340.0438				ION=[M+H]+		4122.15	1202.33	1982.50	0.00
2526	93.9	246.12084	18.063	Loxoprofen	C15H18O3	ION=[M+H]+		13647.85	1922.17	4416.67	7420.33
2527	94.19	246.05105	4.756	[(2,3,4,6-tetrahydroxyhexyl)oxy]phosphonic acid	C6H15O8P	ION=[M+H]+		26165.23	5671.33	28324.83	11515.33
2528	95.59	305.24562				ION=[M+H+H]2+		0.00	62434.83	0.00	0.00
2529	94.31	318.06231				ION=[M+H]+		7659.69	1656.83	3871.83	1719.33
2530	94.7	377.1455				ION=[M+H]+		1976.31	160.00	1215.67	33.33
2531	94.43	328.12541				ION=[M+H]+		0.00	42.17	3965.83	0.00
2532	94.25	214.01016				ION=[M+H]+		860.46	4418.17	6237.67	207.33
2533	94.32	217.09892	11.629	Pymetrozine	C10H11N5O	ION=[M+H]+	123312-89-0	153.38	9942.67	653.50	505.67
2534	95.49	643.36835				ION=[M+H+H2]3+		0.00	78.50	13.83	15171.33

2535	95.49	643.3682				ION=[M+H+H]2+		371.23	164.00	226.50	16296.67
2536	96.3	238.16761	9.21	1,5-Ditetrahydro-1H-pyrrol-1-ylpentane-1,5-dione	C13H22N2O2	ION=[M+H]+		0.00	0.00	0.00	5775.00
2537	94.58	306.14272	19.044	9a-Hydroxy-3,8a-dimethyl-5-methylene-2-oxo-2,4,4a,5,6,7,8,8a,9,9a-decahydronaphtho[2,3-b]furan-8-yl acetate	C17H22O5	ION=[M+H]+		96.46	2454.83	12966.17	23.00
2538	95.59	250.15059	19.209	Desmethylmianserin	C17H18N2	ION=[M+H]+		1604.77	2308.50	1480.17	948.33
2539	95.48	304.13806				ION=[M+H]+		625.69	98.33	5865.67	221.00
2540	96	244.1083	40.52	Palythine	C10H16N2O5	ION=[M+H]+		750.46	2586.17	179.67	49.33
2541	95.81	659.36266				ION=[M+H+H2]3+		0.00	0.00	0.00	107509.67
2542	95.93	306.15807				ION=[M+H]+		567.38	2976.17	0.00	190.33
2543	95.72	288.13219				ION=[M+H]+		0.00	3174.17	20804.00	0.00
2544	96.14	659.36277				ION=[M+H+H]2+		868.62	438.83	70.83	196869.67
2545	95.46	275.10784				ION=[M+H]+		0.00	67.83	1691.33	0.00
2546	94.81	215.08046	9.512	Kinetin	C10H9N5O	ION=[M+H]+		256.46	840.50	4055.33	0.00
2547	94.85	231.14777				ION=[M+H]+		1126.46	7175.00	24.67	0.00
2548	95.2	279.13293	14.261	Tert-butyl n-({2-amino-4-oxo-3h,4h,7h-pyrrolo[2,3-d]pyrimidin-5-yl}methyl)carbamate	C12H17N5O3	ION=[M+H]+		392.62	794.00	403.67	57956.33
2549	95.63	256.10511				ION=[M+H]+		184.15	587.50	3325.33	593.67
2550	95.42	252.10015	35.466	Guanitoxin	C7H17N4O4P	ION=[M+H]+		9657.69	14086.33	0.00	113.33
2551	95.55	131.09418	3.688	Leucine	C6H13NO2	ION=[M+H]+		5912.62	3450.83	4804.17	6401.00
2552	95.59	342.20975				ION=[M+H]+		884.62	43.33	0.00	0.00
2553	96.13	1276.57253				ION=[M+H+H]2+		1466.46	733.83	6760.00	580.33
2554	95.75	1276.56535				ION=[M+H+H2]3+		7724.62	6022.50	28340.50	9536.00
2555	96.29	234.111	6.848	Triethyleneglycol diacetate	C10H18O6	ION=[M+H]+		0.00	76773.83	71.00	0.00
2556	96.54	252.12205				ION=[M+H]+		0.00	6768.17	57.00	0.00
2557	96.66	202.08608	4.346	Metamitron	C10H10N4O	ION=[M-H2O+H]+	41394-05-2	10.46	25094.17	70.83	0.00
2558	97.18	350.09709				ION=[M+H]+		8.62	17113.17	14.17	67.00
2559	96.52	198.09099	12.094	Guaifenesin	C10H14O4	ION=[M+H]+		55.85	8992.00	9.50	0.00
2560	97.3	243.12532	NA	2-(4-Methoxybenzylidene)quinuclidin-3-one	C15H17NO2	ION=[M+H]+		108.00	1177.00	82.67	3827.00
2561	97.87	252.11153	10.125	N-cyclopropyl-4-{pyrazolo[1,5-b]pyridazin-3-yl}pyrimidin-2-amine	C13H12N6	ION=[M+H]+	551919-54-1	9930.31	11259.67	7011.17	7460.67
2562	97.64	347.06384	1.853	2'-Deoxyguanosine 5'-phosphate	C10H14N5O7P	ION=[M+H]+		11361.85	24766.33	69789.50	10427.00
2563	96.66	142.0628	7.308	Glycidyl methacrylate	C7H10O3	ION=[M+H]+	106-91-2	0.00	14211.83	38.83	11.00
2564	97.44	673.35781				ION=[M+H+H]2+		756.46	73.33	78.67	22202.33
2565	97.79	612.15418	2.505	Oxiglutatione	C20H32N6O12S2	ION=[M+H]+	27025-41-8	4770.00	2660.67	22760.50	8007.33
2566	97.57	514.23806				ION=[M+H]+		2493.69	62.33	76.50	61.33
2567	96.64	428.33756				ION=[M+H]+		2144.00	0.00	0.00	0.00
2568	97.33	358.18717				ION=[M+H]+		898.31	2248.83	461.67	6022.00
2569	97.68	293.11049				ION=[M+H]+		14269.23	6547.50	12109.83	16876.67
2570	97.11	673.35562				ION=[M+H+H2]3+		17.69	12.17	0.00	20816.00
2571	97.34	260.13798	11.316	Gaburedin B	C11H20N2O5	ION=[M+H]+		37591.38	10333.17	10321.50	6855.00
2572	97.13	276.09391				ION=[M+H]+		2025.38	1370.50	4051.17	0.00
2573	96.03	278.126	NA	Oxadixyl	C14H18N2O4	ION=[M+H]+	77732-09-3	3030.92	1083.00	497.33	2481.00
2574	96.07	192.02848	11.681	Citric acid	C6H8O7	ION=[M+H]+		1111.23	2134.33	3809.50	4662.67
2575	97.72	275.10003				ION=[M+H]+		23611.54	7200.33	23231.83	30748.67
2576	97.97	188.11724	15.836	Glycyl-L-leucine	C8H16N2O3	ION=[M+H]+		9421.69	2026.33	3526.67	5280.00
2577	98.23	115.06292	9.613	Proline	C5H9NO2	ION=[M+H]+		2485.38	347.00	852.33	1484.67
2578	98.87	198.17272	NA	Cycluron	C11H22N2O	ION=[M+H]+		2730.77	11.00	246.17	0.00
2579	96.43	344.1525	NA	N7-butyl-n2-(5-chloro-2-methylphenyl)-5-methyl[1	C17H21ClN6	ION=[M+H]+		16.46	27.67	2231.17	0.00
2580	98.14	497.46213				ION=[M+H+H2]3+		59624.92	0.00	9495.67	0.00
2581	97.93	214.13347	11.104	Desthiobiotin	C10H18N2O3	ION=[M+H]+		16975.38	3014.83	5303.50	12794.00
2582	98.98	1251.52146				ION=[M+H+H]2+		176.46	272.33	1668.17	845.33
2583	97.64	696.21921				ION=[M+H]+		3045.23	30.00	8361.33	2578.00
2584	96.92	321.1803				ION=[M+H]+		2447.23	645.17	1219.83	1862.00
2585	98.07	216.14912	22.175	Anaephene C	C15H20O	ION=[M+H]+		31349.38	5753.83	8286.50	18174.00
2586	96.92	282.12183	NA	2-(heptan-2-yl)-4,6-dinitrophenol	C13H18N2O5	ION=[M+H]+		2816.77	206.17	601.17	258.67
2587	97.79	232.10725	19.361	N~2~-(3-Carboxy-1-hydroxypropylidene)ornithine	C9H16N2O5	ION=[M+H]+	80102-04-1	9170.31	42129.67	11016.50	4359.00
2588	98.96	1712.74932				ION=[M+H+H]2+		874.31	804.17	4076.67	212.67
2589	96.25	171.64143				ION=[M+H]+		816.00	0.00	0.00	0.00
2590	97.34	970.30716				ION=[M+H+H]2+		3776.62	3812.50	9604.00	3136.67
2591	98.41	1269.53261				ION=[M+H+H2]3+		20113.54	8546.00	37079.00	20937.67
2592	96.73	1262.50858				ION=[M+H+H]2+		2683.38	2187.67	2885.00	2073.33
2593	98.26	1269.542				ION=[M+H+H]2+		11526.62	3917.33	20190.17	16299.67
2594	96.47	1262.50058				ION=[M+H+H2]3+		5951.54	4574.50	5069.83	2491.67
2595	98.8	555.13204				ION=[M+H]+		3832.00	2938.33	7395.50	5963.67
2596	97.59	273.16722				ION=[M+H]+		942.00	0.00	90.17	0.00
2597	98.24	497.46376				ION=[M+H+H]2+		29523.38	0.00	5733.67	0.00
2598	96.45	262.11621	12.21	1-(5,8-Dimethoxy-2,2-dimethyl-2H-1-benzopyran-6-yl)ethan-1-one	C15H18O4	ION=[M+H]+	62458-48-4	146.15	2266.67	50.17	113.00

2599	97.52	428.33331	17.038	Nandrolone decanoate	C28H44O3	ION=[M+H+H]2+		139249.85	0.00	0.00	0.00
2600	96.77	554.23381	2.497	Anabaenolysin var 5	C28H34N4O8	ION=[M+H]+		10695.38	976.67	14317.67	5194.00
2601	99.06	193.62823				ION=[M+H]+		159.54	66.33	96.50	9186.67
2602	99.21	318.1617				ION=[M+H]+		767.69	48.33	465.33	8099.00
2603	98.65	202.13282	11.663	Glutamine T-Butyl Ester	C9H18N2O3	ION=[M+H]+	39741-62-3	26205.38	6962.17	9546.00	16969.00
2604	96.66	166.06391	16.702	(+-)-atrolactic acid	C9H10O3	ION=[M+H]+		0.00	3563.50	0.00	0.00
2605	99.09	432.29539				ION=[M+H+H]2+		0.00	0.00	42.50	4255.00
2606	97.16	274.10426				ION=[M+H]+		0.00	3477.67	0.00	0.00
2607	96.71	302.18322	4.386	Tandamine	C18H26N2S	ION=[M+H]+	58167-78-5	6406.46	0.00	0.00	0.00
2608	97.03	215.66494				ION=[M+H]+		190.92	0.00	3981.83	17.33
2609	97.13	264.1428				ION=[M+H]+		0.00	0.00	566.83	0.00
2610	97.25	515.41886				ION=[M+H+H]2+		2006.62	0.00	145.17	0.00
2611	97.3	229.11601				ION=[M+H]+		251.08	0.00	0.00	2152.67
2612	97.57	287.15078				ION=[M+H]+		1509.54	234.17	412.00	186.33
2613	97.58	515.41891				ION=[M+H+H2]3+		2949.23	0.00	892.83	0.00
2614	97.72	519.43145				ION=[M+H]+		636.62	1741.17	2188.83	920.67
2615	97.57	253.62537				ION=[M+H]+		858.00	137.33	15.17	105.00
2616	98.1	296.10066	16.146	Pukeleimide D	C13H16N2O6	ION=[M+H]+		4941.08	219.50	490.33	2778.00
2617	98.5	129.04195	12.933	Pyroglutamate	C5H7NO3	ION=[M+H]+		1500.62	1444.50	4428.17	2566.33
2618	99.09	288.15118				ION=[M+H]+		429.08	643.17	75.83	8794.00
2619	99.1	237.15027	11.517	4-Fluoro IPV	C14H20FNO	ION=[M+H]+		0.00	0.00	518.50	0.00
2620	99.19	474.30076				ION=[M+H+H]2+		221.69	20.33	3980.83	0.00
2621	99.23	310.11763	NA	6-[2-(4-hydroxy-3-nitrophenyl)acetamido]hexanoic acid	C14H18N2O6	ION=[M+H]+	10463-23-7	3670.92	219.17	377.33	325.33
2622	99.26	249.08479	16.638	Daniquidone	C15H11N3O	ION=[M+H]+	67199-66-0	1202.31	2449.17	320.83	0.00
2623	100.95	496.1912				ION=[M+H]+		11919.54	6694.50	14687.50	16529.67
2624	99.89	471.15944				ION=[M-H2O+H]+		7042.77	3358.67	15117.67	8814.00
2625	100.67	184.12197				ION=[M+H]+		3082.77	815.00	1254.67	2723.00
2626	99.75	498.10686				ION=[M+H+H]2+		13583.23	15504.50	12555.50	19408.33
2627	99.33	347.17049				ION=[M+H]+		3757.23	1127.00	1024.17	3527.33
2628	100.29	1340.5706				ION=[M+H+H2]3+		3173.69	1731.67	5322.50	3782.67
2629	99.59	1340.57661				ION=[M+H+H]2+		1448.62	690.67	2377.00	1995.00
2630	101.11	127.09936				ION=[M+H]+		373.54	0.00	4895.50	17.00
2631	100.22	309.13572	14.253	Fluoxetine	C17H18F3NO	ION=[M+H]+		2145.08	101.17	350.50	550.67
2632	101.17	238.09659	15.984	Felbamate	C11H14N2O4	ION=[M+H]+		5070.31	253.17	1278.33	2059.33
2633	99.52	203.08128	15.923	3-amino-4,5,6-trihydroxy-2-methoxy-5-methyl-2-cyclohexen-1-one	C8H13NO5	ION=[M+H]+		2111.08	3658.83	4921.17	2890.67
2634	99.54	267.0968	3.398	Vidarabine | Adenosin	C10H13N5O4	ION=[M+H]+		107848.31	225053.17	191551.17	261373.67
2635	99.42	164.04841	17.301	2-Hydroxycinnamic acid	C9H8O3	ION=[M+H]+		9390.00	1185.17	4531.17	6890.67
2636	101.08	187.20623				ION=[M+H]+		811.38	10.67	16248.33	274.67
2637	99.74	181.07517	12.679	Tyrosin	C9H11NO3	ION=[M+H]+		44555.85	4613.17	21114.67	32268.00
2638	100.76	532.25256				ION=[M+H+H]2+		838.15	0.00	0.00	0.00
2639	99.54	176.15995				ION=[M+H]+		2636.46	0.00	19.83	0.00
2640	99.62	332.07821	13.013	2-{4-[(4-hydroxyphenyl)methylidene]-2-[3-(methylsulfanyl)propanoyl]-5-oxo-4,5-dihydro-1h-imidazol-1-yl}acetaldehyde	C16H16N2O4S	ION=[M+H]+		105.38	116.17	2147.00	674.67
2641	99.74	136.03905				ION=[M+H]+		4203.23	1602.50	0.00	1925.67
2642	99.88	387.14784	NA	4-{[5-(cyclohexylamino)-[1,2,4]triazolo[1,5-a]pyrimidin-7-yl]amino}benzene-1-sulfonamide	C17H21N7O2S	ION=[M+H]+		1206.62	2006.50	2334.83	490.67
2643	100.08	498.10983				ION=[M+H]+		2532.46	2602.33	2059.50	3593.00
2644	100.24	403.13599				ION=[M+H]+		1990.00	4613.33	1146.00	0.00
2645	100.62	273.16842	18.382	Tilidine	C17H23NO2	ION=[M+H]+		2848.31	1494.00	623.33	952.33
2646	100.3	203.13406	NA	Aphanorphine	C13H17NO	ION=[M+H]+		2906.62	1721.17	1217.17	2313.67
2647	100.3	295.10272	16.229	7-(hydroxymethyl)-3-phenyl-1,6-dioxa-2-azaspiro[4.5]dec-2-ene-8,9,10-triol	C14H17NO6	ION=[M+H]+		2939.38	4853.17	139.33	0.00
2648	100.41	371.31406				ION=[M+H+H]2+		60.92	156984.33	35.83	0.00
2649	102.01	228.11218	9.863	Bisphenol A	C15H16O2	ION=[M+H]+		2814.15	853.83	673.33	1731.33
2650	100.58	442.35157				ION=[M+H]+		7546.00	0.00	0.00	0.00
2651	103.32	179.04549	12.353	Isoxanthopterin	C6H5N5O2	ION=[M+H]+		1217.23	1573.83	7416.67	2841.00
2652	101.75	257.24664				ION=[M+H]+		4194.00	178.83	463.67	21.67
2653	102.01	507.20697				ION=[M+H]+		1610.62	1416.17	7191.00	5724.33
2654	101.4	512.26178				ION=[M+H]+		159.08	539.83	82.67	14477.33
2655	101.21	424.34142				ION=[M+H+H]2+		9909.38	0.00	0.00	0.00
2656	101.54	490.27674				ION=[M+H+H]2+		3664.92	0.00	0.00	0.00
2657	102.3	331.06824	16.21	2'-Deoxyadenosine 5'-monophosphate (dAMP)	C10H14N5O6P	ION=[M+H]+		1292.46	5876.83	7912.33	1062.67
2658	102.27	696.21867				ION=[M+H]+		1698.00	0.00	5056.00	1607.67
2659	101.24	342.33296				ION=[M+H+H]2+		8400.92	0.00	0.00	0.00
2660	101.18	266.12702	NA	2-{2-Oxo-2-[4-(1H-pyrrol-1-yl)piperidino]ethoxy}acetic acid	C13H18N2O4	ION=[M+H]+		10884.15	2465.33	5073.17	10770.00
2661	101.89	245.13763	18.095	Tasimelteon	C15H19NO2	ION=[M+H]+	609799-22-6	15275.38	3430.83	3494.17	10269.67
2662	101.4	414.28164	13.15	Desoxycortone	C26H38O4	ION=[M+H+H]2+	808-48-0	0.00	258.50	0.00	632498.00
2663	100.45	331.177	NA	Tetramethrin	C19H25NO4	ION=[M+H]+		1258.77	0.00	501.83	0.00

2664	100.71	244.07078				ION=[M+H]+		16156.92	7757.83	9382.83	17862.33
2665	100.93	272.0787				ION=[M+H+H]2+		2010.00	991.83	1068.33	2953.33
2666	101.05	251.10265	17.667	2-(2-Hydroxy-Phenyl)-1h-Indole-5-Carboxamidine	C15H13N3O	ION=[M+H]+	179748-10-8	25469.23	37094.67	40454.67	35238.67
2667	101.97	232.14289	10.811	Mebutamate	C10H20N2O4	ION=[M+H]+		11924.92	1920.00	2283.00	3488.33
2668	102.05	530.27105	8.997	(5xi,6alpha,7alpha,9xi,16xi)-16-(beta-D-Glucopyranosyloxy)-6,7,17-trihydroxykauran-19-oic acid	C26H42O11	ION=[M+H]+		541.38	246.17	1135.83	17180.67
2669	104.21	342.33528				ION=[M+H+H]2+		27858.00	24.67	132.17	99.67
2670	105.34	155.64742				ION=[M+H]+		9255.54	0.00	508.17	76.33
2671	103.22	442.34923				ION=[M+H+H]2+		1071393.38	0.00	0.00	10924.33
2672	102.57	414.284				ION=[M+H]+		0.00	0.00	0.00	14888.67
2673	103.3	634.21004				ION=[M+H]+		1111.85	264.67	9693.83	1441.33
2674	104.47	259.15319	10.279	(tert-butyloxycarbonyl)-alanyl-amino ethyl-formamide	C11H21N3O4	ION=[M+H]+		15574.31	4887.50	5217.50	7674.67
2675	102.83	124.05186				ION=[M+H]+		6.46	2964.83	17.33	19.00
2676	104.09	479.20013				ION=[M+H]+		51.08	4574.83	139.83	154.00
2677	103.9	319.12844				ION=[M+H]+		0.00	2526.50	0.00	0.00
2678	105.07	195.95529				ION=[M+H]+		5759.54	12750.83	1734.33	8175.33
2679	104.46	442.35199				ION=[M+H]+		20506.62	0.00	0.00	91.67
2680	103.97	241.14317				ION=[M+H]+		2282.31	736.50	840.33	1264.33
2681	104.19	282.12179	11.538	Erteberel	C18H18O3	ION=[M+H]+	533884-09-2	5532.00	199.67	1072.33	3183.00
2682	104.29	497.21	5.97	Validamycin A	C20H35NO13	ION=[M+H]+		72.62	28954.67	236.83	135.00
2683	106.52	181.06073				ION=[M-H2O+H]+		7985.85	4931.67	9215.83	2230.00
2684	106.33	172.07174				ION=[M+H]+		1716.92	2392.17	2191.67	1557.67
2685	106.55	345.19227	NA	Bevantolol	C20H27NO4	ION=[M+H]+		1830.92	253.17	342.00	1232.33
2686	106.28	278.12982	12.824	1-phenyl-6-phenylimino-1,3,5-triazine-2,4-diamine	C15H14N6	ION=[M+H]+		4020.15	1105.67	918.50	2409.67
2687	106.55	260.13827				ION=[M+H]+		3900.77	1160.50	1281.67	1924.33
2688	104.37	656.23227				ION=[M+H]+		948.46	1925.67	9058.00	1376.00
2689	105.08	424.33985				ION=[M+H+H]2+		18876.15	0.00	0.00	132.00
2690	107.06	280.10606				ION=[M+H]+		14180.15	2960.67	4824.50	6258.33
2691	106.65	232.14288	8.855	Mebutamate	C10H20N2O4	ION=[M+H]+		28555.54	9629.83	8098.17	15466.00
2692	106.25	308.95498				ION=[M+H]+		1701.69	2076.83	1992.00	270.67
2693	104.95	131.09425	12.919	Leucine	C6H13NO2	ION=[M+H]+		2416.46	717.67	1229.67	1877.67
2694	104.57	442.34736				ION=[M+H+H]2+		550852.31	0.00	0.00	0.00
2695	104.67	202.13299	6.985	Glutamine T-Butyl Ester	C9H18N2O3	ION=[M+H]+	39741-62-3	17763.08	4905.33	5326.50	15059.67
2696	104.77	152.03337	8.065	Xanthine	C5H4N4O2	ION=[M+H]+		2392.15	11903.17	1800.17	3225.67
2697	105.22	188.1174	10.71	Glycyl-L-leucine	C8H16N2O3	ION=[M+H]+		9610.62	2123.50	3265.33	4299.67
2698	106.58	257.24641				ION=[M+H]+		1685.08	0.00	41.83	0.00
2699	106.6	214.0101				ION=[M+H]+		6270.77	9391.67	5693.83	301.67
2700	106.82	715.37576				ION=[M+H+H]2+		312.92	119.00	29.83	6771.33
2701	106.01	150.08925	5.885	Triethylene glycol	C6H14O4	ION=[M+H]+		2028.15	2428.67	1851.67	5264.67
2702	108.36	302.18449	7.484	Formestane	C19H26O3	ION=[M+H]+	566-48-3	5785.54	0.00	0.00	0.00
2703	107.09	1009.4376				ION=[M+H+H]2+		616.15	50.67	2683.33	52.33
2704	108.46	538.34038				ION=[M+H]+		7494.62	0.00	0.00	0.00
2705	108.1	715.37332				ION=[M+H+H2]3+		0.00	0.00	0.00	24543.33
2706	107.68	281.07719	NA	Nitrazepam	C15H11N3O3	ION=[M+H]+		0.00	1953.00	430.67	4411.33
2707	107.13	428.33343				ION=[M+H+H]2+		1826.92	0.00	0.00	0.00
2708	107.4	247.12453				ION=[M+H]+		0.00	0.00	0.00	1010.67
2709	106.77	290.94402				ION=[M+H]+		3460.00	4317.33	4417.33	587.00
2710	107.39	197.05606	12.176	Aplodan	C4H12N3O4P	ION=[M+H]+		0.00	0.00	2322.00	92.00
2711	107.44	292.09097	NA	Ethylenediaminetetraacetic acid	C10H16N2O8	ION=[M+H]+		5448.46	7340.83	3333.83	7860.00
2712	110.9	496.19056				ION=[M+H]+		87743.69	61235.00	98724.33	130000.00
2713	109.18	293.11085				ION=[M+H]+		62754.31	18684.33	57023.50	83882.00
2714	109.01	586.22357				ION=[M+Na]+		8194.62	2389.17	10197.17	78.33
2715	109.81	232.10666	12.356	N~2~-(3-Carboxy-1-hydroxypropylidene)ornithine	C9H16N2O5	ION=[M+Na]+	80102-04-1	1039.38	551.00	6138.50	75.33
2716	110.74	203.0808				ION=[M+H]+		5208.77	4578.67	6817.33	5945.33
2717	110.49	248.09393	NA	Sudan I	C16H12N2O	ION=[M+H]+		0.00	809.17	0.00	0.00
2718	109.03	236.11704	14.557	Carbetamide	C12H16N2O3	ION=[M+H]+	16118-49-3	11163.85	3619.50	5456.50	4370.33
2719	110.2	185.06988				ION=[M+H]+		6645.69	3344.83	6945.67	9077.00
2720	108.72	248.11906				ION=[M+H]+		2024.77	1029.83	2646.00	2220.00
2721	109.26	547.19996				ION=[M+H]+		720.31	267.17	3399.17	0.00
2722	110.88	518.17285				ION=[M+H]+		12586.62	17427.50	22499.17	1861.00
2723	112.11	294.11885	10.997	Aspartame	C14H18N2O5	ION=[M+H]+		10908.77	2902.67	5824.17	11058.33
2724	111.73	268.08117	6.895	2-{7-hydroxy-1h-pyrazolo[4,3-d]pyrimidin-3-yl}-5-(hydroxymethyl)oxolane-3,4-diol	C10H12N4O5	ION=[M+H]+		28272.92	3017.00	22268.50	13935.00
2725	111.4	656.23119				ION=[M+H]+		2751.69	4046.50	13246.17	7834.33
2726	112.42	222.10161	NA	Buthionine Sulfoxime	C8H18N2O3S	ION=[M+H]+		8701.38	2426.83	3909.50	2382.33
2727	111.42	347.06397	17.676	2'-Deoxyguanosine 5'-phosphate	C10H14N5O7P	ION=[M+H]+		1875.08	29611.33	17726.33	4153.67
2728	111.38	137.04687	12.476	Sunbrella	C7H7NO2	ION=[M+H]+		4319.69	1703.50	3779.00	4548.67
2729	109.51	285.07693				ION=[M+H]+		1004.92	0.00	0.00	0.00
2730	111.77	136.03818				ION=[M+H]+		38049.69	3276.67	31329.67	6805.33
2731	110.96	444.04284				ION=[M+H+H]2+		0.00	1328.50	589.00	0.00

2732	111.38	363.07981				ION=[M+H]+		0.00	1190.83	0.00	0.00
2733	110.88	496.18936				ION=[M+H+H]2+		4113.08	2315.33	3736.17	5074.00
2734	111.93	272.07635				ION=[M+H+H]2+		656.46	718.83	0.00	14270.00
2735	112.32	243.12316				ION=[M+H]+		247.85	813.50	479.17	7780.33
2736	115.03	283.09166	11.679	Guanosine	C10H13N5O5	ION=[M+H]+		56001.38	51726.33	46965.17	18945.00
2737	114.13	230.16383	8.21	Tert-butyl N-(1-amino-4-methyl-1-oxopentan-2-yl)carbamate	C11H22N2O3	ION=[M+H]+	70533-96-9	22004.62	6252.33	5157.17	4222.67
2738	114.8	151.04948	2.6	Guanine	C5H5N5O	ION=[M+H]+		31933.38	30023.83	23667.50	10833.33
2739	112.38	242.12749	7.047	2,4-Diamino-6-Phenyl-5,6,7,8,-Tetrahydropteridine	C12H14N6	ION=[M+H]+		4057.08	1868.33	1789.00	1487.33
2740	116.33	157.95359				ION=[M+H]+		4956.77	5433.33	5451.00	1157.00
2741	116.33	185.95006				ION=[M+H]+		6584.77	5471.50	5466.00	1651.33
2742	114.88	228.14781	8.357	4-{[(cyclohexylamino)carbonyl]amino}butanoic acid	C11H20N2O3	ION=[M+H]+	511552-46-8	9856.31	1481.83	14854.67	1767.00
2743	113.5	262.08042	1.776	8,9,10-trihydroxy-7-(hydroxymethyl)-3-methyl-6-oxa-1,3-diazaspiro[4.5]decane-2,4-dione	C9H14N2O7	ION=[M+H]+		13887.69	25709.83	21904.17	31320.33
2744	116.34	265.08191	8.36	4-(Trifluoromethyl)-5,6-dihydrobenzo[H]quinazolin-2-amine	C13H10F3N3	ION=[M+H]+		0.00	1977.50	319.67	907.00
2745	115.01	260.13783	4.877	4-[5-(hydroxymethyl)-8-methyl-3-oxabicyclo[3.3.1]non-7-en-2-yl]phenol	C16H20O3	ION=[M+H]+		7235.08	2666.67	2377.17	1514.67
2746	117.66	345.1898				ION=[M+H]+		1575.54	209.67	355.83	488.67
2747	120.64	442.35078				ION=[M+H+H]2+		90174.77	112.17	0.00	481.67
2748	119.51	405.29849				ION=[M+H+H]2+		29.85	43892.33	0.00	0.00
2749	117.95	195.64159				ION=[M+H]+		8.15	1615.83	0.00	0.00
2750	120.53	159.08994	6.708	N-Acetylvaline	C7H13NO3	ION=[M+H]+		0.00	8.33	8.83	10466.33
2751	119.17	174.01718	NA	trans-Aconitic acid	C6H6O6	ION=[M+Na]+		1764.31	1842.33	2434.17	0.00
2752	121.21	324.03605	12.134	Uridine monophosphate	C9H13N2O9P	ION=[M+H]+		773.85	2524.67	4021.83	1786.33
2753	119.58	271.00022				ION=[M+H]+		1827.23	1967.50	2772.17	0.00
2754	121.66	616.19969	11.442	Polyoxin A	C23H32N6O14	ION=[M+H]+		44.46	4143.33	293.67	65.67
2755	120.58	246.12169				ION=[M+H]+		99307.38	159127.67	262684.83	55978.67
2756	121.69	222.02269				ION=[M+H]+		6.77	1311.00	689.67	0.00
2757	121.83	444.04437				ION=[M+H+H]2+		0.00	1425.17	0.00	0.00
2758	121.06	329.05273	3.87	Adenosine 3'5'-cyclic monophosphate	C10H12N5O6P	ION=[M+H]+		345.69	26659.33	5948.00	1574.33
2759	123.69	715.37541				ION=[M+H+H]2+		34.92	49.17	43.00	4347.67
2760	124.3	136.03837	3.453	Hypoxanthine	C5H4N4O	ION=[M+H]+		5034.46	747.67	3671.17	4121.00
2761	127.15	347.06301	9.354	2'-Deoxyguanosine 5'-phosphate	C10H14N5O7P	ION=[M+H]+		719.08	2880.83	5167.33	7544.67
2762	127.06	151.04949	3.962	Guanine	C5H5N5O	ION=[M+H]+		8431.38	4030.67	6748.50	8639.00
2763	127.27	267.09708	13.043	Vidarabine | Adenosin	C10H13N5O4	ION=[M+H]+		4916.77	2365.83	3720.67	4650.67
2764	128.91	474.30249				ION=[M+H+H]2+		373.38	21.33	8720.17	0.00
2765	127.86	187.20626				ION=[M+H]+		197.85	25.33	3651.17	29.00
2766	129.2	230.1637	2.617	Tert-butyl N-(1-amino-4-methyl-1-oxopentan-2-yl)carbamate	C11H22N2O3	ION=[M+H]+	70533-96-9	22546.62	3576.67	4119.67	13922.00
2767	131.51	252.1115	2.594	Alanyltyrosine	C12H16N2O4	ION=[M+H]+		4177.69	738.33	1436.17	2925.00
2768	131.73	232.0708	10.576	Kifunensine	C8H12N2O6	ION=[M+H]+	109944-15-2	130.46	31.67	2442.50	784.33
2769	132.83	248.09037				ION=[M+H]+		0.00	2697.67	0.00	0.00
2770	133.48	791.31041				ION=[M+H]+		0.00	0.00	0.00	13208.33
2771	133.43	244.179				ION=[M+H]+		93.23	3464.33	0.00	0.00
2772	134.81	1009.43772				ION=[M+H+H]2+		3165.08	343.67	10174.83	333.67
2773	137.07	345.00237				ION=[M+H]+		6172.62	9752.17	4606.00	17204.67
2774	138.58	222.10151	NA	Buthionine Sulfoxime	C8H18N2O3S	ION=[M+H]+		3106.00	532.83	894.67	1736.67
2775	138.78	228.14838	8.105	4-{[(cyclohexylamino)carbonyl]amino}butanoic acid	C11H20N2O3	ION=[M+H]+	511552-46-8	6000.00	408.83	2133.00	5279.67
2776	139.41	292.09079	1.585	Ethylenediaminetetraacetic acid	C10H16N2O8	ION=[M+H]+		7188.92	21591.33	4050.50	25689.67
2777	140.53	194.11601	12.238	N'',N'''-Di-(1E,2E)-2-buten-1-ylidenecarbonohydrazide	C9H14N4O	ION=[M+H]+		7487.23	4762.00	3705.67	12684.67
2778	141.9	206.04427	7.663	1-hydroxy-1-methylpropane-1,2,3-tricarboxylic acid	C7H10O7	ION=[M+H]+	71183-66-9	3820.31	1585.50	6214.00	4631.00
2779	140.51	234.08623				ION=[M+H]+		5078.15	10138.83	5102.50	12124.33
2780	141.85	244.1788				ION=[M+H]+		5203.69	1373.17	0.00	0.00
2781	142.21	297.08331				ION=[M+H]+		2959.85	692.50	1873.67	1136.00
2782	145.24	275.10064	14.133	5'-deoxytoyocamycin	C12H13N5O3	ION=[M+H]+		30672.31	4778.17	12367.00	24280.67
2783	147.25	324.03593	3.839	Uridine monophosphate	C9H13N2O9P	ION=[M-H2O+H]+		96.92	3983.33	1129.00	1451.33
2784	147.68	616.1992	9.321	Polyoxin A	C23H32N6O14	ION=[M+H]+		10609.08	11.67	26729.17	9306.67
2785	148.83	228.14867				ION=[M+H]+		6101.08	622.00	2013.17	4634.33
2786	149.88	230.16369				ION=[M+H]+		18658.15	4141.67	15851.83	12326.33
2787	150.89	345.00239				ION=[M+H]+		5588.92	5981.83	3936.33	3631.67
2788	151.37	292.09078	3.738	Ethylenediaminetetraacetic acid	C10H16N2O8	ION=[M+H]+		8387.54	6599.67	5338.33	13125.67
2789	154.61	216.09951	16.943	4-Oxosebacic Acid	C10H16O5	ION=[M+H]+	114212-45-2	340.31	3274.50	220.17	152.67
2790	155.12	234.08617				ION=[M+H]+		10104.15	11774.50	8346.67	21640.00
2791	158.98	214.00983				ION=[M+H]+		704.00	1764.67	3169.00	346.33
2792	159.71	1705.701				ION=[M+H+H2]3+		1565.38	776.33	1555.00	1973.33
2793	160.1	208.09577	6.16	2-butyl-1h,4h,5h,6h,7h-imidazo[4,5-d]pyridazine-4,7-dione	C9H12N4O2	ION=[M+H]+	140199-54-8	2457.23	969.00	454.17	3953.33

2794	161.23	244.17883				ION=[M+H]+		34904.00	67.50	6753.17	0.00
2795	161.31	174.01826	3.928	trans-Aconitic acid	C6H6O6	ION=[M+Na]+		140.31	19258.67	43337.33	31.33
2796	161.62	403.98887				ION=[M+H+H]2+		98.31	2687.67	4078.00	60.33
2797	161.7	230.1639	8.409	Tert-butyl N-(1-amino-4-methyl-1-oxopentan-2-yl)carbamate	C11H22N2O3	ION=[M+H]+	70533-96-9	657.54	68.50	35761.67	1318.67
2798	161.33	559.99724				ION=[M+H]+		0.00	5452.50	13207.17	0.00
2799	161.33	268.96198				ION=[M+H]+		0.00	2480.17	3195.83	0.00
2800	160.76	271.0002				ION=[M+H]+		215.23	2427.50	2960.83	0.00
2801	161.65	401.95203				ION=[M+H]+		21.54	2994.00	5718.33	0.00
2802	161.31	421.99988				ION=[M+H+H]2+		12.00	2178.50	3866.00	0.00
2803	161.16	385.97854				ION=[M+H]+		25.38	1729.17	2457.50	55.00
2804	163.8	286.97259				ION=[M+H]+		214.62	2110.00	2703.50	0.00
2805	165.87	599.24655	10.998	2-(carboxymethoxy)-5-{2-[2-(3-carboxypropanamido)-3-phenylpropanamido]-2-(pentylcarbamoyl)ethyl}benzoic acid	C30H37N3O10	ION=[M+H]+		38.31	9677.17	0.00	0.00
2806	166.24	258.19434	5.918	Elaiomycin	C13H26N2O3	ION=[M+H]+		90832.00	65745.17	32.50	503.00
2807	166.29	432.34324				ION=[M+H+H]2+		0.00	1939.00	18.50	0.00
2808	168.56	271.00031				ION=[M+H]+		55.54	1955.67	1970.17	97.00
2809	171.16	1705.7017				ION=[M+H+H2]3+		1028.62	134.33	1232.67	2334.00
2810	177.99	258.19422	3.147	Elaiomycin	C13H26N2O3	ION=[M+H]+		62645.08	664.17	1077.00	375.33
2811	181.82	185.94993				ION=[M+H]+		5906.31	7597.00	3558.00	3240.00
2812	183.87	432.34593				ION=[M+H+H]2+		7.54	1589.00	0.00	0.00
2813	186.1	260.08388	13.697	Diethyl 4-methoxyphenyl phosphate	C11H17O5P	ION=[M+H]+		0.00	3018.33	0.00	0.00
2814	190.27	432.34566				ION=[M+H+H]2+		0.00	2037.83	0.00	0.00
2815	193.15	294.15869	0.424	Cyclocarbamide B	C15H22N2O4	ION=[M+H]+		3900.15	52.33	704.67	494.67
2816	198.99	345.04774	5.699	Cyclic guanosine monophosphate	C10H12N5O7P	ION=[M+H]+		385.69	15806.33	4184.33	0.00
2817	204.26	599.24091	5.003	Methyl 4-{[4-(dimethylamino)-5-hydroxy-6-methyloxan-2-yl]oxy}-2-ethyl-2,5,12-trihydroxy-7-methoxy-6,11-dioxo-1,2,3,4,6,11-hexahydrotetracene-1-carboxylate	C31H37NO11	ION=[M+H+H]2+		50.77	8467.17	4.33	0.00
2818	204.76	185.94971				ION=[M+H]+		6164.92	4896.67	3614.67	3265.67
2819	207.24	240.99207				ION=[M+H]+		53.85	44.00	1520.50	222.00
2820	207.66	363.0583	5.621	5'-Guanylic acid	C10H14N5O8P	ION=[M+H]+		49.23	1114.83	2113.00	172.33
2821	210.02	599.24033	9.05	Methyl 4-{[4-(dimethylamino)-5-hydroxy-6-methyloxan-2-yl]oxy}-2-ethyl-2,5,12-trihydroxy-7-methoxy-6,11-dioxo-1,2,3,4,6,11-hexahydrotetracene-1-carboxylate	C31H37NO11	ION=[M+H+H]2+		98.77	35530.50	0.00	19.00
2822	209.16	242.07301	1.989	N-(4-sulfamoylphenethyl)acetamide	C10H14N2O3S	ION=[M+H]+	41472-49-5	313.85	25827.00	0.00	155.00
2823	209.84	599.24617	3.424	2-(carboxymethoxy)-5-{2-[2-(3-carboxypropanamido)-3-phenylpropanamido]-2-(pentylcarbamoyl)ethyl}benzoic acid	C30H37N3O10	ION=[M+H]+		35.54	13409.00	18.33	9.67
2824	211.19	260.13712	18.287	4-[5-(hydroxymethyl)-8-methyl-3-oxabicyclo[3.3.1]non-7-en-2-yl]phenol	C16H20O3	ION=[M+H]+		1796.00	189.00	20859.33	1958.33
2825	211.39	345.04501	18.137	Cyclic guanosine monophosphate	C10H12N5O7P	ION=[M+H]+		0.00	2835.50	48.17	1163.00
2826	212.91	131.09423	4.283	Leucine	C6H13NO2	ION=[M+H]+		1295.54	1950.00	3247.17	269.33
2827	215.95	187.06452	1.454	Indole-3-acrylic acid	C11H9NO2	ION=[M+H]+		6156.31	2222.50	4180.00	1280.00
2828	218.28	204.09113				ION=[M+H]+		10325.23	3175.50	6745.33	3561.67
2829	219.32	294.15794	7.551	Cyclocarbamide B	C15H22N2O4	ION=[M+H]+		3690.31	258.00	939.33	618.67
2830	219.48	260.13704	6.304	Gaburedin B	C11H20N2O5	ION=[M+H]+		74735.54	99390.33	136467.67	74694.33
2831	219.8	240.99181				ION=[M+H]+		77.23	35.33	1688.67	216.67
2832	220.1	185.94967				ION=[M+H]+		7677.38	7934.17	7979.33	3827.00
2833	225.5	294.15823	2.876	Cyclocarbamide B	C15H22N2O4	ION=[M+H]+		3814.31	209.50	415.50	2880.33
2834	227.91	204.09115	NA	Ethotoin	C11H12N2O2	ION=[M+H]+	86-35-1	1297.69	895.83	1108.67	1900.00
2835	235.43	242.07288	7.854	N-(4-sulfamoylphenethyl)acetamide	C10H14N2O3S	ION=[M+H]+	41472-49-5	159.23	9905.00	0.00	99.67
2836	237.48	240.99219				ION=[M+H]+		163.38	74.00	2490.83	233.00
2837	238.9	297.08973	2.751	5'-Methylthioadenosine	C11H15N5O3S	ION=[M+H]+	2457-80-9	13825.85	12885.00	19802.00	14398.67
2838	242.35	260.13719	3.545	Gaburedin B	C11H20N2O5	ION=[M+H]+		71710.77	71631.00	118912.17	74944.67
2839	245.91	244.17894				ION=[M+H]+		24684.15	4144.50	7386.33	14776.67
2840	246.19	297.08937	13.632	5'-Methylthioadenosine	C11H15N5O3S	ION=[M+H]+	2457-80-9	21.38	2533.00	241.50	250.67
2841	259.06	504.36612				ION=[M+H+H]2+		0.00	64750.83	0.00	0.00
2842	262.5	750.38572				ION=[M+H+H]2+		1303.85	2420.33	461.17	847.67
2843	265.89	292.17862	13.122	Carteolol	C16H24N2O3	ION=[M+H]+	51781-06-7	3116.46	563.83	174.00	301.33
2844	268.64	244.17899				ION=[M+H]+		42734.46	10000.50	11776.50	38785.67
2845	270.62	540.32259				ION=[M+H+H]2+		0.00	1064.50	308.00	592.33
2846	270.22	270.16096				ION=[M+H]+		0.00	511.00	0.00	0.00
2847	270.78	540.3269				ION=[M+H]+		0.00	2092.67	234.50	354.00
2848	271.33	299.25762				ION=[M+H]+		40.00	18969.67	0.00	0.00
2849	275.28	306.19444				ION=[M+H]+		19589.85	88116.00	6.00	71.00
2850	279.4	115.06266	4.059	Proline	C5H9NO2	ION=[M+H]+		234.92	337.67	2267.17	21435.67

2851	279.25	812.40734				ION=[M+H]+		14.77	71.67	1918.00	14691.67
2852	278.19	292.14194	8.631	Filaminast	C15H20N2O4	ION=[M+H]+	141184-34-1	748.00	12702.33	599.17	407.67
2853	281.75	357.20064				ION=[M+H]+		597.08	259.83	100.83	3595.00
2854	282.51	750.38568				ION=[M+H+H]2+		527.23	2297.83	4040.33	907.00
2855	283.67	328.14301	19.59	Dnc008536	C18H20N2O4	ION=[M+H]+		5332.46	283.33	767.17	3895.67
2856	286.11	306.19398				ION=[M+H]+		15335.23	378267.33	0.00	0.00
2857	290.89	341.23171	16.546	Diprotin A	C17H31N3O4	ION=[M+H]+		4307.69	286.50	1610.33	2464.67
2858	293.35	115.06267	9.835	Proline	C5H9NO2	ION=[M+H]+		337.69	174.17	1247.83	8493.67
2859	295.3	325.17387				ION=[M+H]+		1170.62	4378.83	1480.83	689.33
2860	296.21	278.16301	7.759	Santacruzamate A	C15H22N2O3	ION=[M+H]+		14683.38	1590.67	5253.67	8379.33
2861	295.39	330.12964	17.874	4-(2-Hydroxyethyl)-2-methoxyphenyl beta-D-glucopyranoside	C15H22O8	ION=[M+H]+		877.69	3639.67	1493.00	602.67
2862	296.75	333.24201				ION=[M+H]+		0.00	8129.83	0.00	0.00
2863	296	299.25744				ION=[M+H]+		0.00	1939.00	0.00	0.00
2864	295.67	132.07827	3.29	2-Hydroxycaproic acid	C6H12O3	ION=[M+H]+		1095.08	5953.83	2062.50	690.67
2865	299.54	246.13692	9.686	4-Acetoxy DMT	C14H18N2O2	ION=[M+H]+		3000.31	2145.83	2825.67	1881.67
2866	301.29	328.14251	19.512	Dnc008536	C18H20N2O4	ION=[M+H]+		4666.15	474.83	1548.33	3496.67
2867	302.72	456.33097				ION=[M+H]+		2932.15	0.00	0.00	0.00
2868	302.83	278.16282	8.35	Santacruzamate A	C15H22N2O3	ION=[M+H]+		16566.62	3514.00	4561.00	17959.33
2869	305.41	459.26883				ION=[M+H]+		960.00	3339.33	349.00	171.67
2870	305.6	939.51225				ION=[M+H+H]2+		18448.00	393.17	2690.33	1175.67
2871	311.33	308.14761				ION=[M+Na]+		463.85	3420.83	415.50	940.00
2872	312.46	439.14134				ION=[M-H2O+H]+		922.15	7519.17	265.33	150.00
2873	311.18	132.0783	5.382	2-Hydroxycaproic acid	C6H12O3	ION=[M+H]+		488.15	2730.83	264.00	739.67
2874	311.51	115.06263	3.749	Proline	C5H9NO2	ION=[M+H]+		100.15	320.67	1624.67	17769.00
2875	311.79	325.17392	7.065	Cycloxydim	C17H27NO3S	ION=[M+H]+	101205-02-1	575.38	3249.83	260.17	853.67
2876	311.02	443.29887				ION=[M+H]+		71623.85	0.00	0.00	0.00
2877	313.99	333.24197				ION=[M+H]+		0.00	10506.00	0.00	0.00
2878	314.78	278.16296	2.076	Santacruzamate A	C15H22N2O3	ION=[M+H]+		35347.38	7353.50	10617.83	25943.67
2879	315.25	301.2002	14.87	Phormidinine B	C19H27NO2	ION=[M+H]+		2777.54	782.50	1895.33	3079.33
2880	316.77	1432.71918				ION=[M+H+H2]3+		10.92	1921.67	21.83	0.00
2881	318.15	1144.56889				ION=[M+H+H]2+		1706.15	1254.33	439.67	518.00
2882	318.87	278.16333	1.245	Santacruzamate A	C15H22N2O3	ION=[M+H]+		28574.15	1347.33	2641.83	5024.33
2883	319.5	485.35586				ION=[M+H]+		2550.31	0.00	0.00	0.00
2884	320	456.33013				ION=[M+H]+		2117.54	9.00	10.00	0.00
2885	323.06	939.50981				ION=[M+H]+		20.00	0.00	725.67	5563.33
2886	321.98	387.24644				ION=[M+H]+		3879.85	1381.00	651.17	5635.00
2887	322.96	115.0628	9.508	Proline	C5H9NO2	ION=[M+H]+		82.46	88.00	806.67	7823.00
2888	323.62	1385.73897				ION=[M+H+H2]3+		83.69	1891.00	113.17	51.33
2889	326.09	222.14686	11.671	Tetraglyme	C10H22O5	ION=[M+H]+		14080.92	20429.33	39305.83	18548.67
2890	326.07	240.16177				ION=[M+H]+		1842.62	2747.83	6749.00	3400.33
2891	329.2	347.25749				ION=[M+H]+		0.00	4325.00	10.17	0.00
2892	328.77	364.12664				ION=[M+H]+		724.00	2765.33	607.33	70.00
2893	335.31	1385.73962				ION=[M+H+H2]3+		292.00	3119.17	237.67	77.67
2894	335.64	542.36592				ION=[M+H]+		97524.31	0.00	0.00	253.33
2895	337.73	405.26246	13.493	N2-(4-aminocyclohexyl)-n6-benzyl-9-cyclopentyl-9h-purine-2,6-diamine	C23H31N7	ION=[M+H]+		0.00	33623.17	15.83	50.33
2896	337.92	421.13124				ION=[M+H]+		610.15	4834.67	199.83	250.67
2897	341.42	1432.72905				ION=[M+H+H]2+		482.77	1145.50	292.17	345.00
2898	342.3	317.1745				ION=[M+H]+		2922.62	542.50	933.33	4080.67
2899	345.9	1144.57325				ION=[M+H+H]2+		570.62	2051.00	1455.67	651.00
2900	350.38	445.25375				ION=[M+H]+		1053.38	184.17	69.17	1166.33
2901	352.3	670.25082				ION=[M+H]+		38827.08	118595.17	130673.50	80561.33
2902	353.4	266.17283	13.246	Pentaglyme	C12H26O6	ION=[M+H]+	1191-87-3	2448.46	2733.83	4930.33	2787.00
2903	353.31	317.17459				ION=[M+H]+		4458.00	894.67	1607.33	3934.67
2904	354.22	576.35347				ION=[M+H]+		13626.31	0.00	0.00	0.00
2905	355.29	1634.86015				ION=[M+H+H]2+		1192.77	65.50	153.00	48.33
2906	355.81	346.11659				ION=[M+H]+		8619.85	30966.17	6580.00	717.33
2907	360.19	585.19663				ION=[M+H]+		10671.54	4905.83	2485.67	7656.67
2908	362.01	185.95013				ION=[M+H]+		3314.46	2198.83	2848.00	5647.33
2909	362.11	652.23953				ION=[M+H]+		4518.00	6755.50	1835.17	424.33
2910	367.07	585.19637	15.733	Cadazolid	C29H29F2N3O8	ION=[M+H]+	1025097-10-2	540937.54	330848.33	204058.67	402183.33
2911	369.86	437.16278				ION=[M+H]+		18.15	4396.17	0.00	0.00
2912	371.29	670.25143				ION=[M+H]+		5784.15	4084.50	1332.17	2102.33
2913	371.92	157.95416				ION=[M+H]+		9094.62	5379.17	5516.17	6549.67
2914	372.22	680.34081				ION=[M+H]+		2252.00	13031.50	2502.33	621.33
2915	373.44	601.19184				ION=[M+H]+		1646.92	684.83	8655.50	16871.00
2916	377.72	682.25106				ION=[M+H]+		116072.77	58886.17	22552.17	43175.67
2917	380.64	501.27996				ION=[M+H]+		3398.00	206.67	8386.00	3894.67
2918	380.88	437.16125	14.046	3-[3-(dimethyl-1,3-thiazol-5-yl)-4-oxo-2h,4h-indeno[1,2-c]pyrazol-5-yl]-1-(4-methylpiperazin-1-yl)urea	C21H23N7O2S	ION=[M+H]+		14.00	9489.83	25.67	0.00
2919	381.06	346.11784				ION=[M+H]+		1163.38	2810.83	698.00	866.33
2920	384.14	216.10182	9.86	4-Oxosebacic Acid	C10H16O5	ION=[M+H]+	114212-45-2	1668.00	946.00	1022.50	3107.33

2921	387.5	232.13243	14.356	1,5-Dimethyl-N-[(1-methylpyrrol-2-yl)methyl]pyrazole-3-carboxamide	C12H16N4O	ION=[M-H2O+H]+		1685.54	4255.00	5660.17	5339.67
2922	387.16	185.94976				ION=[M+H]+		12460.92	5939.67	5356.33	8901.33
2923	387.73	435.14681				ION=[M+H]+		109.54	5569.50	14.33	53.33
2924	388.98	157.95388				ION=[M+H]+		6331.38	9675.17	7454.17	10577.00
2925	391	1400.73072				ION=[M+H+H2]3+		0.00	9.33	52254.67	9.00
2926	389.77	1400.73883				ION=[M+H+H]2+		192.31	27.17	6669.83	205.67
2927	391.12	601.19144				ION=[M-H2O+H]+		4727.85	1869.50	888.50	4403.00
2928	391.34	160.11061	7.359	2-Butoxyethyl acetate	C8H16O3	ION=[M+H]+		1330.15	2257.83	3234.00	6812.33
2929	393.46	196.11163	17.937	(Z)-4-hydroxy-4-methyl-2-(1-hexenyl)-2-butenolide	C11H16O3	ION=[M+H]+		1969.08	3572.67	2078.67	1522.33
2930	394.79	684.26516				ION=[M+H]+		1323037.85	582287.33	1133030.17	1050993.33
2931	395.75	700.26272				ION=[M+H]+		5018.62	2369.17	4479.17	4242.00
2932	395.63	256.09688	10.388	Ethyl (3-cyano-1H-1,5-benzodiazepin-4-yl)carbamate	C13H12N4O2	ION=[M+H]+		6121.54	6844.50	71.33	0.00
2933	395.16	435.14726				ION=[M+H]+		0.00	2593.33	20.33	0.00
2934	395.63	182.09533	8.805	Mephenesin	C10H14O3	ION=[M+H]+	59-47-2	1187.38	3889.50	401.17	643.33
2935	404.7	407.20575				ION=[M+H]+		6602.00	685.83	5760.67	3199.00
2936	405.27	272.17321	3.424	Elaiomycin D	C13H24N2O4	ION=[M+H]+		1237.38	274.67	52590.50	172.67
2937	409.32	274.1308				ION=[M+H]+		2033.38	1568.67	1371.50	1299.67
2938	409.65	184.10914	7.027	3-(1-Hydroxy-2-methylbutyl)-4-methyl-2(5H)-furanone)	C10H16O3	ION=[M+H]+		4839.54	5357.50	4204.83	5084.67
2939	411.24	318.12219				ION=[M+H]+		616.15	2514.50	515.83	607.00
2940	412.39	162.12616	6.083	4-(3-hydroxybutoxy)butan-2-ol	C8H18O3	ION=[M+H]+		4779.08	3882.67	2101.83	8126.33
2941	415.61	256.12285	12.448	N-[4-(benzyloxy)phenyl]glycinamide	C15H16N2O2	ION=[M+H]+		2402.00	3903.00	3124.00	4227.33
2942	416.12	407.20564				ION=[M+H]+		3662.00	243.67	3048.50	1599.00
2943	423.64	318.12294	13.103	2-(4-hydroxy-5-phenyl-1h-pyrazol-3-yl)-1h-benzoimidazole-5-carboxamidine	C17H14N6O	ION=[M+H]+		285.54	3096.67	88.17	230.00
2944	427.47	157.95414				ION=[M+H]+		3158.46	4180.50	9593.50	5780.33
2945	428.91	245.0849	8.735	5-Fluoro-2'-deoxycytidine	C9H12FN3O4	ION=[M+H]+		4192.92	1020.83	3774.50	5508.00
2946	432.15	175.96578				ION=[M+H]+		6591.23	5972.33	5518.50	5156.67
2947	435.26	320.13841				ION=[M-H2O+H]+		0.00	9598.50	0.00	0.00
2948	436.16	303.11741	9.602	8-[(2,5-dimethoxyphenyl)methyl]-2-fluoro-9h-purin-6-amine	C14H14FN5O2	ION=[M+H]+		689.69	21105.83	1010.67	464.33
2949	434.75	198.98312				ION=[M+H]+		4737.69	4523.33	3237.00	3353.33
2950	435.54	622.26644				ION=[M+H]+		0.00	13546.50	0.00	0.00
2951	437.48	245.08516	10.73			ION=[M+H]+		5288.92	1078.83	4094.33	4995.33
2952	438	226.97806				ION=[M+H]+		4633.85	3530.67	3186.50	3477.00
2953	438	198.98348				ION=[M+H]+		7429.69	4498.83	4846.50	5171.00
2954	438.23	185.95009				ION=[M+H]+		6531.54	12680.33	6843.50	8629.67
2955	440.03	1697.79535				ION=[M+H+H]2+		1197.23	0.00	0.00	37.33
2956	442.71	203.96154				ION=[M+H]+		5209.38	5057.17	4493.17	4012.00
2957	444.76	294.14692				ION=[M+H]+		1761.38	1625.83	3739.50	2356.33
2958	444.91	198.98356				ION=[M+H]+		10616.15	5341.33	5064.33	5314.67
2959	446.48	175.96535				ION=[M+H]+		5900.00	6164.33	5421.83	5311.00
2960	444.93	226.97829				ION=[M+H]+		5370.92	3673.50	3158.50	3474.67
2961	450.44	320.1381	7.382	Z-ala-pro-oh	C16H20N2O5	ION=[M+H]+	21027-01-0	1911.23	56984.33	2325.50	845.00
2962	450.53	640.27627				ION=[M-H2O+H]+		40.15	14986.67	0.00	0.00
2963	450.68	303.11406	6.512	8-[(2,5-dimethoxyphenyl)methyl]-2-fluoro-9h-purin-6-amine	C14H14FN5O2	ION=[M+H]+		1134.00	33138.17	1442.50	738.33
2964	454.47	185.94993				ION=[M+H]+		4550.00	5291.00	7259.00	5273.33
2965	453.05	157.95404				ION=[M+H]+		3867.54	4231.00	4552.50	10189.67
2966	453.55	198.98428				ION=[M+H]+		5610.92	5217.33	5047.67	5228.00
2967	456.65	253.94313				ION=[M+H]+		4429.08	3848.67	3657.67	1230.33
2968	458.22	1697.79866				ION=[M+H+H]2+		1053.69	30.17	0.00	103.00
2969	463.16	254.15231	11.897	Tricholactone	C14H22O4	ION=[M+H]+		396.62	3917.00	286.50	389.33
2970	464.88	175.96543				ION=[M-H2O+H]+		4606.15	10619.33	5552.00	6450.00
2971	464.95	185.95003				ION=[M+H]+		9015.23	9523.67	10372.17	5291.67
2972	470.31	320.1381				ION=[M+H]+		155.85	5474.83	145.50	88.67
2973	473.18	198.98306				ION=[M+H]+		6231.38	5499.33	5000.17	5635.00
2974	475.17	203.9617				ION=[M+H]+		6250.15	5026.67	4465.67	4116.33
2975	478.13	253.94295				ION=[M+H]+		3930.77	4032.83	3527.83	1273.67
2976	478	321.12802				ION=[M+H]+		0.00	2179.83	11.17	0.00
2977	478.22	274.13174				ION=[M+H]+		4961.85	1111.00	3066.33	1286.33
2978	480.52	320.13784	16.895	Z-ala-pro-oh	C16H20N2O5	ION=[M-H2O+H]+	21027-01-0	9248.77	20479.17	10470.50	6876.67
2979	479.88	705.28017				ION=[M+H+H]2+		24.00	3576.83	8.67	0.00
2980	482.25	2650.21044				ION=[M+H+H]2+		2315.08	123.33	0.00	0.00
2981	482.66	1398.50145				ION=[M+H]+		28.46	1668.50	0.00	0.00
2982	482.64	2650.20478				ION=[M+H+H2]3+		2655.23	190.17	0.00	0.00
2983	480.89	334.15339	5.523	N-methoxyseptorinol	C17H22N2O5	ION=[M+H]+		7188.77	16002.83	8182.50	5179.00
2984	481.64	185.95018				ION=[M+H]+		5837.69	10969.50	6363.50	7380.33
2985	480.85	157.95399				ION=[M+H]+		2956.15	7484.83	8717.50	2152.67
2986	483.1	198.98274				ION=[M+H]+		6196.15	5280.33	4966.67	5493.33

2987	486.42	203.96128				ION=[M+H]+		6368.77	5268.17	4601.00	4188.00
2988	487.36	934.24325				ION=[M+H]+		330.15	985.17	3875.50	219.33
2989	489.86	175.9656	14.043	Medronic Acid	CH6O6P2	ION=[M+H]+	1984-15-2	7662.62	5416.83	5379.83	5902.33
2990	491.42	334.15348	3.295	N-methoxyseptorinol	C17H22N2O5	ION=[M+H]+		610.31	19050.50	704.33	242.67
2991	492.08	157.95408				ION=[M+H]+		8244.31	8900.50	6823.67	9003.67
2992	494.99	705.27844				ION=[M+H+H]2+		9.69	64785.83	21.17	0.00
2993	495.07	705.28605				ION=[M+H]+		12.62	5378.33	0.00	47.00
2994	495.73	198.98296				ION=[M+H]+		6462.00	5467.33	4986.67	5273.33
2995	497.43	934.24194				ION=[M+H]+		279.38	946.00	3846.83	574.00
2996	500.35	203.96119				ION=[M+H]+		6625.69	5062.00	4705.33	4134.33
2997	502.83	250.11937	15.843	Coenzyme Q1	C14H18O4	ION=[M+H]+		3840.92	1974.50	4926.83	1717.67
2998	503.91	226.15832	10.74	(7E)-(-)-7-ethyl-9-hydroxy-7-undecene-3,6-dione	C13H22O3	ION=[M+H]+		4277.85	218.00	0.00	215.67
2999	505.47	256.12171				ION=[M+H]+		8309.38	2509.67	7216.50	3070.67
3000	506.4	185.95013				ION=[M+H]+		7410.15	4685.00	3001.17	7321.33
3001	508.44	228.13803				ION=[M+H]+		2691.23	945.33	2067.33	3207.67
3002	509.53	208.14759	5.954	Nopyl acetate	C13H20O2	ION=[M+H]+		35961.08	34576.33	1131.50	2474.67
3003	510.51	235.93244				ION=[M+H]+		4267.23	4114.17	4022.67	763.00
3004	513.49	253.94279				ION=[M+H]+		4110.92	4183.50	3021.33	1763.33
3005	514.67	157.9545				ION=[M+H]+		8021.08	3268.83	6408.33	4389.33
3006	513.49	198.98286				ION=[M+H]+		6478.77	5483.17	4645.17	5449.33
3007	514.28	316.14277				ION=[M+H]+		11199.23	7320.50	16940.00	9784.00
3008	516.99	202.15841	6.313	CK13b	C11H22O3	ION=[M-H2O+H]+		1545.38	4062.50	2242.83	3835.00
3009	516.6	276.14773				ION=[M-H2O+H]+		235.69	17942.83	0.00	0.00
3010	519.16	278.15237	13.778	Dibutyl phthalate	C16H22O4	ION=[M+H]+		2479.54	2829.33	683.67	1237.33
3011	519.66	185.95014				ION=[M+H]+		7228.62	8677.67	9378.83	7640.33
3012	525.55	235.93229				ION=[M+H]+		2580.31	3641.50	3493.50	1271.33
3013	525.62	184.14768	9.659	2-Propenoic acid, 2-ethylhexyl ester	C11H20O2	ION=[M+H]+		2464.62	7208.33	3496.67	7935.33
3014	527.99	175.96577				ION=[M+H]+		7297.85	5794.50	5351.50	5499.33
3015	528.48	216.04147	11.469	Bergapten	C12H8O4	ION=[M+H]+	484-20-8	8971.08	8929.17	7258.50	8852.67
3016	528.63	250.15746	11.044	Gemfibrozil	C15H22O3	ION=[M+H]+	25812-30-0	2815.38	2069.67	5172.83	3126.33
3017	529.83	198.98313				ION=[M+H]+		6259.23	5371.50	4669.83	5230.33
3018	530.77	185.95016				ION=[M+H]+		6107.38	4021.67	5607.33	3969.00
3019	531.92	253.94243				ION=[M+H]+		4101.54	3615.83	3720.17	1367.00
3020	531.24	194.05987	4.063	Dimethyl phthalate	C10H10O4	ION=[M+H]+		8384.92	3936.83	4142.33	15641.00
3021	531.32	278.15183				ION=[M+H]+		3196.31	3873.50	786.67	1135.00
3022	535.68	203.96167				ION=[M+H]+		6480.15	5021.00	4379.50	4443.00
3023	536.13	157.95419				ION=[M+H]+		6745.23	8594.17	5437.17	4549.33
3024	539.06	210.16318	7.154	Altretamine	C9H18N6	ION=[M+H]+		406.92	16761.50	85.50	199.67
3025	544.27	198.9831				ION=[M+H]+		6327.54	5377.67	4694.50	5444.33
3026	542.86	185.95027				ION=[M+H]+		2366.15	2744.83	1839.50	7878.00
3027	543.12	226.97723				ION=[M+H]+		4396.31	3415.33	3142.00	3550.33
3028	544.48	175.96581				ION=[M+H]+		7870.15	6154.67	5158.83	6149.00
3029	547.77	226.11978	16.841	1,4-Butanediol dimethacrylate	C12H18O4	ION=[M+H]+	2082-81-7	15247.38	17456.00	15334.33	23799.00
3030	549.6	157.95427				ION=[M+H]+		9906.15	3467.67	8472.00	11259.33
3031	550.3	204.1378	8.036	(3R,5R)-3,5-dihydroxydecanoic acid	C10H20O4	ION=[M+H]+		19075.23	15000.33	9875.67	36338.33
3032	550.4	280.16774	11.405	1,13-dihydroxy-6-methyl-1h,4h,6h,7h,8h,9h,11ah,12h,13h,14h,14ah-cyclopenta[f]oxacyclotridecan-4-one	C16H24O4	ION=[M+H]+	20350-15-6	7398.46	5374.00	265.67	572.00
3033	553.42	198.98309				ION=[M+H]+		5648.15	4900.50	4447.17	5380.33
3034	552.6	185.9502				ION=[M+H]+		8404.77	9168.83	11423.50	7353.67
3035	557.77	206.13519				ION=[M+H]+		2162.77	1743.83	1508.00	5412.33
3036	558.62	235.93215				ION=[M+H]+		4549.08	4504.17	4013.67	1681.00
3037	559.95	226.97758				ION=[M+H]+		4352.46	3510.33	3108.50	3541.67
3038	562.76	157.95393				ION=[M+H]+		8805.23	6970.00	5514.00	8731.67
3039	566.07	206.13261	9.124	Ibuprofen	C13H18O2	ION=[M+H]+		10323.85	4227.83	9423.50	19441.00
3040	566.37	292.20395	15.025	Mueggelone	C18H28O3	ION=[M+H]+		4342.46	2112.17	1999.17	3749.67
3041	567.09	186.12141				ION=[M+H]+		2318.00	4393.00	2894.50	4919.67
3042	568.89	502.27858				ION=[M-H2O+H]+		3037.38	3356.00	3327.67	2565.33
3043	570.35	185.95002				ION=[M+H]+		7632.77	8393.67	9149.00	7417.67
3044	570.56	290.1885	15.337	Octinoxate | Octyl 4-methoxycinnamate	C18H26O3	ION=[M+H]+		3995.69	1917.00	1439.00	3443.00
3045	571.03	274.19351	13.258	Empenthrin	C18H26O2	ION=[M+H]+	54406-48-3	2640.46	1378.17	1299.67	2406.33
3046	574.53	175.96534				ION=[M-H2O+H]+		4064.92	5930.33	4971.83	4482.67
3047	572.45	235.93239				ION=[M+H]+		3265.08	4379.00	3554.33	1342.00
3048	573.11	198.98197				ION=[M+H]+		6556.15	5273.33	4755.67	5541.33
3049	573.83	203.96142				ION=[M+H]+		6461.69	4749.50	4318.17	4336.67
3050	572.6	234.16312	NA	2,10,10-trimethyltricyclo[6.3.0.0π,?]undec-6-ene-6-carboxylic acid	C15H22O2	ION=[M+H]+		2724.62	1484.33	1251.33	2349.00
3051	575.27	260.10172	13.853	(9aR,9bS)-9a-Hydroxy-6,9-dimethyl-3-methylene-3,3a,4,5,9a,9b-hexahydroazuleno[4,5-b]furan-2,7-dione	C15H16O4	ION=[M+H]+		2609.69	2314.50	2308.50	3498.67
3052	577.02	197.98004				ION=[M+H]+		496.77	55.67	52.33	35.33

3053	577.1	262.15678				ION=[M+H]+		2849.85	1335.17	1241.67	3283.33
3054	578.15	280.16827	17.003	1,13-dihydroxy-6-methyl-1h,4h,6h,7h,8h,9h,11ah,12h,13h,14h,14ah-cyclopenta[f]oxacyclotridecan-4-one	C16H24O4	ION=[M+H]+	20350-15-6	7251.08	6413.67	407.17	1137.67
3055	579.08	222.16261				ION=[M+H]+		120.31	2716.17	0.00	0.00
3056	580.28	220.1092	9.611	Benzoic acid, 2-hydroxy-, cyclohexyl ester	C13H16O3	ION=[M-H2O+H]+		22235.08	14832.50	18003.83	5973.67
3057	579.4	1250.73829				ION=[M+H+H]2+		0.00	3059.83	3171.00	978.00
3058	579.45	272.17757				ION=[M+H]+		2226.15	1423.83	5328.50	2954.00
3059	580.99	185.95003				ION=[M+H]+		8218.62	2879.17	3877.33	3856.33
3060	581.74	180.11585	3.751	3-tert-Butyl-4-hydroxyanisole	C11H16O2	ION=[M+H]+		112312.31	69606.17	35251.50	60124.33
3061	582.88	360.22986	7.69	Iloprost	C22H32O4	ION=[M+H]+		6225.08	2722.00	290.83	1298.67
3062	584.73	235.93265				ION=[M+H]+		4236.15	4323.17	3952.50	1758.33
3063	585.53	1222.71178				ION=[M+H+H]2+		27.54	1915.33	6333.67	2584.67
3065	587.59	883.34866				ION=[M+H+H]2+		2860.77	960.83	221.33	940.00
3066	590.63	243.18463	9.38	Cyclohexyl-norstatine	C13H25NO3	ION=[M-H2O+H]+		3561.08	3809.33	2964.00	4239.67
3067	590.78	737.5114	19.95	(-)-Ternatin	C37H67N7O8	ION=[M+H]+		107.08	0.00	0.00	12544.33
3068	591.48	262.15735				ION=[M+H]+		4482.92	2643.33	503.67	425.33
3069	596.95	185.95084				ION=[M+H]+		2407.38	1252.83	5184.83	3203.67
3070	597.65	238.15785	11.695	Saccharomonopyrone B	C14H22O3	ION=[M+H]+		590.77	88375.83	42.17	93.67
3071	598.53	1206.71506				ION=[M+H+H]2+		29.85	2073.67	5483.50	3661.67
3072	599.67	222.16263				ION=[M+H]+		93.69	3242.17	31.00	0.00
3073	601.4	175.9652				ION=[M-H2O+H]+		6944.46	4823.50	8536.17	10681.33
3074	600.47	253.94297				ION=[M-H2O+H]+		3841.85	4616.50	4280.67	1910.67
3075	600.48	198.98262				ION=[M+H]+		6672.46	5089.83	4887.00	5569.67
3076	600.9	252.17259	19.823	Ilicic acid	C15H24O3	ION=[M+H]+	4586-68-9	3229.85	1507.17	99.33	107.67
3077	605.94	1222.71161				ION=[M+H+H]2+		31.85	3637.50	4298.50	28439.33
3078	604.35	1222.72769				ION=[M+H]+		0.00	169.83	409.33	3071.00
3079	605.22	1275.62068				ION=[M+H+H]2+		0.00	69.67	154.67	2824.00
3080	604.76	203.96089				ION=[M-H2O+H]+		9273.54	10777.00	9037.33	7221.00
3081	607.5	262.15733	4.669	7-hydroxy-10-methoxydehydrodihydrobotrydial	C16H22O3	ION=[M+H]+		1188.77	31725.83	312.17	843.67
3082	607.28	883.35055				ION=[M+H+H]2+		4451.23	1575.33	545.50	1538.00
3083	608.18	253.94283				ION=[M+H]+		5571.23	4677.67	3980.33	1847.33
3084	607.92	175.9657				ION=[M+H]+		7987.08	5765.67	4839.67	5508.67
3085	609.66	326.20959				ION=[M-H2O+H]+		7728.62	4101.00	3656.67	9255.33
3086	611.52	1236.72486				ION=[M+H+H]2+		0.00	1684.17	2121.00	8501.33
3087	609.69	1220.6938				ION=[M+H+H]2+		27.69	677.67	500.17	17467.00
3088	617.29	1234.70961				ION=[M+H+H]2+		0.00	635.83	752.67	7528.67
3089	617.43	198.98264				ION=[M+H]+		6380.62	5243.00	4711.17	4915.67
3090	620.7	290.18845	14.406	Octinoxate | Octyl 4-methoxycinnamate	C18H26O3	ION=[M+H]+		9907.69	7434.83	21701.00	14482.00
3091	619.99	737.51122				ION=[M+H]+		0.00	0.00	0.00	7688.00
3092	623	232.14752				ION=[M+H]+		2318.31	1120.17	2099.00	5247.00
3093	623.29	1275.62528				ION=[M+H+H]2+		17.69	6286.50	2186.00	6325.67
3094	623.96	1206.71292				ION=[M+H+H]2+		15.38	3569.83	3144.33	13815.67
3095	623	181.9769				ION=[M+H]+		1300.00	805.00	490.83	368.00
3096	628.31	235.93246				ION=[M+H]+		3923.85	3702.67	4046.67	1235.67
3097	625.71	253.94291				ION=[M+H]+		5279.85	4257.83	3812.00	1699.67
3098	626.81	185.95025				ION=[M+H]+		2734.77	4649.33	7405.33	0.00
3099	627.89	175.96593				ION=[M+H]+		7496.92	5609.33	4598.33	3479.33
3100	627.44	1222.72575				ION=[M+H]+		0.00	3329.00	1288.83	3780.33
3101	626.47	1222.71154				ION=[M+H+H]2+		75.23	20244.67	7195.50	26377.33
3102	626.53	262.15762	13.777	7-hydroxy-10-methoxydehydrodihydrobotrydial	C16H22O3	ION=[M+H]+		515.38	2891.83	220.33	194.00
3103	629.33	248.14205	10.351	Parthenium	C15H20O3	ION=[M-H2O+H]+		160.46	3763.00	111.50	145.00
3104	628.2	1220.73517				ION=[M+H]+		0.00	720.33	321.83	3240.67
3105	629.14	1220.71053				ION=[M+H+H]2+		0.00	7360.33	4018.00	29021.33
3106	628.63	1218.72293				ION=[M+H+H]2+		16.31	831.00	1367.83	2942.33
3107	629.54	335.21	5.239	Bulbiferate A	C19H29NO4	ION=[M-H2O+H]+		5000.77	1113.83	922.33	2245.00
3108	631.18	512.29867	20.488	Muscoride A	C28H40N4O5	ION=[M+H]+		10471.08	6831.33	12583.17	13303.33
3109	633.11	1220.71154				ION=[M+H]+		0.00	2198.83	161.00	2503.67
3110	632.66	552.29245				ION=[M+H]+		2010.31	2159.83	3086.17	1617.67
3111	634.05	1236.72541				ION=[M+H+H]2+		24.31	54588.83	174988.17	507935.33
3112	634.46	1220.6949				ION=[M+H+H]2+		26.15	10131.33	1524.50	23898.00
3113	634.49	252.17278	12.973	Ilicic acid	C15H24O3	ION=[M+H]+	4586-68-9	289.23	3932.17	31.50	95.67
3115	636.27	324.2293				ION=[M+H]+		6020.92	2335.50	6362.50	10292.33
3116	636.59	1234.70672				ION=[M+H+H]2+		26.15	50468.00	80315.33	690134.33
3117	639.53	292.20387	13.683	Mueggelone	C18H28O3	ION=[M-H2O+H]+		31915.23	13125.17	10422.67	18389.33
3118	640.02	332.19646	9.743	Kalkipyrone A	C20H28O4	ION=[M+H]+		4152.92	2966.33	1600.00	488.33
3119	639.55	234.1629	12.383	2,10,10-trimethyltricyclo[6.3.0.0π,?]undec-6-ene-6-carboxylic acid	C15H22O2	ION=[M+H]+		19015.08	6951.50	5825.00	11063.33
3120	639.47	364.22263	28.036	Yoshinone A	C21H32O5	ION=[M+H]+		2678.92	1867.17	2271.33	690.00
3121	640.32	1167.97955				ION=[M+H]+		4854.31	2748.00	13548.17	10394.00

3122	641.12	1250.7539				ION=[M+H]+		0.00	2233.83	14198.83	27757.33
3123	641.99	232.14726	8.691	Mebutamate	C10H20N2O4	ION=[M+H]+		3570.31	1594.33	2756.33	2427.67
3125	643.95	3500.93674				ION=[M+H+H2]3+		11037.38	11995.50	29168.33	10989.00
3126	644.83	198.98272				ION=[M+H]+		6122.62	4181.17	4040.00	2323.33
3127	645.64	1250.59985				ION=[M+H+H]2+		0.00	1097.33	0.00	0.00
3128	649.32	318.22019				ION=[M-H2O+H]+		0.00	0.00	4142.17	0.00
3129	650.15	336.23079	19.372	Debromogrenadadien	C20H32O4	ION=[M+H]+		0.00	0.00	3116.67	0.00
3130	649.6	376.22276				ION=[M+H]+		0.00	0.00	2161.67	0.00
3131	652.15	3500.94096				ION=[M+H+H2]3+		6018.77	33.83	132.33	183.67
3132	650.69	248.14256	12.259	Parthenium	C15H20O3	ION=[M+H]+		2978.77	3871.67	1779.50	4125.33
3133	651.29	1218.71856				ION=[M+H+H]2+		0.00	1506.67	716.17	14949.00
3134	651.34	175.96575				ION=[M+H]+		6576.15	4411.33	4298.00	4097.33
3135	652.77	1167.97733				ION=[M+H]+		1877.38	4349.00	4616.17	210.33
3136	653.46	262.15779	17.633	7-hydroxy-10-methoxydehydrodihydrobotrydial	C16H22O3	ION=[M+H]+		629.08	4304.67	361.50	332.67
3137	655.23	1218.71879				ION=[M+H+H]2+		0.00	9200.33	1413.83	14315.67
3138	656.72	1320.45339				ION=[M+H]+		6853.69	5487.17	30330.50	15714.67
3139	655.85	1320.20271				ION=[M+H]+		6886.31	5599.50	25954.67	19172.00
3140	657.25	308.19915	14.517	Sacrolide A	C18H28O4	ION=[M-H2O+H]+		29901.54	13643.17	11548.83	22300.33
3141	658.38	308.19932	14.766	Sacrolide A	C18H28O4	ION=[M+H]+		9002.31	9122.83	3379.00	8469.00
3142	659.84	1232.74155				ION=[M+H]+		0.00	2045.33	3492.33	77231.33
3143	659.13	1260.70141				ION=[M+H]+		0.00	60.50	13.83	7013.00
3145	663.98	238.15771	16.122	Saccharomonopyrone B	C14H22O3	ION=[M-H2O+H]+		3733.85	2191.33	1291.00	3665.33
3146	662.26	1250.59402				ION=[M+H+H]2+		11.85	2778.17	3231.17	0.00
3147	667.31	1320.20282				ION=[M+H]+		1244.46	1331.00	3702.33	730.33
3148	665.77	1320.45149				ION=[M+H]+		2158.77	1822.33	3360.33	4600.33
3149	666.92	1118.34788				ION=[M+H]+		2650.92	1891.33	8121.33	7009.67
3150	667.02	1118.09757				ION=[M+H]+		2961.23	1569.33	8662.33	8336.67
3151	667.36	234.19927	12.23	Ethanone, 1-(1,2,3,4,5,6,7,8-octahydro-2,3,8,8-tetramethyl-2-naphthalenyl)-	C16H26O	ION=[M+H]+		2055.85	724.17	1704.67	3965.33
3152	666.92	1117.84675				ION=[M+H]+		717.54	0.00	2947.00	1847.33
3154	667.4	2235.69275				ION=[M+H+H]2+		1592.15	1311.17	4151.33	5103.67
3155	670.25	222.16287				ION=[M+H]+		811.08	4660.67	162.00	167.00
3156	676.19	157.95435				ION=[M+H]+		5770.46	2657.50	5898.33	10214.33
3157	678.28	308.1991	14.418	Sacrolide A	C18H28O4	ION=[M-H2O+H]+		5597.08	5235.67	2196.83	5838.67
3158	678.29	468.27253	13.55	Carolacton	C25H40O8	ION=[M+H]+		2428.62	1596.50	4870.67	3937.33
3159	680.03	248.17799	16.598	13-hydroxy-marasm-7(8)-en-5-methoxy Œ≥-acetal	C16H24O2	ION=[M+H]+		3321.38	6039.83	1209.17	2251.67
3160	680.31	306.18318				ION=[M+H]+		5645.08	1834.50	1418.00	5902.00
3161	680.62	1117.84574				ION=[M+H]+		0.00	0.00	1130.17	0.00
3162	680.36	2235.69033				ION=[M+H+H]2+		724.62	1181.17	2500.00	394.33
3163	680.05	1118.34856				ION=[M+H]+		801.23	816.67	4293.67	739.33
3164	681.22	1118.09793				ION=[M+H]+		974.62	1175.17	4557.33	541.67
3165	681.03	676.36852	11.719	Granadaene	C39H52N2O8	ION=[M+H]+		1368.00	164.50	1621.17	3354.33
3166	684.58	1210.66706				ION=[M+H+H]2+		12.92	3981.00	9729.50	0.00
3168	683.59	1248.61183				ION=[M+H+H]2+		0.00	1574.00	2019.83	0.00
3169	684.91	1250.59802				ION=[M+H+H]2+		0.00	6220.17	2419.17	0.00
3170	684.44	302.24479	15.553	Takinolide seco-acid	C17H34O4	ION=[M+H]+		1660.00	327.83	696.33	2060.00
3171	685.42	508.26503				ION=[M+H]+		1898.00	1244.67	4006.00	4832.33
3172	686.75	216.17368				ION=[M+H]+		1863.08	1213.50	1052.17	2893.00
3173	688.12	324.22974	5.551	TOFA	C19H32O4	ION=[M+H]+		13285.08	5094.00	17379.33	33284.67
3174	687.79	232.1832				ION=[M+H]+		2803.85	1088.50	3738.83	6870.67
3175	687.73	486.2829				ION=[M+H]+		2471.69	1038.50	3214.67	5622.00
3176	686.4	128.11974	3.649	Sulcatol	C8H16O	ION=[M+H]+		2916.77	1910.50	1645.00	4268.00
3177	692.59	676.36953				ION=[M+H]+		3950.00	340.83	3670.00	9974.67
3178	690.46	238.15707	9.557	Saccharomonopyrone B	C14H22O3	ION=[M+H]+		6885.08	10433.67	2564.67	3376.00
3179	690.72	1260.62527				ION=[M+H+H]2+		0.00	2333.67	8276.17	0.00
3182	693.89	1248.65691				ION=[M+H]+		24.15	262.33	3879.33	0.00
3183	700.52	1268.64258				ION=[M+H]+		0.00	14.67	1985.83	0.00
3184	702.15	216.17364	10.093	12-Hydroxydodecanoic acid	C12H24O3	ION=[M-H2O+H]+		4459.23	3122.33	2415.67	7749.33
3185	702.31	110.10896	3.264	Cyclooctene	C8H14	ION=[M+H]+		2402.62	1747.17	1167.33	4252.00
3186	704.31	272.17761	15.173	Galaxolidone	C18H24O2	ION=[M+H]+		4886.62	1259.67	4025.83	14786.67
3187	706.17	1210.66748				ION=[M+H+H]2+		20.31	13470.00	4191.67	0.00
3188	706.67	652.36693				ION=[M+H]+		3662.46	1226.17	2097.67	8407.00
3189	707.31	292.20371	18.458	Mueggelone	C18H28O3	ION=[M+H]+		7256.31	4204.17	3535.17	8572.33
3190	706.93	1248.6151				ION=[M+H+H]2+		16.00	3160.17	720.67	0.00
3191	710.42	310.21455	16.442	15.16-dihydrosacrolide A	C18H30O4	ION=[M+Na]+		8803.08	6704.83	5869.17	5504.33
3192	708.94	266.189	9.961	Tetranor-12(S)-HETE	C16H26O3	ION=[M-H2O+H]+		18784.92	9726.67	7323.33	15528.00
3193	711.09	306.21736	17.871	Oxandrolone | Anavar	C19H30O3	ION=[M+H]+	53-39-4	5143.38	1994.67	3149.67	3560.00
3194	710.9	346.21196	19.633	21-Deoxycortisol	C21H30O4	ION=[M+H]+		2175.54	1071.00	1348.67	732.33
3195	709.36	274.19302	14.593	Empenthrin	C18H26O2	ION=[M+H]+	54406-48-3	8510.46	4546.33	5300.33	9597.67
3196	709.16	1260.63182				ION=[M+H+H]2+		43.08	1377.50	5053.50	0.00
3197	710.68	270.13742	15.227	N-phenyl-N-piperidin-4-ylfuran-2-carboxamide	C16H18N2O2	ION=[M+H]+		1821.69	817.33	610.83	1052.00
3198	715.28	250.19312	12.711	1,4-Benzenediol, 2,5-bis(1,1-dimethylpropyl)-	C16H26O2	ION=[M+H]+		1327.38	1576.50	418.33	1068.67

3199	716.72	326.24574	16.091	Avocadyne 1-acetate	C19H34O4	ION=[M+H]+	24607-06-5	3255.54	1701.17	4658.67	6025.67
3200	717.04	1246.66078				ION=[M+H+H]2+		0.00	2888.50	7424.67	0.00
3201	718.01	470.28822				ION=[M+H]+		3717.23	2176.17	5804.33	6831.67
3202	719.33	272.17751	13.346	Galaxolidone	C18H24O2	ION=[M+H]+		26174.46	8168.50	22214.67	58194.33
3203	720.37	1192.71259				ION=[M+H]+		5.23	194.17	4386.67	0.00
3204	724.98	326.24457	7.074	Avocadyne 1-acetate	C19H34O4	ION=[M+H]+	24607-06-5	15632.62	7618.00	16716.50	41259.67
3205	725.45	234.19908	11.772	Ethanone, 1-(1,2,3,4,5,6,7,8-octahydro-2,3,8,8-tetramethyl-2-naphthalenyl)-	C16H26O	ION=[M+H]+		9409.54	4137.83	9884.00	24710.67
3206	725.6	488.29917	13.996	WSS2221	C26H40N4O5	ION=[M+H]+		3631.54	1628.67	3786.83	8869.33
3207	723.23	510.28084	12.566	Virescenoside Z13	C27H42O9	ION=[M+H]+		3049.23	2229.67	6028.33	7506.33
3208	726.4	505.32537	19.499	AAL Toxin Tb1	C25H47NO9	ION=[M+H]+		10566.00	6946.83	9394.33	16929.00
3209	728.11	322.21449	15.97	Decylubiquinone	C19H30O4	ION=[M-H2O+H]+	55486-00-5	8453.23	2485.17	3424.83	5123.00
3210	728.43	322.21408	18.218	Decylubiquinone	C19H30O4	ION=[M+H]+	55486-00-5	4710.62	1300.50	2050.17	3484.67
3211	729.36	1260.63635				ION=[M+H+H]2+		0.00	724.50	4245.00	0.00
3212	735.01	264.17294				ION=[M+H]+		4587.54	6570.50	551.50	2135.67
3213	735.47	312.22946	18.554	Punaíauic acid	C18H32O4	ION=[M-H2O+H]+		5073.69	1860.67	2835.00	5353.67
3214	736.36	523.3385				ION=[M+H]+		5736.46	5605.00	4252.67	7077.33
3215	741.54	654.38465				ION=[M+H]+		2503.23	1587.83	4297.17	5868.33
3216	740.81	330.27594				ION=[M+H]+		1683.85	1060.83	2695.00	4660.00
3217	745.53	676.36642				ION=[M+H]+		640.31	1236.50	2029.00	1130.33
3218	746.19	334.21254	31.397	Kalkipyrone B	C20H30O4	ION=[M+H]+		3960.31	2060.83	1748.17	2412.67
3219	745.61	710.29937				ION=[M+H+H]2+		0.00	340.17	981.17	0.00
3220	744.93	290.18793	17.698	Octinoxate | Octyl 4-methoxycinnamate	C18H26O3	ION=[M+H]+		3928.31	2119.17	1783.50	3734.67
3221	746.8	250.19338	14.992	1,4-Benzenediol, 2,5-bis(1,1-dimethylpropyl)-	C16H26O2	ION=[M+H]+		6176.77	3179.33	1831.83	5415.67
3222	745.53	492.32868	32.888	(2S)-1-O-Palmitoyl-3-O-?-D-galactopyranosylglycerol	C25H48O9	ION=[M+H]+		1342.00	906.17	2432.50	3357.67
3223	746.44	496.3044	9.157	Malacosterone	C27H44O8	ION=[M+H]+		4887.38	948.00	8307.17	9770.00
3224	748.47	302.24539	14.53	Takinolide seco-acid	C17H34O4	ION=[M+H]+		5416.15	2272.50	2901.33	9888.67
3225	747.39	747.49522				ION=[M+H]+		22.15	18.17	0.00	3465.33
3226	747.43	911.34211				ION=[M+H+H]2+		1864.46	323.83	40.33	225.00
3227	750.28	320.19884	17.715	Agglomerin D	C19H28O4	ION=[M+H]+		3990.00	1108.67	1620.67	2358.00
3228	749.69	851.32612				ION=[M+H+H]2+		2998.92	402.33	397.33	1348.67
3229	752.12	352.2609	4.709	1-[4-(2,4,4-trimethylpentan-2-yl)phenyl]-1,4,7,10-tetraoxaundecane	C21H36O4	ION=[M+H]+		37046.31	6135.67	54709.17	86755.67
3230	752.09	514.31419	12.189	Microsporin B	C28H42N4O5	ION=[M+H]+		6156.15	1127.67	9706.83	15718.33
3231	753.18	294.21954	6.669	9-Oxo-10(E),12(E)-octadecadienoic acid	C18H30O3	ION=[M-H2O+H]+		11876.62	21099.00	7127.33	12745.67
3232	756.46	266.16562	2.151	Phosphoric acid tributyl ester	C12H27O4P	ION=[M+H]+		41343.38	46866.50	35655.00	54955.67
3233	755.47	330.27602	4.642	1-Palmitoylglycerol	C19H38O4	ION=[M+H]+		16264.00	2016.33	12145.67	41787.00
3234	754.87	654.38443	9.485	Kribelloside C	C31H58O14	ION=[M+H]+		6284.77	836.33	4616.33	16239.67
3235	756.4	266.18522	16.819	Tetranor-12(S)-HETE	C16H26O3	ION=[M+H]+		1831.54	689.33	59.67	0.00
3236	755.53	492.32902	9.102	(2S)-1-O-Palmitoyl-3-O-?-D-galactopyranosylglycerol	C25H48O9	ION=[M+H]+		6076.15	718.17	4475.33	15157.33
3237	754.74	710.29682				ION=[M+H+H]2+		3125.69	755.67	2690.33	5676.67
3238	757.91	330.18205				ION=[M+H]+		2093.38	1210.83	1179.33	1631.33
3239	761.37	851.32594				ION=[M+H+H]2+		2936.15	393.17	1957.33	0.00
3240	761.48	911.34259				ION=[M+H+H]2+		1951.23	241.00	30.17	77.67
3241	763.77	472.30342	18.577	Cordyglycoside A	C25H44O8	ION=[M+H]+		4784.92	2313.67	8554.50	14227.33
3242	764.47	316.20174				ION=[M+H]+		2130.62	1451.00	1408.17	1050.67
3243	765.91	276.20839				ION=[M+H]+		13779.38	7276.17	6454.50	13739.33
3244	770.09	342.21946	8.234	Trimegestone	C22H30O3	ION=[M+H]+	74513-62-5	4607.23	2695.50	11660.83	11442.00
3245	768.56	266.18246	11.504	Desipramine	C18H22N2	ION=[M+H]+		2790.15	393.17	478.50	3874.00
3246	771.06	328.26042	17.221	Valsafungin B	C19H36O4	ION=[M+H]+		39809.69	13975.17	40230.50	129607.33
3247	770.71	490.31402	19.655	Sapacitabine	C26H42N4O5	ION=[M+Na]+	151823-14-2	4594.77	2568.17	8695.17	12253.67
3248	771.35	236.21491	4.136	8-Cyclohexadecen-1-one	C16H28O	ION=[M+H]+		6444.92	2389.00	5845.00	23389.67
3249	771.44	254.2277	14.87	(E)-11-Tetradecen-1-ol acetate	C16H30O2	ION=[M+H]+	33189-72-9	1248.00	583.17	1489.00	4237.67
3250	771.89	707.50562				ION=[M+H]+		399.23	0.00	0.00	481.33
3251	770.37	707.53555	21.488	Triterpene	C41H73NO8	ION=[M+H]+		9530.62	83954.50	181057.83	20118.67
3252	770.73	479.30107				ION=[M+H]+		393.69	117.83	406.00	3723.67
3253	774.73	292.20343	24.19	Mueggelone	C18H28O3	ION=[M+H]+		4484.46	2309.33	1889.33	4285.00
3254	774.82	266.15131	14.072	Verrucarol	C15H22O4	ION=[M+H]+		5098.62	5897.17	6294.33	3696.33
3255	776.52	244.16803	10.891	(-)-ethyl homononactate	C13H24O4	ION=[M+H]+		4464.00	4643.50	4403.33	7055.00
3256	777.88	252.20921	16.167	Wortmannine F	C16H28O2	ION=[M+H]+		540.77	2160.50	343.00	566.67
3257	777.92	586.45995				ION=[M+H]+		2109.23	970.67	3078.33	3156.00
3258	779.25	707.53625	24.336	Triterpene	C41H73NO8	ION=[M+H]+		77210.62	252.67	516.50	0.00
3259	780.95	302.18561	19.053	Formestane	C19H26O3	ION=[M+H]+	566-48-3	2444.77	1640.67	1716.83	753.33
3260	779.93	262.19356	15.693	PI-200	C17H26O2	ION=[M+H]+		7586.15	3687.00	6827.00	4909.67
3261	782.07	346.21206	15.976	Pantothenyl-aminoethanol-11-pivalic acid	C16H30N2O6	ION=[M+H]+		13969.38	6836.00	7936.00	5546.00
3262	783.54	324.22983	18.504	TOFA	C19H32O4	ION=[M+H]+		5460.62	1688.67	2334.50	6005.00
3263	784.9	294.2193	3.826	9-Oxo-10(E),12(E)-octadecadienoic acid	C18H30O3	ION=[M-H2O+H]+		79063.69	41682.33	38164.67	86073.33
3264	785.02	470.32352	16.622	Gephyronic acid	C26H46O7	ION=[M+Na]+		2815.54	2428.83	2034.83	2859.67
3265	786.04	298.19336	11.382	Norethisterone	C20H26O2	ION=[M+H]+	68-22-4	3982.15	1419.67	4820.00	8690.67
3266	785.74	268.20358	6.424	Brd-a41145729-001-02-7	C16H28O3	ION=[M+H]+	207597-75-9	2811.08	14030.00	1336.17	2427.33
3267	785.51	222.19927	14.343	Alpha-bisabolol	C15H26O	ION=[M+H]+		137.54	1801.67	67.67	68.33

3268	786.4	498.31967				ION=[M+H]+		1549.38	461.50	3906.83	4100.67
3269	788.35	479.30099				ION=[M+H]+		2564.00	528.17	4521.00	4307.00
3270	789.92	332.19593	47.27	Kalkipyrone A	C20H28O4	ION=[M+H]+		3275.69	1695.50	2076.17	2413.33
3271	792.34	354.27655				ION=[M+H]+		9067.08	2515.33	20444.33	31599.33
3272	792.2	516.32973	19.17	IC202C	C23H44N6O7	ION=[M+H]+		2826.77	599.83	5051.50	7273.67
3273	790.41	382.27202	14.801	Misoprostol	C22H38O5	ION=[M+Na]+	59122-46-2	3399.69	2700.00	1973.17	6219.00
3274	794.01	360.22618				ION=[M+H]+		2090.62	1510.33	1448.67	3634.67
3275	794.43	314.18607	19.596	Estradiol	C20H26O3	ION=[M+H]+	4245-41-4	9738.92	7354.00	6333.00	7219.00
3276	795.51	292.20381	13.483	Mueggelone	C18H28O3	ION=[M+H]+		18699.38	10036.83	9033.17	27026.00
3277	796.97	586.46038				ION=[M+H]+		1747.85	543.00	1655.00	1779.33
3278	803.17	342.14707	5.958	Dipropyleneglycol dibenzoate	C20H22O5	ION=[M+Na]+		16457.85	18137.00	18410.67	33413.67
3279	804.08	792.50208				ION=[M+H+H]2+		800.15	278.17	1790.83	0.00
3280	809.57	730.32468				ION=[M+H]+		3934.31	2196.00	39.50	650.00
3281	810.09	523.33721				ION=[M+H]+		1959.23	0.00	4397.17	0.00
3282	812.15	300.13387	3.24	(1S,3R,4S)-1-(4'-hydroxy-phenyl)-3,4-dihydro-3,4,5-trimethyl-1H-2-benzopyran-6,8-diol	C18H20O4	ION=[M+H]+		19473.69	20244.00	21097.33	12944.67
3283	812.96	278.15182	13.313	Dibutyl phthalate	C16H22O4	ION=[M+H]+		24094.15	19634.67	21852.33	53943.00
3284	817.65	474.31918				ION=[M+H]+		8404.31	4279.83	21875.17	26782.33
3285	818.12	330.2769	2.519	1-Palmitoylglycerol	C19H38O4	ION=[M+H]+		11477.69	4828.50	18016.00	34958.33
3286	821.11	296.23441	9.275	7-methoxy-9-methylhexadecadienoic acid	C18H32O3	ION=[M-H2O+H]+		25946.77	11191.83	13144.33	24162.33
3287	819.58	492.32923	4.49	(2S)-1-O-Palmitoyl-3-O-?-D-galactopyranosylglycerol	C25H48O9	ION=[M+Na]+		1854.31	1200.17	6259.33	5673.33
3288	819.6	270.21956	1.232	Malyngolide	C16H30O3	ION=[M+H]+		964.77	3575.33	1218.00	2122.67
3289	821.16	393.18269				ION=[M+H]+		2335.38	1621.33	1441.00	1369.00
3290	820.44	566.25613				ION=[M+H+H]2+		1990.46	2096.83	3727.00	3795.00
3291	819.68	1040.57515				ION=[M+H+H]2+		647.23	94.50	1111.00	1817.67
3292	819.23	312.26645	15.794	Ricinoleic acid methyl ester	C19H36O3	ION=[M+H]+		4747.54	2583.17	5739.67	11645.67
3293	820.96	436.36714				ION=[M+H]+		1056.92	2765.83	445.83	1865.33
3294	819.68	589.27236				ION=[M+H+H]2+		1110.77	1022.67	1686.83	2415.33
3295	821.1	523.33603				ION=[M+H]+		19528.31	21938.67	20178.33	24894.33
3296	823.76	290.22425				ION=[M+H]+		4873.85	7318.33	4634.50	4503.00
3297	824.83	280.20436				ION=[M+H]+		3828.77	1131.83	1596.50	1952.00
3298	824.99	302.18601	16.616	Formestane	C19H26O3	ION=[M+H]+	566-48-3	3737.08	1260.83	1321.50	767.67
3299	827.06	589.27185				ION=[M+H+H]2+		2978.92	1863.17	6221.17	5763.00
3300	826.35	514.31197				ION=[M+H]+		6552.15	4477.50	17830.50	16903.00
3301	827.67	566.25537				ION=[M+H+H]2+		3172.46	1381.50	6766.00	5814.67
3302	827.31	330.27662	9.296	1-Palmitoylglycerol	C19H38O4	ION=[M+H]+		53626.46	12763.00	70909.33	175997.00
3303	827.71	492.32957	6.297	(2S)-1-O-Palmitoyl-3-O-?-D-galactopyranosylglycerol	C25H48O9	ION=[M-H2O+H]+		5746.62	1077.50	7700.83	28698.67
3304	827.63	388.1051				ION=[M+H]+		7750.77	8853.17	2556.83	8485.33
3305	828.43	324.21149				ION=[M+H]+		2413.69	446.50	2518.33	7316.33
3306	828.38	238.23014	10.13	Bombykol	C16H30O	ION=[M+H]+		4807.08	1351.17	6266.83	16917.00
3307	827.11	730.32011				ION=[M+H]+		2401.69	592.00	16.33	152.67
3308	828.4	736.57468				ION=[M+H]+		0.00	8279.50	0.00	0.00
3309	827.46	1040.57202				ION=[M+H+H]2+		2914.15	931.50	4493.83	6944.00
3310	829.52	792.49985				ION=[M+H+H]2+		1147.23	480.17	1498.17	0.00
3311	830.68	548.24322	34.646	Bistratamide I	C25H36N6O6S	ION=[M+H]+		6258.92	723.00	1482.33	4484.67
3312	833.31	294.21935	14.228	9-Oxo-10(E),12(E)-octadecadienoic acid	C18H30O3	ION=[M-H2O+H]+		11337.38	4109.83	5538.17	13459.33
3313	835.11	294.21924	10.236	9-Oxo-10(E),12(E)-octadecadienoic acid	C18H30O3	ION=[M+H]+		23128.62	8357.33	10648.50	27574.00
3314	836.2	255.90937				ION=[M+H]+		2534.15	2188.17	1503.67	138.33
3315	839.35	688.30786				ION=[M+H]+		2288.92	690.17	0.00	189.00
3316	840.59	612.47714				ION=[M+H]+		1271.54	864.67	2466.33	0.00
3317	843.92	324.21037				ION=[M+H]+		12203.23	3435.50	13573.67	28049.67
3318	846.78	750.58944				ION=[M+H]+		0.00	9000.83	0.00	30.33
3319	846.92	830.44222				ION=[M+H+H]2+		1380.62	750.50	1930.83	0.00
3320	848.79	330.21667	19.518	17alpha-Hydroxyprogesterone	C21H30O3	ION=[M+H]+	68-96-2	8763.85	6121.83	7731.33	4300.33
3321	848.9	538.40752				ION=[M+Na]+		4006.31	1161.67	955.50	495.00
3322	849.23	290.2245	2.487	Androstanolone	C19H30O2	ION=[M+H]+	521-18-6	45972.92	31212.00	43499.83	52189.33
3323	849.7	792.50027				ION=[M+H+H]2+		1082.15	318.50	1667.17	0.00
3324	850.14	752.54756				ION=[M+H]+		918.46	310.83	1596.83	1595.67
3325	850.2	255.90943				ION=[M+H]+		2776.46	2113.00	1329.83	293.33
3326	851.36	282.21925	9.458	Pseudopyronine B	C17H30O3	ION=[M+H]+		2700.00	4408.00	766.67	1305.33
3327	854.06	590.49324				ION=[M+Na]+		2235.38	884.33	3259.67	165.00
3328	852.47	494.38103				ION=[M+Na]+		4560.00	1143.33	1091.83	575.00
3329	854.46	402.2247	10.129	1,2,3-Propanetricarboxylic acid, 2-(acetyloxy)-, 1,2,3-tributyl ester	C20H34O8	ION=[M+Na]+		6925.85	6624.33	6222.17	12244.67
3330	856.76	450.35495	4.968	Poly THF n6	C24H50O7	ION=[M+Na]+		6718.62	1338.50	1303.00	1007.67
3331	856.56	738.5905				ION=[M+H]+		0.00	3400.33	0.00	0.00
3332	858.37	328.20106				ION=[M+H]+		10442.92	7610.00	10591.67	6393.33
3333	858.41	306.21925	10.783	Oxandrolone | Anavar	C19H30O3	ION=[M+H]+	53-39-4	13623.38	11976.67	12399.17	15562.33
3334	858.94	830.44168				ION=[M+H+H]2+		1802.15	768.50	2117.33	0.00
3335	860.77	406.32869				ION=[M+Na]+		9084.46	1265.50	1346.00	751.67
3336	861.16	750.59081				ION=[M+H]+		0.00	6477.67	0.00	0.00

3337	861.88	296.23418				ION=[M+H]+		2736.15	2851.83	1231.67	1779.67
3338	864.13	776.54463				ION=[M+H]+		1721.69	1025.33	2576.67	0.00
3339	863.81	592.50793				ION=[M+H]+		2138.15	1310.00	5246.00	416.00
3340	864.51	430.30415				ION=[M+H]+		1632.62	328.33	1085.83	448.00
3341	865.33	362.30332				ION=[M+Na]+		9681.85	1675.50	1506.33	1413.33
3342	865.56	670.29878				ION=[M+H]+		3081.69	2255.50	75.00	777.00
3343	869.11	255.90915				ION=[M+H]+		2591.08	2054.17	1279.17	591.67
3344	870.51	337.33434	10.234	Erucamide	C22H43NO	ION=[M+H]+		25197.38	23924.50	17366.33	508.00
3345	871.08	318.2772	18.421	Tridihexethyl	C21H36NO	ION=[M+Na]+		8663.85	1806.50	1751.50	1850.00
3346	873.75	590.49033				ION=[M+H]+		787.69	1051.00	1892.67	1577.00
3347	873.91	654.27071				ION=[M+H]+		5122.62	1364.50	352.17	1174.33
3348	875.28	430.30391				ION=[M+H]+		4255.85	636.33	731.67	2706.00
3349	877	283.90348				ION=[M+H]+		3840.46	3598.00	1866.17	416.33
3350	879.29	274.25116				ION=[M+H]+		3411.23	676.50	749.17	1668.33
3351	879.52	776.54477				ION=[M+H]+		1675.54	923.17	3650.50	508.33
3352	879.89	337.33364	5.154	Erucamide	C22H43NO	ION=[M+H]+		0.00	0.00	3391.00	282.00
3353	884.59	592.50721				ION=[M+H]+		2613.85	1508.67	3842.00	706.67
3354	884.71	638.27566				ION=[M+H]+		3664.77	2395.00	473.00	1478.67
3355	887.49	403.31115				ION=[M+H]+		3109.08	1460.33	3921.00	3256.33
3356	887.98	774.531				ION=[M+H]+		1231.85	785.50	2514.67	2426.67
3357	891.43	337.33431	18.84	Erucamide	C22H43NO	ION=[M+H]+		2039.85	6574.33	0.00	12887.67
3358	891.72	308.235				ION=[M+H]+		15309.69	10347.67	7810.83	12954.00
3359	891.19	796.514				ION=[M+H]+		1700.62	768.83	3304.00	2551.67
3360	893.63	612.47609				ION=[M+H]+		7389.38	2916.17	12375.17	14198.67
3361	891.2	330.21695				ION=[M+H]+		9996.92	6028.00	7129.50	5358.33
3362	892.35	183.97834				ION=[M+H]+		4657.54	4696.83	3693.50	1395.67
3363	896.39	283.90371				ION=[M+H]+		4116.46	3472.83	1894.67	561.67
3364	897.19	550.41583				ION=[M+H]+		1982.31	1463.83	2437.33	0.00
3365	899.12	698.29794				ION=[M+H]+		5542.77	1973.83	222.67	1482.67
3366	898.55	654.27127				ION=[M+H]+		5772.00	1931.67	763.67	2644.00
3367	897.56	686.29475				ION=[M+H]+		5594.00	1079.17	93.17	346.67
3368	898.48	337.33429	19.015	Erucamide	C22H43NO	ION=[M+H]+		8031.69	9204.50	8349.83	19547.33
3369	898.56	260.8882				ION=[M+H]+		2128.15	1619.83	999.00	105.00
3370	898.88	592.50688				ION=[M+H]+		2478.62	1851.33	3868.83	1309.00
3371	900.2	810.47337				ION=[M+H+H]2+		4104.77	2627.50	5163.50	470.33
3372	901.89	385.30063				ION=[M+H]+		1817.69	3889.17	7012.50	0.00
3373	904.92	183.97857				ION=[M+H]+		3801.38	3755.83	3419.17	1401.00
3374	905.09	595.37688				ION=[M+H]+		2820.00	3598.33	2551.17	3769.00
3375	907.89	670.2722				ION=[M+H]+		1926.92	237.00	118.67	289.00
3376	910.17	403.31137				ION=[M+H]+		24139.23	22812.33	21419.83	26998.33
3377	910.91	656.28462				ION=[M+H]+		3078.15	1618.67	97.33	426.67
3378	912.16	716.3081				ION=[M+H]+		3048.92	482.33	65.33	209.33
3379	914.23	550.41624				ION=[M+H]+		1982.31	1498.67	1677.17	0.00
3380	915	312.26618				ION=[M+H]+		2372.92	1687.50	5290.67	2013.67
3381	914.94	183.97844				ION=[M+H]+		3958.46	3834.17	2774.33	1565.00
3382	914.88	752.54629				ION=[M+H]+		411.69	1219.17	1186.00	194.33
3383	915.7	592.50532				ION=[M+H]+		2257.23	1682.50	922.33	98.00
3384	916.91	695.54657				ION=[M+H]+		3062.46	417.00	221.00	1351.00
3385	917.63	595.37575				ION=[M+H]+		4254.31	3249.00	3530.17	5158.67
3386	917.05	590.49161				ION=[M+H]+		704.15	2445.83	11305.83	597.33
3387	917.18	1561.00687				ION=[M+H+H]2+		250.92	651.00	3056.17	477.67
3388	917.2	808.45743				ION=[M+H+H]2+		151.85	1239.83	8351.33	181.00
3389	921.79	870.57106				ION=[M+H]+		139.69	1146.67	2809.83	66.67
3390	920.01	774.52904				ION=[M+H]+		3977.54	3820.33	8674.50	1126.33
3391	922.85	770.33121				ION=[M+H]+		6863.08	155.50	15452.17	30612.00
3392	924.54	668.28612				ION=[M+H]+		3635.69	1141.83	146.17	822.67
3393	927.31	183.97829				ION=[M+H]+		3889.23	4526.33	2583.33	1409.67
3394	925.68	260.88811				ION=[M+H]+		1517.85	1693.33	464.83	91.67
3395	924.38	810.47052				ION=[M+H+H]2+		2192.31	2977.00	2564.67	2158.33
3396	925.05	752.54628	16.475	(2S)-3-O-?-D-galactopyranosyl-1-O-(9Z.12Z-octadecadienoyl)-2-O-(4Z-hexadecenoyl)glycerol	C43H76O10	ION=[M+H]+		1550.15	1195.17	2352.33	256.33
3397	926.59	312.26642	19.23	Ricinoleic acid methyl ester	C19H36O3	ION=[M+H]+		2999.85	3078.50	3780.33	3595.00
3398	925.34	849.48484				ION=[M+H+H]2+		1773.23	2217.00	2010.33	147.00
3399	926.33	588.42228				ION=[M+H]+		2394.77	910.17	843.83	146.00
3400	927.85	592.5069				ION=[M+H]+		1477.23	2398.17	1854.33	4442.67
3401	929.06	334.25074				ION=[M+H]+		1553.85	1092.33	1970.50	58.00
3402	929.25	808.45749				ION=[M+H+H]2+		12351.85	9160.50	8162.33	142.33
3403	929.1	590.49163				ION=[M+H]+		14696.15	9148.17	9661.17	1810.67
3404	928.98	992.63404				ION=[M+H+H]2+		4436.62	969.67	1022.33	1273.67
3405	930.01	683.54843				ION=[M+H]+		1800.00	146.67	12.33	204.33
3406	928.67	682.30269				ION=[M+H]+		15843.69	6178.00	902.00	5157.67
3407	930.08	283.90388				ION=[M+H]+		2692.00	3087.33	1355.50	587.33
3408	930.25	612.25945	15.161	Roseopurpurin H	C33H40O11	ION=[M+H]+		2162.62	367.50	242.83	3968.67
3409	930.88	649.42974				ION=[M+H]+		1864.31	6521.67	1195.00	1366.67

3410	930.31	670.2703				ION=[M+H]+		1439.38	401.00	102.67	734.67
3411	932.7	642.4695				ION=[M+H]+		1694.92	2422.00	2175.17	3696.00
3412	932.55	616.29066	7.586	YM-47524	C33H44O11	ION=[M+H]+		4753.08	3646.67	299.00	1239.00
3413	933.93	676.39944				ION=[M+H+H]2+		2452.00	3442.67	2307.17	3515.33
3414	934.36	618.47113				ION=[M+H]+		9043.38	10039.67	18049.33	14804.67
3415	934.12	1561.00567				ION=[M+H+H]2+		3919.08	2310.83	3912.67	220.00
3416	935.43	624.26094				ION=[M+H]+		3025.23	316.33	717.50	1167.33
3417	932.74	774.52999				ION=[M+H]+		2798.00	1458.33	1717.00	687.00
3418	935.4	695.54818				ION=[M+H]+		2243.23	106.67	110.33	51.33
3419	935.22	698.29772				ION=[M+H]+		8613.85	3861.33	23.00	1006.33
3420	936.73	849.48555				ION=[M+H+H]2+		3350.92	2291.17	2767.17	9.33
3421	937.2	620.4866				ION=[M+H]+		40632.77	48455.83	48107.50	71124.33
3422	937.69	784.47798				ION=[M+H]+		1252.92	269.17	3868.67	1993.67
3423	938.91	870.57036				ION=[M+H]+		110.15	1164.83	3084.00	79.00
3424	940.84	595.37631				ION=[M+H]+		3457.08	3166.83	3241.33	3102.00
3425	939.34	590.49244				ION=[M+H]+		2356.92	258.17	868.50	191.00
3426	940.2	652.28921				ION=[M+H]+		2644.00	1564.00	83.33	498.00
3427	941.21	628.41703				ION=[M+H]+		2314.92	458.83	1312.33	2449.67
3428	940.92	612.25933				ION=[M+H]+		4222.31	429.17	162.17	2652.00
3429	940.92	744.48463				ION=[M+H]+		6196.15	1508.33	19596.67	10268.33
3430	942.33	649.42979				ION=[M+H]+		748.92	3360.50	146.00	0.00
3431	943.38	683.54849				ION=[M+H]+		1610.62	149.33	12.83	0.00
3432	944.56	390.33295				ION=[M+Na]+		4400.46	1303.83	1132.83	847.33
3433	944.73	992.63513				ION=[M+H+H]2+		4423.08	1209.83	903.50	1403.33
3434	946.38	427.34741				ION=[M+H]+		3130.62	3656.17	2769.50	5843.67
3435	947.97	730.3242				ION=[M-H2O+H]+		3786.15	3765.33	84.50	858.67
3436	946.53	744.48499				ION=[M+H]+		3204.92	845.17	9859.67	5821.33
3437	946.71	618.4699				ION=[M+H]+		944.15	842.00	2594.83	804.00
3438	946.68	668.41334				ION=[M+H]+		3089.23	1358.00	3501.33	2039.00
3439	948.74	445.35775				ION=[M+H]+		3934.46	4205.83	4096.00	6832.33
3440	951.63	346.30721				ION=[M+Na]+		4698.62	1247.83	1178.33	1026.33
3441	952.3	577.36536				ION=[M+H]+		7135.54	7261.17	6559.33	11454.67
3442	951.63	668.28688				ION=[M+H]+		5167.54	1347.00	293.33	841.33
3443	950.38	682.30217				ION=[M+H]+		5825.85	6014.33	626.50	2312.33
3444	952.36	590.49105				ION=[M+H]+		3937.38	4612.67	4948.33	8779.00
3445	951.51	808.45915				ION=[M+H+H]2+		1195.23	3406.67	2769.67	2084.33
3446	951.07	620.48742				ION=[M+H]+		8054.92	3034.00	20019.00	565.67
3447	950.83	676.40027				ION=[M+H+H]2+		2745.85	3199.00	4203.00	202.33
3448	952.14	283.90359				ION=[M+H]+		2712.92	3099.67	1145.50	262.00
3449	952.75	595.37603				ION=[M+H]+		5104.77	4901.33	4261.33	7079.33
3450	953.17	670.29833				ION=[M+H]+		5062.62	2652.33	303.33	1144.67
3451	953.98	642.27053				ION=[M+H]+		3914.62	298.33	455.33	2458.00
3452	954	700.31129	10.546	Tolyporphin B	C38H44N4O9	ION=[M+H]+		1684.15	440.67	14.33	186.67
3453	954.7	652.26239				ION=[M+H]+		3860.00	128.33	304.67	1483.67
3454	969.58	590.49173				ION=[M+H]+		3502.15	2348.50	3750.50	1208.33
