# Supplementary material for: Intra-population genomic diversity of the bloom-forming cyanobacterium, Aphanizomenon gracile, at low spatial scale
Source: ISME Commun. 2023 Jun 7;3:57. doi: 10.1038/s43705-023-00263-3 (PMC10244403; doi:10.1038/s43705-023-00263-3)
Supplement: Supplementary file 6 — Table S4 [file 43705_2023_263_MOESM6_ESM.pdf]

PMC Strains				Gene	Encoding Protein Function	COG category
627	638	644	-	glsF	PFAM Conserved region in glutamate synthase	C - Energy production and conversion
627	-	-	-	ntaA_2	Nitrilotriacetate monooxygenase component A	C - Energy production and conversion
627	-	-	-	ssuD	Alkanesulfonate monooxygenase	C - Energy production and conversion
-	638	-	-	parA	chromosome partitioning	D - Cell cycle control, cell division, chromosome partitioning
627	638	-	649	aroQ	3-dehydroquinate dehydratase	E - Amino acid transport and metabolism
627	638	-	-	cdd	Cytidine deaminase	F - Nucleotide transport and metabolism
-	638	-	-	tktC	Transketolase	G - Carbohydrate transport and metabolism
627	638	-	649	fdtB	Aminotransferase	H - Coenzyme transport and metabolism
627	638	644	-	rpaB	Two-component response regulator	K - Transcription
-	638	644	-	nblR	Response regulator receiver domain	K - Transcription
627	-	644	-	cmr4	CRISPR system Cmr endoribonuclease	L - Replication, recombination and repair
627	-	644	-	xerC	Tyrosine recombinase	L - Replication, recombination and repair
627	-	-	-	cmr6	CRISPR system Cmr subunit	L - Replication, recombination and repair
-	638	644	649	mrr	Type IV methyl-directed restriction enzyme	L - Replication, recombination and repair
-	638	644	-	hup	DNA-binding protein	L - Replication, recombination and repair
-	638	-	-	res	Type III restriction enzyme, res subunit	L - Replication, recombination and repair
-	-	-	649	rnhC	Ribonuclease HIII	L - Replication, recombination and repair
627	638	644	-	murB	UDP-N-acetylenolpyruvoylglucosamine reductase	M - Cell wall/membrane/envelope biogenesis
627	638	644	-	murC	UDP-N-acetylmuramate--L-alanine ligase	M - Cell wall/membrane/envelope biogenesis
627	638	-	649	wbsE	Putative glycosyl transferase	M - Cell wall/membrane/envelope biogenesis
627	-	644	-	kdsA	2-dehydro-3-deoxyphosphooctonate aldolase	M - Cell wall/membrane/envelope biogenesis
627	-	644	-	kdsB	3-deoxy-manno-octulosonate cytidylyltransferase	M - Cell wall/membrane/envelope biogenesis
627	-	644	-	kpsF	Arabinose 5-phosphate isomerase	M - Cell wall/membrane/envelope biogenesis
-	638	-	649	gumK	UDP-glucuronate:glycolipid 2-beta-glucuronosyltransferase	M - Cell wall/membrane/envelope biogenesis
-	-	644	649	mtfB	Glycosyl transferases group 1	M - Cell wall/membrane/envelope biogenesis
627	-	-	-	ssuB	Aliphatic sulfonates import ATP-binding protein	P - Inorganic ion transport and metabolism
627	-	-	-	ssuC	Putative aliphatic sulfonates transport permease protein	P - Inorganic ion transport and metabolism
-	-	644	649	cutA	Divalent-cation tolerance protein	P - Inorganic ion transport and metabolism
627	638	644	-	psaX	Photosystem I 4.8 kDa protein	S - Function unknown
627	638	-	649	wbpD	UDP-2-acetamido-3-amino-2,3-dideoxy-D-glucuronate N-acetyltransferase	S - Function unknown
627	638	-	-	yqcI	Uncharacterized protein	S - Function unknown
627	-	644	649	crm2-2	CRISPR-associated protein Cas10 Cmr2, subtype III-B	S - Function unknown
-	-	644	-	yxaI	Uncharacterized protein	S - Function unknown
-	638	-	649	hsdM	Type I restriction system adenine methylase	V - Defense mechanisms
-	-	644	-	hsdS	Type I restriction modification DNA specificity domain (methylase)	V - Defense mechanisms

PMC Strains				Gene	Biological process / Pathway (from Uniprot)
627	638	644	-	glsF	Amino-acid biosynthesis; Glutamate biosynthesis
627	-	-	-	ntaA_2	-
627	-	-	-	ssuD	Cellular response to sulfur starvation; response to heat
-	638	-	-	parA	Plasmid partitioning
627	638	-	649	aroQ	Amino-acid biosynthesis; Aromatic amino acid biosynthesis
627	638	-	-	cdd	Cytidine deamination; deoxycytidine catabolic process; nucleobase-containing small molecule interconversion
-	638	-	-	tktC	Pentose-phosphate shunt, oxidative branch; reductive pentose-phosphate cycle
627	638	-	649	fdtB	-
627	638	644	-	rpaB	Phosphorelay signal transduction system
-	638	644	-	nblR	Phosphorelay signal transduction system; Biological Process; Regulation of DNA-templated transcription
627	-	644	-	cmr4	-
627	-	644	-	xerC	-
627	-	-	-	cmr6	-
-	638	644	649	mrr	Restriction system
-	638	644	-	hup	DNA condensation
-	638	-	-	res	Toxin-antitoxin system
-	-	-	649	rnhC	DNA replication, removal of RNA primer; Mismatch repair
627	638	644	-	murB	Cell wall biogenesis; peptidoglycan biosynthesis.
627	638	644	-	murC	Cell wall biogenesis; peptidoglycan biosynthesis.
627	638	-	649	wbsE	-
627	-	644	-	kdsA	Carbohydrate biosynthesis; 3-deoxy-D-manno-octulosonate biosynthesis; 3-deoxy-D-manno-octulosonate from D-ribulose 5-phosphate: step 2/3.<br>Bacterial outer membrane biogenesis; lipopolysaccharide biosynthesis.
627	-	644	-	kdsB	Nucleotide-sugar biosynthesis; CMP-3-deoxy-D-manno-octulosonate biosynthesis; CMP-3-deoxy-D-manno-octulosonate from 3-deoxy-D-manno-octulosonate and CTP: step 1/1.<br>Bacterial outer membrane biogenesis; lipopolysaccharide biosynthesis.
627	-	644	-	kpsF	Lipopolysaccharide biosynthesis
-	638	-	649	gumK	Glycan biosynthesis; xanthan biosynthesis.
-	-	644	649	mtfB	Methylation
627	-	-	-	ssuB	Cellular response to sulfur starvation
627	-	-	-	ssuC	Transport
-	-	644	649	cutA	Response to copper ion
627	638	644	-	psaX	Photosynthesis
627	638	-	649	wbpD	Bacterial outer membrane biogenesis; LPS O-antigen biosynthesis.
627	638	-	-	yqcI	-
627	-	644	649	crm2-2	-
-	-	644	-	yxaI	-
-	638	-	649	hsdM	DNA restriction-modification system
-	-	644	-	hsdS	DNA restriction-modification system

PMC Strains				Gene	Comments	Reference
627	638	644	-	glsF	glutamate biosynthesis	
627	-	-	-	ntaA_2	long-chain alkane monooxygenase - Unclassified: metabolism	
627	-	-	-	ssuD	**cellular response to sulfur starvation**	
-	638	-	-	parA	-	
627	638	-	649	aroQ	chorismate biosynthesis	
627	638	-	-	cdd	scavenges exogenous and endogenous cytidine and 2'-deoxycytidine for UMP synthesis	
-	638	-	-	tktC	Carbohydrate biosynthesis	
627	638	-	649	fdtB	-	
627	638	644	-	rpaB	**OmpR/PhoB family - regulation of energy transfer of excitation from phycobilisomes to photosystems**	Ashby MK, et al. FEMS Microbiol Lett. (2022) Aug 27;214(1):25-30.
-	638	644	-	nblR	**OmpR/PhoB family - phycobilisome degradation/acclimation responses after nutrient or high-light stress.**	R. Schwarz, A.R. Grossman. Proc. Natl. Acad. Sci. U. S. A. (1998) 95
627	-	644	-	cmr4	-	
627	-	644	-	xerC	Phage integrase family	
627	-	-	-	cmr6	-	
-	638	644	649	mrr	Mrr is involved in the RecB-dependent high pressure induction of the SOS stress response in E. coli.	
-	638	644	-	hup	-	
-	638	-	-	res	-	
-	-	-	649	rnhC	Endonuclease that specifically degrades the RNA of RNA-DNA hybrids.	
627	638	644	-	murB	peptidoglycan biosynthesis	
627	638	644	-	murC	peptidoglycan biosynthesis	
627	638	-	649	wbsE	-	
627	-	644	-	kdsA	3-deoxy-D-manno-octulosonate biosynthesis - Carbohydrate biosynthesis.	
627	-	644	-	kdsB	CMP-3-deoxy-D-manno-octulosonate biosynthesis - Nucleotide-sugar biosynthesis.	
627	-	644	-	kpsF	Involved in the biosynthesis of K-antigen capsules.	
-	638	-	649	gumK	xanthan biosynthesis - Glycan biosynthesis.	
-	-	644	649	mtfB	-	
627	-	-	-	ssuB	**cellular response to sulfur starvation**	
627	-	-	-	ssuC	**cellular response to sulfur starvation**	
-	-	644	649	cutA	Involved in resistance toward heavy metals.	
627	638	644	-	psaX	**accessory protein potentially involved in chlorophyll stabilization**	Çoruh O, et al. Commun Biol. 2021 Mar 8;4(1):304.
627	638	-	649	wbpD	LPS O-antigen biosynthesis and in Bacterial outer membrane biogenesis.	
627	638	-	-	yqcI	-	
627	-	644	649	crm2-2	-	
-	-	644	-	yxaI	-	
-	638	-	649	hsdM	-	
-	-	644	-	hsdS	-	
